# Supplementary material for: Tailored photoenzymatic systems for selective reduction of aliphatic and aromatic nitro compounds fueled by light
Source: Nat Commun. 2023 Sep 6;14:5442. doi: 10.1038/s41467-023-41194-w (PMC10482925; doi:10.1038/s41467-023-41194-w)
Supplement: Supplementary file 1 — Supplementary Information [file 41467_2023_41194_MOESM1_ESM.pdf]

Supplementary Information

Tailored photoenzymatic systems for selective reduction of aliphatic and aromatic nitro compounds fueled by light

Alejandro Prats Luján,[1] Mohammad Faizan Bhat,[1] Sona Tsaturyan,[1] Ronald van Merkerk,[1] Haigen Fu,[2] Gerrit J. Poelarends[1*]

[1]Department of Chemical and Pharmaceutical Biology, Groningen Research Institute of Pharmacy, University of Groningen, Antonius Deusinglaan 1, 9713 AV Groningen (The Netherlands). E-mail: g.j.poelarends@rug.nl

[2]Department of Chemistry and Chemical Biology, Cornell University, Ithaca, New York 14850, United States.

Table of Contents

Supplementary List of Gene Sequences……………………………………………………….....3

Supplementary Figure 1…..……….…………………………………………………….…….4

Supplementary Figure 2……………………………………………………………………….5

Supplementary Results…………………………………………………………………….....6

Photoenzymatic Transformations……………………………..……………………….....6

Supplementary Tables 1-7………………………………………………...……6

Supplementary Figures 3-7…………………………………...……………13

Supplementary Tables 8-11………..……………………………………………16

Supplementary Figure 8……….…………………………………………….….20

Gas Chromatography and Mass Spectrometry……………………………...….…………22

Supplementary Figures 9-60…………………………………………...….……..22

NMR Data…………………………………..…………………………………….......51

Supplementary Figures 61-130…………………………………….……………...62

Supplementary References…………………………………………………………...…..132

Supplementary List of Gene Sequences

Synthetic gene sequence encoding BaNTR1 from *Bacillus amyloliquefaciens* (UniProt accession number: A0A1Y0XCZ9)[1,2]

GTCCAGACTGCATATGCATCACCATCACCATCATTCCGACAAGAAACACGAGATCATT
GAAGCGTACCAATTTCGCCATGCCACCAAAGAGTTTGATCCGGAGAAGAAAATCTCCG
AATCTGACTTCGAATTCATTCTCGAAACAGGTCGCCTGTCACCGTCGAGTTTGGGTCTG
GAACCATGGCGCTTTGTCGTTGTGCAGAATCCCGAATTACGCGAGAAACTGCGCGAGT
TTACCTGGGGTGCGCAGAAACAGTTACCGACTGCCTCTCACTTCGTACTGCTGCTCGCT
CGTACTGCGAAAGATATTCGGTTTGACGCCGACTATGTGAAGAACCATCTTCGCGAAG
TGAAGAAAATGCCGGATGACATGTATGAAGGCTATATCTCGAAAACCGAAGAGTTTCA
GAAACACGATTTCAATCTGCTGGAAAGCGATCGTGCGTTGTTTGATTGGGCCAGCAAA
CAAACGTACATTGCGCTGGGGAACATGATGACCGCAGCAGCACAGATTGGGATCGATA
GTTGCCCTGTAGAAGGATTCAACTACAAAGAGATTCATCGGATCCTGGAAGAAGAAGG
CCTTCTGGAAGATGGCTCATTCGACATTTCGGTTATGGCTGCTTTTGGCTATCGTGTGC
GCGAACCACGTCCGAAAACGCGTAGCGTTAGCAAAGATGTCGTGAAATGGGTCTAACT
CGAGTGACTGAGCT

Synthetic gene sequence encoding EcNR from *Enterobacter cloacae* (UniProt accession number: Q01234)[2,3]

GTCCAGACTGCATATGCATCACCACCATCACCACGACATCATCTCTGTCGCGTTGAAAC
GCCATAGCACGAAAGCCTTTGATGCGTCGAAGAAACTGACTGCGGAAGAAGCCGAAA
AGATCAAAACGCTGCTGCAGTATAGCCCGTCATCGACCAATTCCCAACCTTGGCACTTC
ATTGTGGCATCAACCGAAGAGGGGAAAGCACGTGTGGCCAAAAGTGCTGCAGGCACA
TACGTGTTCAACGAACGCAAGATGCTTGATGCTAGCCATGTGGTTGTCTTTTGTGCCAA
AACCGCCATGGATGACGCGTGGCTGGAACGTGTAGTGGATCAGGAGGAAGCAGATGG
TCGCTTTAACACGCCAGAAGCGAAAGCCGCTAACCACAAAGGTCGCACTTATTTCGCG
GATATGCATCGTGTGGATCTGAAGGACGATGACCAGTGGATGGCGAAACAAGTCTACC
TCAATGTTGGCAACTTCCTTCTGGGTGTGGGAGCAATGGGTTTAGACGCTGTACCCATT
GAAGGCTTTGATGCCGCGATTCTGGATGAAGAGTTTGGGCTGAAAGAGAAAGGCTTTA
CCAGCCTGGTAGTTGTTCCGGTTGGCCATCATTCCGTGGAGGACTTCAATGCGACACTC
CCGAAATCTCGGTTGCCGTTAAGTACCATTGTCACCGAATGCTAACTCGAGTGACTGAG
CT

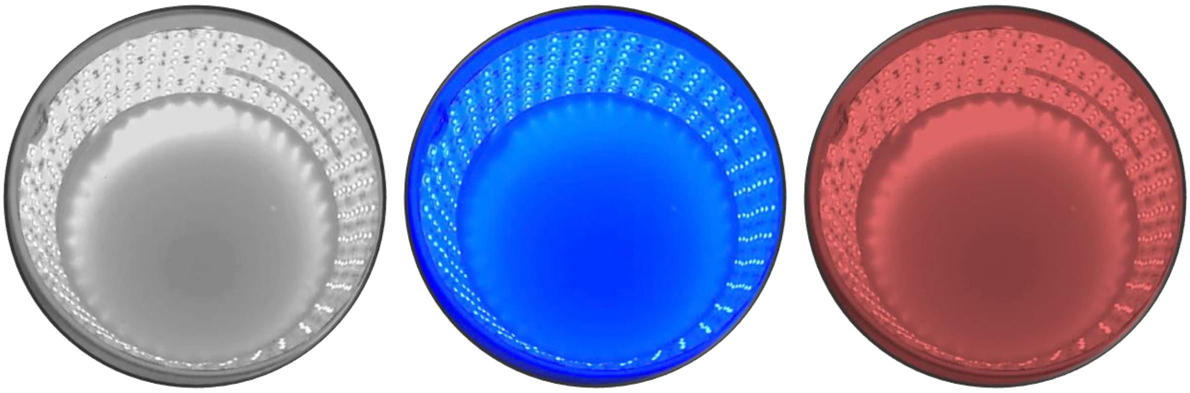

White LEDs Blue LEDs Red LEDs

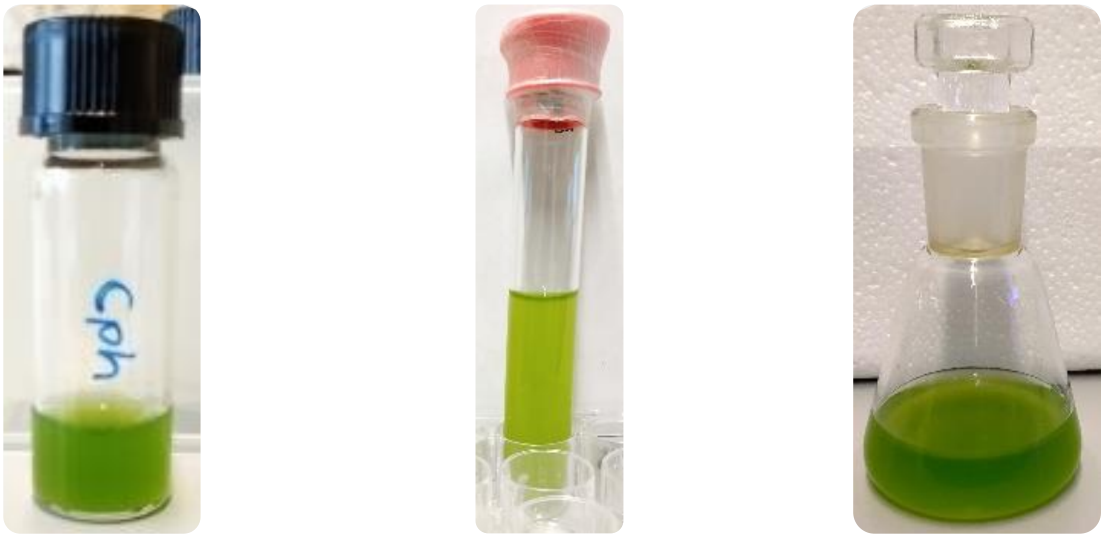

Glass vial, 1mL Glass tube, 10 mL Erlenmeyer flask, 10 mL

Supplementary Figure 1. Top, In-house made photoreactors with white, blue (λ max 440 nm) and red (λ max 660 nm) LEDs (19W, 1000 lx). **Bottom,** Glass vial (for analytical scale; 1 mL), glass tube and Erlenmeyer (for semi-preparative scale; 10 mL) employed to perform the photoenzymatic reactions.

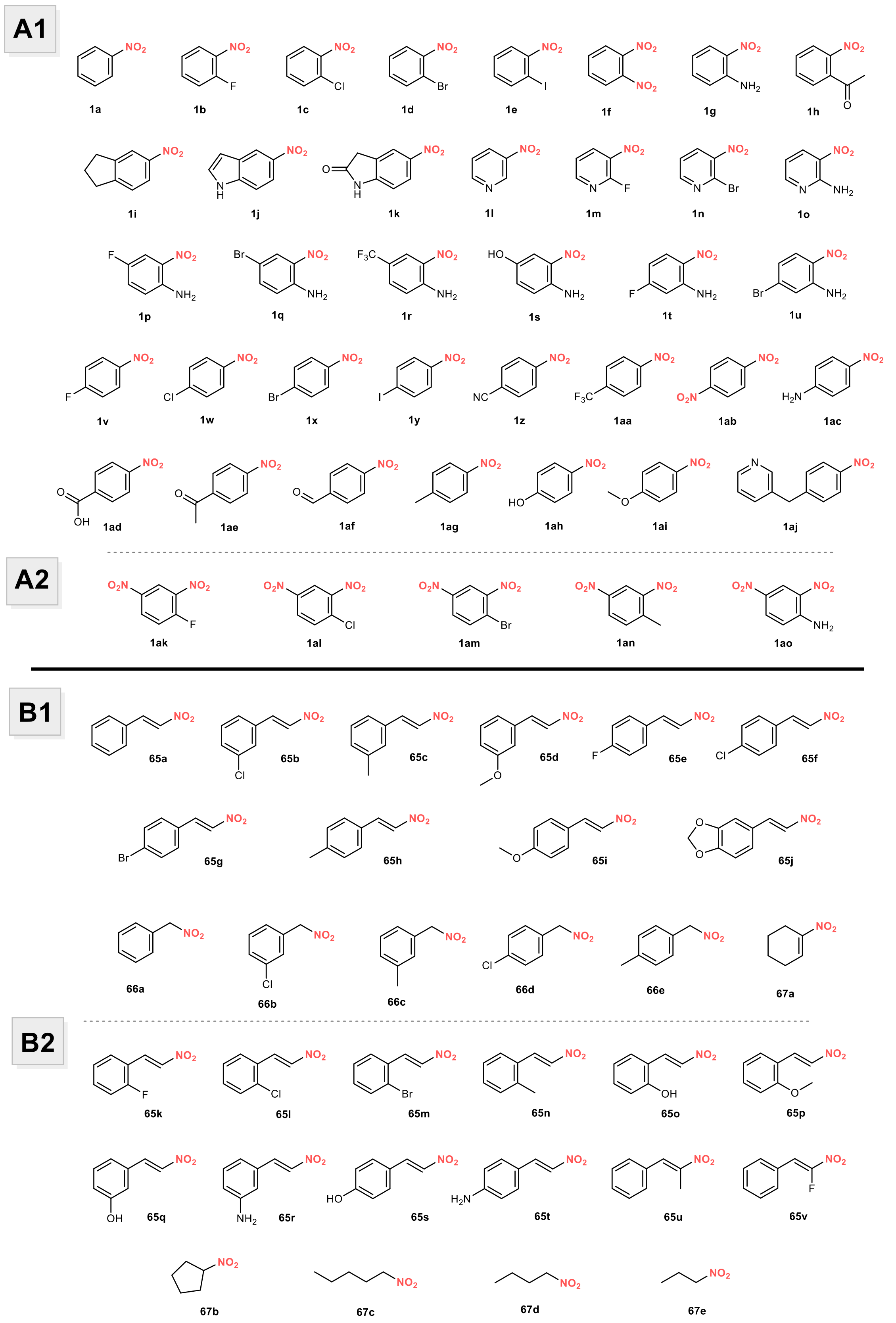

Supplementary Figure 2. Panel of nitro compounds screened for the photoenzymatic reactions. A1) Substrates that resulted in the formation of aromatic amino, azoxy and azo compounds. **A2)** Substrates that led to the formation of a mixture of multiple uncharacterized products. **B1)** Substrates that resulted in the formation of aliphatic amines. **B2)** Compounds that are not accepted as substrates.

Supplementary Results

Photoenzymatic Transformations

Supplementary Table 1. Combination of photocatalyst and LEDs light towards the photoenzymatic reduction of nitrobenzene (**1a**) into aniline (**2**).

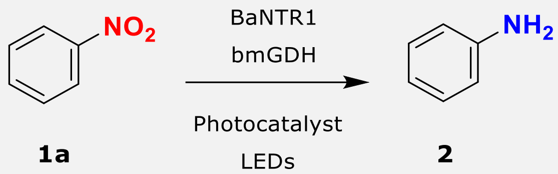

Photocatalyst	LEDs Light	Conversion into **2** (%)[a]
Chlorophyll	White	>99
Rose Bengal	White	46
$[Ru(bpy)_3]Cl_2$	White	77
Chlorophyll	Blue	91
Eosin B	Blue	56
Eosin Y	Blue	68
Free FMN	Blue	60
$[Ru(bpy)_3]Cl_2$	Blue	>99
Chlorophyll	Red	88
Standard enzymatic reaction[b]	No	25

Reaction conditions: anaerobic 0.1 M MOPS buffer pH=7 (1 mL), photocatalyst (0.25 mM), nitrobenzene (5 mM, 10% DMSO), NAD^+ (0.5 mM), glucose (50 mM), bmGDH (2 µM) and BaNTR1 (10 µM). [a] Conversion was determined by GC-MS (reaction time = 18 h) [b] Standard enzymatic nitroreduction reaction (without photocatalyst and LEDs) shown for comparison, resulting in a mixture of products. Reaction conditions: 0.1 M MOPS buffer pH=7 (1 mL), nitrobenzene (5 mM, 10% DMSO), NAD^+ (0.5 mM), glucose (50 mM), bmGDH (2 µM) and BaNTR1 (10 µM), performed on bench under ambient light. BaNTR1, nitroreductase from *Bacillus amyloliquefaciens*; bmGDH, glucose dehydrogenase from *Bacillus megaterium*.

Supplementary Table 2. Control reactions for the photoenzymatic reduction of nitrobenzene (**1a**) to aniline (**2**).

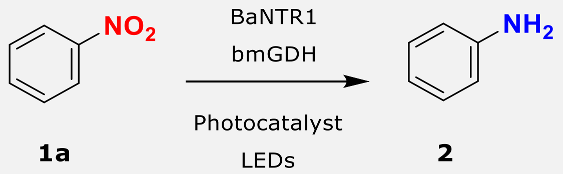

Control reaction condition	Conversion into **2** (%)[a]	
	White LEDs Chlorophyll	Blue LEDs $[Ru(bpy)_3]Cl_2$
Complete reaction	>99	>99
Without BaNTR1	N.C	45
Without bmGDH	N.C	N.C
Without BaNTR1/bmGDH	N.C	N.C
Without NAD^+	N.C	N.C
Without glucose	N.C	N.C
Without photocatalyst	48	58
Without BaNTR1/bmGDH + NADH (20 mM)	N.C	33
Without BaNTR1 + free FMN (5 mM)	11	27
Dark (Without LEDs)	45	44
Without BaNTR1/bmGDH + gum arabic (0.25 mM)	N.C	-----
Without BaNTR1/bmGDH + lactose (0.25 mM)	N.C	-----
Without BaNTR1/bmGDH + gum arabic (0.25 mM) + lactose (0.25 mM)	N.C	-----
In aerobic buffer (instead of N_2)	27	35
Standard aerobic enzymatic reaction[b]	25	

Reaction conditions: 0.1 M anaerobic MOPS buffer pH=7 (1 mL), photocatalyst (0.25 mM), nitrobenzene (5 mM, 10% DMSO), NAD^+ (0.5 mM), glucose (50 mM), bmGDH (2 μM) and BaNTR1 (10 μM). [N.C] No conversion. [a] Conversion was determined by GC-MS (reaction time = 18 h) [b] Standard enzymatic nitroreduction reaction (without photocatalyst and LEDs) shown for comparison, resulting in a mixture of products. Reaction conditions: 0.1 M MOPS buffer pH = 7 (1 mL), nitrobenzene (5 mM, 10% DMSO), NAD^+ (0.5 mM), glucose (50 mM), bmGDH (2 μM) and BaNTR1 (10 μM), performed on bench under ambient light.

Supplementary Table 3. Control reactions for the photoenzymatic reduction of nitrobenzene (**1a**) to azoxybenzene (**37**).

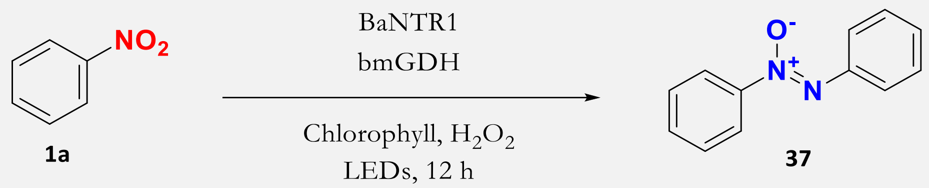

Control reaction condition	Conversion into **37** (%)[a]	
	White LEDs	Red LEDs
Complete reaction	>99	90
Dioxygen saturated buffer (instead of H_2O_2)	91	82
Without BaNTR1	N.C	N.C
Without bmGDH	N.C	N.C
Without BaNTR1/bmGDH	N.C	N.C
Without NAD^+	N.C	N.C
Without glucose	N.C	N.C
Without chlorophyll	42	36
Without BaNTR1/bmGDH + NADH (20 mM)	N.C	N.C
Without BaNTR1 + free FMN (5 mM)	21	10
Dark (Without LEDs)	29	26
Nitrosobenzene (2.5 mM) + N-phenylhydroxylamine (2.5 mM)[c]	68	62
Nitrosobenzene (5 mM)[c]	52	44
N-phenylhydroxylamine (5 mM)[c]	33	28
Aniline (5 mM)[c]	N.C	N.C
Standard aerobic enzymatic reaction[b]	19	

Reaction conditions: 0.1 M MOPS buffer pH=7 (1 mL), chlorophyll (0.25 mM), nitrobenzene (5 mM, 10% DMSO), NAD^+ (0.5 mM), glucose (50 mM), H_2O_2 (15 mM), bmGDH (2 μM) and BaNTR1 (10 μM). [N.C] No conversion. [a] Conversion was determined by GC-MS (reaction time = 12 h). [b] Standard enzymatic nitroreduction reaction shown for comparison, resulting in a mixture of products. Reaction conditions: 0.1 M MOPS buffer pH = 7 (1 mL), nitrobenzene (5 mM, 10% DMSO), NAD^+ (0.5 mM), glucose (50 mM), bmGDH (2 μM) and BaNTR1 (10 μM), performed on bench under ambient light. [c] Compound used as starting material.

Supplementary Table 4. Control reactions for the photoenzymatic reduction of nitrobenzene (**1a**) to azobenzene (**53**).

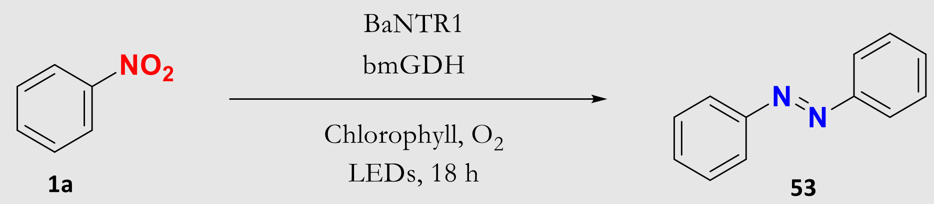

Control reaction condition	Conversion into **53** (%)[a]	
	White LEDs	Red LEDs
Complete reaction in tandem photoreactors	>99	
Complete reaction in single photoreactor	N.C	47
Without BaNTR1	N.C	N.C
Without bmGDH	N.C	N.C
Without BaNTR1/bmGDH	N.C	N.C
Without NAD^+	N.C	N.C
Without glucose	N.C	N.C
Without chlorophyll	N.C	N.C
Without BaNTR1/bmGDH + NADH (20 mM)	N.C	N.C
Without BaNTR1 + free FMN (5 mM)	N.C	N.C
+ H_2O_2 (15 mM)	N.C	N.C
Anerobic buffer (N_2)	N.C	N.C
Dark (Without LED)	N.C	N.C
Nitrosobenzene (2.5 mM) + N-phenylhydroxylamine (2.5 mM)[c]	N.C	49
Nitrosobenzene (5 mM)[c]	N.C	36
N-phenylhydroxylamine (5 mM)[c]	N.C	10
Aniline (5 mM)[c]	N.C	N.C
Azoxybenzene (5mM)[c]	N.C	>99%
Standard aerobic enzymatic reaction[b]	N.C	

Reaction conditions: dioxygen saturated 0.1 M MOPS buffer pH=7 (1 mL), chlorophyll (0.5 mM), nitrobenzene (5 mM, 10% DMSO), NAD^+ (0.5 mM), glucose (50 mM), bmGDH (2 μM) and BaNTR1 (10 μM). [N.C] No conversion. [a] Conversion was determined by GC-MS (reaction time = 18 h). [b] Standard enzymatic nitroreduction reaction shown for comparison, resulting in a mixture of products. Reaction conditions: 0.1 M MOPS buffer pH=7 (1 mL), nitrobenzene (5 mM, 10% DMSO), NAD^+ (0.5 mM), glucose (50 mM), bmGDH (2 μM) and BaNTR1 (10 μM), performed on bench under ambient light. [c] Compound used as substrate.

Supplementary Table 5. Flavoenzyme screening for the enzymatic reduction of **65a** and **66a**.

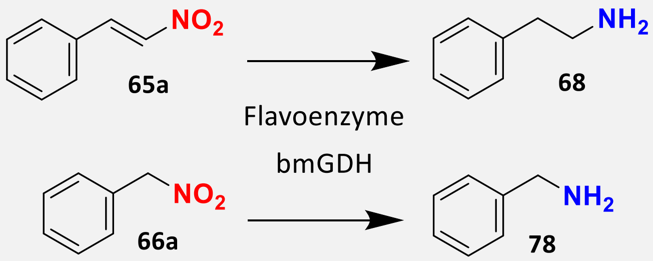

Flavoenzyme	Conversion into **68** (%)[a]	Conversion into **78** (%)[a]
AzoR	N.C	N.C
AzoR1	N.C	N.C
BaNTR1	N.C	N.C
EcNR	2	3
NfsA	N.C	N.C
NfsB	N.C	N.C
NRSal	N.C	N.C
YdgI	N.C	N.C

Reaction conditions: 0.1 M MOPS buffer pH=6.5 (1 mL), substrate (5 mM, 10% DMSO), NAD^+ (0.5 mM), glucose (50 mM), bmGDH (2 µM) and flavoenzyme (20 µM). [a] Conversion determined by GC-MS (reaction time = 10 h). AzoR (*Escherichia coli*, UniProt accession number: P41407); AzoR1 (*Pseudomonas aeruginosa*, UniProt accession number: Q9I5F3); BaNTR1 (*Bacillus amyloliquefaciens*, UniProt accession number: A0A1Y0XCZ9); EcNR (*Enterobacter cloacae*, UniProt accession number: Q01234); NfsA (*Escherichia coli*, UniProt accession number: P17117); NfsB (*Escherichia coli*, UniProt accession number: P38489); NRSal (*Salmonella typhimurium*, UniProt accession number: P15888); YdgI (*Bacillus subtilis*, UniProt accession number: E0U4H5).

Supplementary Table 6. Reaction optimization for the enzymatic reduction of nitro compounds **65a** and **66a** into aliphatic amines **68** and **78**.

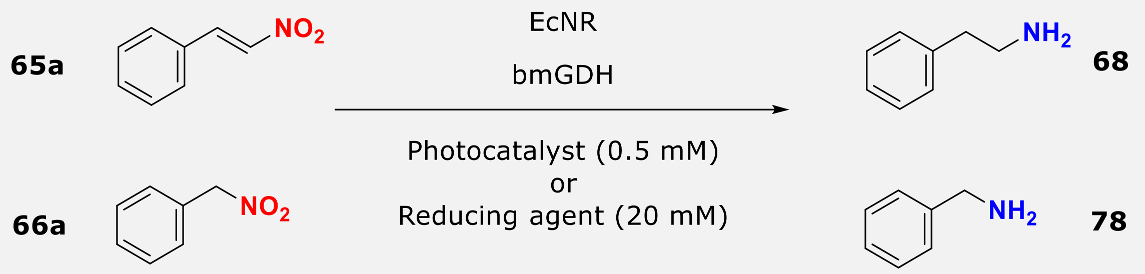

Reaction conditions	Conversion into **68** (%)[a]	Conversion into **78** (%)[a]
NH_4VO_3 (aerobic)[1]	N.C	N.C
NH_4VO_3 (anaerobic)[1]	N.C	N.C
$NaBH_4$ (aerobic)[1]	N.C	N.C
$NaBH_4$ (anaerobic)[1]	N.C	N.C
Ascorbic acid (aerobic)[1]	37	25
Ascorbic acid (anaerobic)[1]	36	27
$Ru(bpy)_3Cl_2$ + blue LEDs (aerobic)[2]	N.C	N.C
$Ru(bpy)_3Cl_2$ + blue LEDs (anaerobic)[2]	N.C	N.C
Chlorophyll + white LEDs (aerobic)[2]	55	63
Chlorophyll + white LEDs (anaerobic)[2]	58	57
Chlorophyll + red LEDs (aerobic)[2]	66	71
Chlorophyll + red LEDs (anaerobic)[2]	64	73
Chlorophyll + ascorbic acid + red LEDs (aerobic)[2]	98	93
Chlorophyll + ascorbic acid + red LEDs (anaerobic)[2]	92	90
Standard aerobic enzymatic reaction[1,b]	2	3

Reaction conditions: 0.1 M MOPS buffer pH=6.5 (1 mL, 10 h), substrate (5 mM, 10% DMSO), NAD^+ (0.5 mM), glucose (50 mM), reducing agent[1] (20 mM) or photocatalyst[2] (0.5 mM), bmGDH (2 µM) and EcNR (20 µM). [a] Conversion determined by GC-MS (reaction time = 10 h). [b] Standard reaction shown for comparison. Reaction conditions: 0.1 M MOPS buffer pH = 6.5 (1 mL, 10 h), substrate (5 mM, 10% DMSO), NAD^+ (0.5 mM), glucose (50 mM), bmGDH (2 µM) and EcNR (20 µM) [1] Reaction performed on bench under ambient light [2] Reaction performed in photoreactor.

Supplementary Table 7. Control reactions towards photobiocatalytic reduction of nitro compounds **65a** and **66a** into aliphatic amines **68** and **78**.

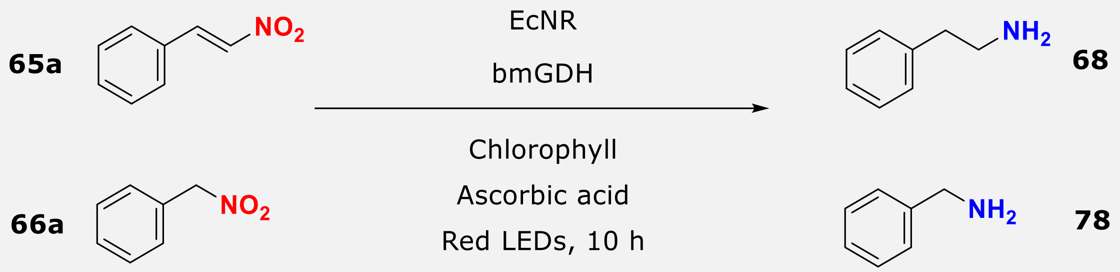

Photoenzymatic control reaction	Conversion into **68** (%)[a]	Conversion into **78** (%)[a]
Complete	98	93
Without EcNR	N.C	N.C
Without bmGDH	N.C	N.C
Without EcNR/bmGDH	N.C	N.C
Without NAD^+	N.C	N.C
Without glucose	N.C	N.C
Without chlorophyll + ascorbic acid	37	25
Without ascorbic acid + chlorophyll	65	73
Without EcNR/bmGDH, without chlorophyll + ascorbic acid (20 mM)	N.C	N.C
Without EcNR/bmGDH + NADH (20 mM)	3[b]	N.C
Without EcNR + free FMN (5 mM)	22[b]	N.C
Dark (Without LEDs)	33	28
Standard aerobic enzymatic reaction[c]	2	3

Reaction conditions: red LEDs (λ max 660 nm), 0.1 M MOPS buffer pH=6.5 (1 mL), chlorophyll (0.5 mM), substrate (5 mM, 10% DMSO), NAD^+ (0.5 mM), glucose (50 mM), ascorbic acid (20 mM), bmGDH (2 µM) and EcNR (20 µM). [a] Conversion determined by GC-MS analysis (reaction time = 10 h). [b] Carbon-carbon double bond reduction. [c] Standard enzymatic reaction shown for comparison. Reaction conditions: 0.1 M MOPS buffer pH = 6.5 (1 mL, 10 h), substrate (5 mM, 10% DMSO), NAD^+ (0.5 mM), glucose (50 mM), bmGDH (2 µM) and EcNR (20 µM), performed on bench under ambient light.

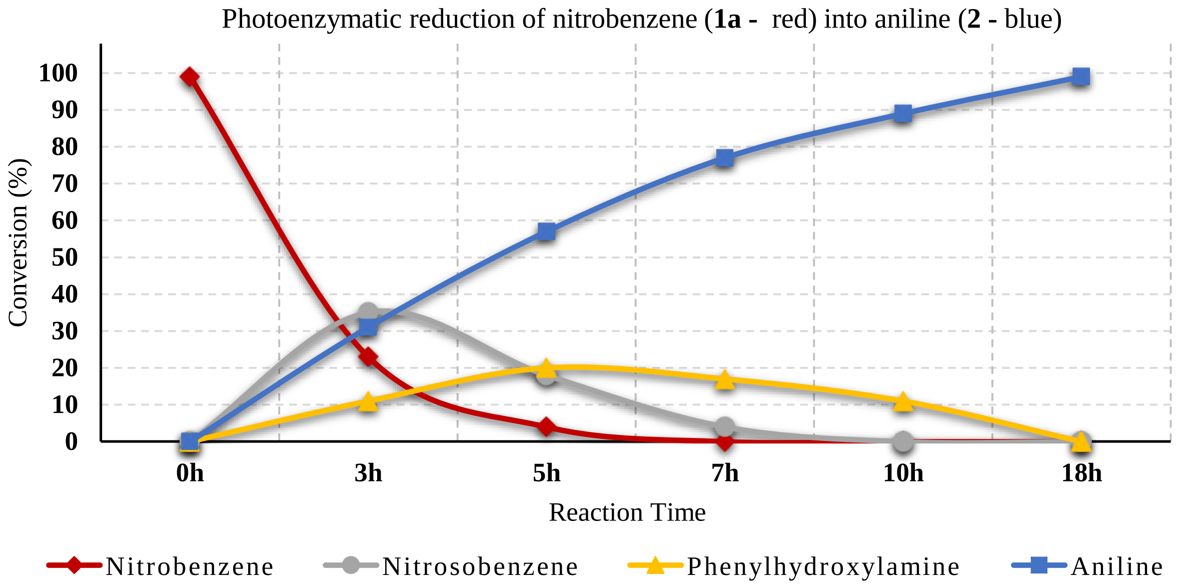

Supplementary Figure 3. Time course measurement of substrate consumption and product formation for the photoenzymatic reduction of nitrobenzene (**1a**) into aniline (**2**). Reaction conditions: white LEDs (1000 lx), 0.1 M anaerobic MOPS buffer pH=7 (1 mL), chlorophyll (0.25 mM), nitrobenzene (5 mM, 10% DMSO), NAD^+ (0.5 mM), glucose (50 mM), bmGDH (2 µM) and BaNTR1 (10 µM). Conversion determined via GC-MS analysis.

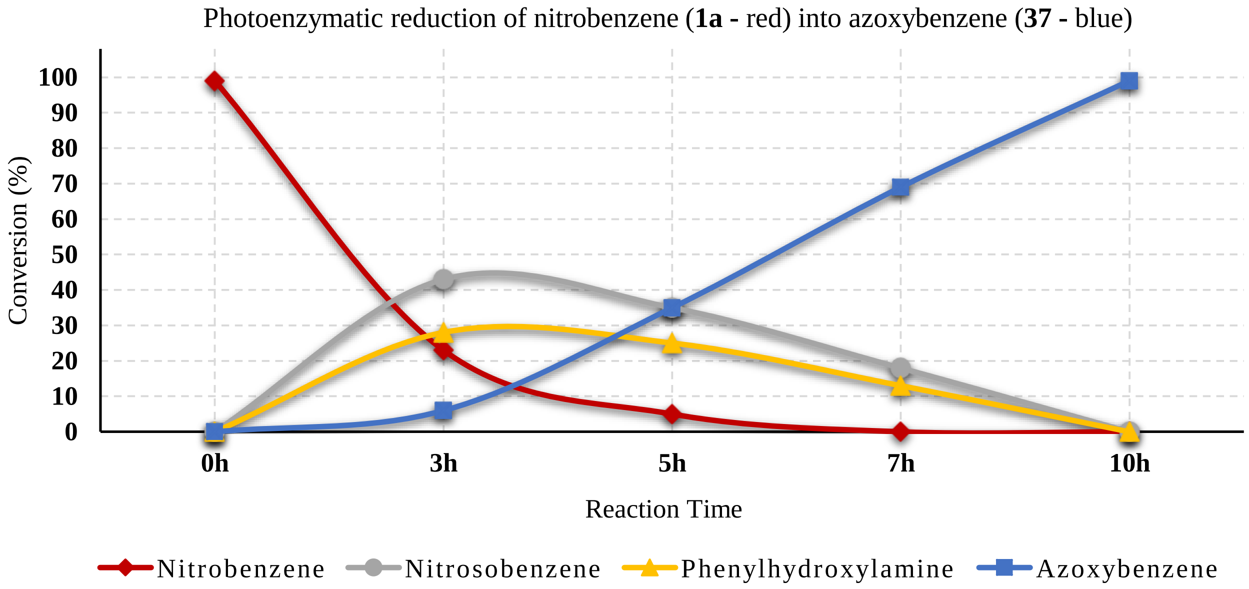

Supplementary Figure 4. Time course measurement of substrate consumption and product formation for the photoenzymatic reduction of nitrobenzene (**1a**) into azoxybenzene (**37**). Reaction conditions: 0.1 M MOPS buffer pH=7 (1 mL), chlorophyll (0.25 mM), nitrobenzene (5 mM, 10% DMSO), NAD^+ (0.5 mM), glucose (50 mM), H_2O_2 (15 mM), bmGDH (2 µM) and BaNTR1 (10 µM). Conversion determined via GC-MS analysis.

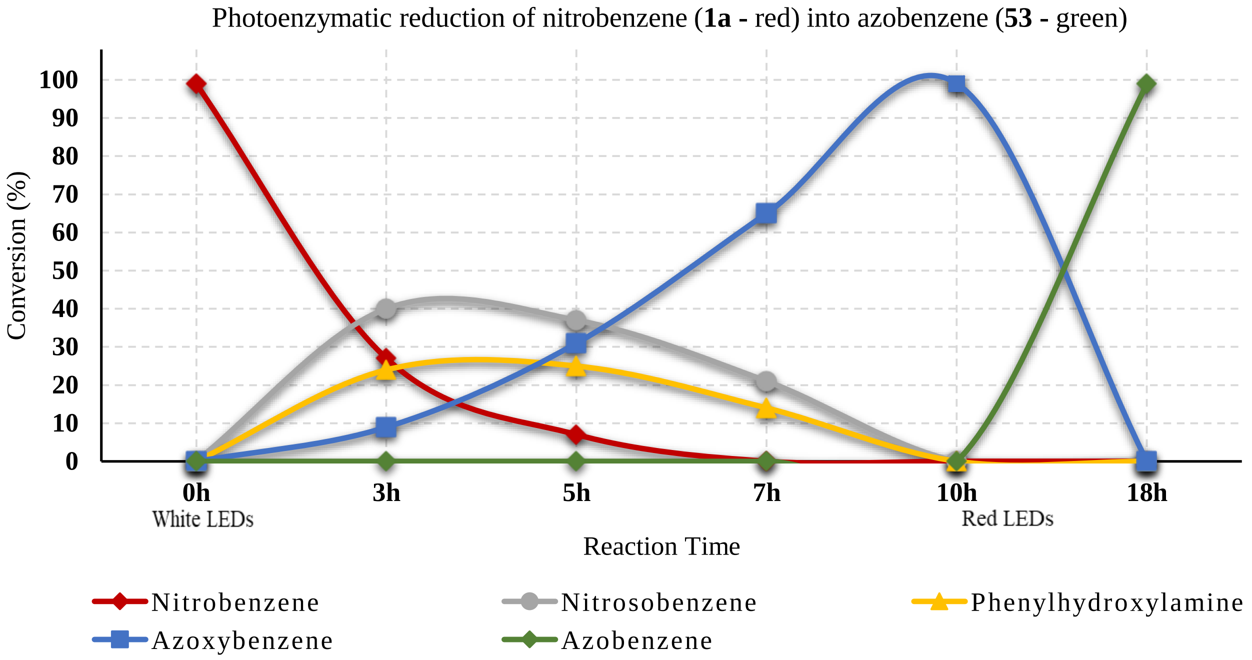

Supplementary Figure 5. Time course measurement of substrate consumption and product formation for the photoenzymatic reduction of nitrobenzene (**1a**) into azobenzene (**53**). Reaction conditions: sequential white and red LEDs (1000 lx), dioxygen saturated 0.1 M MOPS buffer pH=7 (1 mL), chlorophyll (0.5 mM), nitrobenzene (5 mM, 10% DMSO), NAD^+ (0.5 mM), glucose (50 mM), bmGDH (2 µM) and BaNTR1 (10 µM). Conversion determined via GC-MS analysis.

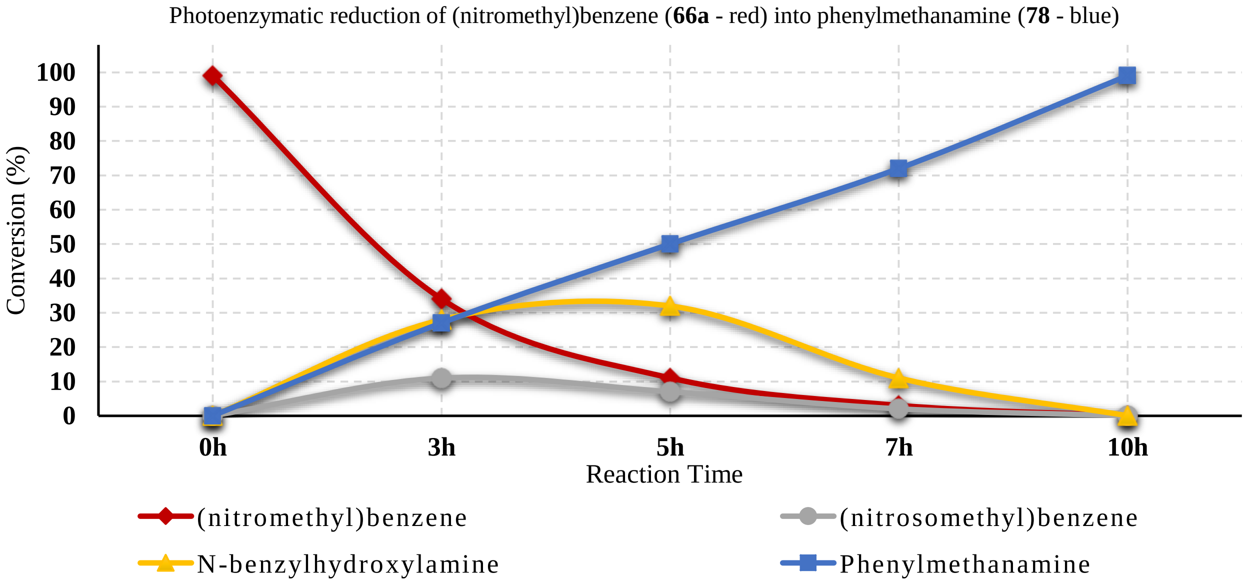

Supplementary Figure 6. Time course measurement of substrate consumption and product formation for the photoenzymatic reduction of (nitromethyl)benzene (**66a**) into phenylmethanamine (**78**). Reaction conditions: red LEDs (1000 lx), 0.1 M MOPS buffer pH=6.5 (1 mL), chlorophyll (0.5 mM), substrate (5 mM, 10% DMSO), NAD^+ (0.5 mM), glucose (50 mM), ascorbic acid (20 mM), bmGDH (2 µM) and EcNR (20 µM). Conversion determined via GC-MS analysis.

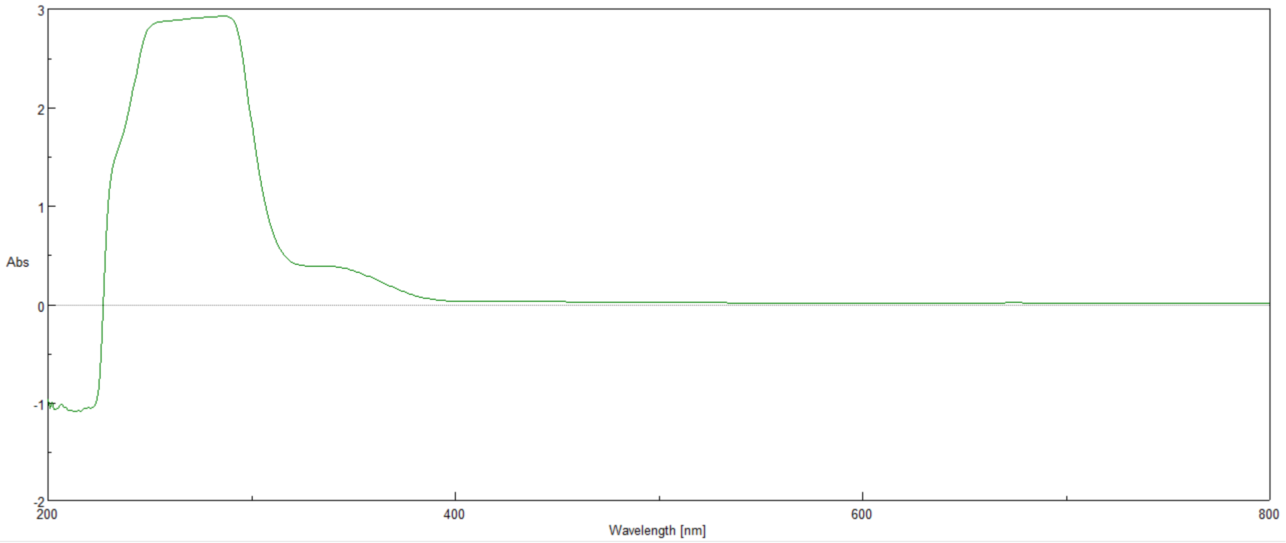

Supplementary Figure 7. UV-Vis spectrum of a representative reaction mixture. The reaction mixture was composed of 0.1 M anaerobic MOPS buffer pH=7 (1 mL), chlorophyll (0.25 mM), 4-nitrobenzonitrile (5 mM, 10% DMSO), NAD^+ (0.5 mM), glucose (50 mM), bmGDH (2 µM) and BaNTR1 (10 µM).

Supplementary Table 8. Control reactions for reduction of nitrobenzene (**I**) into aniline (**IV**).

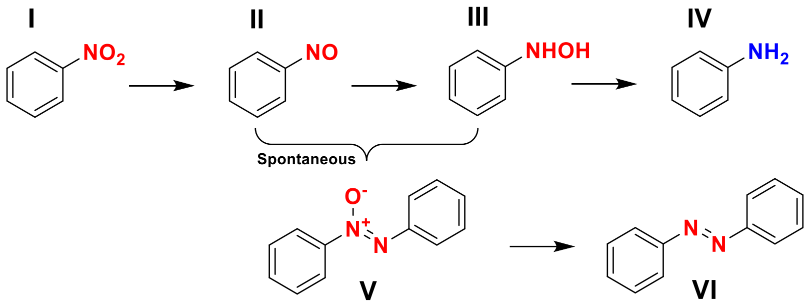

Starting material (I)	Product formation (%)[a]					
	I	II	III	IV	V	VI
Enzymatic[b]	-	37	8	48	7	-
Photocatalytic[c]	>99	-	-	-	-	-
Photobiocatalytic[d]	-	-	-	>99	-	-

Starting material (II)	Product formation (%)[a]					
	I	II	III	IV	V	VI
Enzymatic[b]	-	43	6	40	11	-
Photocatalytic[c]	-	>99	-	-	-	-
Photobiocatalytic[d]	-	-	-	>99	-	-

Starting material (III)	Product formation (%)[a]					
	I	II	III	IV	V	VI
Enzymatic[b]	-	-	-	>99	-	-
Photocatalytic[c]	-	-	>99	-	-	-
Photobiocatalytic[d]	-	-	-	>99	-	-

Reaction conditions: white LEDs (1000 lx), 0.1 M anaerobic MOPS buffer pH=7 (1 mL), chlorophyll a (0.25 mM), starting material (5 mM, 10% DMSO), NAD^+ (0.5 mM), glucose (50 mM), bmGDH (2 μM) and BaNTR1 (10 μM). [a] Product formation was determined by GC-MS (reaction time = 18 h). [b] Enzymatic reaction performed with above conditions excluding chlorophyll. [c] Photocatalytic reaction performed with above conditions excluding BaNTR1. [d] Photobiocatalytic reaction performed with above conditions.

Supplementary Table 9. Control reactions for reduction of nitrobenzene (**I**) into azoxybenzene (**V**).

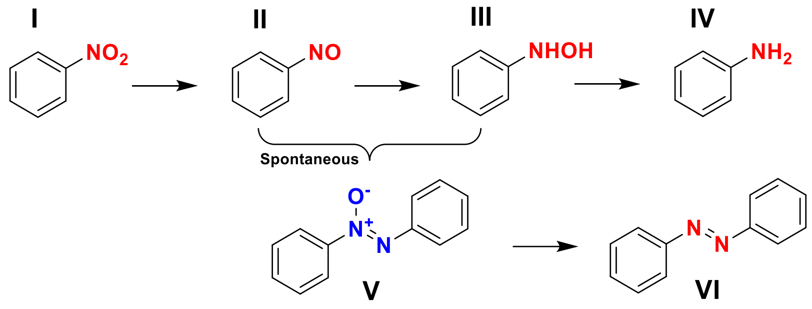

Starting material (I)	Product formation (%)[a]					
	**I**	**II**	**III**	**IV**	**V**	**VI**
Enzymatic[b]	-	55	3	-	42	-
Photocatalytic[c]	>99	-	-	-	-	-
Photobiocatalytic[d]	-	-	-	-	>99	-

Starting material (II)	Product formation (%)[a]					
	**I**	**II**	**III**	**IV**	**V**	**VI**
Enzymatic[b]	-	54	2	-	44	-
Photocatalytic[c]	-	>99	-	-	-	-
Photobiocatalytic[d]	-	-	-	48	52	-

Starting material (III)	Product formation (%)[a]					
	**I**	**II**	**III**	**IV**	**V**	**VI**
Enzymatic[b]	-	9	20	54	17	-
Photocatalytic[c]	-	-	>99	-	-	-
Photobiocatalytic[d]	-	-	-	77	23	-

Starting material (IV)	Product formation (%)[a]					
	**I**	**II**	**III**	**IV**	**V**	**VI**
Enzymatic[b]	-	-	-	>99	-	-
Photocatalytic[c]	-	-	-	>99	-	-
Photobiocatalytic[d]	-	-	-	>99	-	-

Reaction conditions: white LEDs (1000 lx), 0.1 M MOPS buffer pH=7 (1 mL), chlorophyll (0.25 mM), starting material (5 mM, 10% DMSO), NAD^+ (0.5 mM), glucose (50 mM), H_2O_2 (15 mM), bmGDH (2 µM) and BaNTR1 (10 µM). [a] Product formation was determined by GC-MS analysis (reaction time = 12 h). [b] Enzymatic reaction performed with above conditions excluding chlorophyll. [c] Photocatalytic reaction performed with above conditions excluding BaNTR1. [d] Photobiocatalytic reaction performed with above conditions.

Supplementary Table 10. Control reactions for reduction of nitrobenzene (**I**) into azobenzene (**VI**).

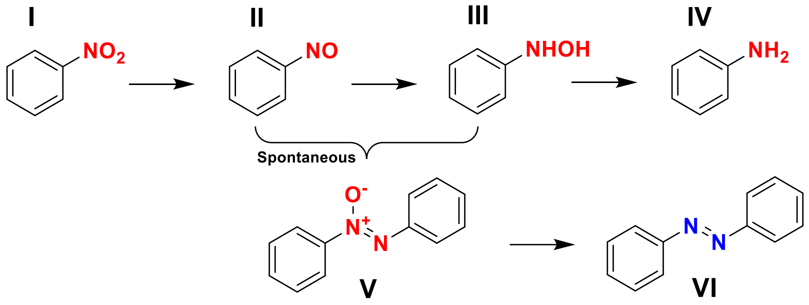

Starting material (I)	Product formation (%)[a]					
	I	II	III	IV	V	VI
Enzymatic[b]	-	41	18	24	17	-
Photocatalytic[c]	>99	-	-	-	-	-
Photobiocatalytic[d]	-	-	-	-	-	>99

Starting material (II)	Product formation (%)[a]					
	I	II	III	IV	V	VI
Enzymatic[b]	-	43	11	28	18	-
Photocatalytic[c]	-	>99	-	-	-	-
Photobiocatalytic[d]	-	-	-	-	-	>99

Starting material (III)	Product formation (%)[a]					
	I	II	III	IV	V	VI
Enzymatic[b]	-	-	-	68	32	-
Photocatalytic[c]	-	-	>99	-	-	-
Photobiocatalytic[d]	-	-	-	74	8	18

Starting material (IV)	Product formation (%)[a]					
	I	II	III	IV	V	VI
Enzymatic[b]	-	-	-	>99	-	-
Photocatalytic[c]	-	-	-	>99	-	-
Photobiocatalytic[d]	-	-	-	>99	-	-

Starting material (V)	Product formation (%)[a]					
	I	II	III	IV	V	VI
Enzymatic[b]	-	-	-	-	>99	-
Photocatalytic[c]	-	-	-	-	>99	-
Photobiocatalytic[d]	-	-	-	-	-	>99

Reaction conditions: white LEDs (1000 lx) then red LEDs (1000 lx, λ max 660 nm), dioxygen saturated 0.1 M MOPS buffer pH=7 (1 mL), chlorophyll (0.5 mM), nitrobenzene (5 mM, 10% DMSO), NAD^+ (0.5 mM), glucose (50 mM), bmGDH (2 μM) and BaNTR1 (10 μM). [a] Product formation was determined by GC-MS (reaction time = 18 h). [b] Enzymatic reaction performed with above conditions excluding chlorophyll. [c] Photocatalytic reaction performed with above conditions excluding BaNTR1. [d] Photobiocatalytic reaction performed with above conditions.

Supplementary Table 11. Control reactions for reduction of (nitromethyl)benzene (**I**) into benzylamine (**IV**).

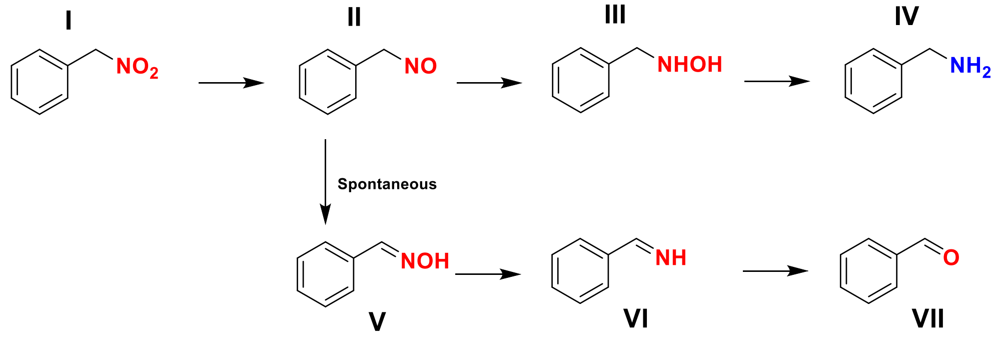

Starting material (I)	Product formation (%)[a]						
	I	II	III	IV	V	VI	VII
Enzymatic[b]	-	32	3	3	52	-	10
Photocatalytic[c]	>99	-	-	-	-	-	-
Ascorbic Acid[d]	>99	-	-	-	-	-	-
Photobiocatalytic[e]	-	-	-	93	4	-	3

Starting material (II)	Product formation (%)[a]						
	I	II	III	IV	V	VI	VII
Enzymatic[b]	-	18	8	7	55	-	12
Photocatalytic[c]	-	>99	-	-	-	-	-
Ascorbic Acid[d]	-	36	17	-	47	-	-
Photobiocatalytic[e]	-	-	-	94	4	-	2

Starting material (III)	Product formation (%)[a]						
	I	II	III	IV	V	VI	VII
Enzymatic[b]	-	-	-	>99	-	-	-
Photocatalytic[c]	-	-	>99	-	-	-	-
Ascorbic Acid[d]	-	-	>99	-	-	-	-
Photobiocatalytic[e]	-	-	-	>99	-	-	-

Reaction conditions: red LEDs (1000 lx, λ max 660 nm), 0.1 M MOPS buffer pH = 6.5 (1 mL), chlorophyll (0.5 mM), substrate (5 mM, 10% DMSO), NAD^+ (0.5 mM), glucose (50 mM), ascorbic acid (20 mM), bmGDH (2 μM) and EcNR (20 μM). [a] Product formation determined by GC-MS analysis (reaction time = 10 h). [b] Reaction performed with above conditions excluding chlorophyll and ascorbic acid. [c] Reaction performed with above conditions excluding BaNTR1 and ascorbic acid. [d] Reaction performed with above conditions excluding BaNTR1 and chlorophyll. [e] Reaction performed with above conditions.

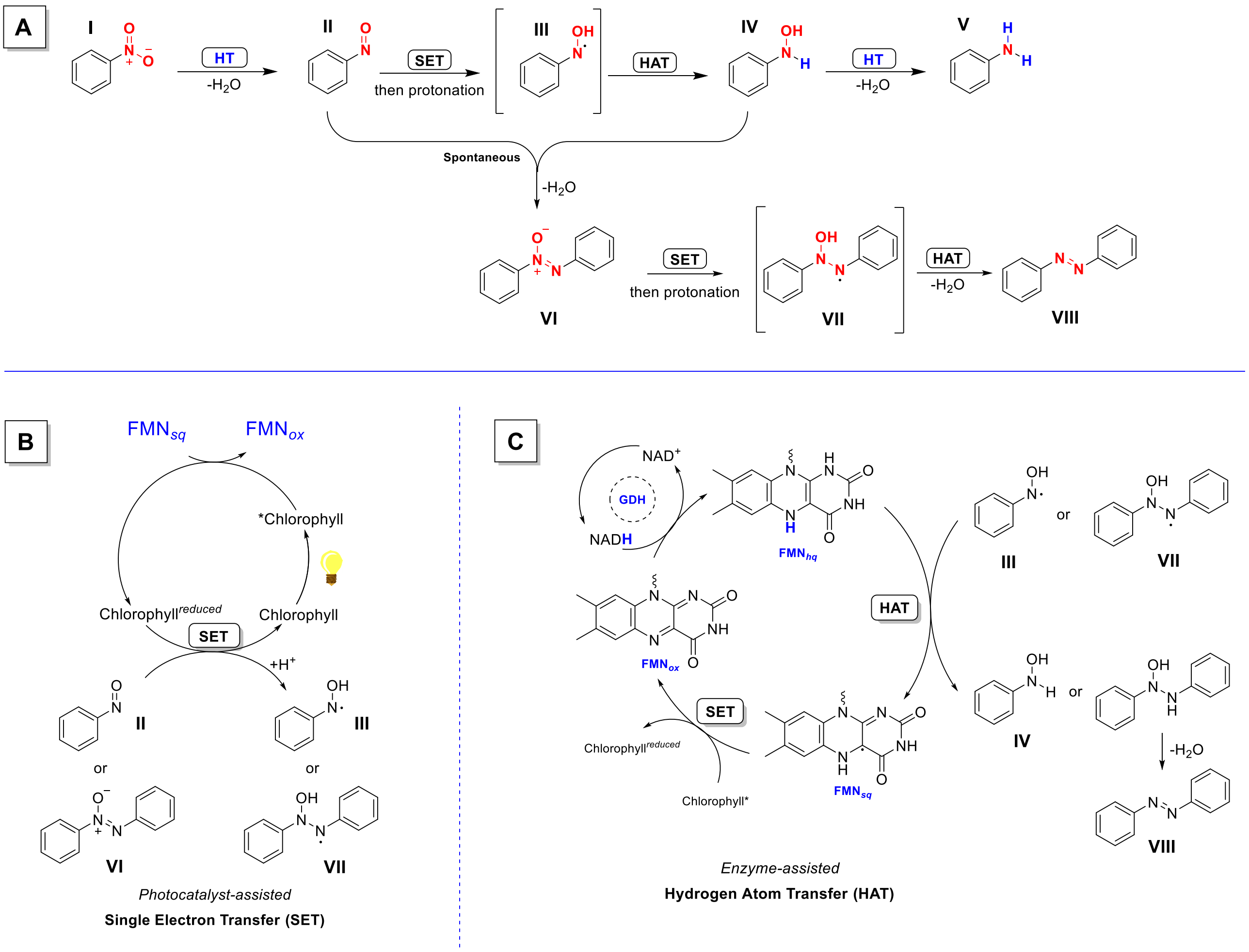

Supplementary Figure 8. Proposed mechanism for the photobiocatalytic reduction of nitro compounds into the corresponding products. [A] Photobiocatalytic pathway for the reduction of nitrobenzene into the corresponding products. [B] Photocatalyst-assisted Single Electron Transfer (SET). [C] Enzyme-assisted Hydrogen Atom Transfer (HAT). A description of this proposed mechanism is given below.

Based on literature data regarding the hydride transfer (HT) mechanism for the enzymatic reduction of nitro compounds,[4–6] flavoenzyme-performed photobiocatalytic reductions,[2,7–10] and supported by the control experiments monitoring the individual contribution of the enzymatic and photochemical catalyst to the overall nitro-reduction pathway (Suppl. Tables 8-11), we propose the following mechanism for the photobiocatalytic reduction of nitrobenzene (**I**) into the corresponding products (Suppl. Figure 8A). This postulated mechanism seems also applicable for the photobiocatalytic reduction of aliphatic nitro compounds. In the first step, the nitrobenzene substrate is properly positioned and activated within the enzymatic active site,[2,4–6,8,11,12] making the nitrogen atom ready for a Hydride Transfer (HT) from the reduced flavin (FMN_{hq}), accompanied by proton acquisition, most likely from solvent. Subsequent dehydration yields the nitrosobenzene intermediate **II**. Given that the nitroso and azoxy intermediates are poorly processed by the individual enzyme system, but are readily reduced by the full photobiocatalytic system under the applied reaction conditions (Suppl. Tables 8-11), we propose that the photocatalytic assistance is executed at these steps. Note that the nitroso group bears a structural resemblance to a keto group, and photoenzymatic ketone reductions by BaNTR1 have previously been reported.[2] Briefly, nitroso intermediate **II** likely undergoes a Single Electron Transfer (SET, Suppl. Figure 8B) performed by the photoexcited chlorophyll, accompanied by a protonation in the enzyme active site, followed by Hydrogen Atom Transfer (HAT, Suppl. Figure 8C) mediated by the enzyme-bound FMN_{hq}, resulting in the formation of N-phenylhydroxylamine intermediate **IV**. The resulting FMN_{sq} and chlorophyll likely quench each other via SET, generating the oxidized flavin (FMN_{ox}). At this stage, the reduction of FMN_{ox} to FMN_{hq} is mediated by NADH, generated via the GDH-based nicotinamide-cofactor recycling system (Suppl. Figure 8C). Like nitrobenzene **I**, the hydroxylamine intermediate **IV** most likely also undergoes enzyme-mediated HT (accompanied by proton acquisition) to yield amine **V** (Suppl. Figure 8A). A similar mechanism can be proposed for the photobiocatalytic radical reduction of azoxybenzene **VI** to provide azobenzene **VIII**.

Gas Chromatography and Mass Spectrometry

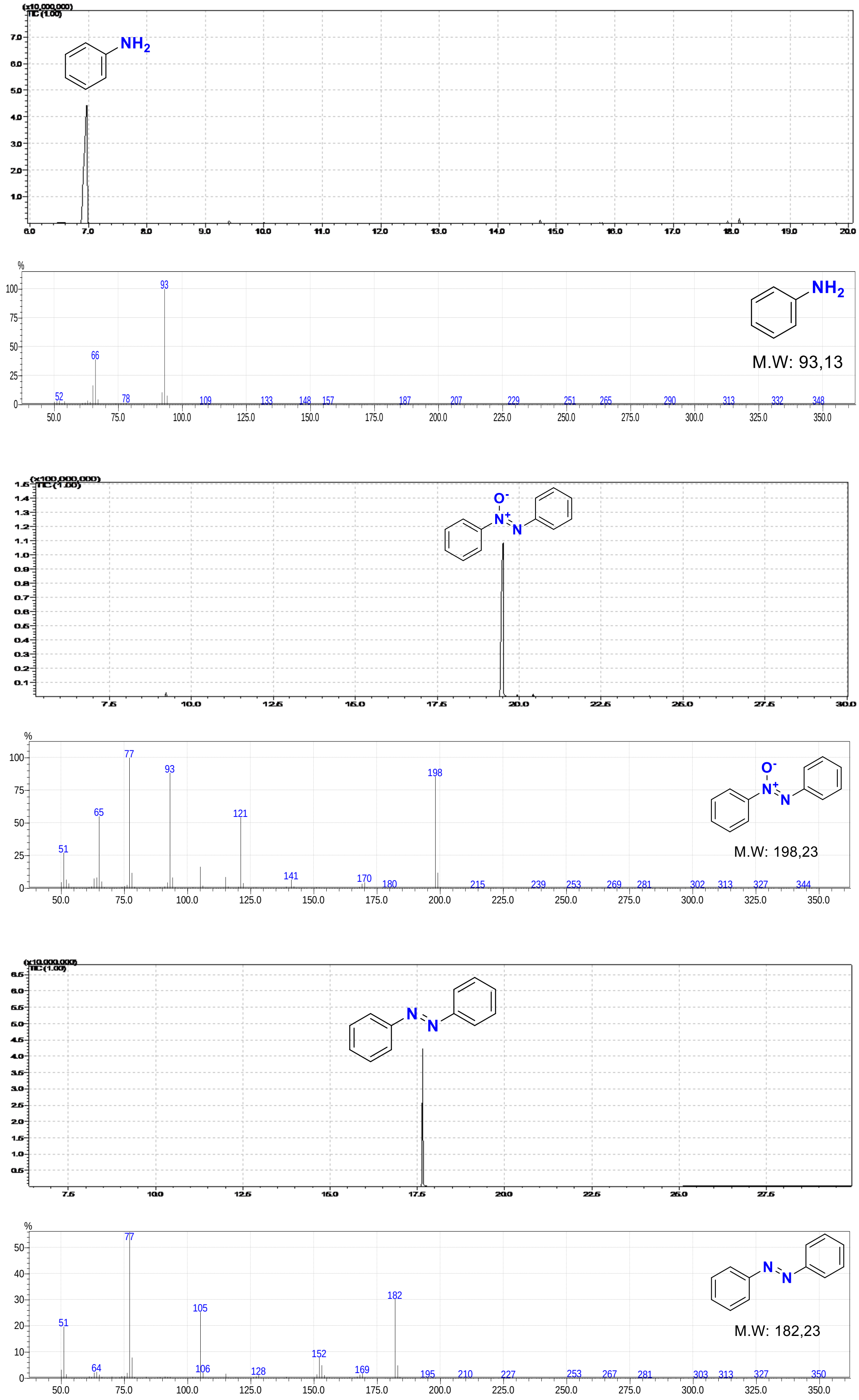

Supplementary Figure 9. Gas chromatography and mass spectra of the products from the photoenzymatic reductions of nitrobenzene. M.W., molecular weight.

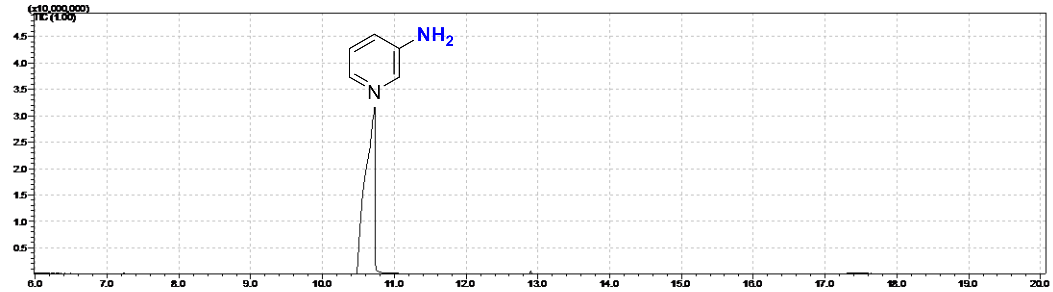

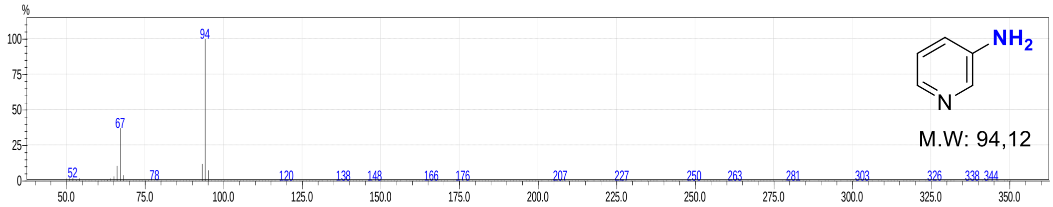

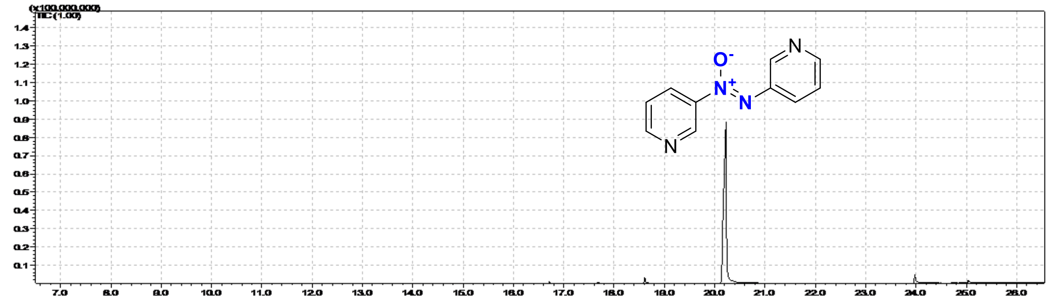

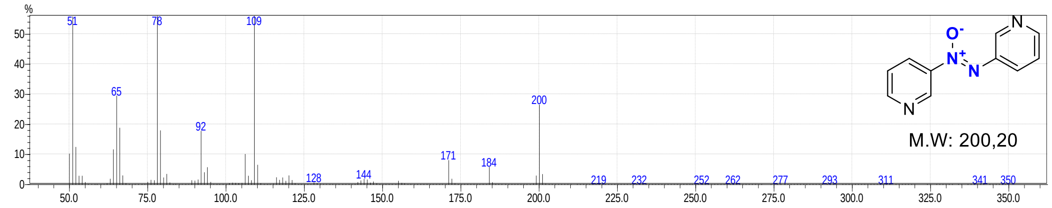

Supplementary Figure 10. Gas chromatography and mass spectra of the products from the photoenzymatic reductions of 3-nitropyridine.

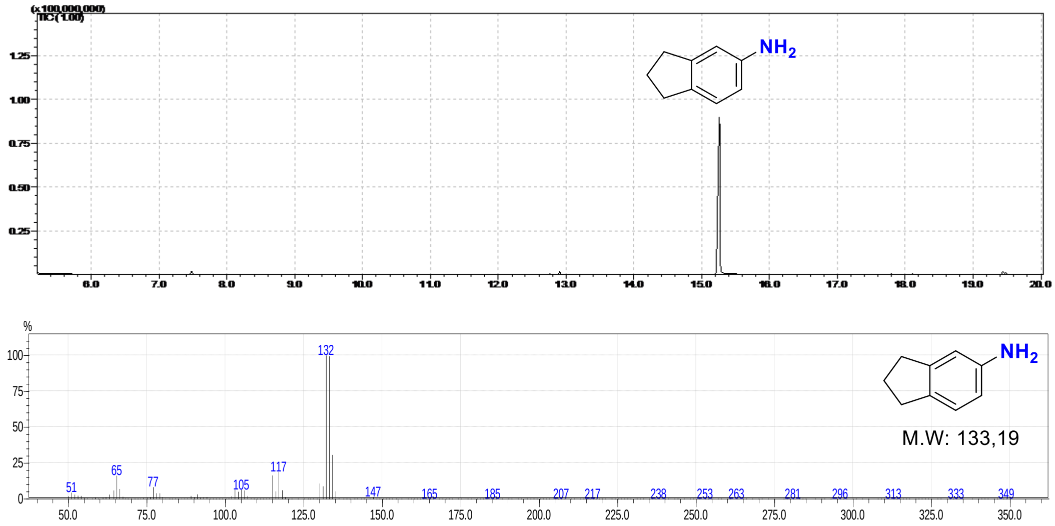

Supplementary Figure 11. Gas chromatography and mass spectra of the product from the photoenzymatic reduction of 5-nitro-2,3-dihydro-1H-indene.

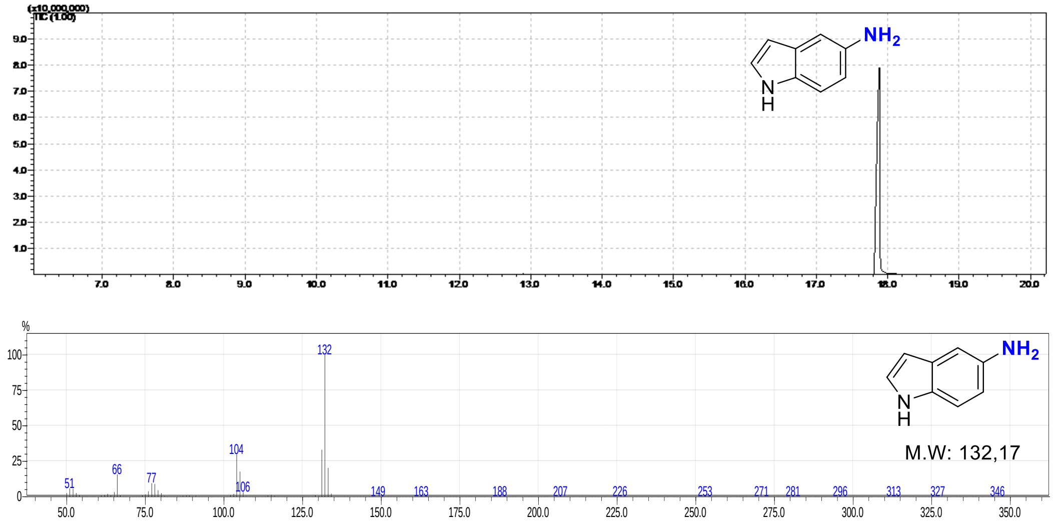

Supplementary Figure 12. Gas chromatography and mass spectra of the product from the photoenzymatic reduction of 5-nitro-1H-indole.

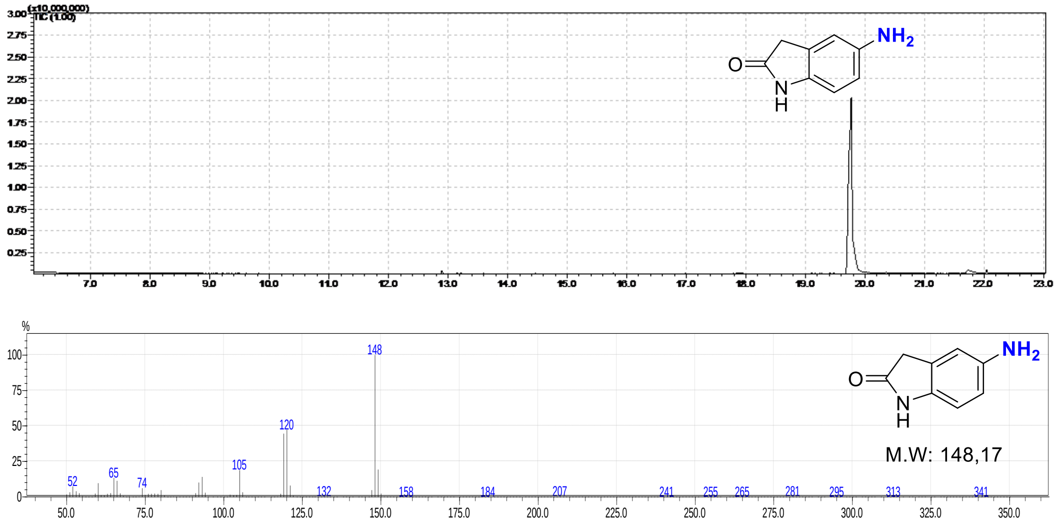

Supplementary Figure 13. Gas chromatography and mass spectra of the product from the photoenzymatic reduction of 5-nitroindolin-2-one.

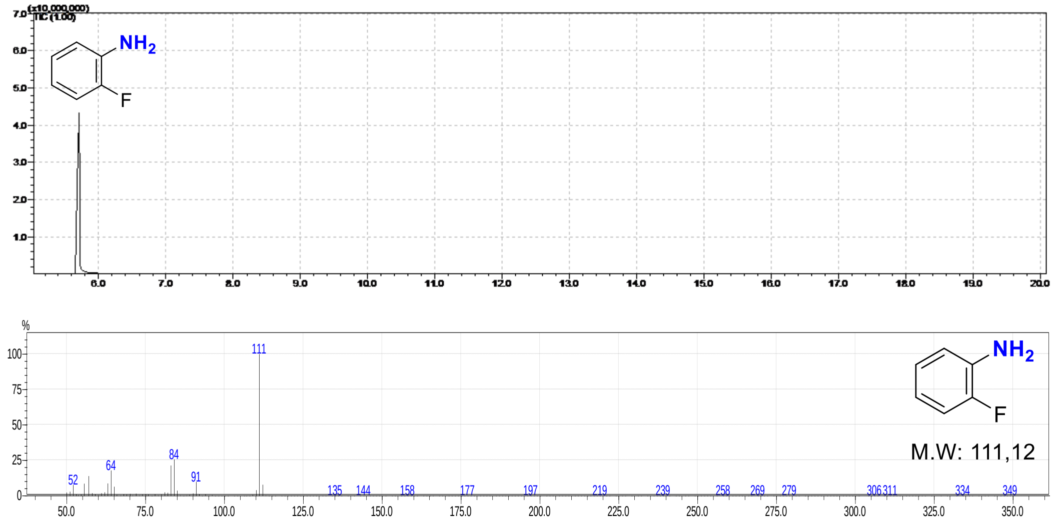

Supplementary Figure 14. Gas chromatography and mass spectra of the products from the photoenzymatic reductions of 1-fluoro-2-nitrobenzene.

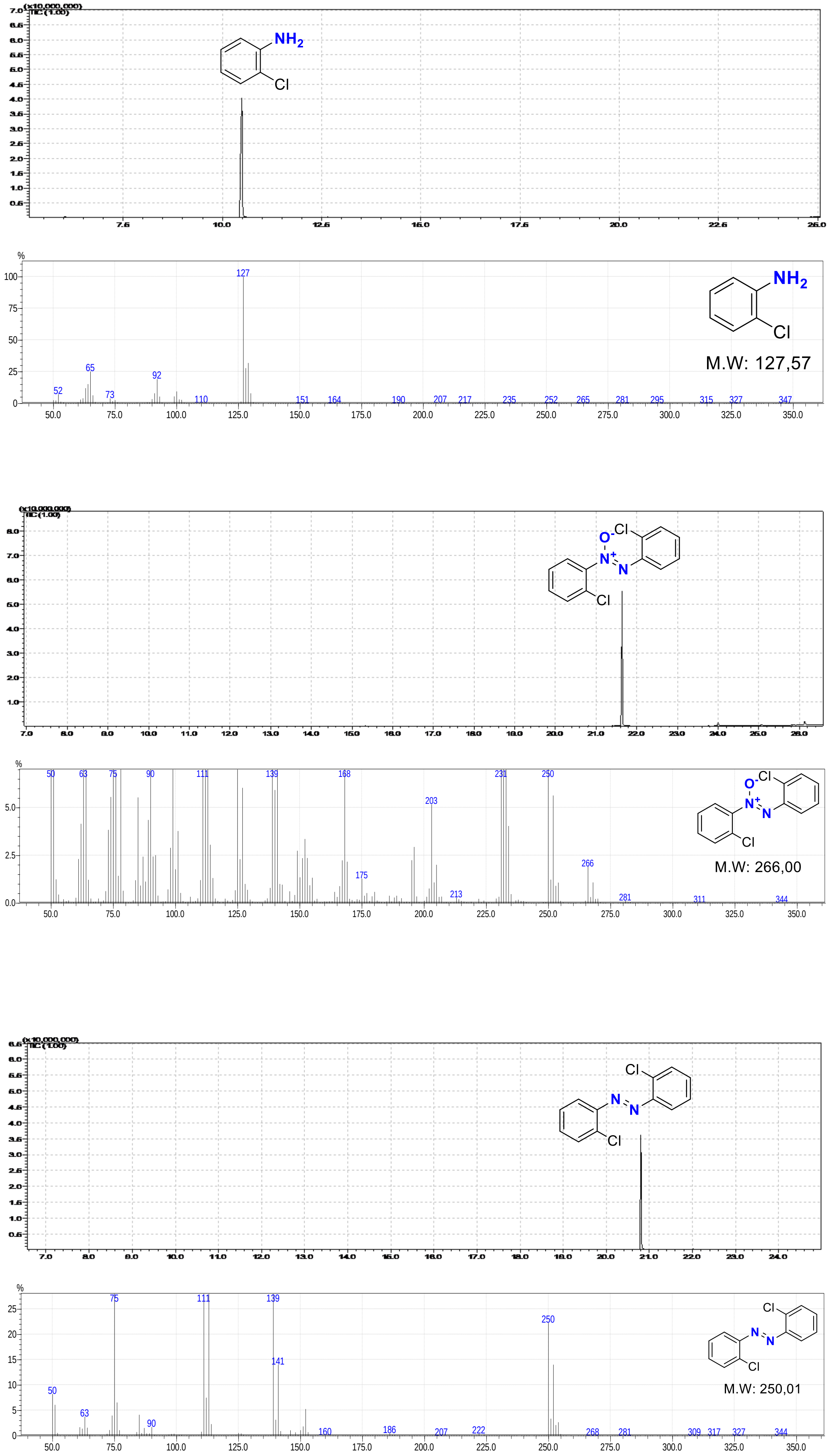

Supplementary Figure 15. Gas chromatography and mass spectra of the products from the photoenzymatic reductions of 1-chloro-2-nitrobenzene.

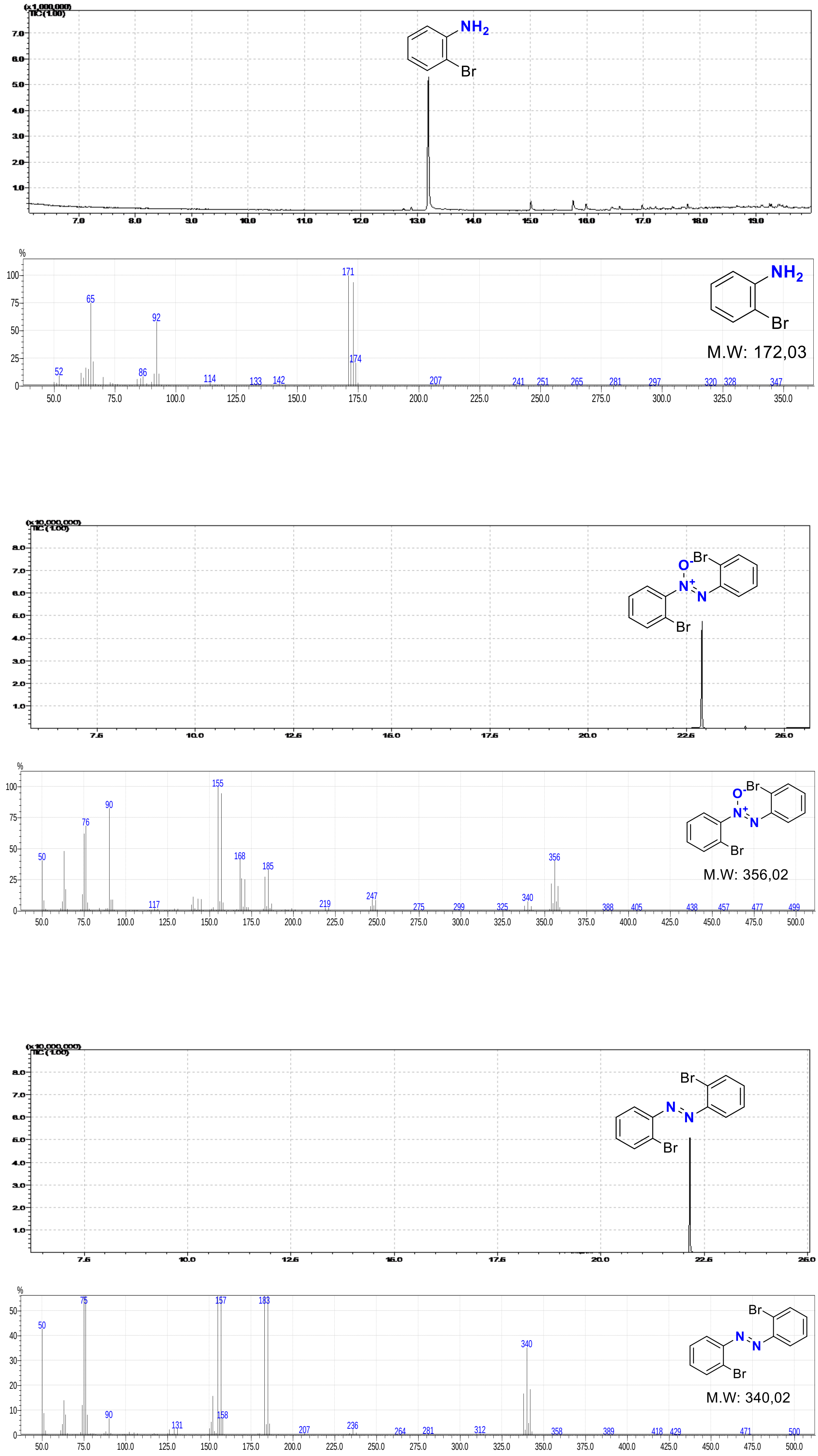

Supplementary Figure 16. Gas chromatography and mass spectra of the products from the photoenzymatic reductions of 1-bromo-2-nitrobenzene.

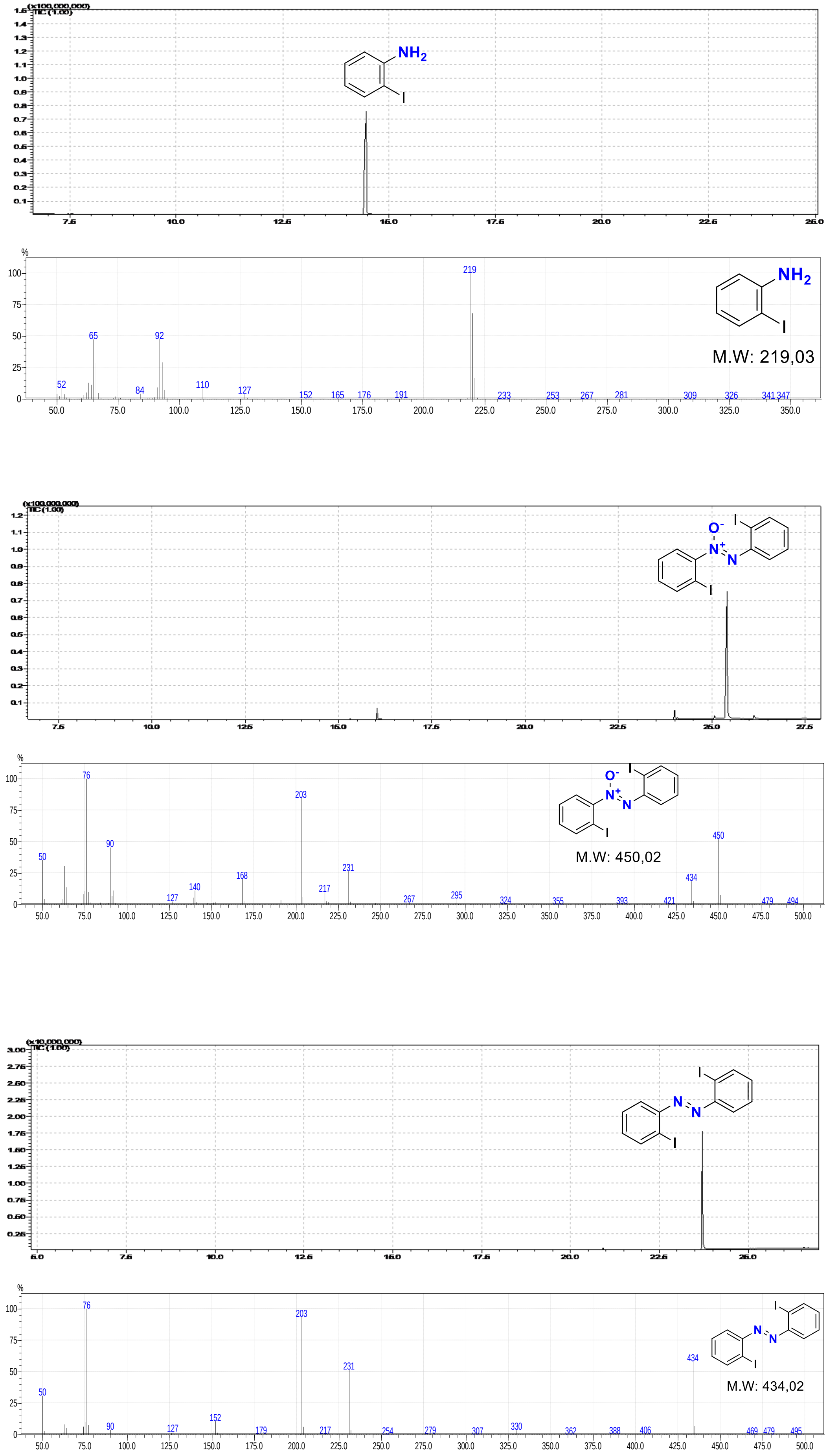

Supplementary Figure 17. Gas chromatography and mass spectra of the products from the photoenzymatic reductions of 1-iodo-2-nitrobenzene.

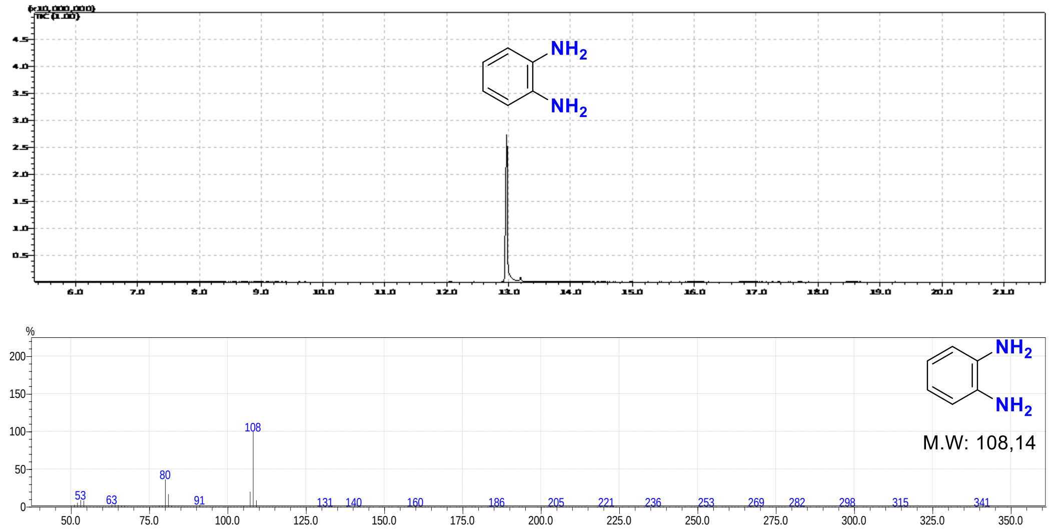

Supplementary Figure 18. Gas chromatography and mass spectra of the product from the photoenzymatic reduction of 1,2-dinitrobenzene.

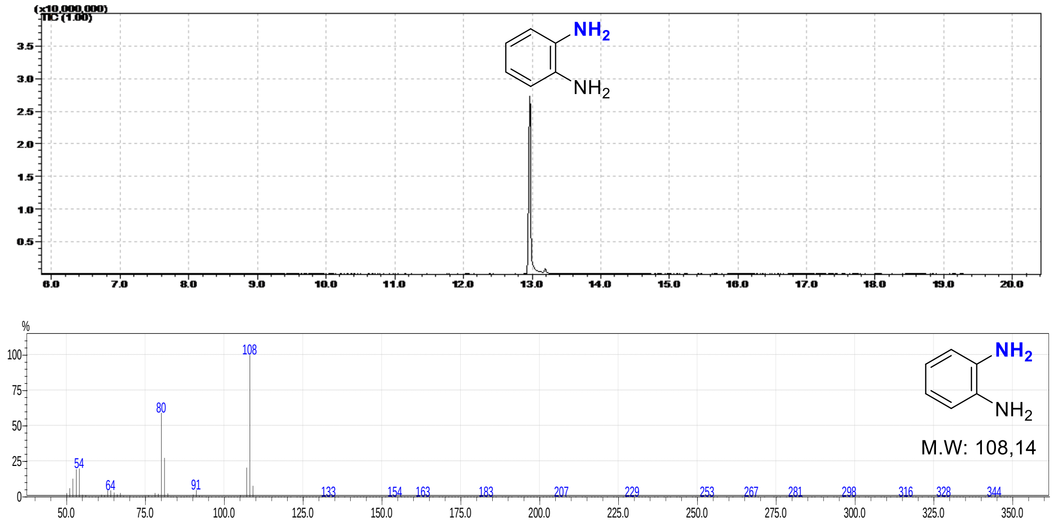

Supplementary Figure 19. Gas chromatography and mass spectra of the product from the photoenzymatic reduction of 2-nitroaniline.

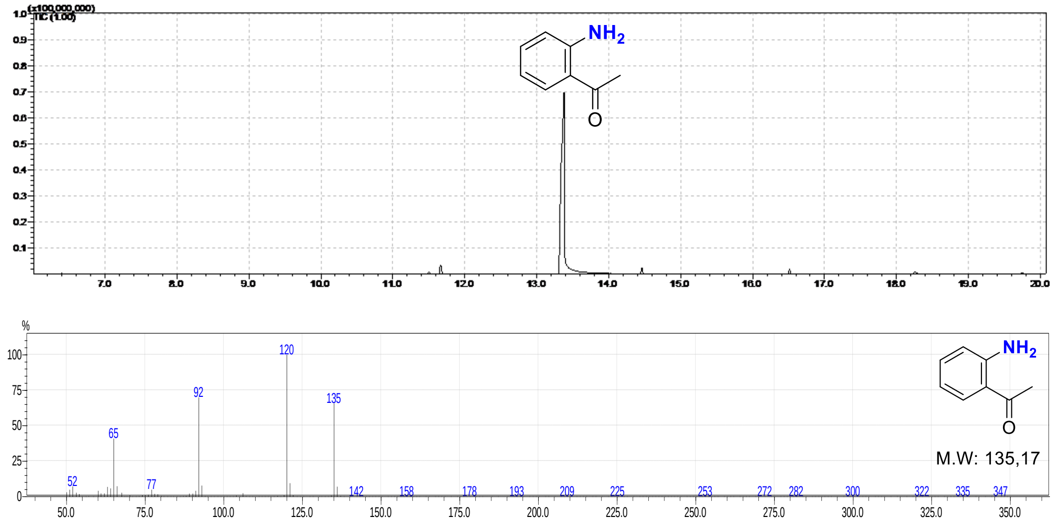

Supplementary Figure 20. Gas chromatography and mass spectra of the product from the photoenzymatic reduction of 2’-nitroacetophenone.

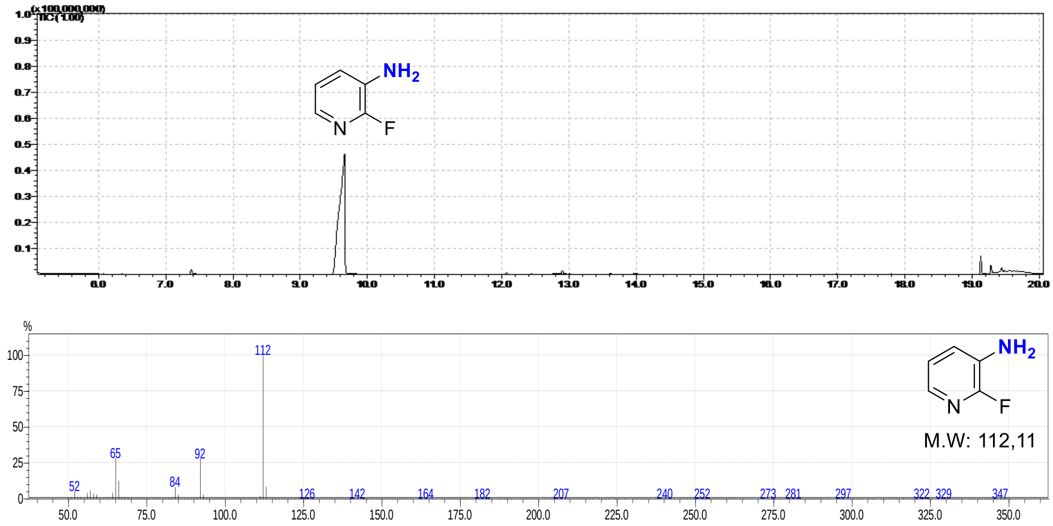

Supplementary Figure 21. Gas chromatography and mass spectra of the product from the photoenzymatic reduction of 2-fluoro-3-nitropyridine.

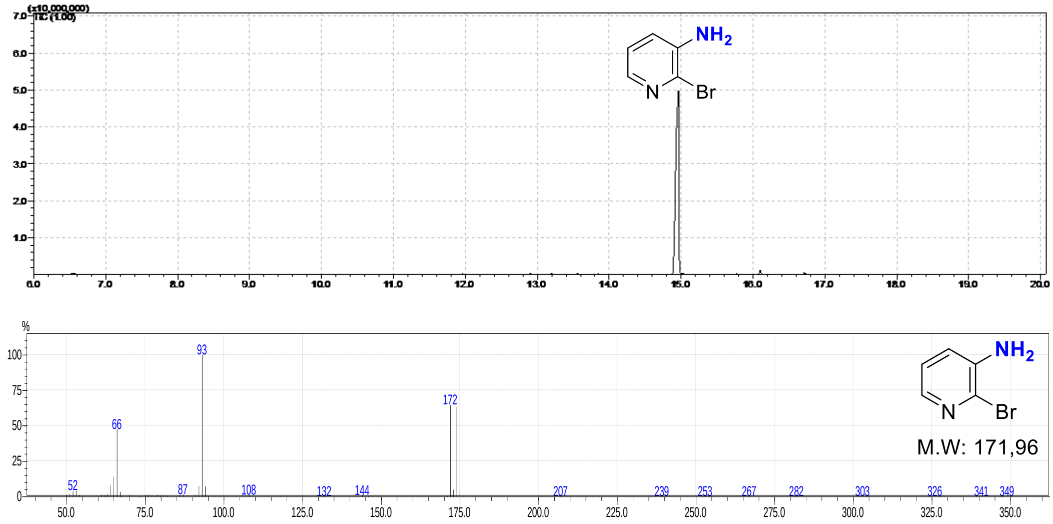

Supplementary Figure 22. Gas chromatography and mass spectra of the product from the photoenzymatic reduction of 2-bromo-3-nitropyridine.

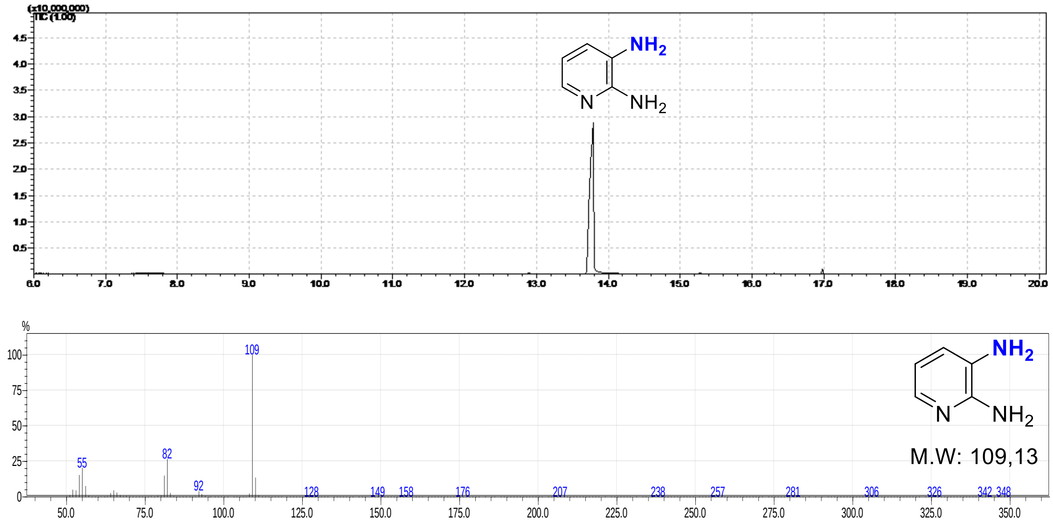

Supplementary Figure 23. Gas chromatography and mass spectra of the product from the photoenzymatic reduction of 3-nitropyridin-2-amine.

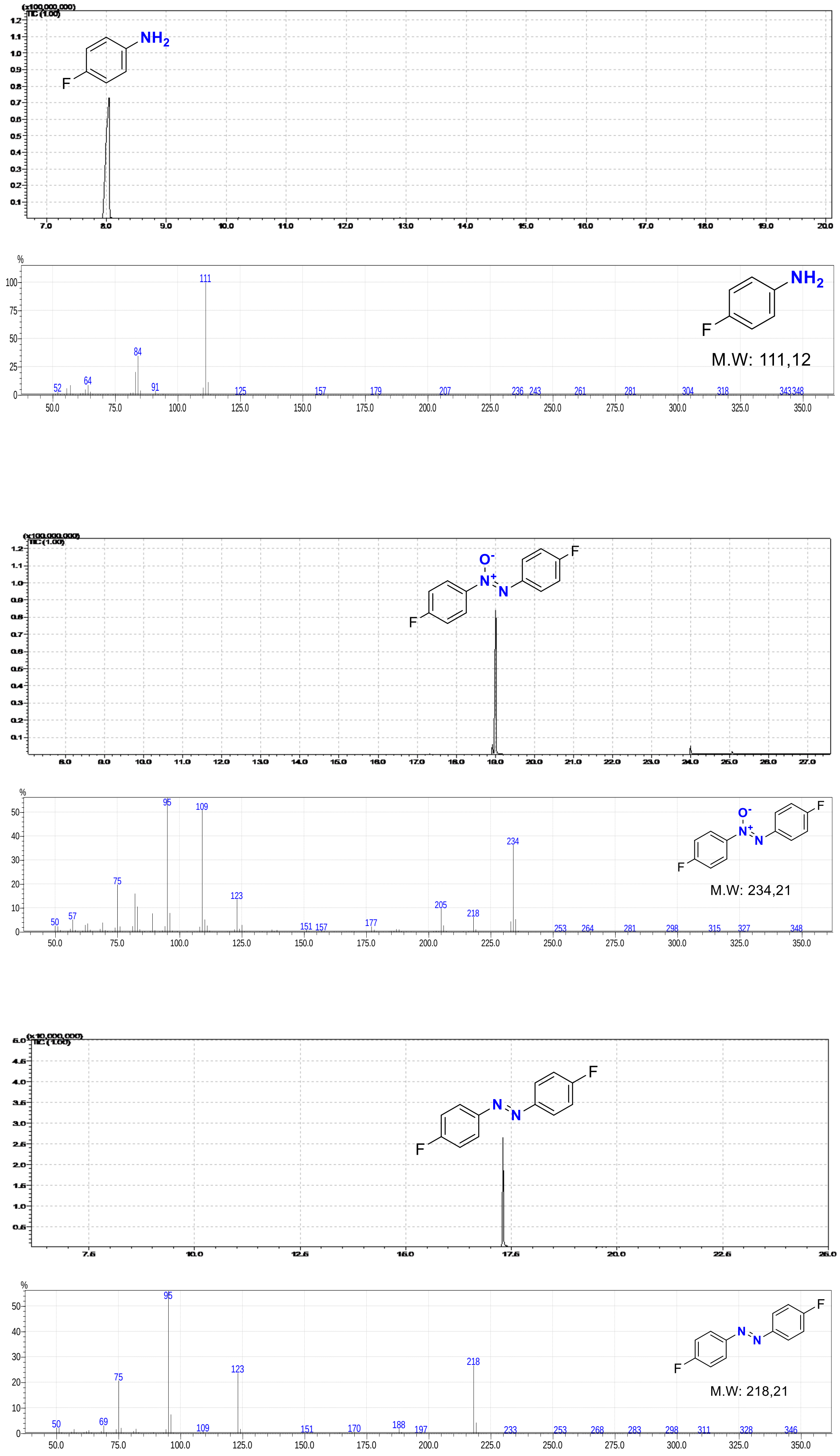

Supplementary Figure 24. Gas chromatography and mass spectra of the products from the photoenzymatic reductions of 1-fluoro-4-nitrobenzene.

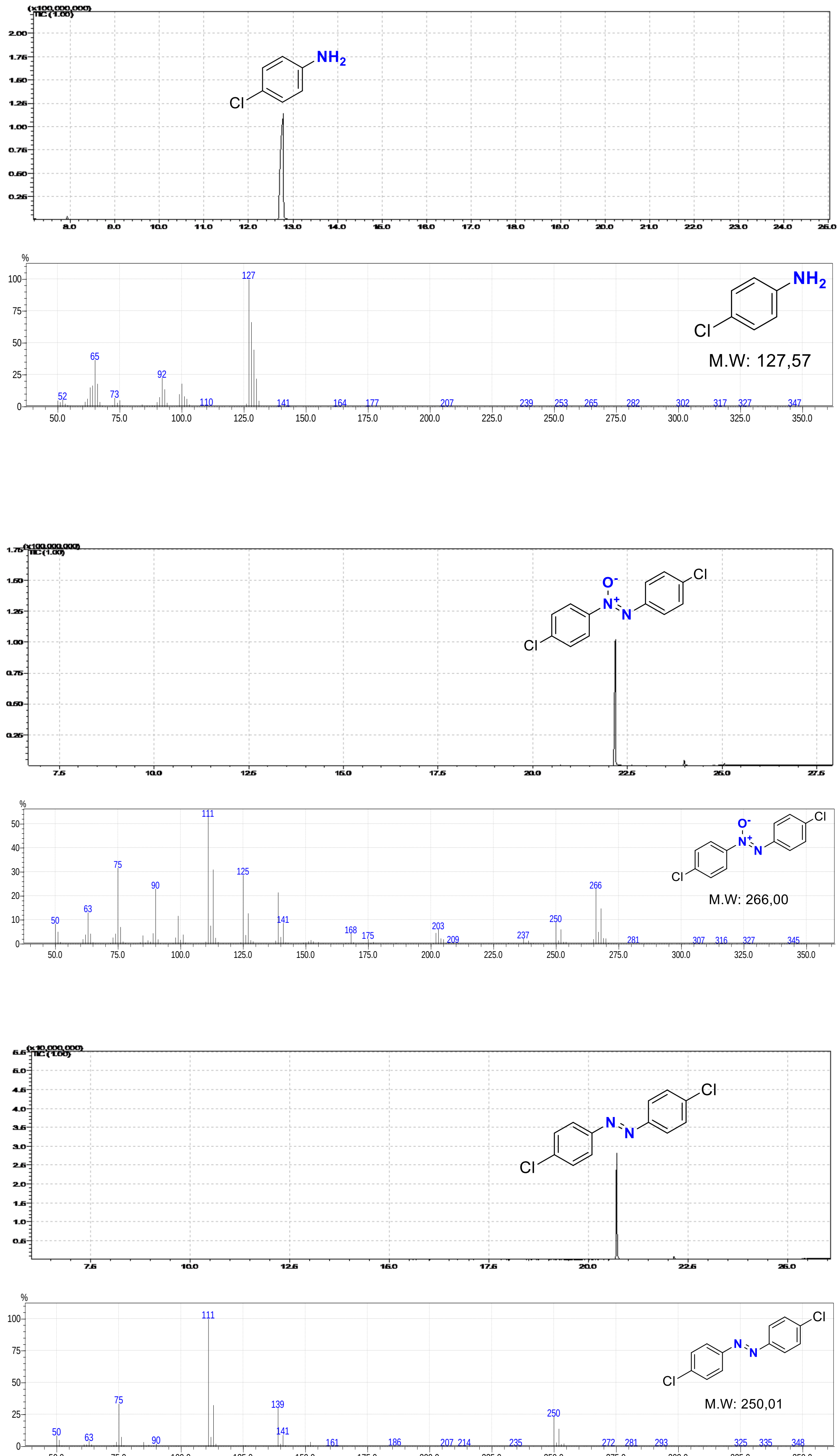

Supplementary Figure 25. Gas chromatography and mass spectra of the products from the photoenzymatic reductions of 1-chloro-4-nitrobenzene.

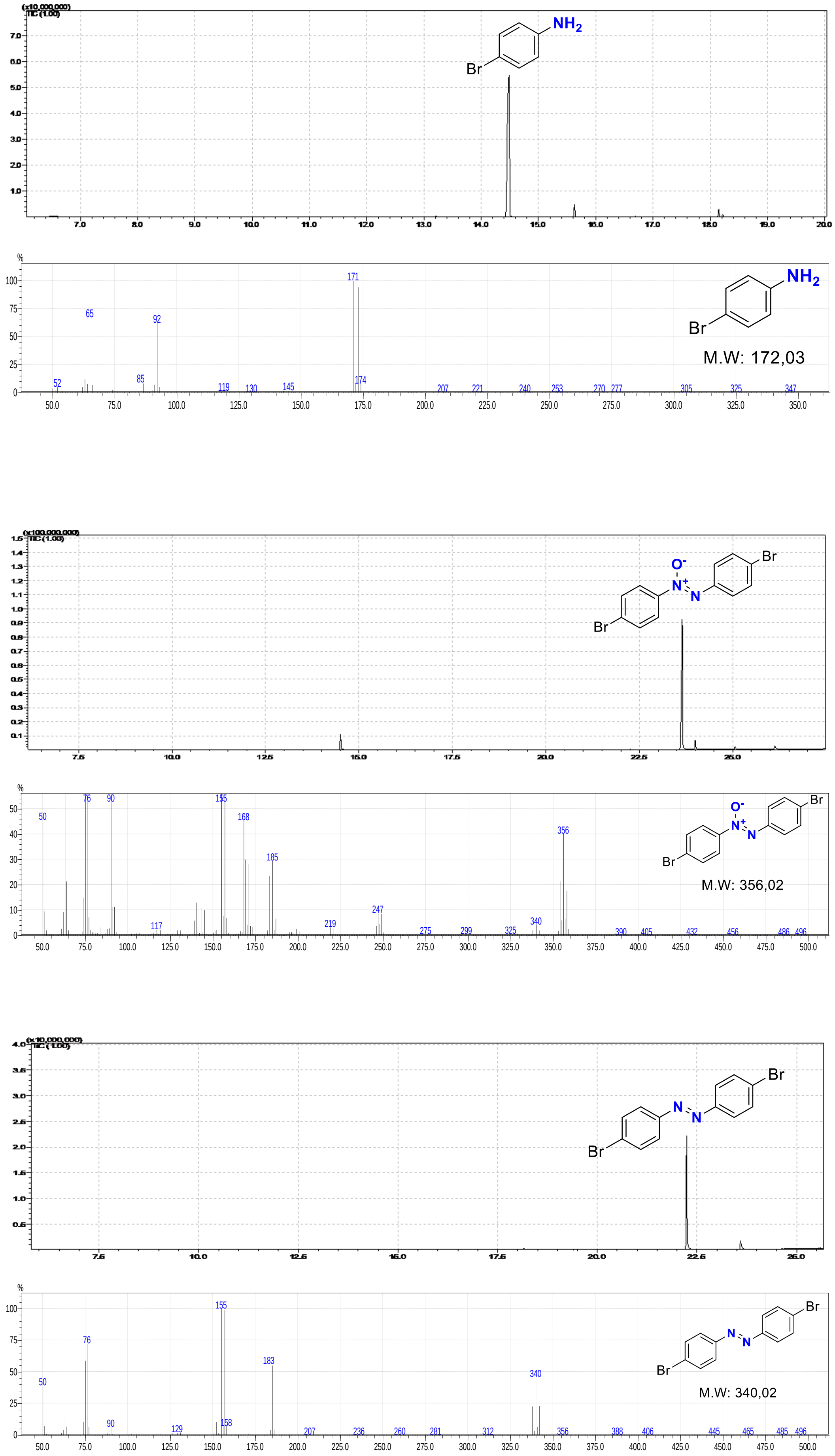

Supplementary Figure 26. Gas chromatography and mass spectra of the products from the photoenzymatic reductions of 1-bromo-4-nitrobenzene.

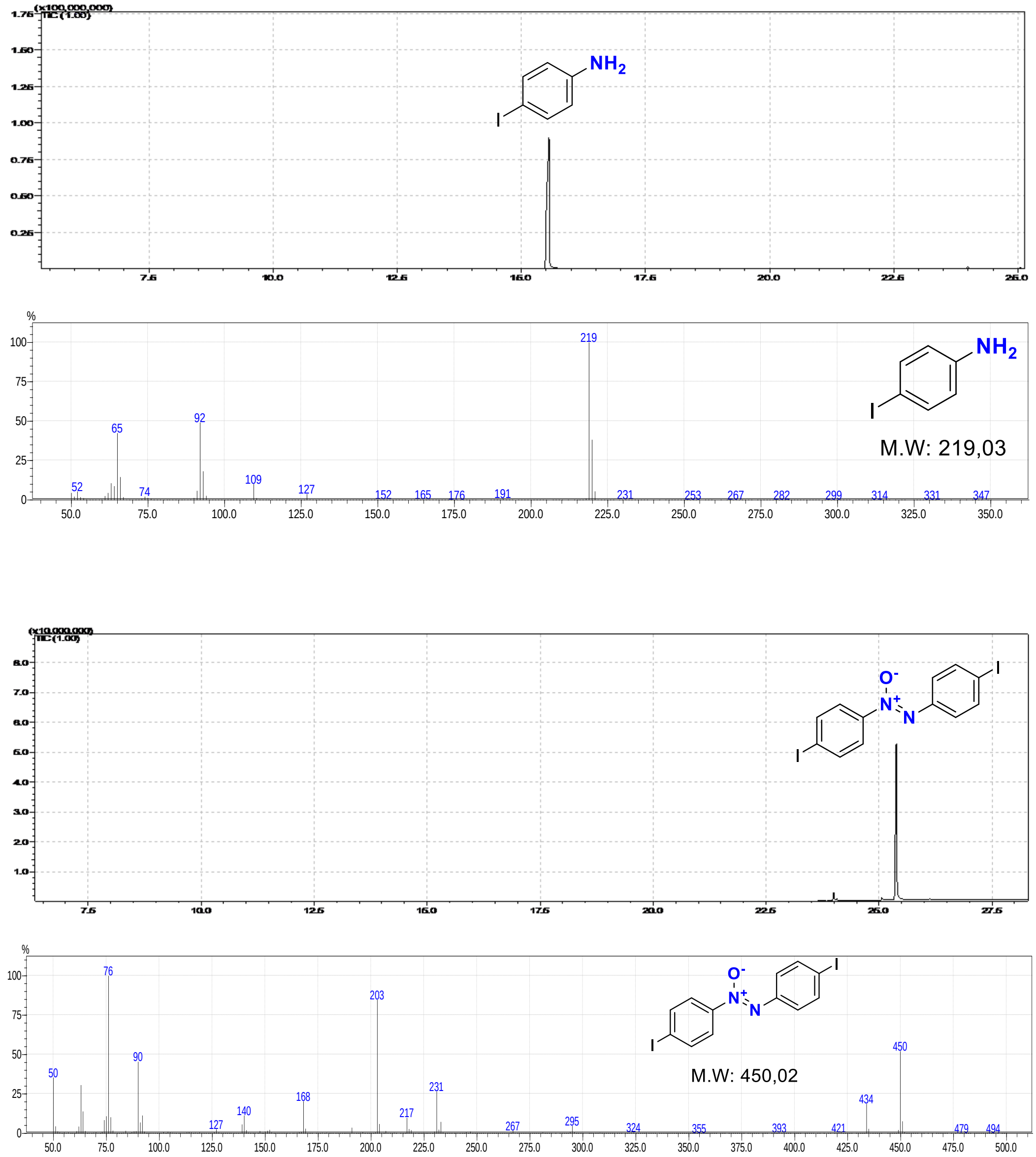

Supplementary Figure 27. Gas chromatography and mass spectra of the products from the photoenzymatic reductions of 1-iodo-4-nitrobenzene.

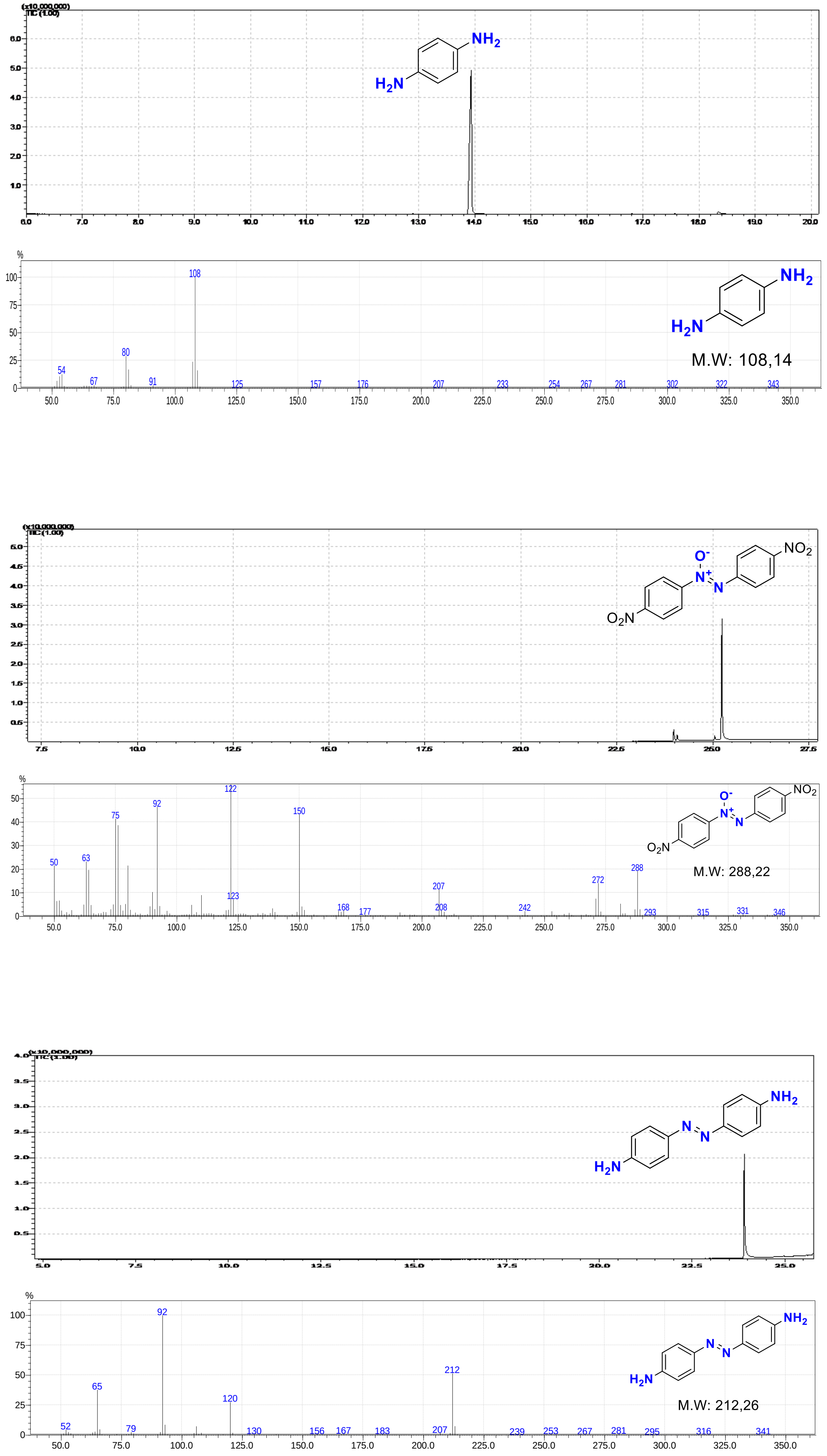

Supplementary Figure 28. Gas chromatography and mass spectra of the products from the photoenzymatic reductions of 1,4-dinitrobenzene.

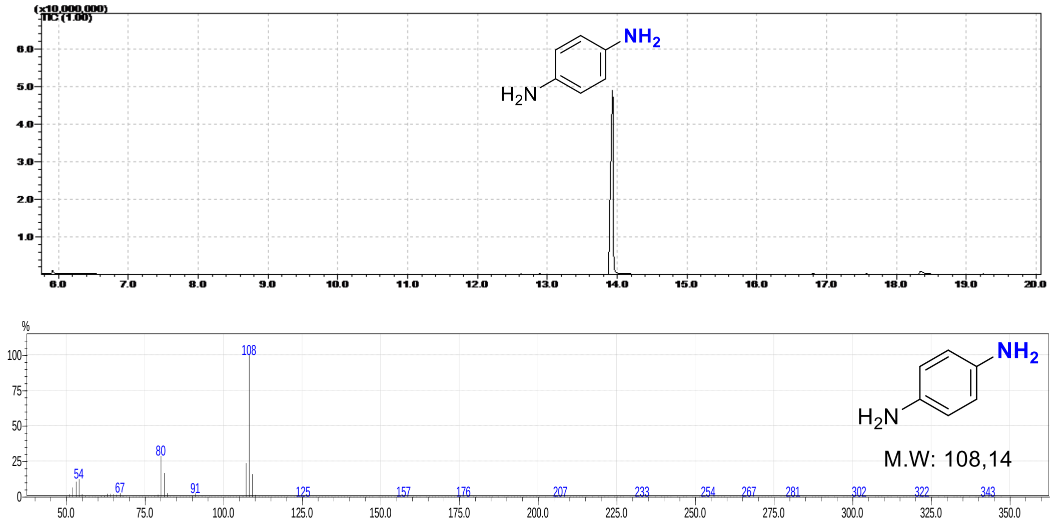

Supplementary Figure 29. Gas chromatography and mass spectra of the product from the photoenzymatic reduction of 4-nitroaniline.

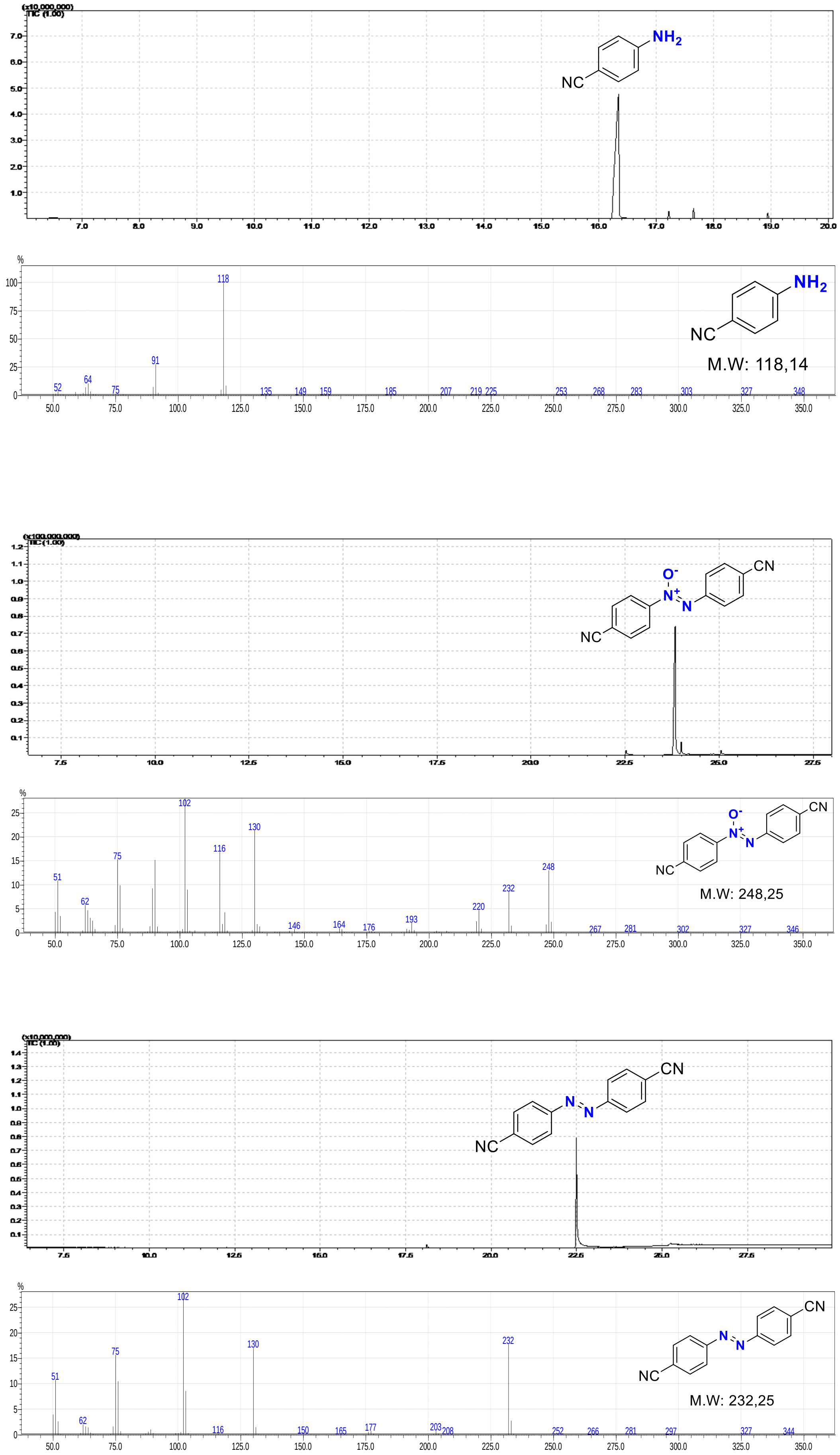

Supplementary Figure 30. Gas chromatography and mass spectra of the products from the photoenzymatic reductions of 4-nitrobenzonitrile.

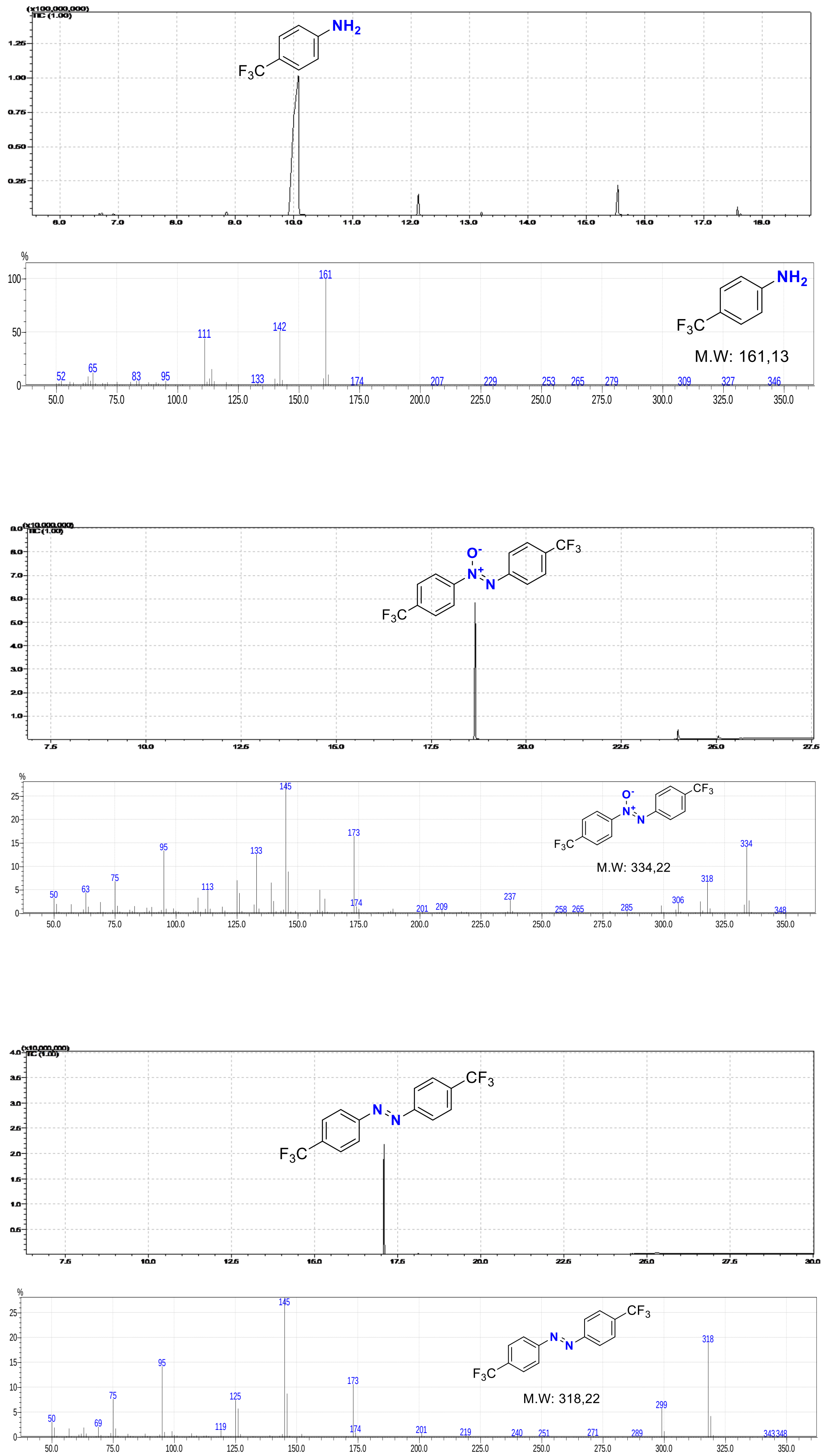

Supplementary Figure 31. Gas chromatography and mass spectra of the products from the photoenzymatic reductions of 1-nitro-4-(trifluoromethyl)benzene.

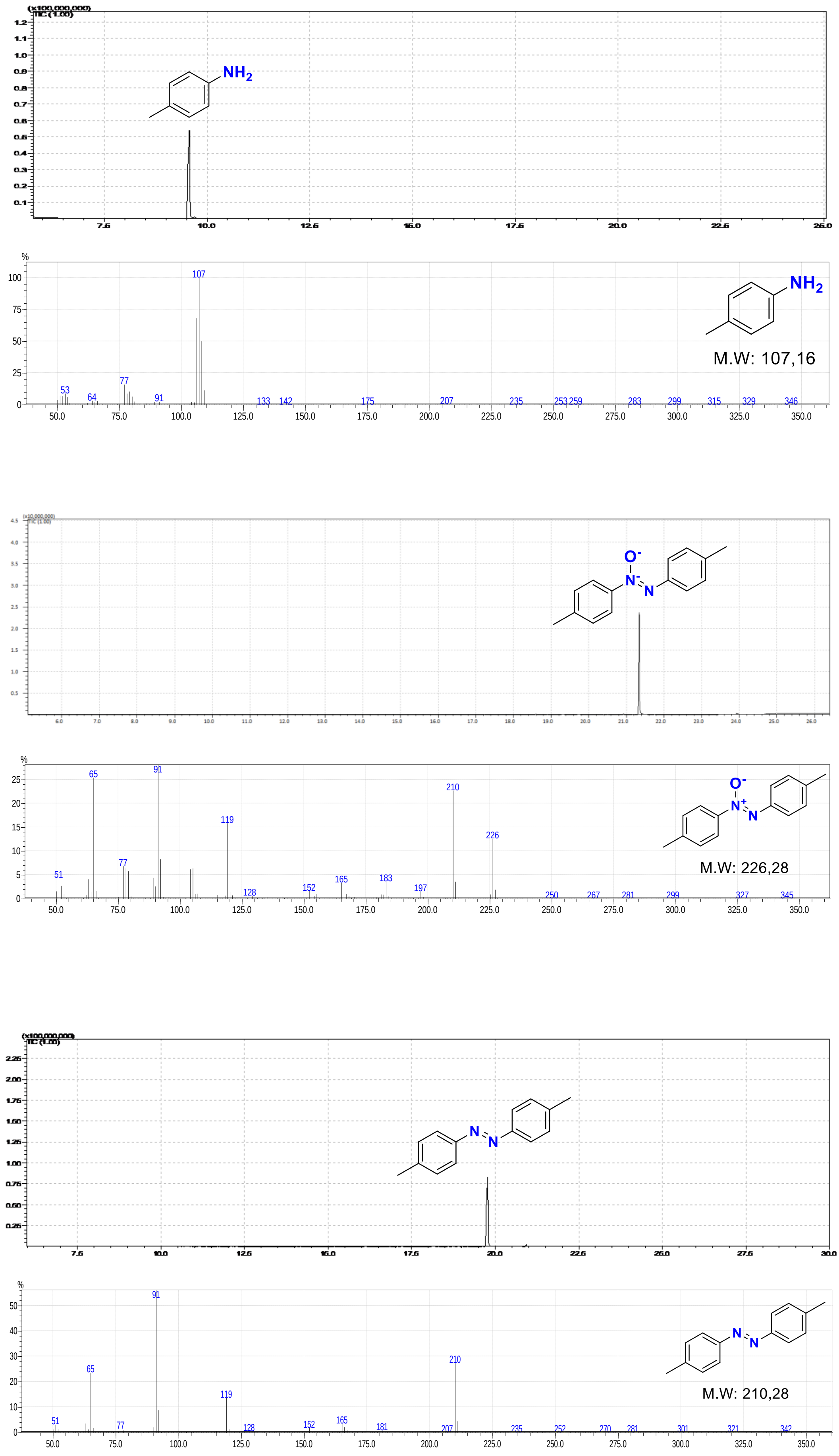

Supplementary Figure 32. Gas chromatography and mass spectra of the products from the photoenzymatic reductions of 4-nitrotoluene.

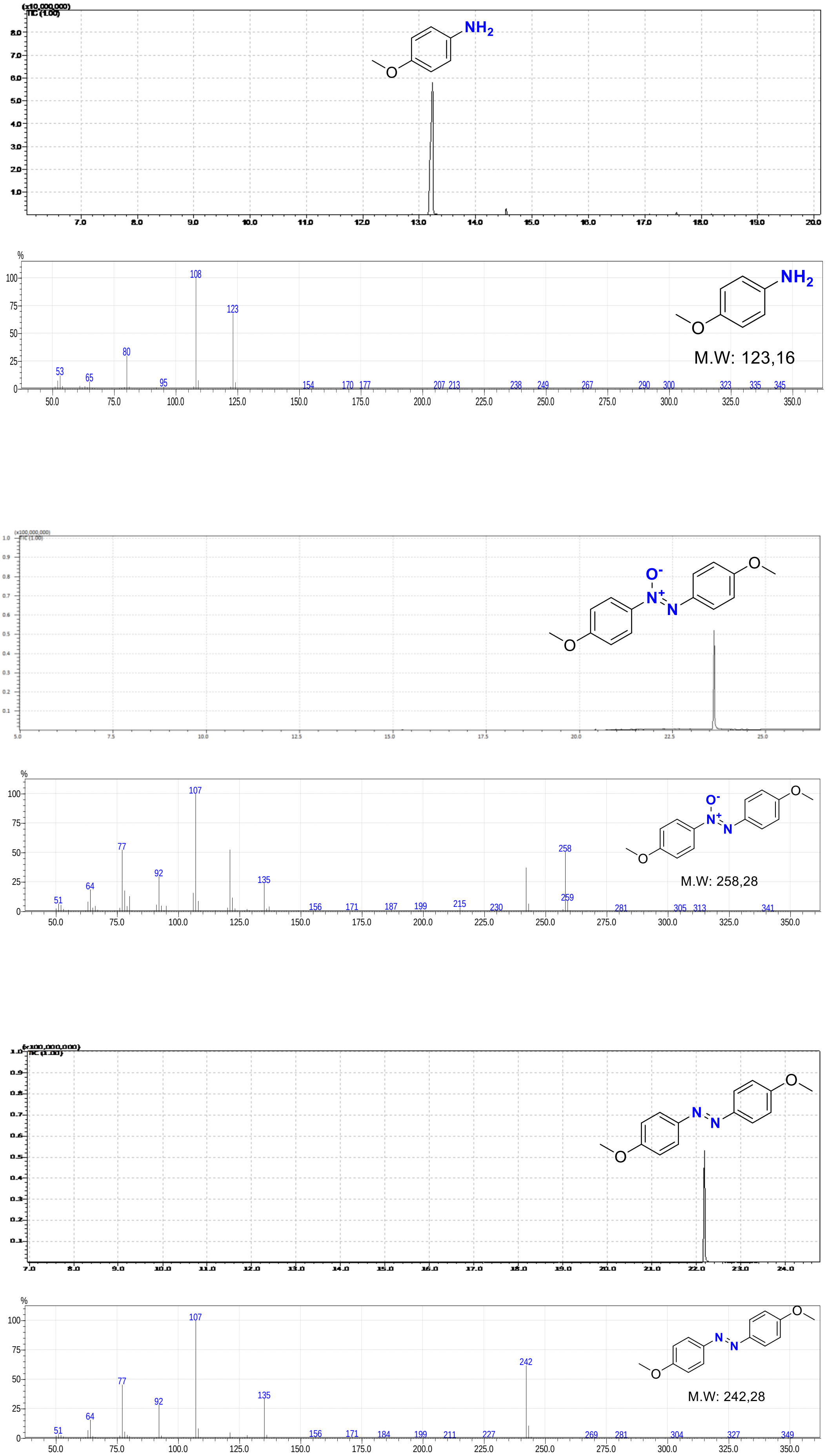

Supplementary Figure 33. Gas chromatography and mass spectra of the products from the photoenzymatic reductions of 4-nitroanisole.

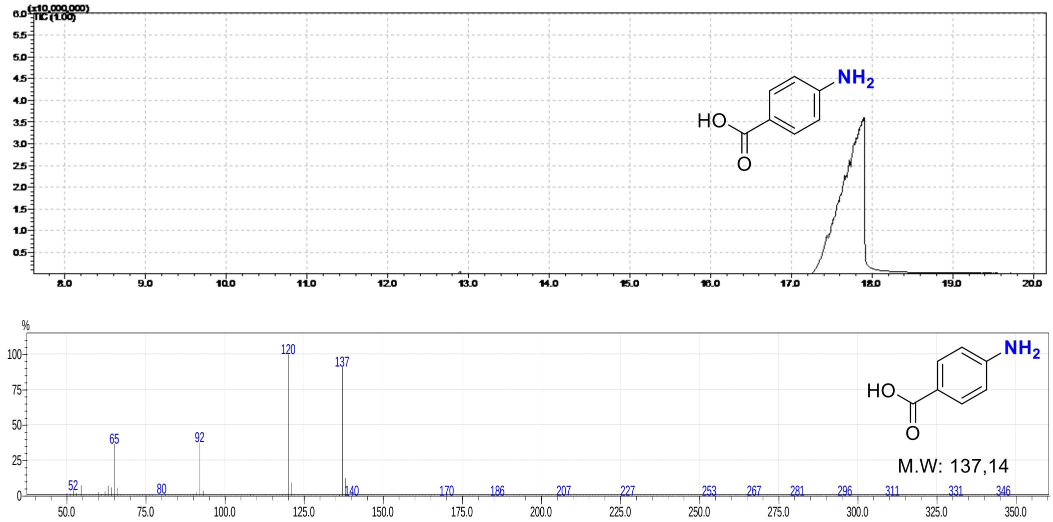

Supplementary Figure 34. Gas chromatography and mass spectra of the product from the photoenzymatic reduction of 4-nitrobenzoic acid.

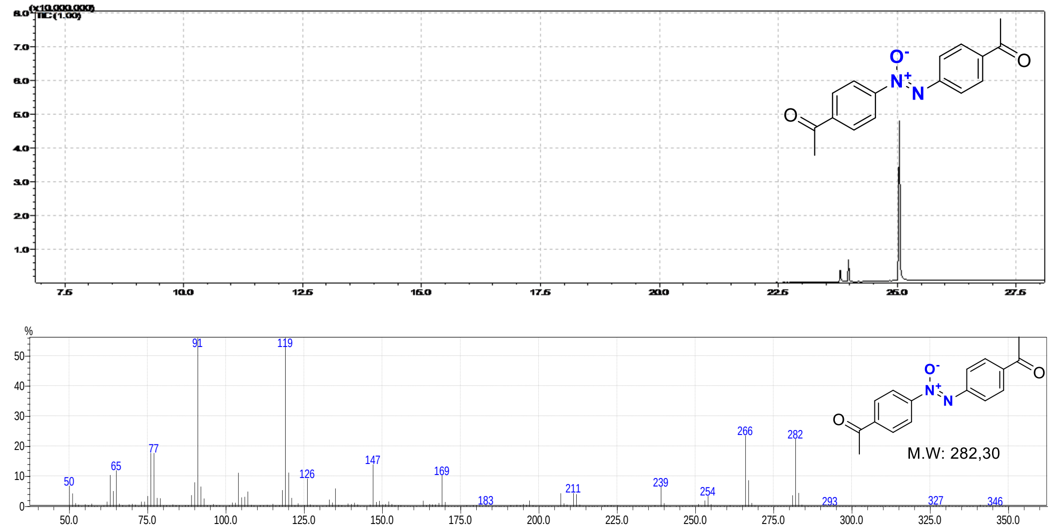

Supplementary Figure 35. Gas chromatography and mass spectra of the product from the photoenzymatic reduction of 4-nitroacetophenone.

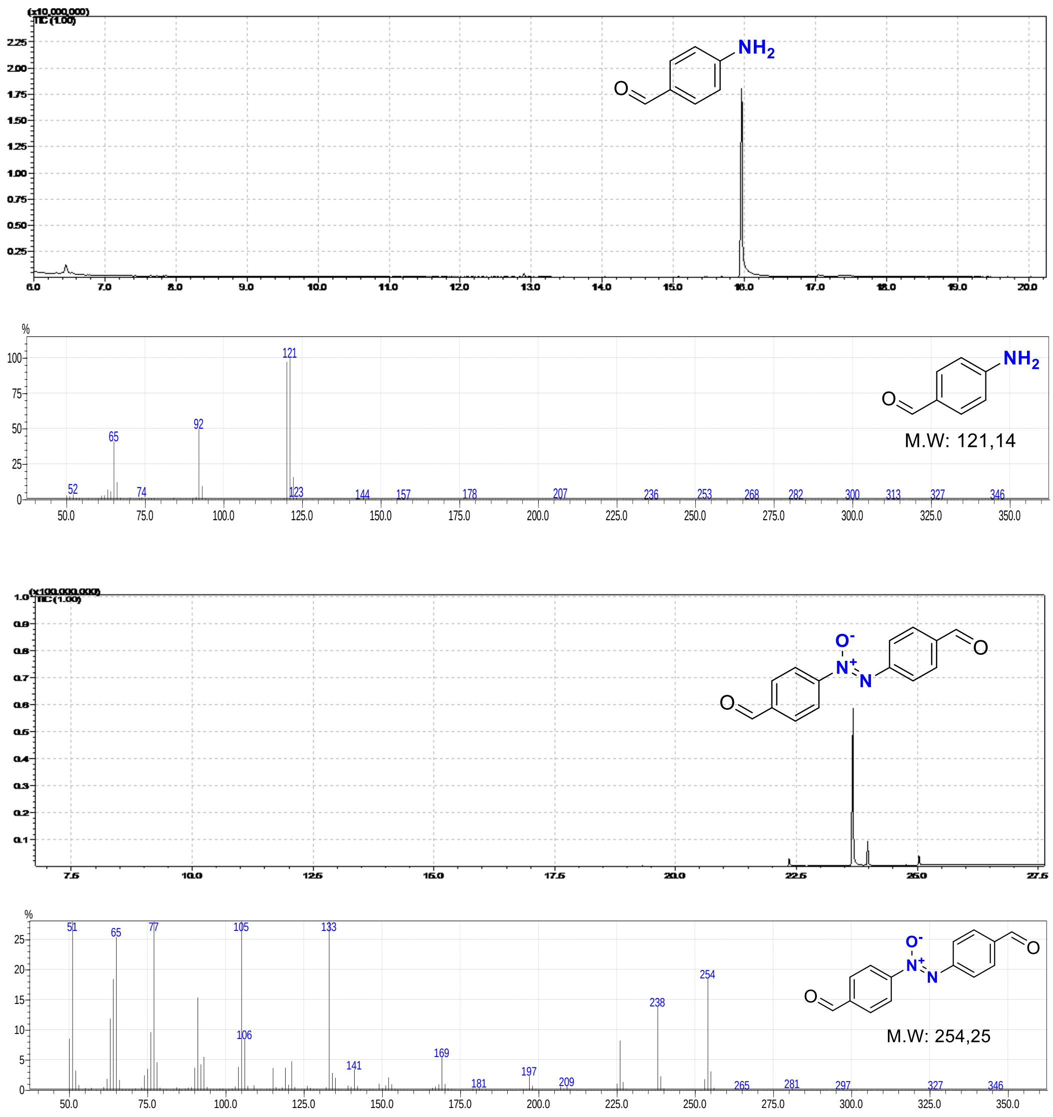

Supplementary Figure 36. Gas chromatography and mass spectra of the products from the photoenzymatic reductions of 4-nitrobenzaldehyde.

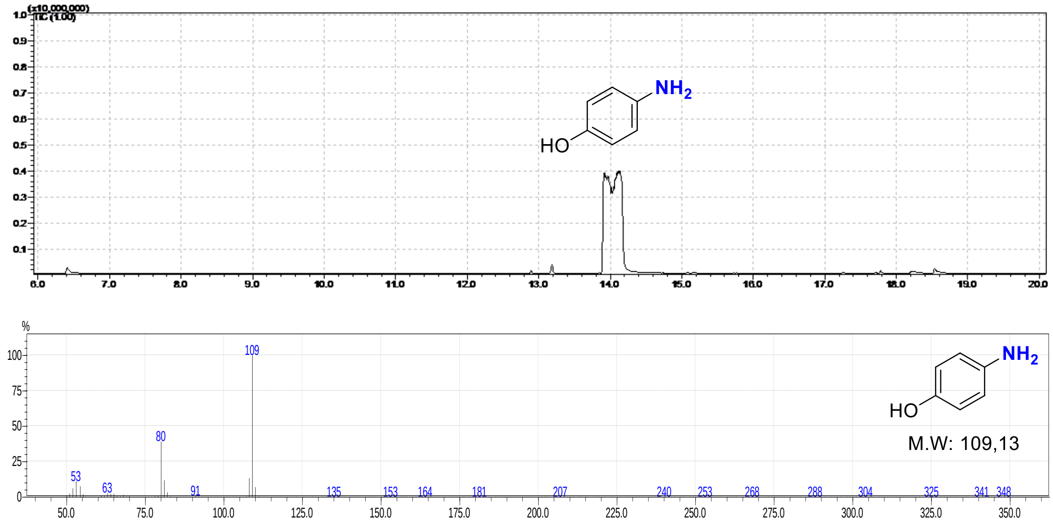

Supplementary Figure 37. Gas chromatography and mass spectra of the product from the photoenzymatic reduction of 4-nitrophenol. The peak corresponds to one product with an identified M.W. of 109.13; the elution pattern was confirmed with a commercial reference, giving the same pattern using the same column and GC conditions.

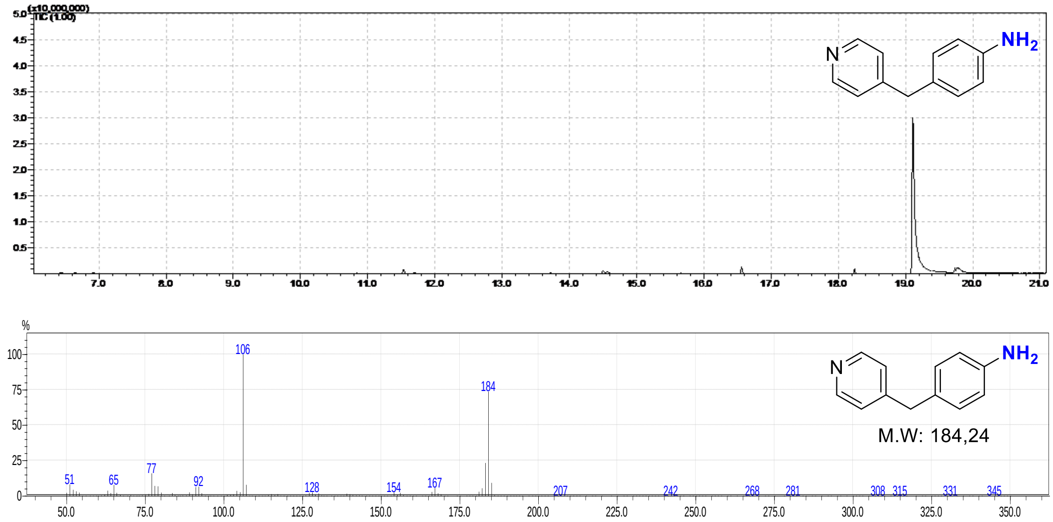

Supplementary Figure 38. Gas chromatography and mass spectra of the product from the photoenzymatic reduction of 4-(4-nitrobenzyl)pyridine.

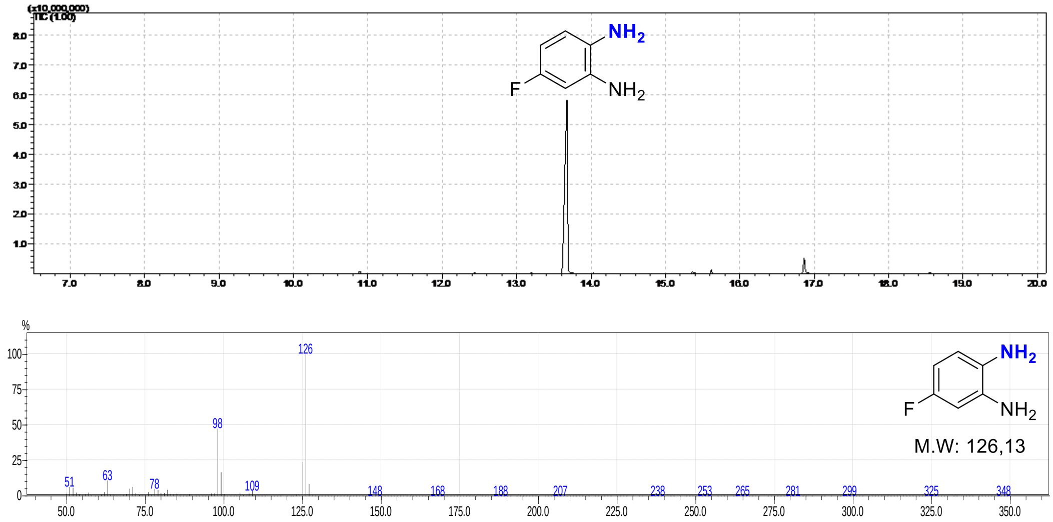

Supplementary Figure 39. Gas chromatography and mass spectra of the product from the photoenzymatic reduction of 5-fluoro-2-nitroaniline.

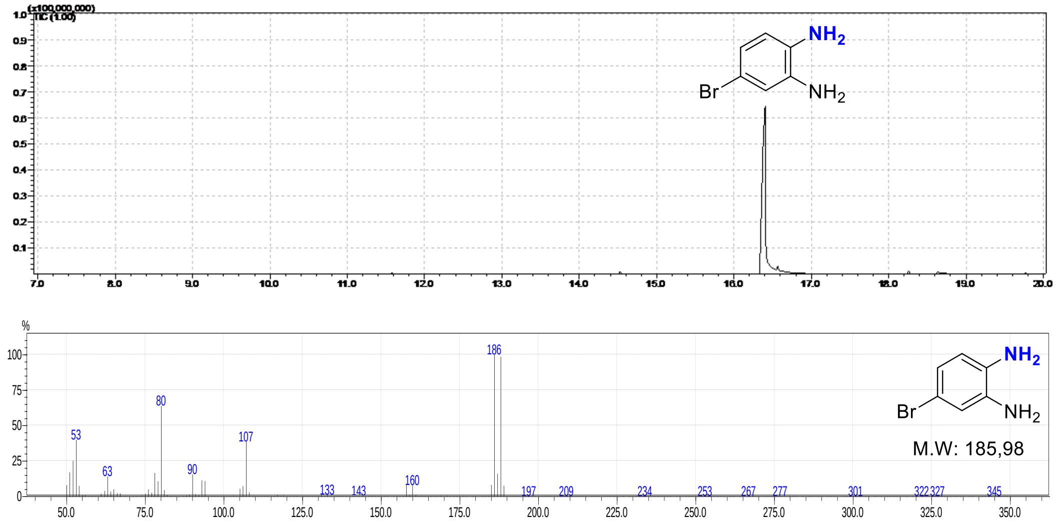

Supplementary Figure 40. Gas chromatography and mass spectra of the product from the photoenzymatic reduction of 5-bromo-2-nitroaniline.

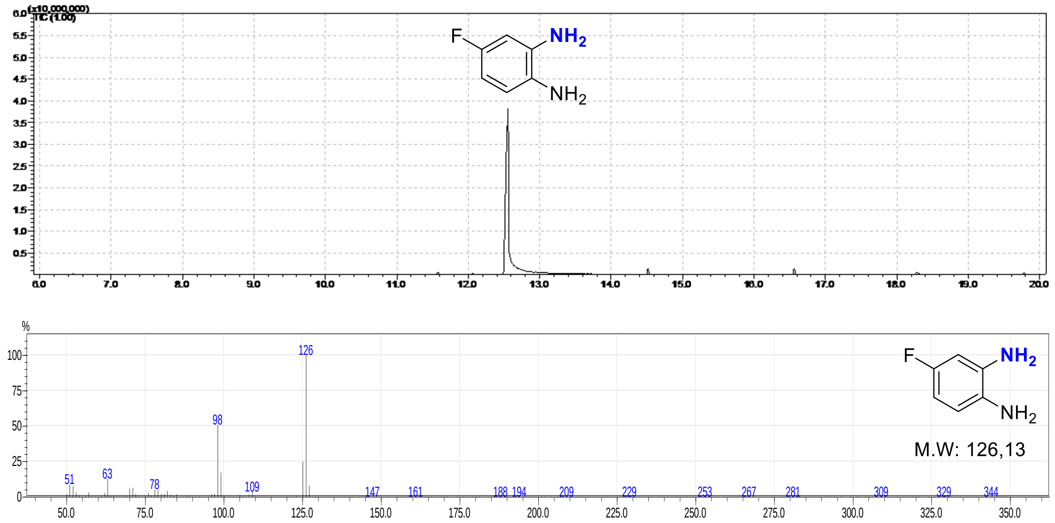

Supplementary Figure 41. Gas chromatography and mass spectra of the products from the photoenzymatic reduction of 4-fluoro-2-nitroaniline.

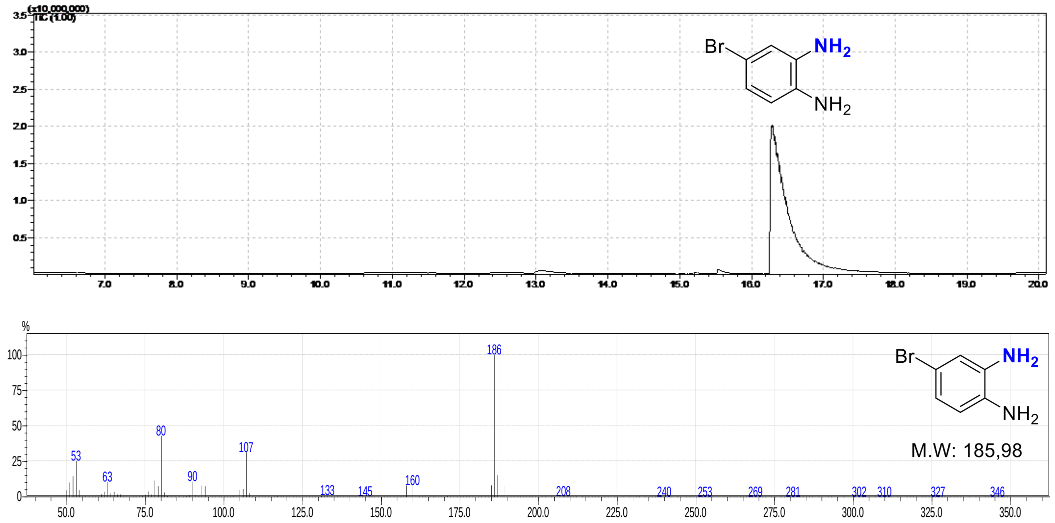

Supplementary Figure 42. Gas chromatography and mass spectra of the products from the photoenzymatic reduction of 4-bromo-2-nitroaniline.

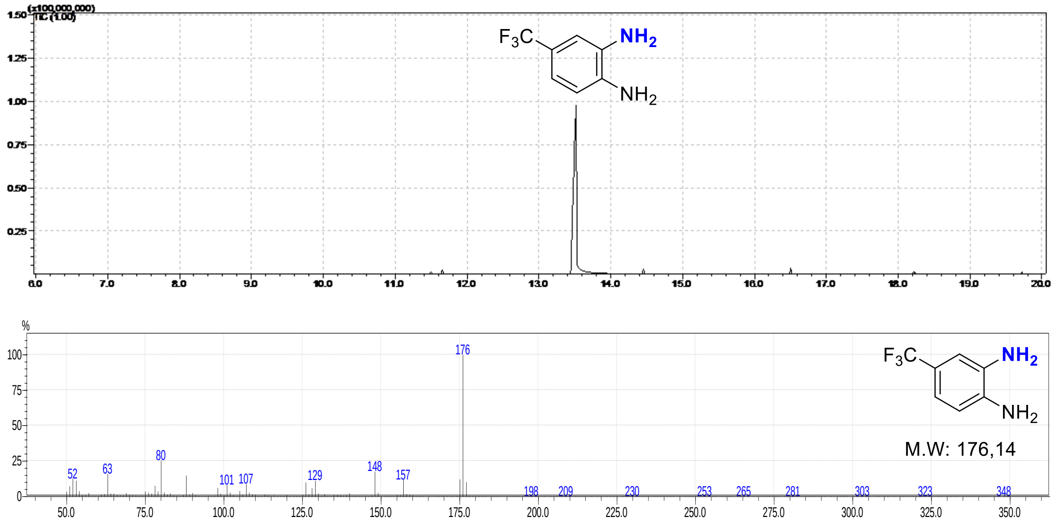

Supplementary Figure 43. Gas chromatography and mass spectra of the products from the photoenzymatic reduction of 4-trifluoromethyl-2-nitroaniline.

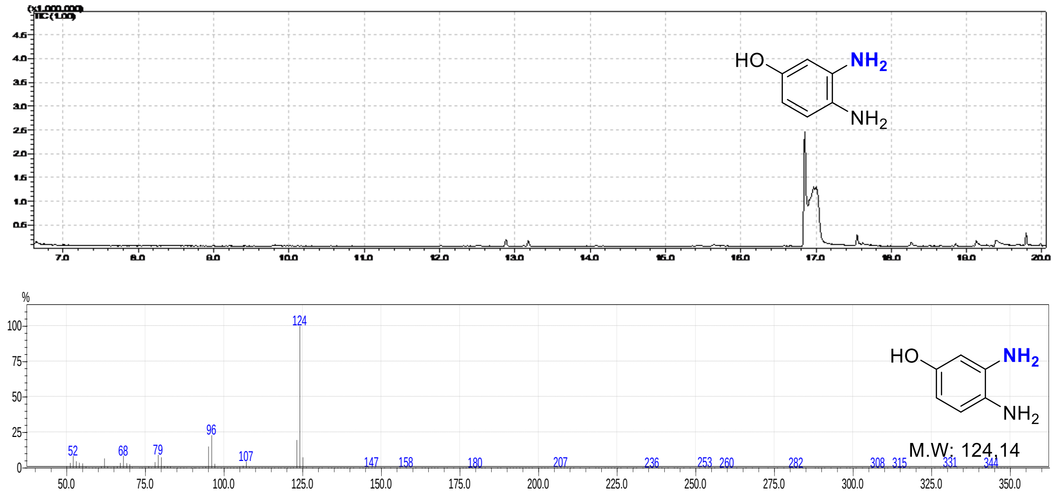

Supplementary Figure 44. Gas chromatography and mass spectra of the products from the photoenzymatic reduction of 4-hydroxy-2-nitroaniline. The peak corresponds to one product with an identified M.W. of 124.14; the elution pattern was confirmed with a commercial reference, giving the same pattern using the same column and GC conditions.

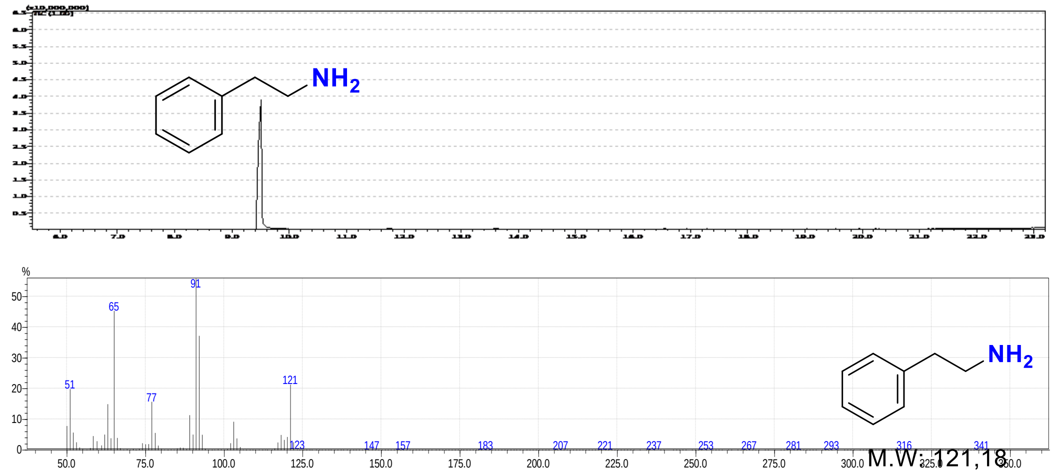

Supplementary Figure 45. Gas chromatography (top) and mass (bottom) spectra from photoenzymatically produced phenethylamine (**68**).

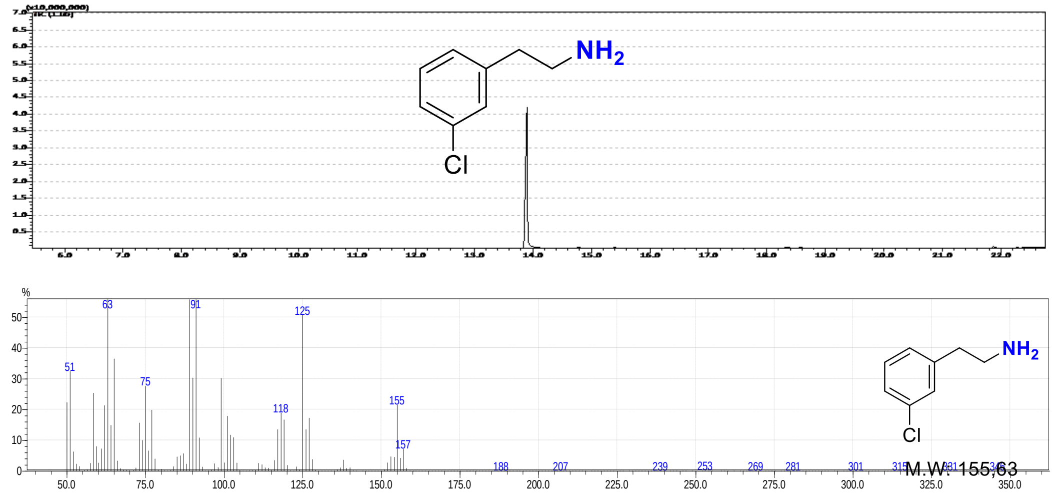

Supplementary Figure 46. Gas chromatography (top) and mass (bottom) spectra from photoenzymatically produced 3-chlorophenethylamine (**69**).

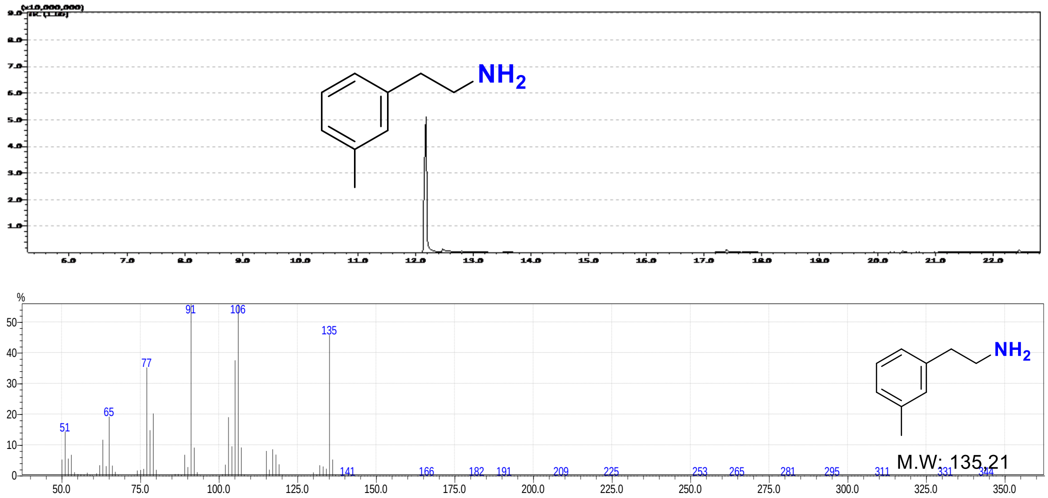

Supplementary Figure 47. Gas chromatography (top) and mass (bottom) spectra from photoenzymatically produced 3-methylphenethylamine (**70**).

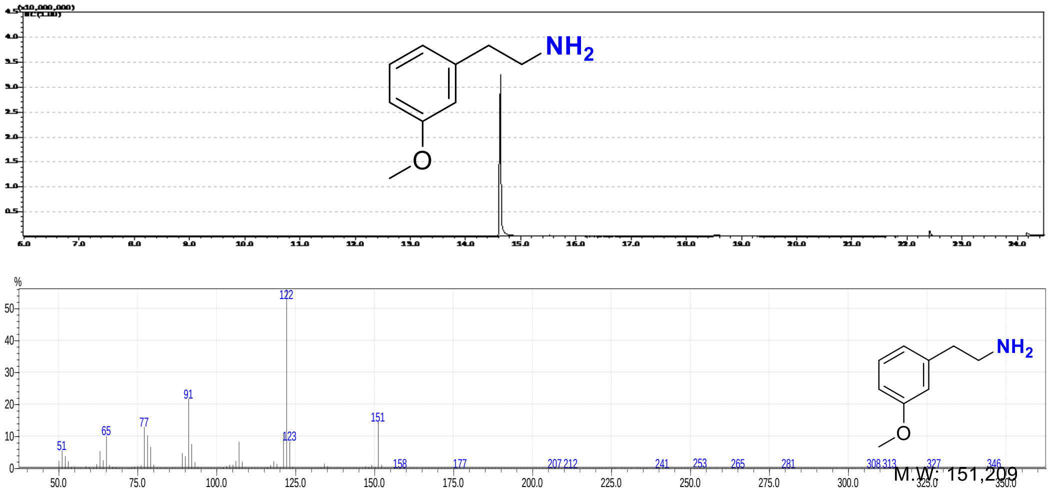

Supplementary Figure 48. Gas chromatography (top) and mass (bottom) spectra from photoenzymatically produced 3-methoxyphenethylamine (**71**).

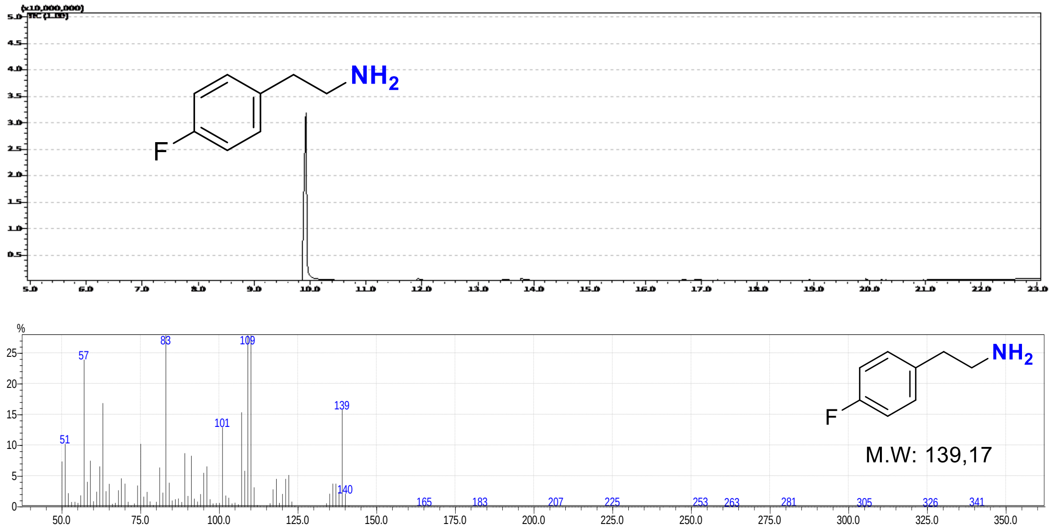

Supplementary Figure 49. Gas chromatography (top) and mass (bottom) spectra from photoenzymatically produced 4-fluorophenethylamine (**72**).

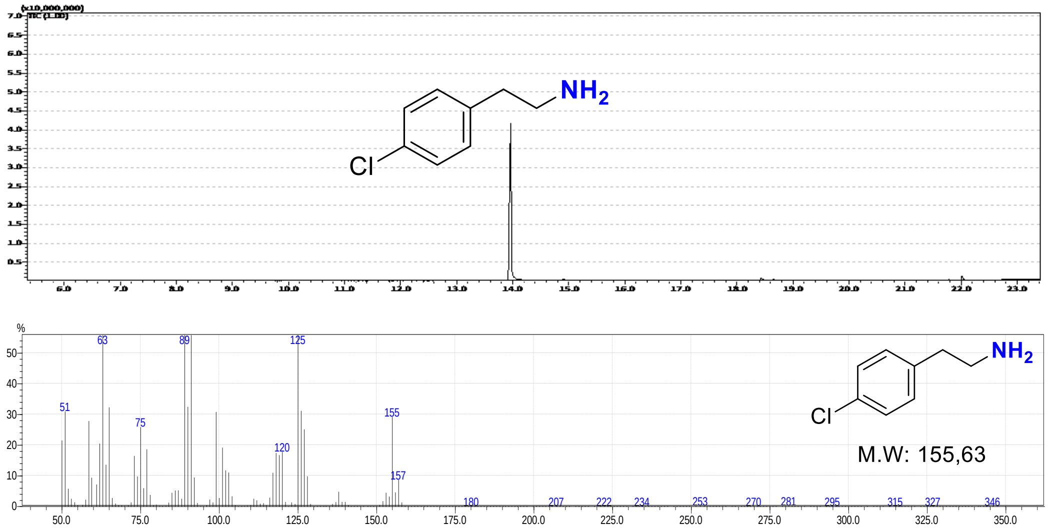

Supplementary Figure 50. Gas chromatography (top) and mass (bottom) spectra from photoenzymatically produced 4-chlorophenethylamine (**73**).

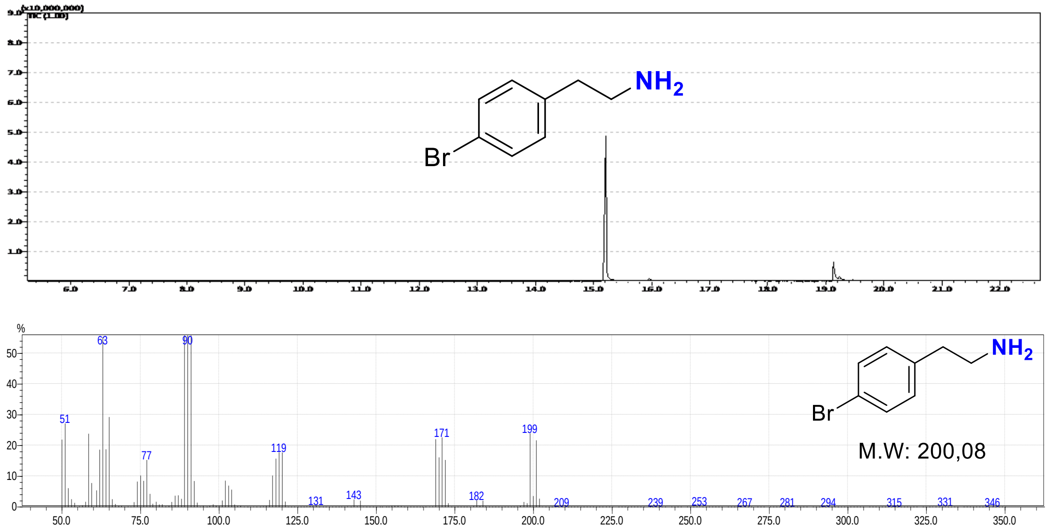

Supplementary Figure 51. Gas chromatography (top) and mass (bottom) spectra from photoenzymatically produced 4-bromophenethylamine (**74**).

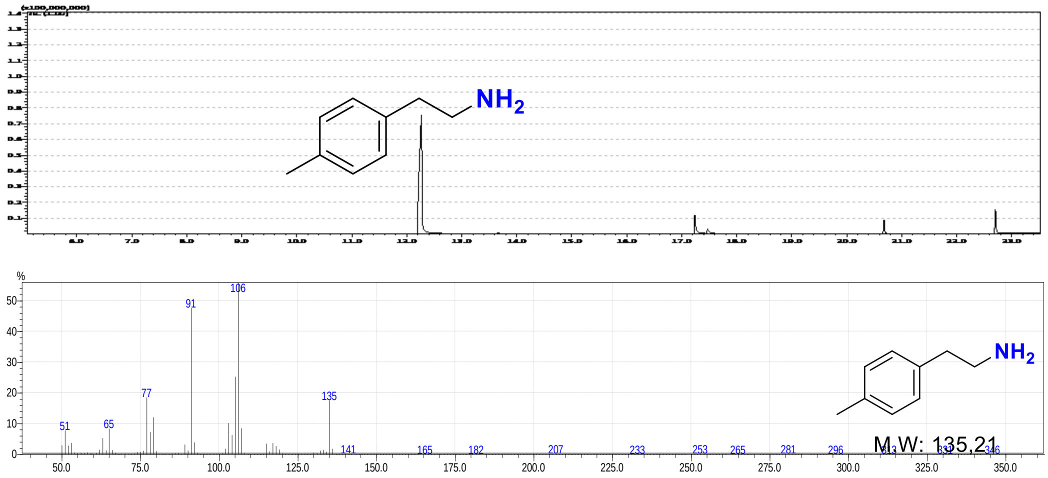

Supplementary Figure 52. Gas chromatography (top) and mass (bottom) spectra from photoenzymatically produced 4-methylphenethylamine (**75**).

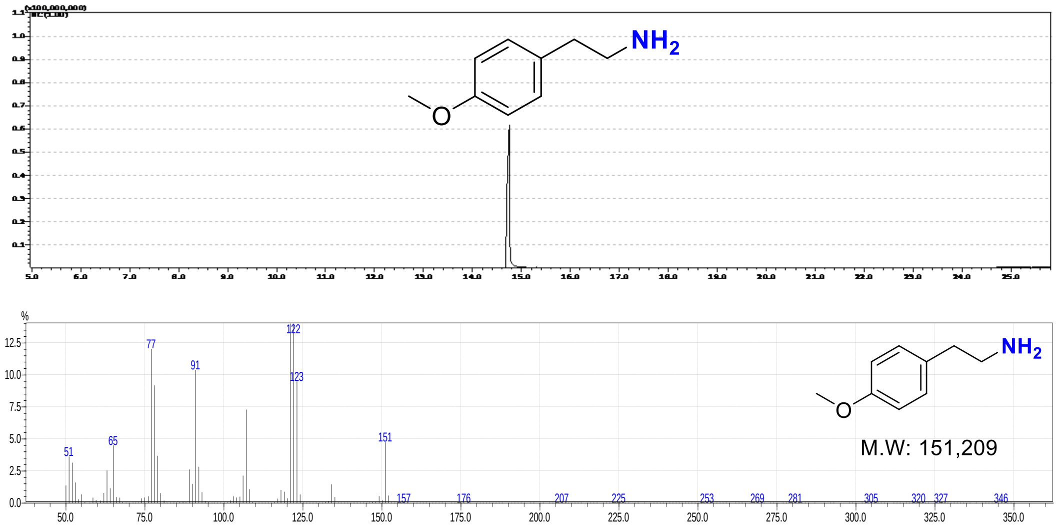

Supplementary Figure 53. Gas chromatography (top) and mass (bottom) spectra from photoenzymatically produced 4-methoxyphenethylamine (**76**).

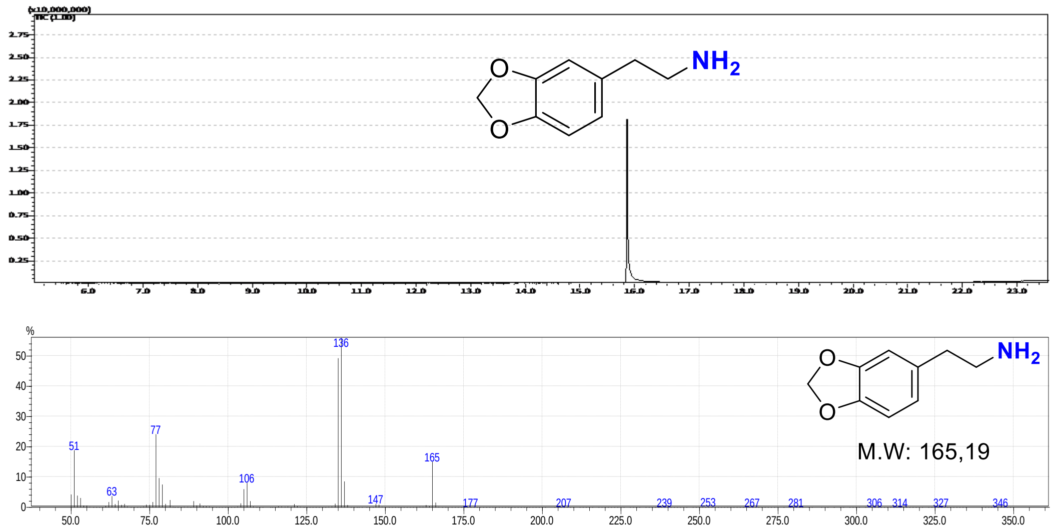

Supplementary Figure 54. Gas chromatography (top) and mass (bottom) spectra from photoenzymatically produced 1,3-benzodioxole-5-ethanamine (**77**).

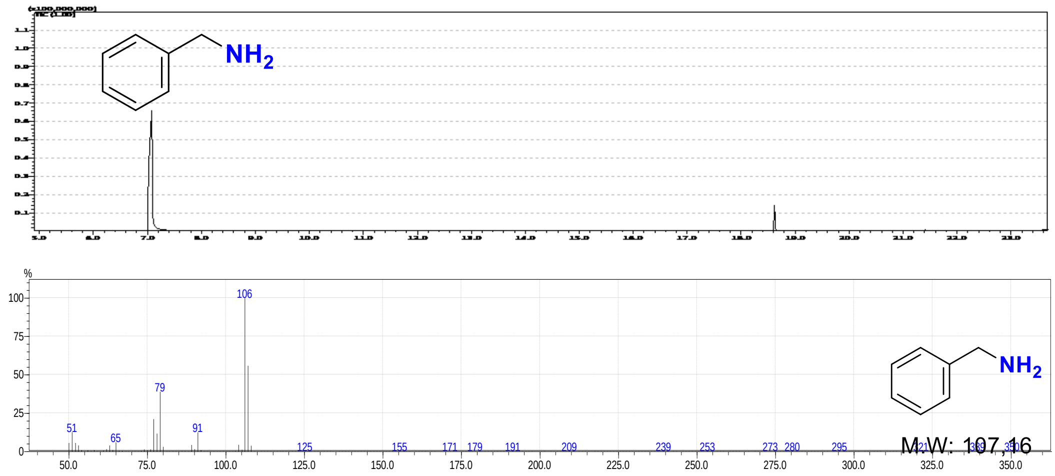

Supplementary Figure 55. Gas chromatography (top) and mass (bottom) spectra from photoenzymatically produced benzylamine (**78**).

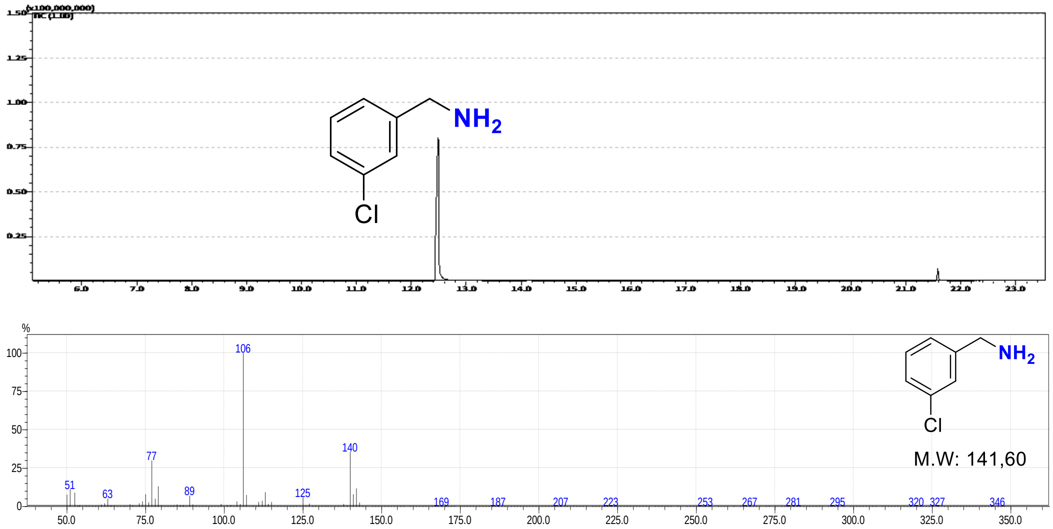

Supplementary Figure 56. Gas chromatography (top) and mass (bottom) spectra from photoenzymatically produced 3-chlorobenzylamine (**79**).

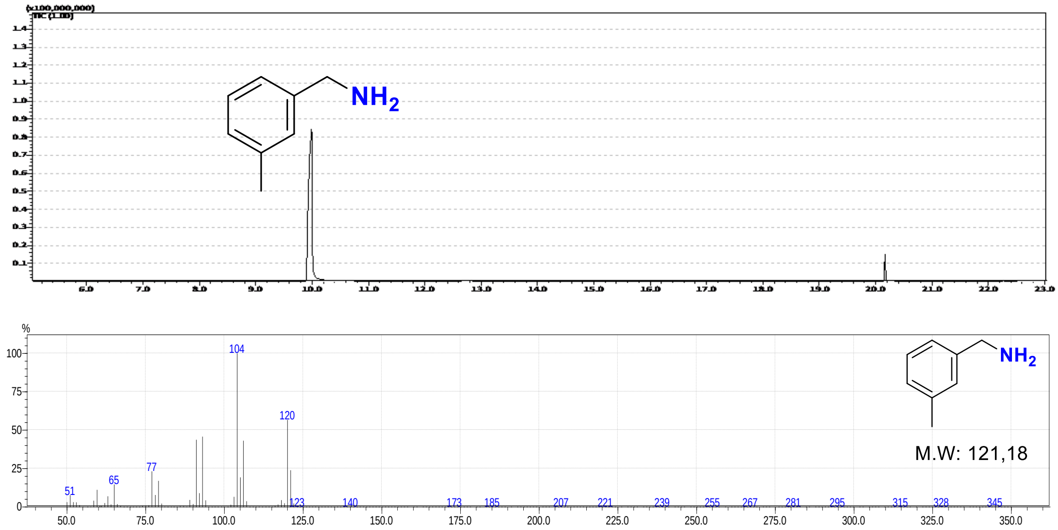

Supplementary Figure 57. Gas chromatography (top) and mass (bottom) spectra from photoenzymatically produced 3-methylbenzylamine (**80**).

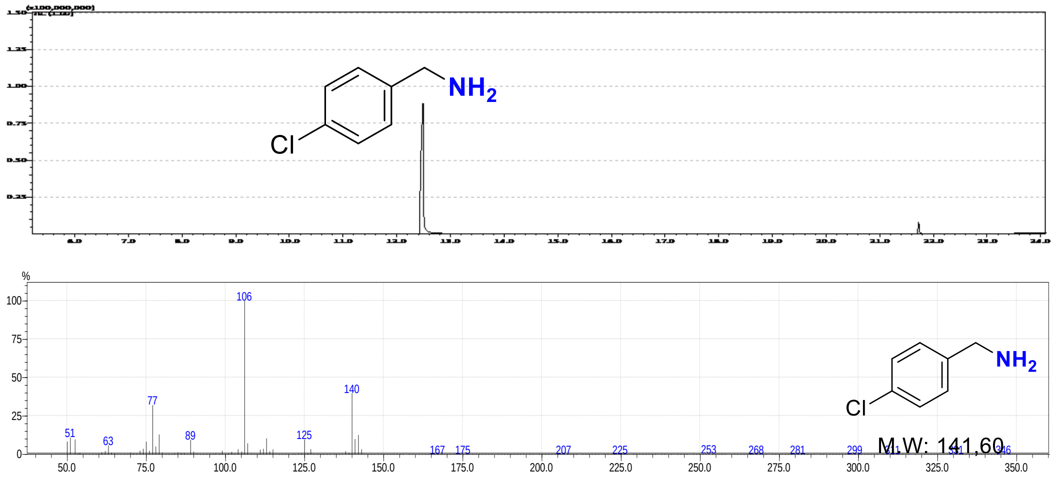

Supplementary Figure 58. Gas chromatography (top) and mass (bottom) spectra from photoenzymatically produced 4-chlorobenzylamine (**81**).

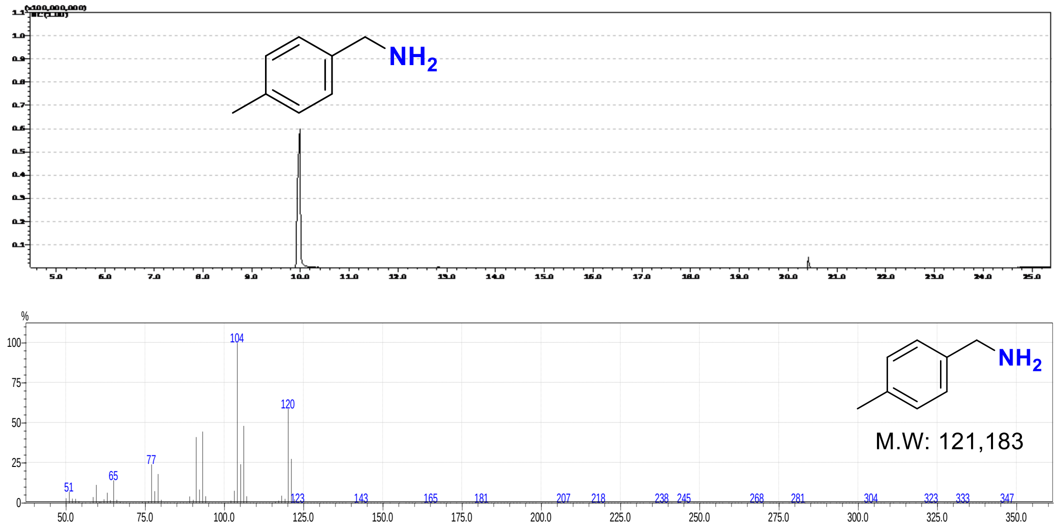

Supplementary Figure 59. Gas chromatography (top) and mass (bottom) spectra from photoenzymatically produced 4-methylbenzylamine (**82**).

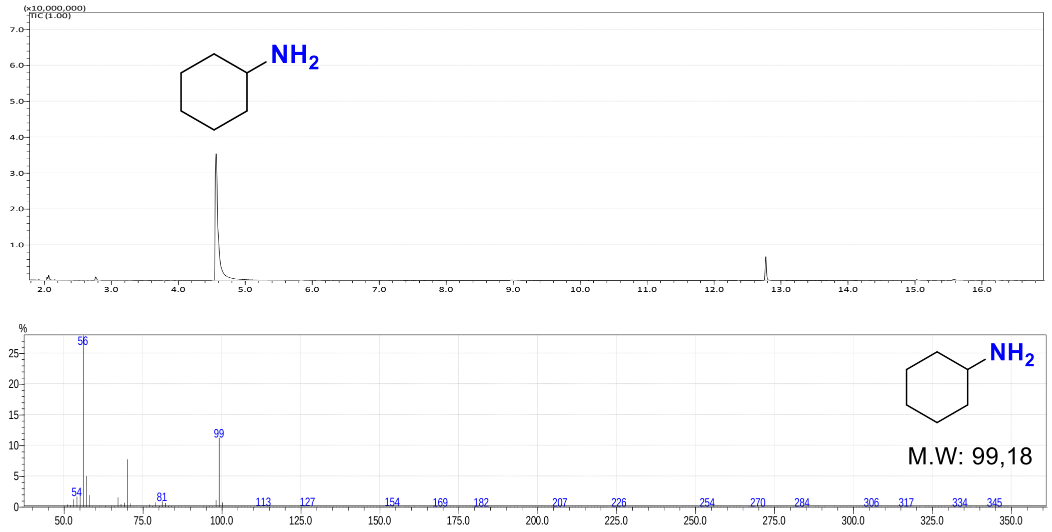

Supplementary Figure 60. Gas chromatography (top) and mass (bottom) spectra from photoenzymatically produced cyclohexylamine (**83**).

NMR Data

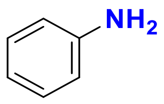

aniline (2)

84% yield (15.5 mg). ^{1}H-NMR (500 MHz, $DMSO_{d6}$): δ 7.09 – 6.83 (m, 2H), 6.55 – 6.50 (m, 2H), 6.49 – 6.43 (m, 1H), 4.98 (s, 2H); ^{13}C-NMR (126 MHz, $DMSO_{d6}$): δ 148.63, 128.81 (2C), 115.67, 113.90 (2C). The spectral data is in agreement with previously reported spectral data.[13,14]

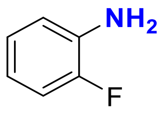

2-fluoroaniline (3)

94% yield (19.8 mg). ^{1}H-NMR (500 MHz, $DMSO_{d6}$): δ 6.99 – 6.91 (m, 1H), 6.89 – 6.82 (m, 1H), 6.79 – 6.71 (m, 1H), 6.53 – 6.45 (m, 1H), 5.07 (*brs*, 2H, NH_2). The spectral data is in agreement with previously reported spectral data.[15]

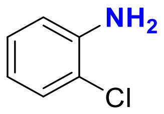

2-chloroaniline (4)

96% yield (22.6 mg). ^{1}H-NMR (500 MHz, $DMSO_{d6}$): δ 7.16 (dd, J = 8.0, 1.5 Hz, 1H), 7.03 – 6.98 (m, 1H), 6.77 (dd, J = 8.0, 1.6 Hz, 1H), 6.55 – 6.49 (m, 1H), 5.30 (*brs*, 2H, NH_2); ^{13}C-NMR (126 MHz, $DMSO_{d6}$): δ 145.13, 129.43, 128.12, 117.50, 117.22, 115.84. The spectral data is in agreement with previously reported spectral data, although slightly different as a result from the variance in the conditions employed.[16] Stacking and spiking NMR experiments of enzymatic product **4** with a commercial reference compound confirmed the structure of **4**.

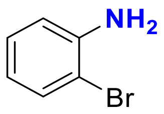

2-bromoaniline (5)

96% yield (29.1 mg). ^{1}H-NMR (500 MHz, $DMSO_{d6}$): δ 6.99 – 6.91 (m, 1H), 6.88 – 6.83 (m, 1H), 6.78 – 6.72 (m, 1H), 6.52 – 6.46 (m, 1H), 5.07 (*brs*, 2H, NH_2). The spectral data is in agreement with previously reported spectral data.[17]

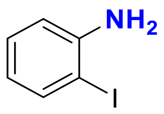

2-iodoaniline (6)

95% yield (35.4 mg). ^{1}H-NMR (500 MHz, $DMSO_{d6}$): δ 7.53 (dd, J = 7.9, 1.6 Hz, 1H), 7.10 – 7.03 (m, 1H), 6.75 (dd, J = 8.0, 1.7 Hz, 1H), 6.36 – 6.28 (m, 1H), 5.17 (*brs*, 2H, NH_2). The spectral data is in agreement with previously reported spectral data.[18]

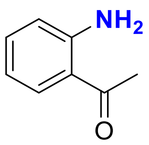

2-aminoacetophenone (7)

90% yield (22.3 mg). ^{1}H-NMR (500 MHz, $DMSO_{d6}$): δ 7.15 (d, J = 8.0 Hz, 1H), 7.15 – 7.07 (m, 2H), 6.82 – 6.77 (m, 1H), 5.33 (*brs*, 2H, NH_2), 2.48 (s, 3H). The spectral data is in agreement with previously reported spectral data.[16]

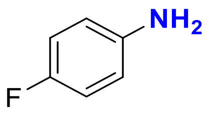

4-fluoroaniline (8)

94% yield (19.9 mg). ^{1}H-NMR (500 MHz, $DMSO_{d6}$): δ 6.87 – 6.79 (m, 2H), 6.56 – 6.49 (m, 2H), 4.91 (*brs*, 2H, NH_2). The spectral data is in agreement with previously reported spectral data.[16]

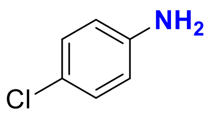

4-chloroaniline (9)

95% yield (22.4 mg). ^{1}H-NMR (500 MHz, DMSO$_{d6}$): δ 6.86 – 6.79 (m, 2H), 6.52 (dd, J = 9.0, 4.7 Hz, 1H), 5.20 (*brs*, 2H, NH_2); ^{13}C-NMR (126 MHz, DMSO$_{d6}$): δ 147.71, 128.50, 128.45, 118.65, 115.21, 115.09. The spectral data is in agreement with previously reported spectral data.[16]

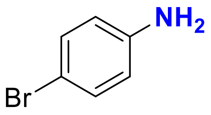

4-bromoaniline (10)

93% yield (28.2 mg). ^{1}H-NMR (500 MHz, DMSO$_{d6}$): δ 7.12 (d, J = 8.7 Hz, 2H), 6.51 (d, J = 8.8 Hz, 2H), 5.35 (*brs*, 2H, NH_2). The spectral data is in agreement with previously reported spectral data.[19]

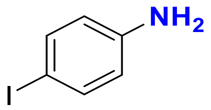

4-iodoaniline (11)

96% yield (35.8 mg). ^{1}H-NMR (500 MHz, DMSO$_{d6}$): δ 7.26 (d, J = 9 Hz, 2H), 6.40 (d, J = 9 Hz, 2H), 5.26 (*brs*, 2H, NH_2). The spectral data is in agreement with previously reported spectral data.[20]

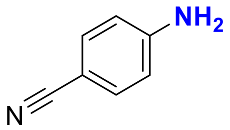

4-aminobenzonitrile (12)

97% yield (21.5 mg). ^{1}H-NMR (500 MHz, DMSO$_{d6}$): δ 7.38 (d, J = 9 Hz, 2H), 6.59 (d, J = 9 Hz, 2H), 6.15 (*brs*, 2H, NH_2). The spectral data is in agreement with previously reported spectral data.[21]

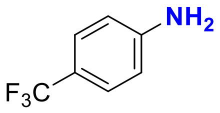

4-(trifluoromethyl)aniline (13)

96% yield (27.5 mg). ^{1}H-NMR (500 MHz, DMSO$_{d6}$): δ 7.00 (d, J = 9 Hz, 2H), 6.54 (d, J = 9 Hz, 2H), 5.21 (*brs*, 2H, NH_2). The spectral data is in agreement with previously reported spectral data.[22]

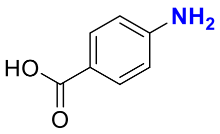

4-aminobenzoic acid (14)

95% yield (23 mg). ^{1}H-NMR (500 MHz, DMSO$_{d6}$): δ 11.94 (s, 1H), 7.61 (d, J = 8.7 Hz, 2H), 6.54 (d, J = 8.7 Hz, 2H), 5.86 (*brs*, 2H, NH_2). The spectral data is in agreement with previously reported spectral data.[23]

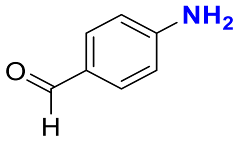

4-aminobenzaldehyde (15)

79% yield (17.9 mg). ^{1}H-NMR (500 MHz, Chloroform-*d*): δ 9.57 (s, 1H), 7.54 (d, J = 8.7 Hz, 2H), 6.62 (d, J = 8.5 Hz, 2H), 6.29 (*brs*, 2H, NH_2). The spectral data is in agreement with previously reported spectral data.[24]

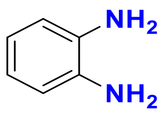

benzene-1,2-diamine (16)

83% yield (20.4 mg). ^{1}H-NMR (500 MHz, DMSO$_{d6}$): δ 6.50 – 6.45 (m, 2H), 6.40 – 6.33 (m, 2H), 4.38 (*brs*, 4H, 2 x NH_2). The spectral data is in agreement with previously reported spectral data.[16]

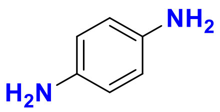

benzene-1,4-diamine (17)

94% yield (23.7 mg). ^{1}H-NMR (500 MHz, DMSO$_{d6}$): δ 6.34 (s, 4H), 4.20 (*brs*, 4H, 2 x NH_2). The spectral data is in agreement with previously reported spectral data.[25]

4-toluidine (18)

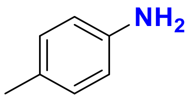

90% yield (18.5 mg). ^{1}H-NMR (500 MHz, $DMSO_{d6}$): δ 6.81 (d, J = 8.4 Hz, 2H), 6.46 (d, J = 8.4 Hz, 2H), 4.77 (*brs*, 2H, NH_2), 2.11 (s, 3H). The spectral data is in agreement with previously reported spectral data.[26]

4-methoxyaniline (19)

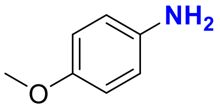

95% yield (21.8 mg). ^{1}H-NMR (500 MHz, $DMSO_{d6}$): δ 6.63 (d, J = 8.7 Hz, 2H), 6.50 (d, J = 8.7 Hz, 2H), 4.58 (*brs*, 2H, NH_2), 3.61 (s, 3H); ^{13}C-NMR (126 MHz, $DMSO_{d6}$): δ 151.10, 142.77 (2C), 115.38 (2C), 114.94, 55.71. The spectral data is in agreement with previously reported spectral data.[27]

4-aminophenol (20)

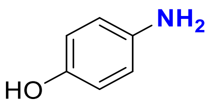

95% yield (19.8 mg). ^{1}H-NMR (500 MHz, $DMSO_{d6}$): δ 8.34 (*brs*, 1H, OH), 6.46 (d, J = 8.8 Hz, 2H), 6.40 (d, J = 8.7 Hz, 2H), 4.41 (*brs*, 2H, NH_2). The spectral data is in agreement with previously reported spectral data.[28]

2,3-dihydro-1H-inden-5-amine (21)

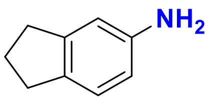

93% yield (22.7 mg). ^{1}H-NMR (500 MHz, $DMSO_{d6}$): δ 6.84 (d, J = 8.0 Hz, 1H), 6.45 – 6.38 (m, 1H), 6.33 (dd, J = 8.0, 2.3 Hz, 1H), 4.75 (*brs*, 2H, NH_2), 2.72 – 2.63 (m, 4H), 1.92 (p, J = 7.5 Hz, 2H). The spectral data is in agreement with previously reported spectral data.[29]

5-aminoindolin-2-one (22)

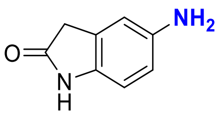

78% yield (20.8 mg). ^{1}H-NMR (500 MHz, $DMSO_{d6}$): δ 9.93 (s, 1H), 6.62 – 6.32 (m, 2H), 6.37 (dd, J = 8.1, 2.3 Hz, 1H), 4.67 (*brs*, 2H, NH_2), 3.31 (s, 2H). The spectral data is in agreement with previously reported spectral data.[30]

4-(pyridin-4-ylmethyl)aniline (23)

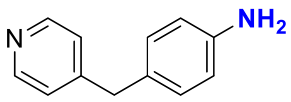

97% yield (31.1 mg). ^{1}H-NMR (500 MHz, $DMSO_{d6}$): δ 8.42 (d, J = 6.1 Hz, 2H), 7.18 (d, J = 6.1 Hz, 2H), 6.88 (d, J = 8.5 Hz, 2H), 6.50 (d, J = 8.5 Hz, 2H), 3.75 (s, 2H), 3.37 (*brs*, 2H, NH_2). The spectral data is in agreement with previously reported spectral data.[31]

benzene-1,2-diamine (24)

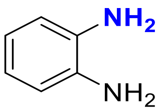

81% yield (16.8 mg). ^{1}H-NMR (500 MHz, $DMSO_{d6}$): δ 6.50 – 6.45 (m, 2H), 6.40 – 6.33 (m, 2H), 4.38 (*brs*, 4H, 2 x NH_2). The spectral data is in agreement with previously reported spectral data.[16]

5-fluorobenzene-1,2-diamine (25)

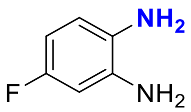

72% yield (16.8 mg). ^{1}H-NMR (500 MHz, $DMSO_{d6}$): δ 6.94 (dd, J = 8.4, 5.9 Hz, 1H), 6.81 (dd, J = 11.0, 2.9 Hz, 1H), 6.64 – 6.56 (m, 1H), 5.25 (s, 2H, NH_2), 3.84 (s, 2H, NH_2). The spectral data is in agreement with previously reported spectral data.[32]

5-bromobenzene-1,2-diamine (26)

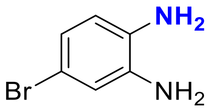

87% yield (28.3 mg). ^{1}H-NMR (500 MHz, $DMSO_{d6}$): δ 6.62 (d, J = 2.4 Hz, 1H), 6.48 – 6.45 (m, 1H), 6.41 (d, J = 8.4 Hz, 1H), 4.72 (*brs*, 2H, NH_2), 4.58 (*brs*, 2H, NH_2). The spectral data is in agreement with previously reported spectral data.[33]

4-fluorobenzene-1,2-diamine (27)

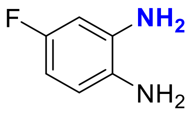

74% yield (17.3 mg). ^{1}H-NMR (500 MHz, $DMSO_{d6}$): δ 6.94 (dd, J = 8.4, 5.9 Hz, 1H), 6.81 (dd, J = 11.0, 2.9 Hz, 1H), 6.64 – 6.56 (m, 1H), 5.25 (s, 2H, NH_2), 3.84 (s, 2H, NH_2). The spectral data is in agreement with previously reported spectral data.[32]

4-bromobenzene-1,2-diamine (28)

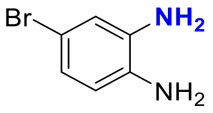

87% yield (28.3 mg). ^{1}H-NMR (500 MHz, $DMSO_{d6}$): δ 6.62 (d, J = 2.4 Hz, 1H), 6.48 – 6.45 (m, 1H), 6.41 (d, J = 8.4 Hz, 1H), 4.67 (4H, *brs*, 2 x NH_2). The spectral data is in agreement with previously reported spectral data.[33]

4-(trifluoromethyl)benzene-1,2-diamine (29)

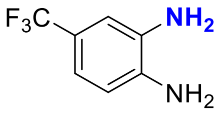

72% yield (22.2 mg). ^{1}H-NMR (500 MHz, $DMSO_{d6}$): δ 6.76 – 6.75 (m, 1H), 6.67 (d, J = 8.2 Hz, 1H), 6.56 (d, J = 8.0 Hz, 1H), 5.08 (*brs*, 2H, NH_2), 4.82 (*brs*, 2H, NH_2). The spectral data is in agreement with previously reported spectral data.[34]

3,4-diaminophenol (30)

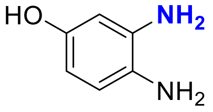

77% yield (17.8 mg). ^{1}H-NMR (500 MHz, $DMSO_{d6}$): δ 9.10 (s, 1H, OH), 7.37 (d, J = 8.4 Hz, 1H), 6.87 (d, J = 5.4 Hz, 1H), 6.67 (dd, J = 8.7, 2.4 Hz, 1H). The spectral data is in agreement with previously reported spectral data.[35]

benzene-1,4-diamine (31)

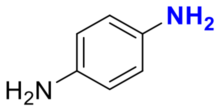

96% yield (19.8 mg). ^{1}H-NMR (500 MHz, $DMSO_{d6}$): δ 6.35 (s, 4H), 4.21 (*brs*, 4H, 2 x NH_2). The spectral data is in agreement with previously reported spectral data.[36]

3-aminopyridine (32)

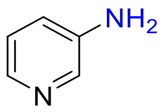

92% yield (17.1 mg). ^{1}H-NMR (500 MHz, $DMSO_{d6}$): δ 7.92 (dd, J = 2.8, 0.7 Hz, 1H), 7.72 (dd, J = 4.6, 1.4 Hz, 1H), 7.07 – 6.95 (m, 1H), 6.89 – 6.82 (m, 1H), 5.25 (s, 2H). The spectral data is in agreement with previously reported spectral data.[37]

2-fluoropyridin-3-amine (33)

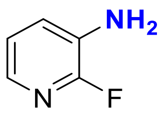

93% yield (19.8 mg). ^{1}H-NMR (500 MHz, $DMSO_{d6}$): δ 7.32 – 7.29 (m, 1H), 7.14 – 7.09 (m, 1H), 7.01 – 6.98 (m, 1H), 5.40 (*brs*, 2H, NH_2). The spectral data is in agreement with previously reported spectral data.[38]

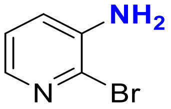

2-bromopyridin-3-amine (34)

91% yield (27.7 mg). ^{1}H-NMR (500 MHz, DMSO$_{d6}$): δ 7.56 (dd, J = 4.1, 2.0 Hz, 1H), 7.13 – 7.06 (m, 2H), 5.48 (*brs*, 2H, NH$_2$). The spectral data is in agreement with previously reported spectral data.[39]

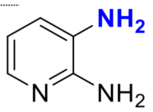

pyridine-2,3-diamine (35)

83% yield (17.3 mg). ^{1}H-NMR (500 MHz, DMSO$_{d6}$): δ 7.22 (dd, J = 5.0, 1.7 Hz, 1H), 6.63 (dd, J = 7.5, 1.7 Hz, 1H), 6.31 (dd, J = 7.4, 5.0 Hz, 1H), 5.26 (*brs*, 2H, NH$_2$), 4.58 (*brs*, 2H, NH$_2$). The spectral data is in agreement with previously reported spectral data.[40]

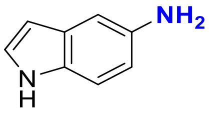

5-aminoindole (36)

91% yield (22.1 mg). ^{1}H-NMR (500 MHz, DMSO$_{d6}$): δ 10.57 (s, 1H), 7.11 (t, J = 2.7 Hz, 1H), 7.07 (d, J = 8.5 Hz, 1H), 6.67 (d, J = 2.0 Hz, 1H), 6.47 (dd, J = 8.4, 2.2 Hz, 1H), 6.12 – 6.10 (m, 1H), 4.48 (*brs*, 2H, NH$_2$). The spectral data is in agreement with previously reported spectral data.[16]

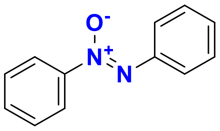

(*Z*)-1,2-diphenyldiazene 1-oxide (37)

97% yield (17.9 mg). ^{1}H-NMR (500 MHz, Chloroform-*d*): δ 8.39 – 8.26 (m, 2H), 8.19 – 8.02 (m, 2H), 7.74 – 7.45 (m, 5H), 7.43 – 7.30 (m, 1H); ^{13}C-NMR (126 MHz, Chloroform-*d*): δ 144.15, 131.75, 129.77 (2C), 128.96 (2C), 128.86 (2C), 125.68, 122.51, 122.51 (2C). The spectral data is in agreement with previously reported spectral data.[41,42]

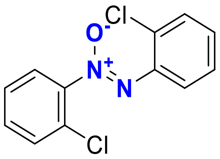

(*Z*)-1,2-bis(2-chlorophenyl)diazene 1-oxide (38)

81% yield (19.1 mg). ^{1}H-NMR (500 MHz, Chloroform-*d*): δ 8.00 (dd, J = 8.0, 1.9 Hz, 1H), 7.80 – 7.73 (m, 2H), 7.58 – 7.28 (m, 6H). The spectral data is in agreement with previously reported spectral data.[43]

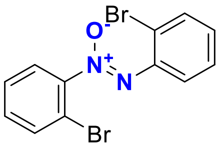

(*Z*)-1,2-bis(2-bromophenyl)diazene 1-oxide (39)

86% yield (26.1 mg). ^{1}H-NMR (500 MHz, DMSO$_{d6}$): δ 7.97 (dd, J = 8.0, 1.6 Hz, 1H), 7.79 – 7.70 (m, 3H), 7.52 – 7.36 (m, 3H), 7.25 – 7.21 (m, 1H). The spectral data is in agreement with previously reported spectral data.[44]

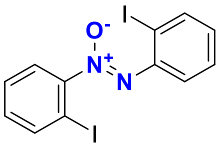

(*Z*)-1,2-bis(2-iodophenyl)diazene 1-oxide (40)

92% yield (34.3 mg). ^{1}H-NMR (500 MHz, Chloroform-*d*): δ 8.03 (d, J = 8.5 Hz, 1H), 7.92 (d, J = 8.8 Hz, 1H), 7.85 (d, J = 8.5 Hz, 1H), 7.81 (d, J = 8.7 Hz, 1H). The spectral data is in agreement with previously reported spectral data.[43]

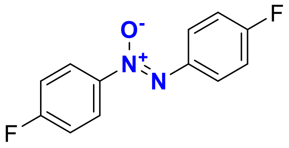

(*Z*)-1,2-bis(4-fluorophenyl)diazene 1-oxide (41)

80% yield (16.9 mg). ^{1}H-NMR (500 MHz, Chloroform-*d*): δ 8.37 – 8.30 (m, 2H), 8.29 – 8.23 (m, 2H), 7.23 – 7.13 (m, 4H). The spectral data is in agreement with previously reported spectral data.[45]

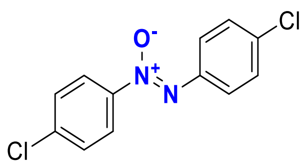

(*Z*)-1,2-bis(4-chlorophenyl)diazene 1-oxide (42)

84% yield (19.8 mg). ^{1}H-NMR (500 MHz, Chloroform-*d*): δ 8.25 (d, J = 8.8 Hz, 2H), 8.16 (d, J = 8.8 Hz, 2H), 7.59 – 7.38 (m, 4H); ^{13}C-NMR (126 MHz, Chloroform-*d*): δ 129.55 (2C), 129.19 (2C), 129.12 (2C), 127.22 (2C), 124.34 (2C), 123.86 (2C). The spectral data is in agreement with previously reported spectral data.[43]

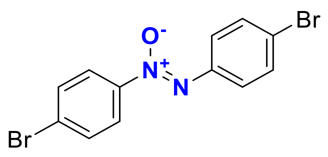

(*Z*)-1,2-bis(4-bromophenyl)diazene 1-oxide (43)

91% yield (27.5 mg). ^{1}H-NMR (500 MHz, Chloroform-*d*): δ 8.18 (d, J = 9.0 Hz, 2H), 8.08 (d, J = 8.8 Hz, 2H), 7.65 (d, J = 9.0 Hz, 2H), 7.61 (d, J = 9 Hz, 2H). The spectral data is in agreement with previously reported spectral data.[46]

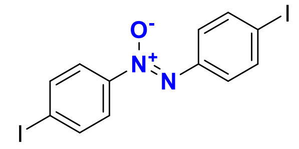

(*Z*)-1,2-bis(4-iodophenyl)diazene 1-oxide (44)

94% yield (35.1 mg). ^{1}H-NMR (500 MHz, Chloroform-*d*): δ 8.03 (d, J = 9.0 Hz, 2H), 7.92 (d, J = 9.0 Hz, 2H), 7.86 (d, J = 9.0 Hz, 2H), 7.81 (d, J = 9.0 Hz, 2H). The spectral data is in agreement with previously reported spectral data.[46]

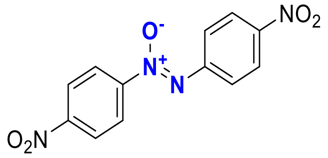

(*Z*)-1,2-bis(4-nitrophenyl)diazene 1-oxide (45)

84% yield (21.2 mg). ^{1}H-NMR (500 MHz, Chloroform-*d*): δ 8.54 (d, J = 9.0 Hz, 2H), 8.43 (d, J = 9.0 Hz, 2H), 8.37 (d, J = 9.0 Hz, 2H), 8.30 (d, J = 9.0 Hz, 2H). The spectral data is in agreement with previously reported spectral data.[36]

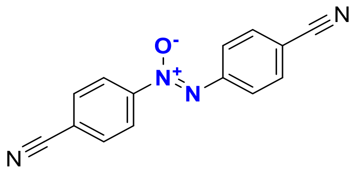

(*Z*)-1,2-bis(4-cyanophenyl)diazene 1-oxide (46)

96% yield (21.3 mg). ^{1}H-NMR (500 MHz, Chloroform-*d*): δ 8.46 (d, J = 9.0 Hz, 2H), 8.23 (d, J = 9.0 Hz, 2H), 7.87 (d, J = 9.0 Hz, 2H), 7.79 (d, J = 9.0 Hz, 2H). The spectral data is in agreement with previously reported spectral data.[46]

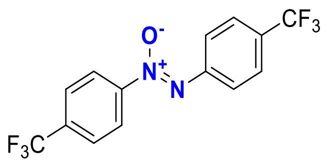

(*Z*)-1,2-bis(4-(trifluoromethyl)phenyl)diazene 1-oxide (47)

91% yield (26.1 mg). ^{1}H-NMR (500 MHz, Chloroform-*d*): δ 8.46 (d, J = 9.0 Hz, 2H), 8.24 (d, J = 8.7 Hz, 2H), 7.79 (dd, J = 21.2, 8.3 Hz, 4H). The spectral data is in agreement with previously reported spectral data. [47]

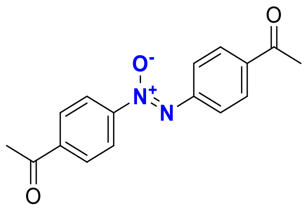

(*Z*)-1,2-bis(4-acetylphenyl)diazene 1-oxide (48)

93% yield (23.1 mg). ^{1}H-NMR (500 MHz, Chloroform-*d*): δ 8.42 (d, J = 8.8 Hz, 2H), 8.22 (d, J = 8.7 Hz, 2H), 8.15 – 8.05 (m, 4H), 2.69 (s, 3H), 2.66 (s, 3H). The spectral data is in agreement with previously reported spectral data.[46]

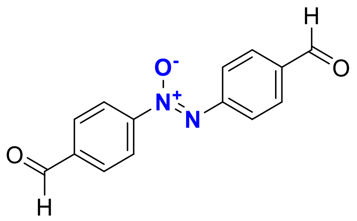

(*Z*)-1,2-bis(4-formylphenyl)diazene 1-oxide (49)

92% yield (20.8 mg). ^{1}H-NMR (500 MHz, Chloroform-*d*): δ 10.16 (s, 1H), 10.07 (s, 1H), 8.51 (d, J = 8.7 Hz, 2H), 8.28 (d, J = 8.7 Hz, 2H), 8.07 (d, J = 8.7 Hz, 2H), 8.03 (d, J = 8.7 Hz, 2H). The spectral data is in agreement with previously reported spectral data.[48]

(*Z*)-1,2-di-p-tolyldiazene 1-oxide (50)

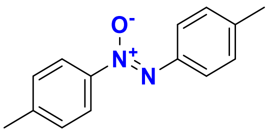

94% yield (19.3 mg). ^{1}H-NMR (500 MHz, Chloroform-*d*): *δ* 8.18 (d, *J* = 8.7 Hz, 2H), 8.11 (d, *J* = 8.5 Hz, 2H), 7.33 – 7.20 (m, 4H), 2.44 (s, 3H), 2.41 (s, 3H). The spectral data is in agreement with previously reported spectral data.[46]

(*Z*)-1,2-bis(4-methoxyphenyl)diazene 1-oxide (51)

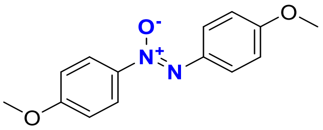

87% yield (19.5 mg). ^{1}H-NMR (500 MHz, Chloroform-*d*) *δ* 8.30 – 8.22 (m, 4H), 7.05 – 6.92 (m, 4H), 3.89 (s, 3H), 3.88 (s, 3H); ^{13}C-NMR (126 MHz, Chloroform-*d*): *δ* 161.99, 160.33, 141.87, 138.14 (2C), 127.95 (2C), 123.92, 113.87 (2C), 113.74 (2C), 55.83, 55.64. The spectral data is in agreement with previously reported spectral data.[41]

(*Z*)-1,2-di(pyridin-3-yl)diazene 1-oxide (52)

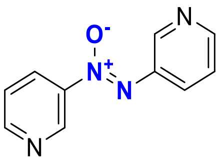

77% yield (14.3 mg). ^{1}H-NMR (500 MHz, Chloroform-*d*): *δ* 9.57 (d, *J* = 4.0 Hz, 1H), 9.26 (d, *J* = 4.0 Hz, 1H), 8.83 (d, *J* = 4.0 Hz, 1H), 8.63 (s, 1H), 8.63 – 8.57 (m, 2H), 7.51 (dd, *J* = 8.2, 4.6 Hz, 1H), 7.47 (dd, *J* = 8.0, 4.0 Hz, 1H). The spectral data is in agreement with previously reported spectral data.[49]

(*E*)-1,2-diphenyldiazene (53)

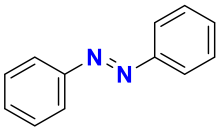

92% yield (16.9 mg). ^{1}H-NMR (500 MHz, Chloroform-*d*): *δ* 7.98 – 7.80 (m, 4H), 7.56 – 7.45 (m, 4H). ^{13}C-NMR (126 MHz, Chloroform-*d*): *δ* 152.78 (2C), 131.14 (2C), 129.23 (4C), 122.99 (4C); HRMS (ESI^{+}): calculated for $C_{12}H_{11}N_2$ $[M+H]^{+}$: 183.0912, found: 183.0911. The spectral data is in agreement with previously reported spectral data.[50]

(*E*)-1,2-bis(2-chlorophenyl)diazene (54)

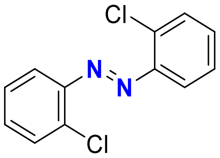

77% yield (18.1 mg). ^{1}H-NMR (500 MHz, Chloroform-*d*): *δ* 7.78 (dd, *J* = 8.0, 1.7 Hz, 2H), 7.57 (dd, *J* = 8.0, 1.6 Hz, 2H), 7.45 – 7.34 (m, 4H). HRMS (ESI^{+}): calculated for $C_{12}H_9N_2Cl_2$ $[M+H]^{+}$: 251.0135, found: 251.0134. The spectral data is in agreement with previously reported spectral data, although slightly different as a result from the variance in the conditions employed.[43]

(*E*)-1,2-bis(2-bromophenyl)diazene (55)

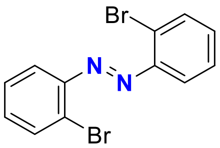

81% yield (24.5 mg). ^{1}H-NMR (500 MHz, Chloroform-*d*): *δ* 7.44 – 7.39 (m, 2H), 7.38 – 7.32 (m, 2H), 7.10 – 7.00 (m, 4H). HRMS (ESI^{+}): calculated for $C_{12}H_9N_2Br_2$ $[M+H]^{+}$: 338.9126, found: 338.9125. The spectral data is in agreement with previously reported spectral data.[51]

(*E*)-1,2-bis(2-iodophenyl)diazene (56)

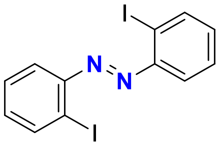

83% yield (30.9 mg). ^{1}H-NMR (500 MHz, Chloroform-*d*): *δ* 8.04 (d, *J* = 7.9 Hz, 2H), 7.77 (d, *J* = 8.0 Hz, 2H), 7.46 (t, *J* = 7.6 Hz, 2H), 7.20 (t, *J* = 7.6 Hz, 2H). HRMS (ESI^{+}): calculated for $C_{12}H_9N_2I_2$

$[M+H]^+$: 434.8852, found: 434.8841. The spectral data is in agreement with previously reported spectral data.[43]

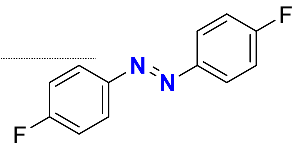

(*E*)-1,2-bis(4-fluorophenyl)diazene (57)

83% yield (17.5 mg). ^{1}H-NMR (500 MHz, Chloroform-*d*): δ 7.96 – 7.88 (m, 4H), 7.22 – 7.16 (m, 4H). HRMS (ESI$^+$): calculated for $C_{12}H_9N_2F_2$ $[M+H]^+$: 219.0721, found: 219.0720. The spectral data is in agreement with previously reported spectral data.[45]

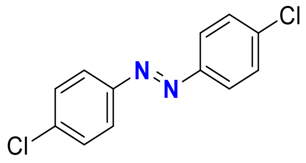

(*E*)-1,2-bis(4-chlorophenyl)diazene (58)

86% yield (20.2 mg). ^{1}H-NMR (500 MHz, Chloroform-*d*): δ 7.87 (d, J = 8.8 Hz, 2H), 7.49 (d, J = 8.7 Hz, 2H). HRMS (ESI$^+$): calculated for $C_{12}H_9N_2Cl_2$ $[M+H]^+$: 251.0138, found: 251.0137. The spectral data is in agreement with previously reported spectral data.[46]

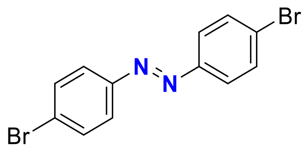

(*E*)-1,2-bis(4-bromophenyl)diazene (59)

88% yield (26.5 mg). ^{1}H-NMR (500 MHz, Chloroform-*d*): δ 7.79 (d, J = 9.0 Hz, 4H), 7.65 (d, J = 9.0 Hz, 4H); ^{13}C-NMR (126 MHz, Chloroform-d): δ 132.56 (4C), 132.30 (2C), 125.92 (4C), 124.57 (2C). HRMS (ESI$^+$): calculated for $C_{12}H_9N_2Br_2$ $[M+H]^+$: 338.9128, found: 338.9127. The spectral data is in agreement with previously reported spectral data.[46]

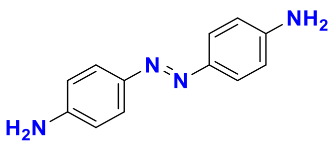

(*E*)-4,4'-(diazene-1,2-diyl)dianiline (60)

70% yield (17.6 mg). ^{1}H-NMR (500 MHz, DMSO$_{d6}$): δ 7.52 (d, J = 8.8 Hz, 4H), 6.62 (d, J = 8.8 Hz, 4H), 5.74 (*brs*, 4H, 2 x NH_2); ^{13}C-NMR (126 MHz, DMSO$_{d6}$): δ 151.45 (4C), 143.58 (4C), 124.29 (2C), 113.91 (2C). HRMS (ESI$^+$): calculated for $C_{12}H_{13}N_4$ $[M+H]^+$: 213.1101, found: 213.1100. The ^{1}H-NMR spectrum is in agreement with previously reported spectral data.[52]

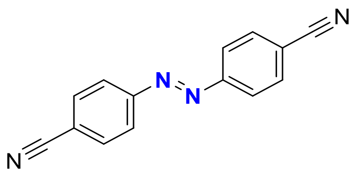

(*E*)-1,2-bis(4-cyanophenyl)diazene (61)

62% yield (13.7 mg). ^{1}H-NMR (500 MHz, Chloroform-*d*): δ 8.04 (d, J = 8.7 Hz, 4H), 7.86 (d, J = 8.5 Hz, 4H). HRMS (ESI$^+$): calculated for $C_{14}H_9N_4$ $[M+H]^+$: 233.0815, found: 233.0814. The spectral data is in agreement with previously reported spectral data.[46]

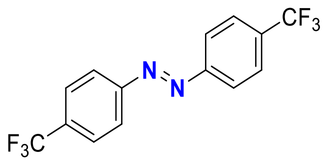

(*E*)-1,2-bis(4-(trifluoromethyl)phenyl)diazene (62)

77% yield (22.08 mg). ^{1}H-NMR (500 MHz, Chloroform-*d*): δ 8.04 (d, J = 8.2 Hz, 4H), 7.81 (d, J = 8.5 Hz, 4H). HRMS (ESI$^+$): calculated for $C_{14}H_9F_6N_2$ $[M+H]^+$: 319.0623, found: 319.0622. The spectral data is in agreement with previously reported spectral data.[47]

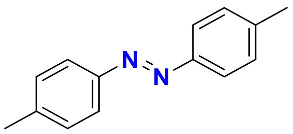

(*E*)-1,2-di-p-tolyldiazene (63)

91% yield (18.6 mg). ^{1}H-NMR (500 MHz, Chloroform-*d*): δ 7.81 (d, J = 8.4 Hz, 4H), 7.31 (d, J = 8.4 Hz, 4H), 2.43 (s, 6H); ^{13}C-NMR (126 MHz, Chloroform-*d*): δ 150.97 (2C), 141.35 (2C), 129.85 (4C), 122.86 (4C), 21.62 (2C). HRMS (ESI$^+$): calculated for $C_{14}H_{15}N_2$

$[M+H]^+$: 211.1232, found: 211.1231. The spectral data is in agreement with previously reported spectral data.[46]

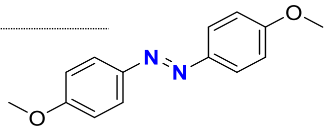

(*E*)-1,2-bis(4-methoxyphenyl)diazene (64)

70% yield (15.6 mg). ^{1}H-NMR (500 MHz, Chloroform-*d*): δ 7.88 (d, *J* = 9.0 Hz, 4H), 7.00 (d, *J* = 9.0 Hz, 4H), 3.89 (s, 6H). HRMS (ESI^+): calculated for $C_{14}H_{15}O_2N_2$ $[M+H]^+$: 243.1122, found: 243.1121. The spectral data is in agreement with previously reported spectral data.[53]

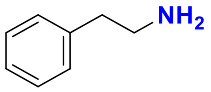

phenethylamine (68)

77% yield (17.2 mg). ^{1}H-NMR (500 MHz, DMSO$_{d6}$): δ 7.36 – 7.25 (m, 2H), 7.24 – 7.17 (m, 3H), 5.08 (*brs*, 2H, NH_2), 2.83 (t, *J* = 6.8 Hz, 2H), 2.71 (t, *J* = 6.8 Hz, 2H); ^{13}C-NMR (126 MHz, DMSO$_{d6}$): δ 140.47, 128.65 (2C), 128.22 (2C), 125.77, 43.77 (2C). HRMS (ESI^+): calculated for $C_8H_{11}N$ $[M+H]^+$: 122.0915, found: 122.0916. The spectral data is in agreement with previously reported spectral data.[54]

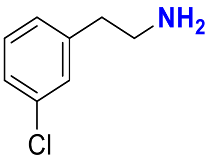

3-chlorophenethylamine (69)

74% yield (20.4 mg). ^{1}H-NMR (500 MHz, DMSO$_{d6}$): δ 7.34 – 7.25 (m, 3H), 7.20 – 7.16 (m, 1H), 2.81 (t, *J* = 7.5 Hz, 2H), 2.69 (t, *J* = 7.3 Hz, 2H); ^{13}C-NMR (126 MHz, DMSO$_{d6}$): δ 143.66, 133.27, 130.47, 129.00, 127.92, 126.21, 43.82 (2C). HRMS (ESI^+): calculated for $C_8H_{10}NCl$ $[M+H]^+$: 157.0445, found: 157.0444. The spectral data is in agreement with previously reported spectral data.[55]

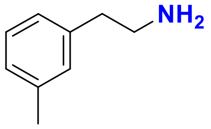

3-methylphenethylamine (70)

79% yield (19.3 mg). ^{1}H-NMR (500 MHz, DMSO$_{d6}$): δ 7.23 – 7.13 (m, 1H), 7.04 – 6.95 (m, 3H), 2.79 (t, *J* = 7.6 Hz, 2H), 2.64 (t, *J* = 7.6 Hz, 2H), 2.27 (s, 3H). HRMS (ESI^+): calculated for $C_9H_{13}N$ $[M+H]^+$: 136.1005, found: 136.1006. The spectral data is in agreement with previously reported spectral data.[56]

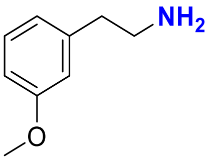

3-methoxyphenethylamine (71)

77% yield (20.7 mg). ^{1}H-NMR (500 MHz, DMSO$_{d6}$): δ 7.23 – 7.10 (m, 1H), 6.80 – 6.70 (m, 3H), 4.09 (*brs*, 2H, NH_2), 3.73 (s, 3H), 2.79 (t, *J* = 7.3 Hz, 2H), 2.64 (t, *J* = 7.4 Hz, 2H). HRMS (ESI^+): calculated for $C_8H_{13}NO$ $[M+H]^+$: 152.1089, found: 152.1090. The spectral data is in agreement with previously reported spectral data.[57]

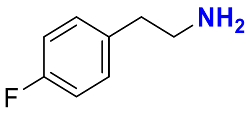

4-fluorophenethylamine (72)

73% yield (18.3 mg). ^{1}H-NMR (500 MHz, DMSO$_{d6}$): δ 7.29 – 7.22 (m, 2H), 7.15 – 7.08 (m, 2H), 2.80 (t, *J* = 7.3 Hz, 2H), 2.68 (t, *J* = 7.4 Hz, 2H). HRMS (ESI^+): calculated for $C_8H_{10}NF$ $[M+H]^+$: 140.0887, found: 140.0886. The spectral data is in agreement with previously reported spectral data.[58]

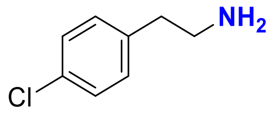

4-chlorophenethylamine (73)

90% yield (24.8 mg). ^{1}H-NMR (500 MHz, DMSO$_{d6}$): δ 7.38 – 7.35 (m, 2H), 7.29 – 7.25 (m, 2H), 2.82 (t, J = 7.4 Hz, 2H), 2.70 (t, J =b 7.3 Hz, 2H); ^{13}C-NMR (126 MHz, DMSO$_{d6}$): δ 140.00, 131.01 (2C), 130.83, 128.56 (2C), 43.97, 43.95. HRMS (ESI$^+$): calculated for $C_8H_{10}NCl$ $[M+H]^+$: 157.0445, found: 157.0444. The spectral data is in agreement with previously reported spectral data.[59]

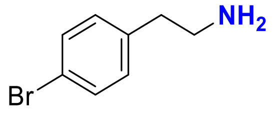

4-bromophenethylamine (74)

72% yield (24.6 mg). ^{1}H-NMR (500 MHz, DMSO$_{d6}$): δ 7.50 – 7.45 (m, 2H), 7.22 – 7.17 (m, 2H), 2.80 (t, J = 7.3 Hz, 2H), 2.67 (t, J = 7.3 Hz, 2H). HRMS (ESI$^+$): calculated for $C_8H_{10}NBr$ $[M+H]^+$: 200.9977, found: 200.9976. The spectral data is in agreement with previously reported spectral data.[60]

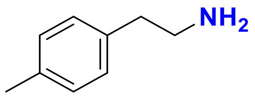

4-methylphenethylamine (75)

78% yield (19.1 mg). ^{1}H-NMR (500 MHz, DMSO$_{d6}$): δ 7.08 (s, 4H), 2.77 (t, J = 7.5 Hz, 2H), 2.63 (t, J = 7.5 Hz, 2H), 2.26 (s, 3H). HRMS (ESI$^+$): calculated for $C_9H_{13}N$ $[M+H]^+$: 136.1005, found: 136.1006. The spectral data is in agreement with previously reported spectral data.[61]

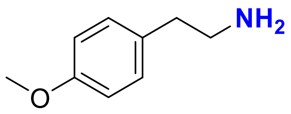

4-methoxyphenethylamine (76)

80% yield (21.5 mg). ^{1}H-NMR (500 MHz, DMSO$_{d6}$): δ 7.11 (d, J = 8.7 Hz, 2H), 6.84 (d, J = 8.7 Hz, 2H), 4.40 (s, 2H), 3.71 (s, 3H), 2.75 (t, J = 7.3 Hz, 2H), 2.60 (t, J = 7.4 Hz, 2H). HRMS (ESI$^+$): calculated for $C_8H_{13}NO$ $[M+H]^+$: 152.1089, found: 152.1087.

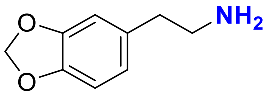

1,3-benzodioxole-5-ethanamine (77)

83% yield (24.1 mg). ^{1}H-NMR (500 MHz, DMSO$_{d6}$): δ 7.98 (*brs*, 2H, NH_2), 6.92 – 6.85 (m, 2H), 6.72 (dd, J = 7.9, 1.9 Hz, 1H), 6.00 (s, 2H), 2.99 (t, J = 7.2 Hz, 2H), 2.80 (t, J = 7.8 Hz, 2H); ^{13}C-NMR (126 MHz, DMSO$_{d6}$): δ 147.39, 145.99, 131.04, 121.70, 109.14, 108.28, 100.84, 32.61 (2C). HRMS (ESI$^+$): calculated for $C_9H_{11}NO_2$ $[M+H]^+$: 166.0832, found: 166.0831. The spectral data is in agreement with previously reported spectral data.[62]

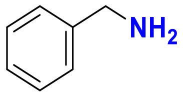

benzylamine (78)

73% yield (15.0 mg). ^{1}H-NMR (500 MHz, DMSO$_{d6}$): δ 7.74 – 6.87 (m, 5H), 3.74 (s, 2H); ^{13}C-NMR (126 MHz, DMSO$_{d6}$): δ 144.79, 128.51 (2C), 127.45 (2C), 126.58, 46.15. HRMS (ESI$^+$): calculated for C_7H_9N $[M+H]^+$: 108.0788, found: 108.0789. The spectral data is in agreement with previously reported spectral data.[63]

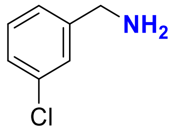

3-chlorobenzylamine (79)

77% yield (19.8 mg). ^{1}H-NMR (500 MHz, DMSO$_{d6}$): δ 7.48 – 7.40 (m, 1H), 7.36 – 7.20 (m, 3H), 3.73 (s, 2H); ^{13}C-NMR (126 MHz, DMSO$_{d6}$): δ 147.13, 132.85, 129.87, 126.83, 125.99, 125.64, 45.07. HRMS (ESI$^+$): calculated for C_7H_8NCl $[M+H]^+$: 143.0136, found:

143.0135. The spectral data is in agreement with previously reported spectral data.[64]

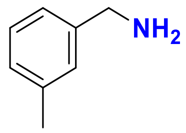

3-methylbenzylamine (80)

75% yield (17.0 mg). ^{1}H-NMR (500 MHz, DMSO$_{d6}$): δ 7.21 – 7.08 (m, 3H), 7.05 – 7.01 (m, 1H), 3.71 (s, 2H), 2.29 (s, 3H). HRMS (ESI$^+$): calculated for $C_8H_{11}N$ [M+H]$^+$: 122.0995, found: 122.0996. The spectral data is in agreement with previously reported spectral data.[65]

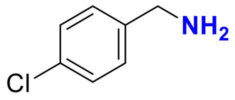

4-chlorobenzylamine (81)

80% yield (20.6 mg). ^{1}H-NMR (500 MHz, DMSO$_{d6}$): δ 7.36 (s, 4H), 3.71 (s, 2H); ^{13}C-NMR (126 MHz, DMSO$_{d6}$): δ 143.84, 131.01, 129.31 (2C), 128.40 (2C), 45.34. HRMS (ESI$^+$): calculated for C_7H_8NCl [M+H]$^+$: 143.0136, found: 143.0137. The spectral data is in agreement with previously reported spectral data.[66]

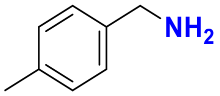

4-methylbenzylamine (82)

79% yield (17.9 mg). ^{1}H-NMR (500 MHz, DMSO$_{d6}$): δ 7.21 (d, J = 8.0 Hz, 2H), 7.11 (d, J = 11.3 Hz, 2H), 4.31 (*brs*, 2H, NH_2), 3.68 (s, 2H), 2.27 (s, 3H). HRMS (ESI$^+$): calculated for $C_8H_{11}N$ [M+H]$^+$: 122.0995, found: 122.0997. The spectral data is in agreement with previously reported spectral data.[67]

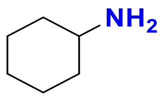

cyclohexylamine (83)

85% yield (16.2 mg). ^{1}H-NMR (500 MHz, DMSO$_{d6}$): δ 2.57 – 2.42 (m, 1H), 1.71 (dd, J = 8.3, 4.2 Hz, 2H), 1.67 – 1.58 (m, 2H), 1.59 – 1.48 (m, 1H), 1.28 – 1.13 (m, 2H), 1.13 – 1.04 (m, 1H), 1.02 – 0.85 (m, 2H). HRMS (ESI$^+$): calculated for $C_6H_{13}N$ [M+H]$^+$: 100.1077, found: 100.1078. The spectral data is in agreement with previously reported spectral data.[68]

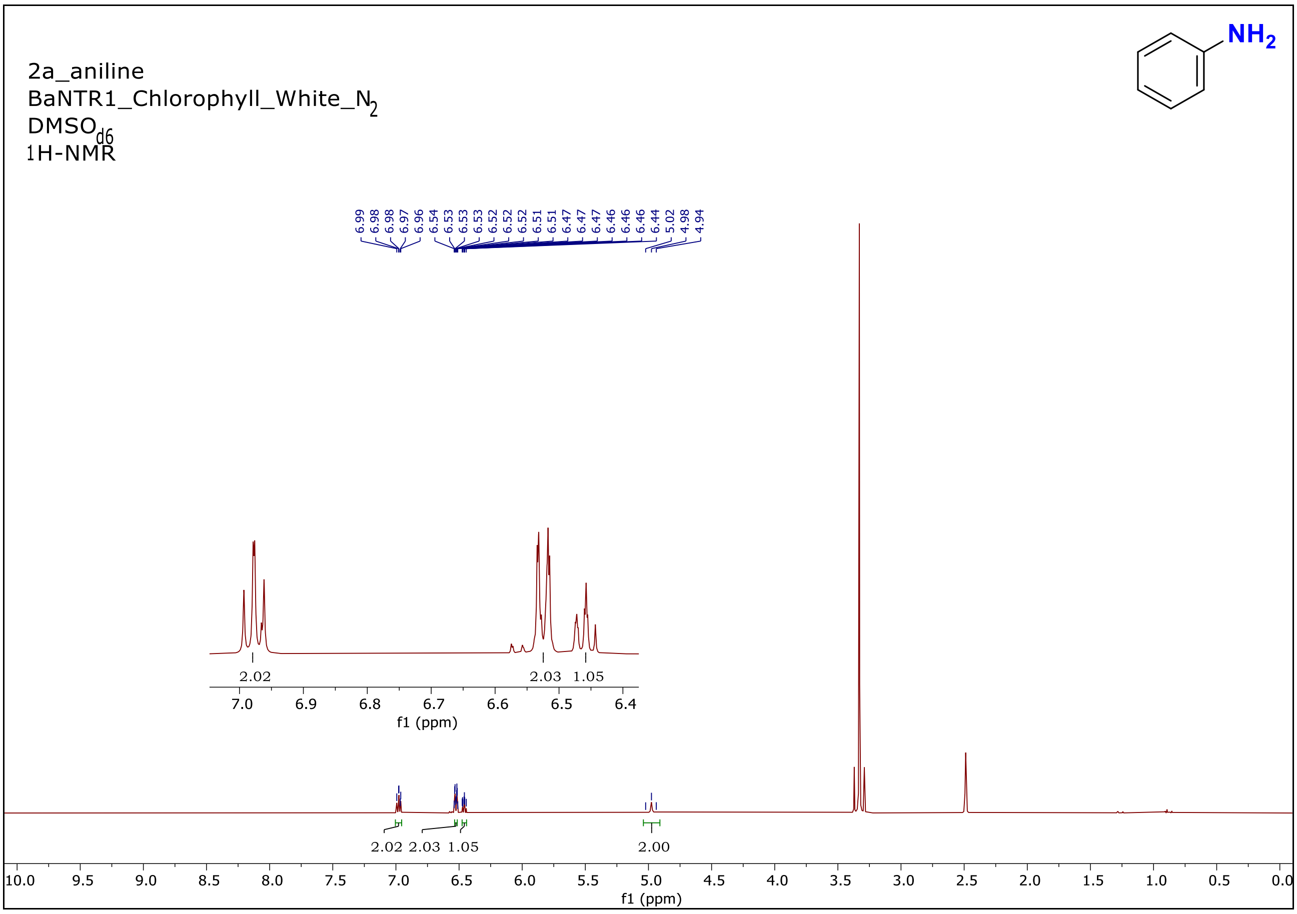

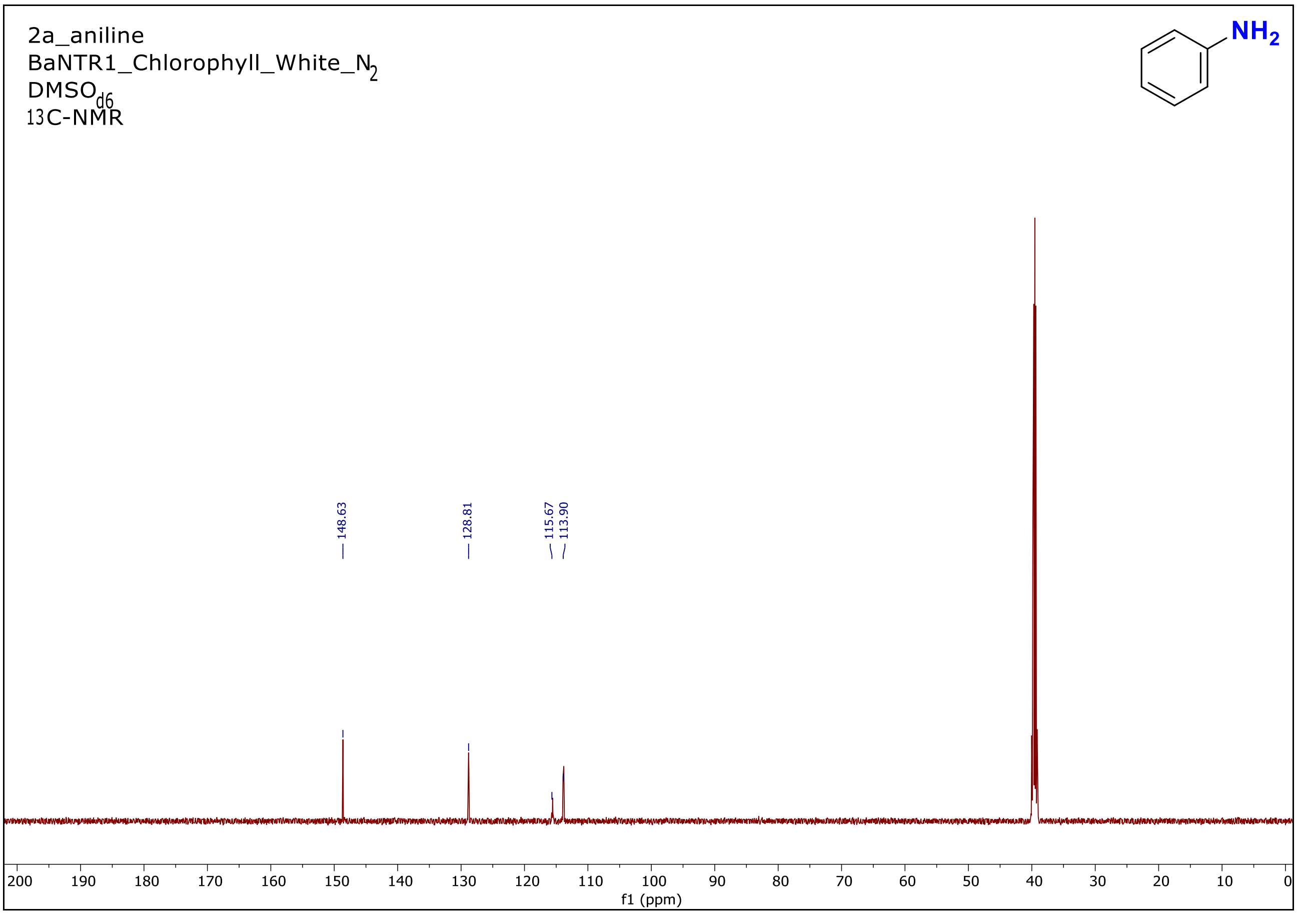

Supplementary Figure 61. ^{1}H-NMR (top) and ^{13}C-NMR (bottom) spectrum of the photoenzymatic product **2**.

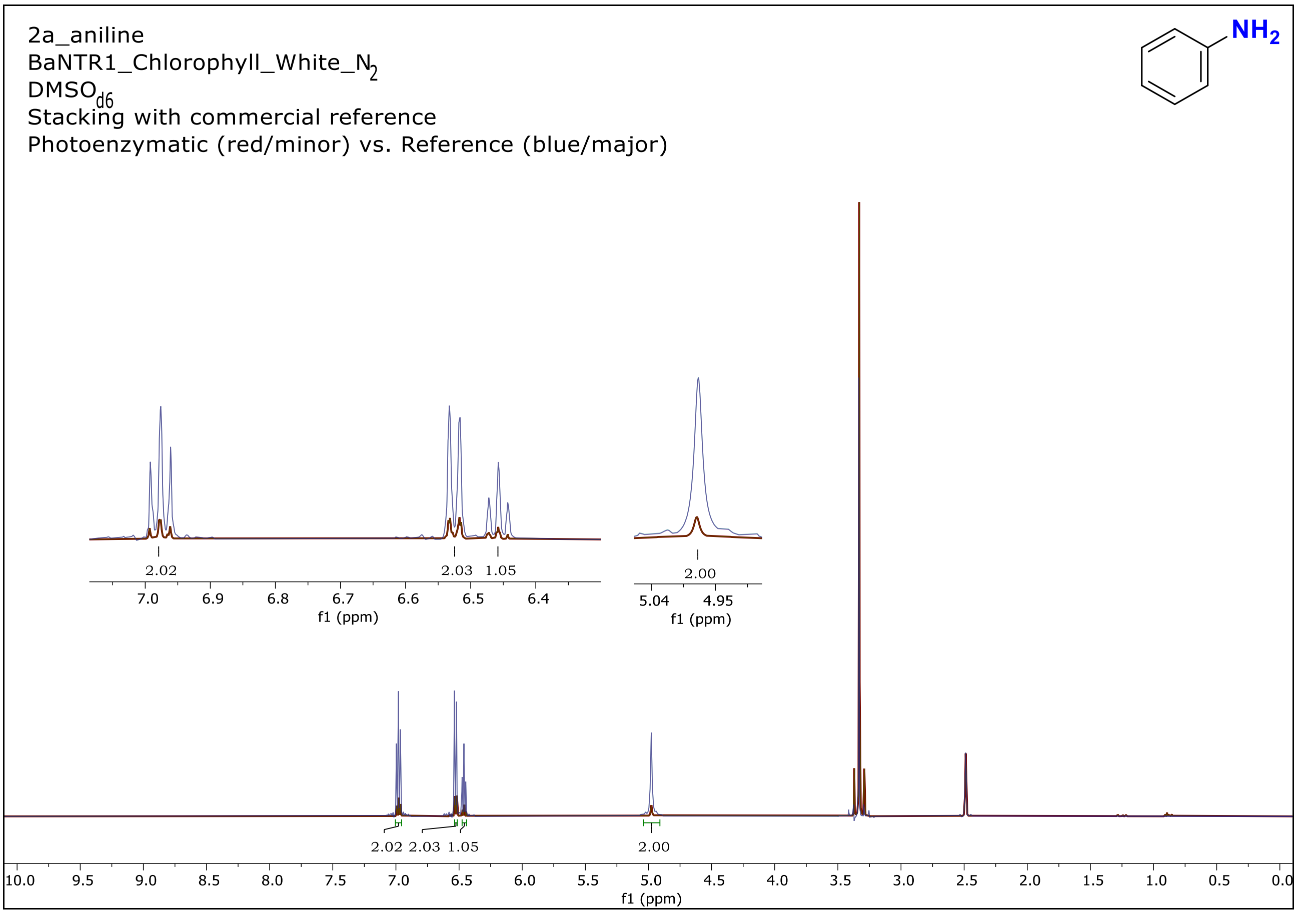

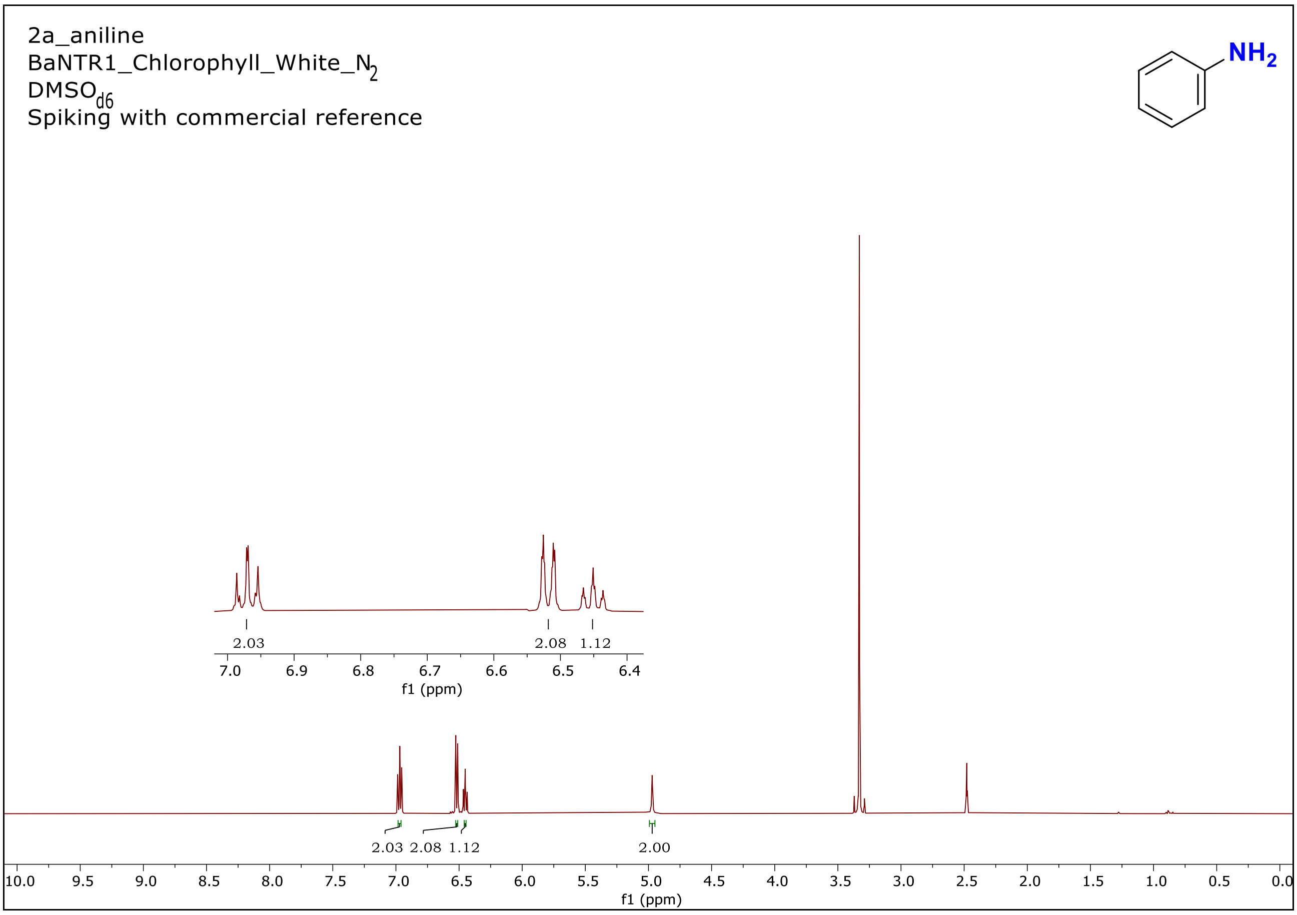

Supplementary Figure 62. Stacking (top) and spiking (bottom) ^{1}H-NMR spectrum of the photoenzymatic product **2** with a commercial reference.

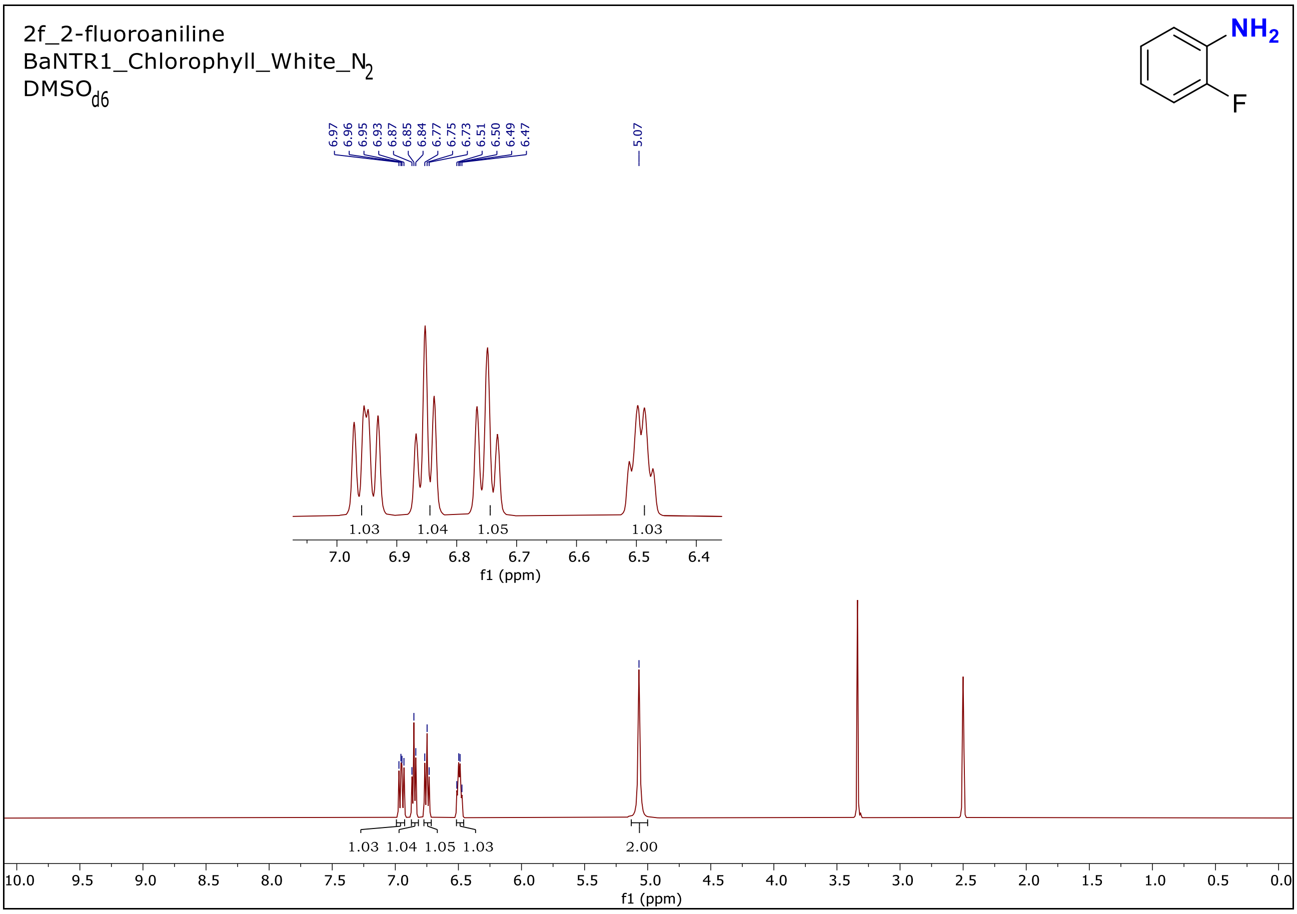

Supplementary Figure 63. ^{1}H-NMR spectrum of the photoenzymatic product **3**.

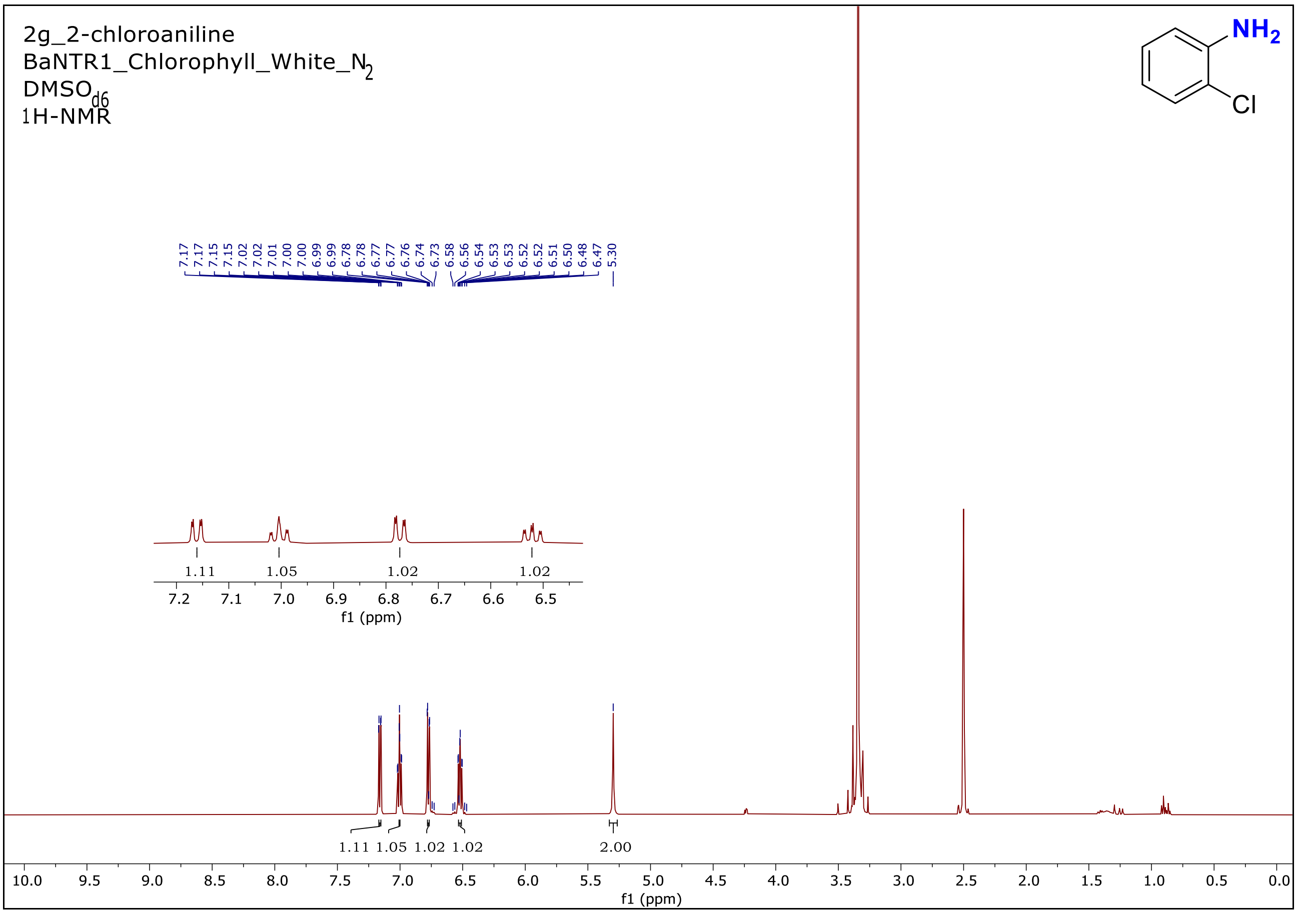

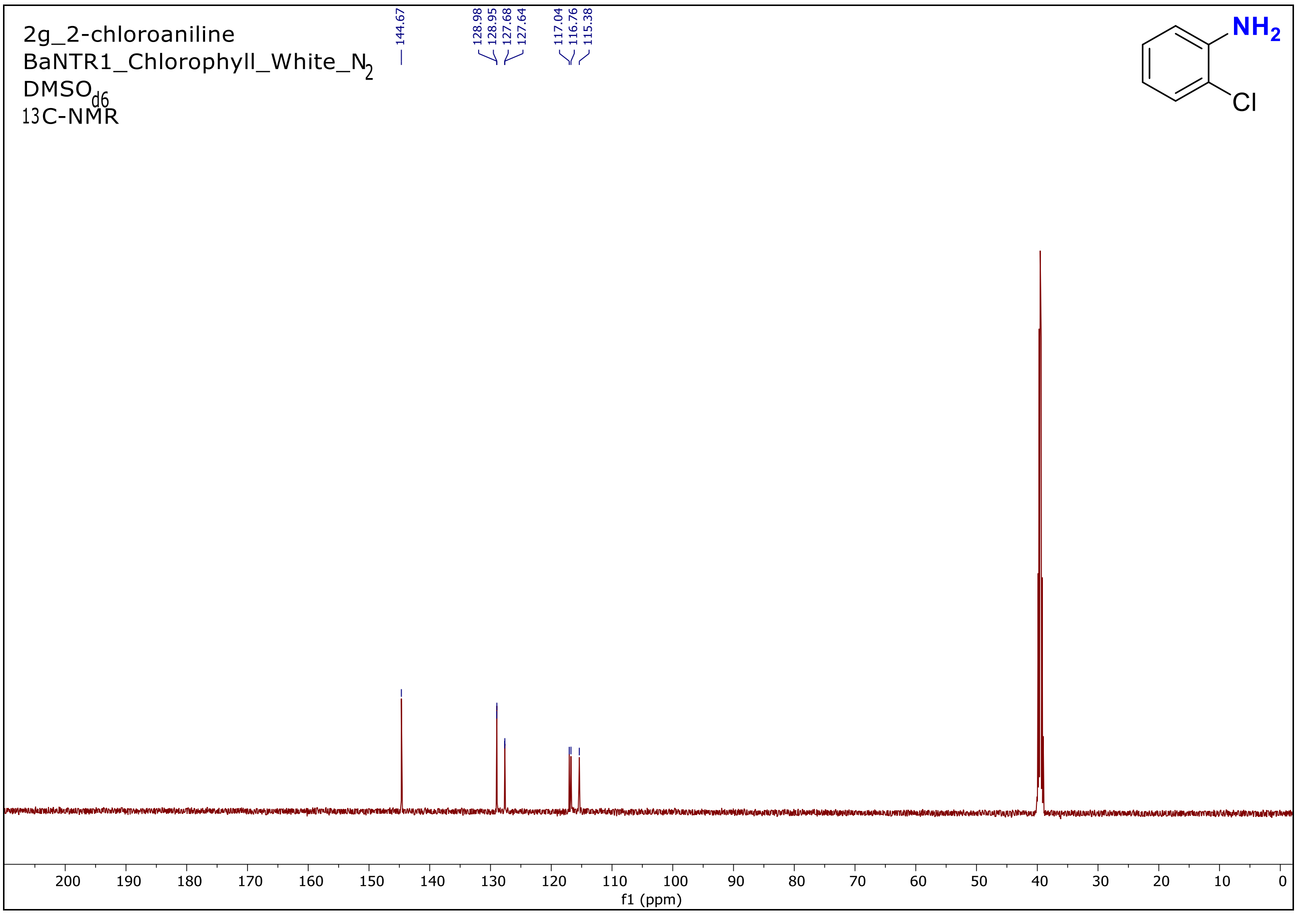

Supplementary Figure 64. ^{1}H-NMR (top) and ^{13}C-NMR (bottom) spectrum of the photoenzymatic product **4**.

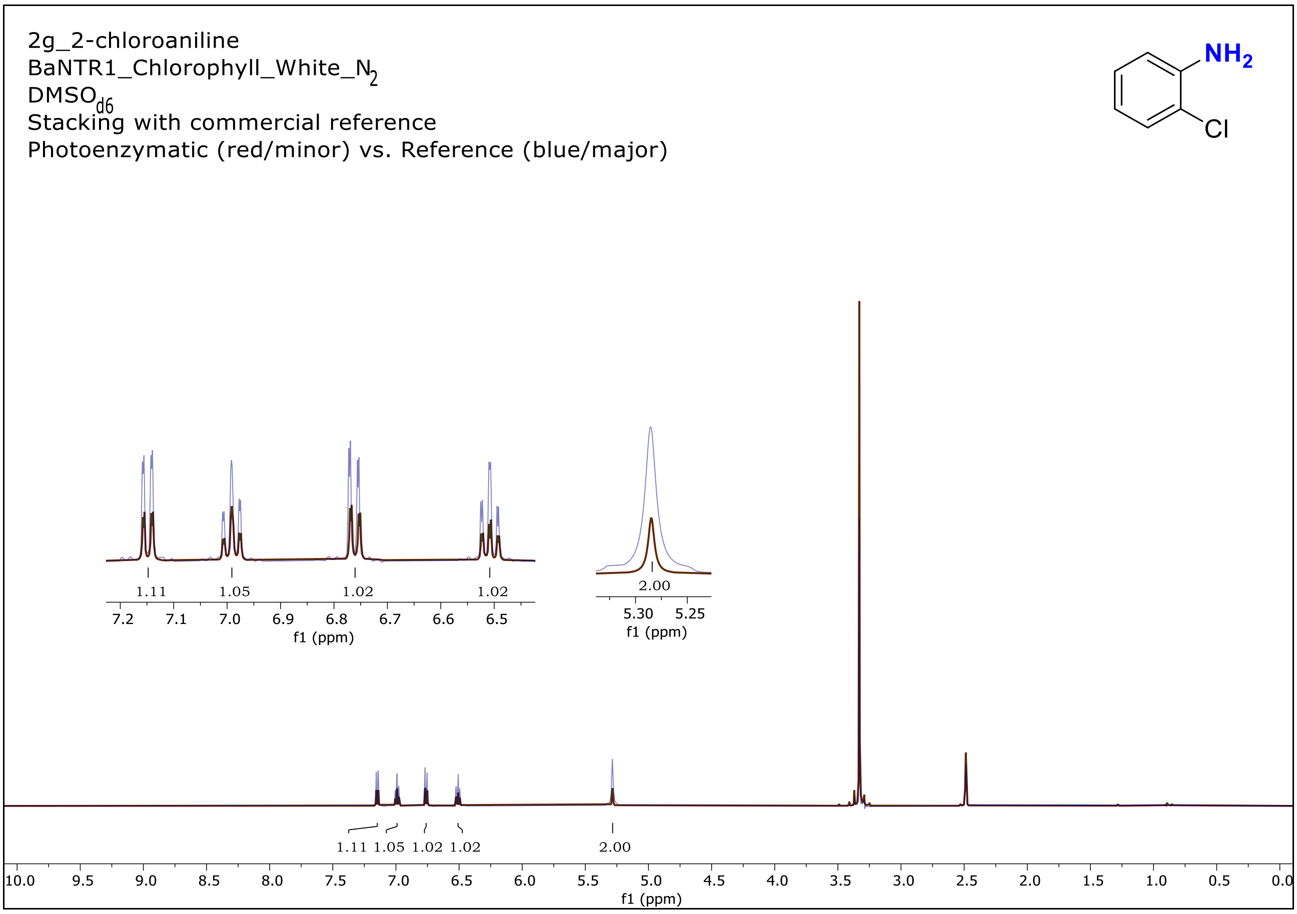

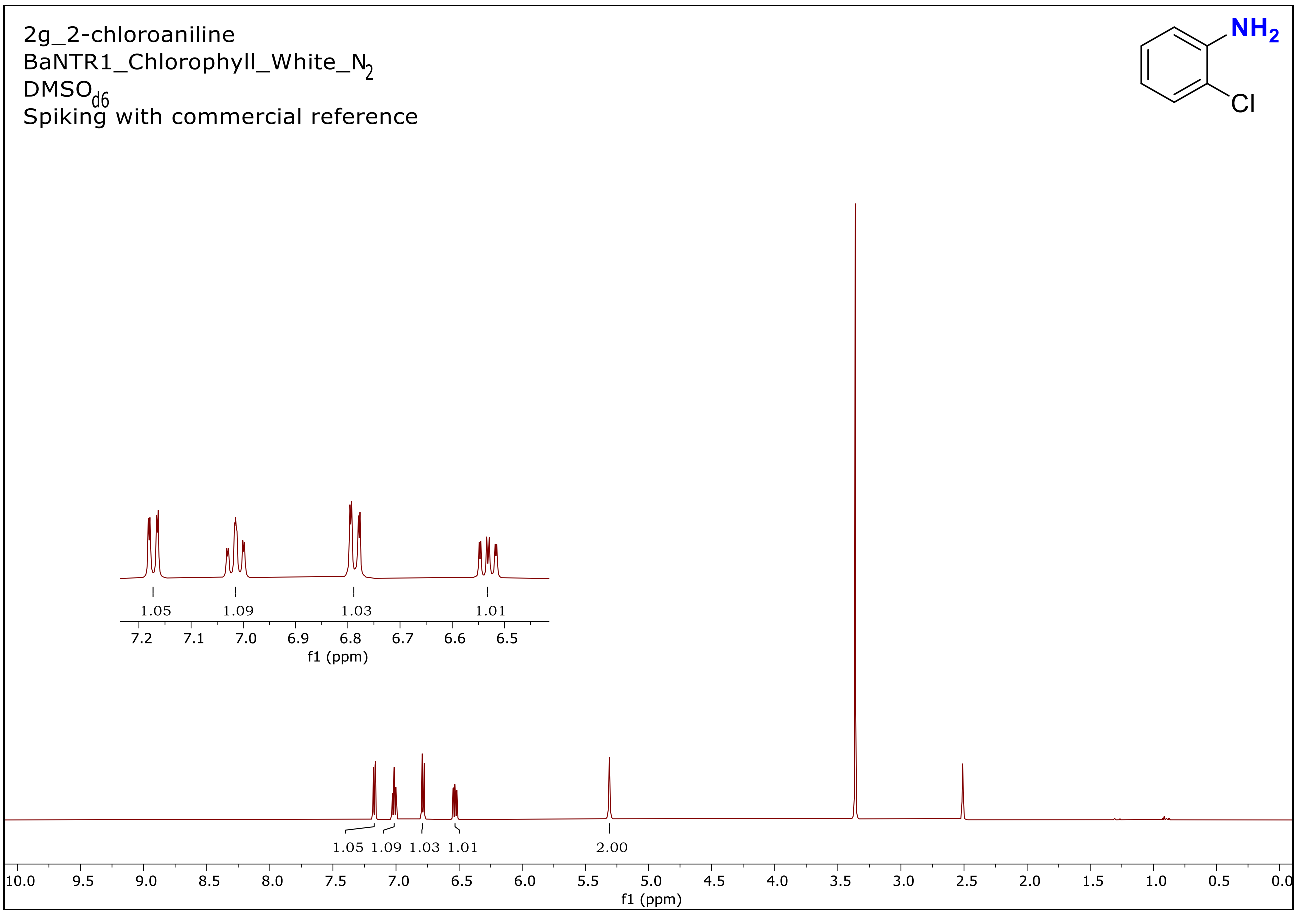

Supplementary Figure 65. Stacking (top) and spiking (bottom) ^{1}H-NMR spectrum of the photoenzymatic product **4** with a commercial reference.

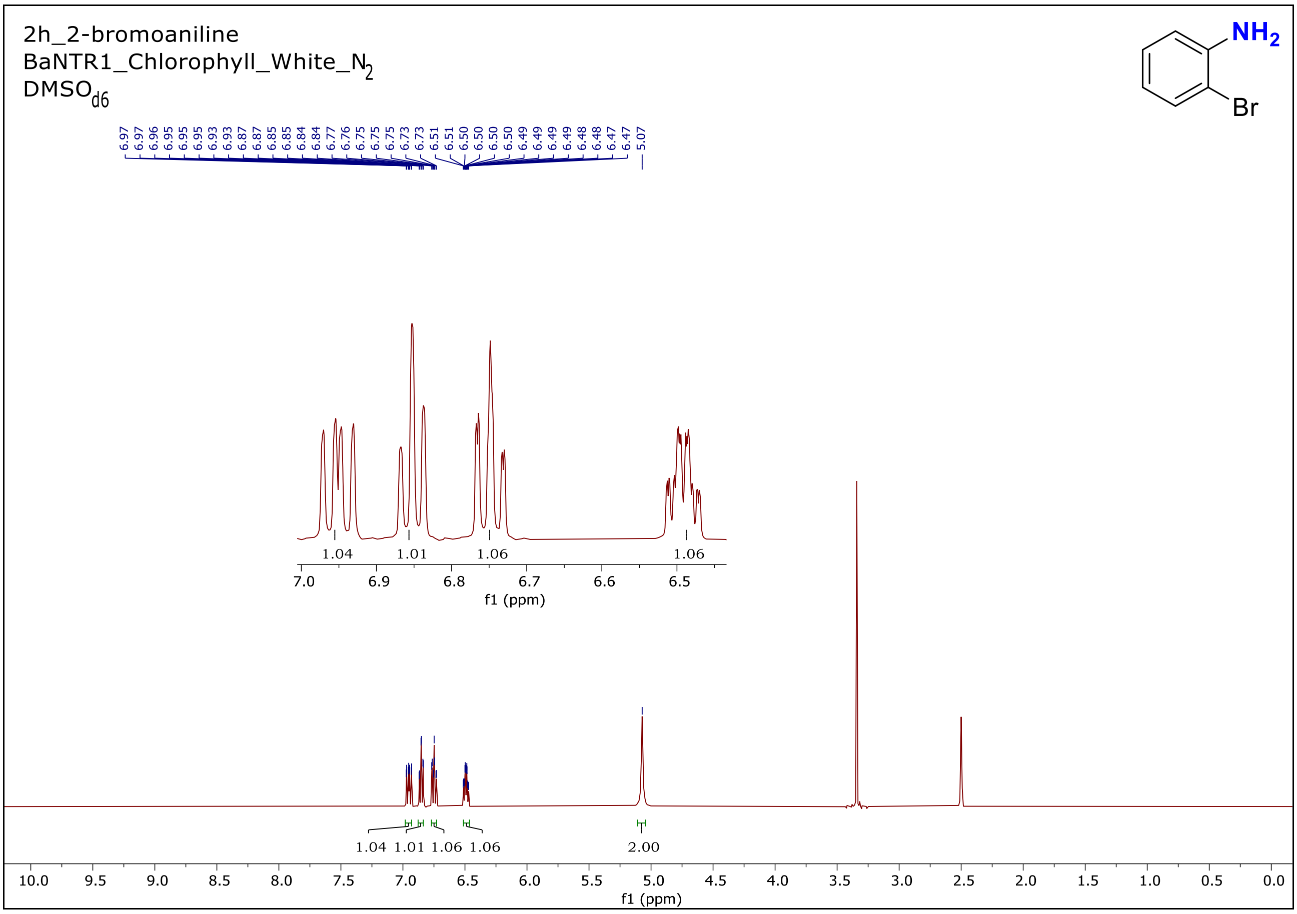

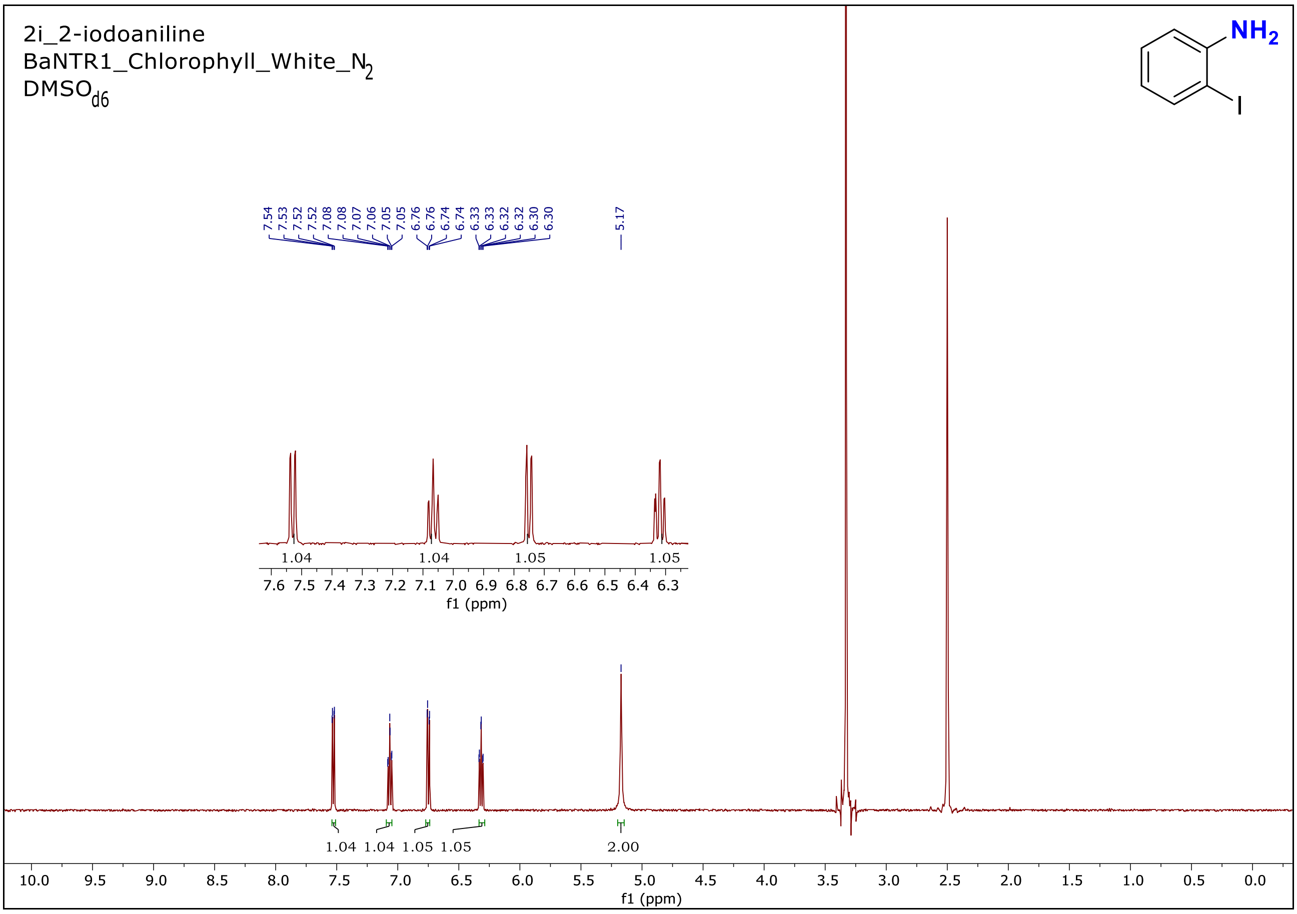

Supplementary Figure 66. ^{1}H-NMR spectrum of the photoenzymatic products **5** (top) and **6** (bottom).

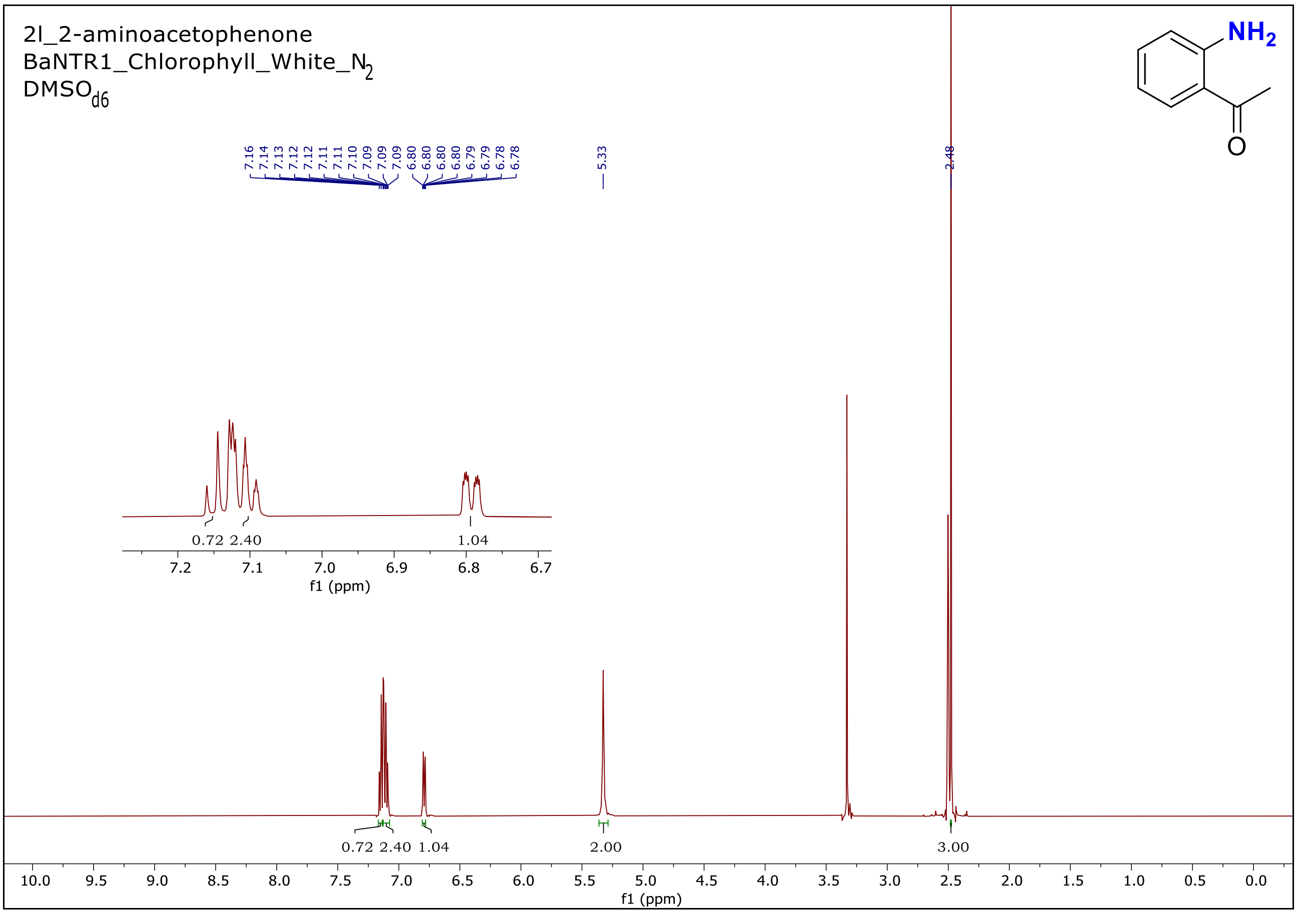

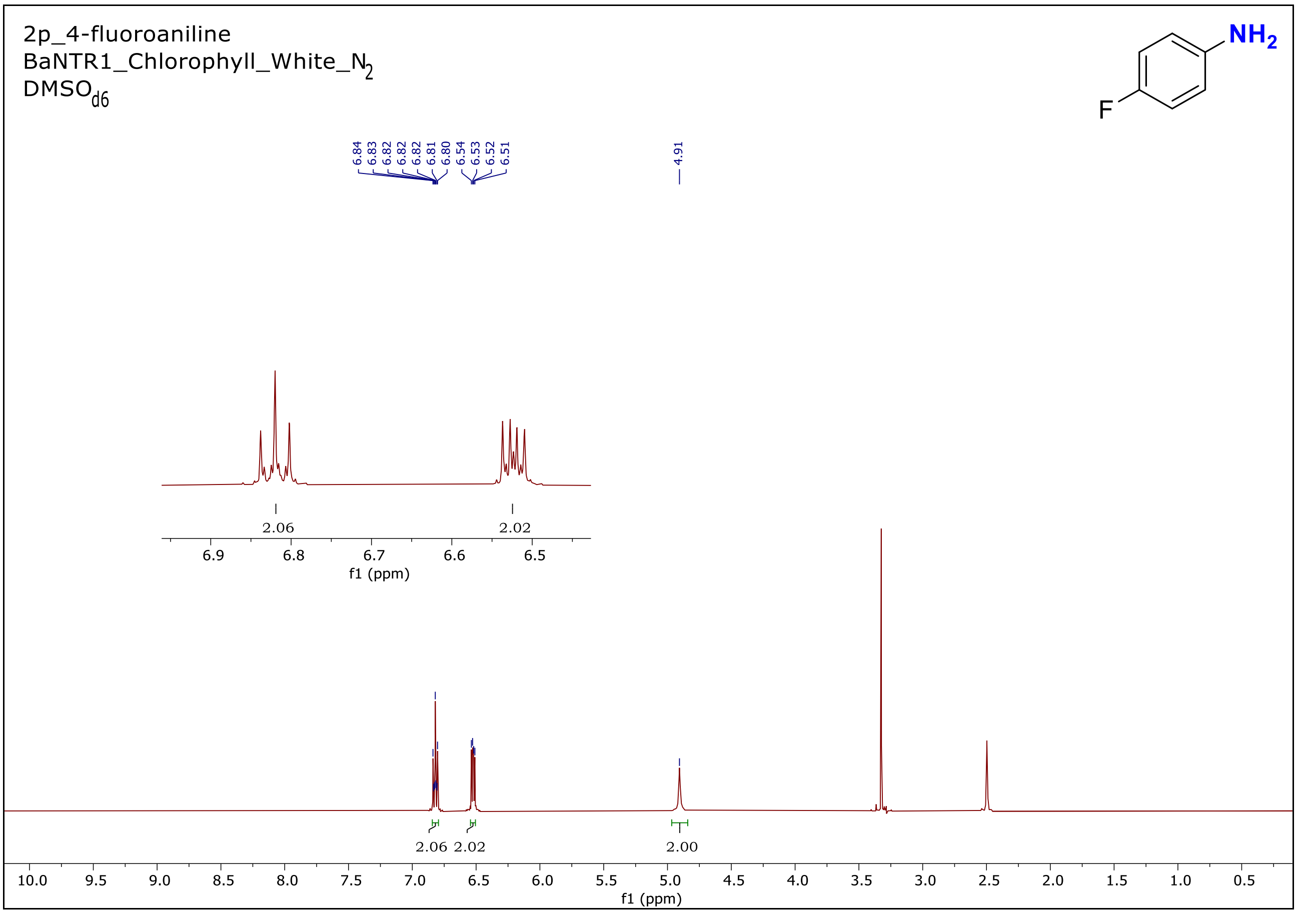

Supplementary Figure 67. ^{1}H-NMR spectrum of the photoenzymatic products **7** (top) and **8** (bottom).

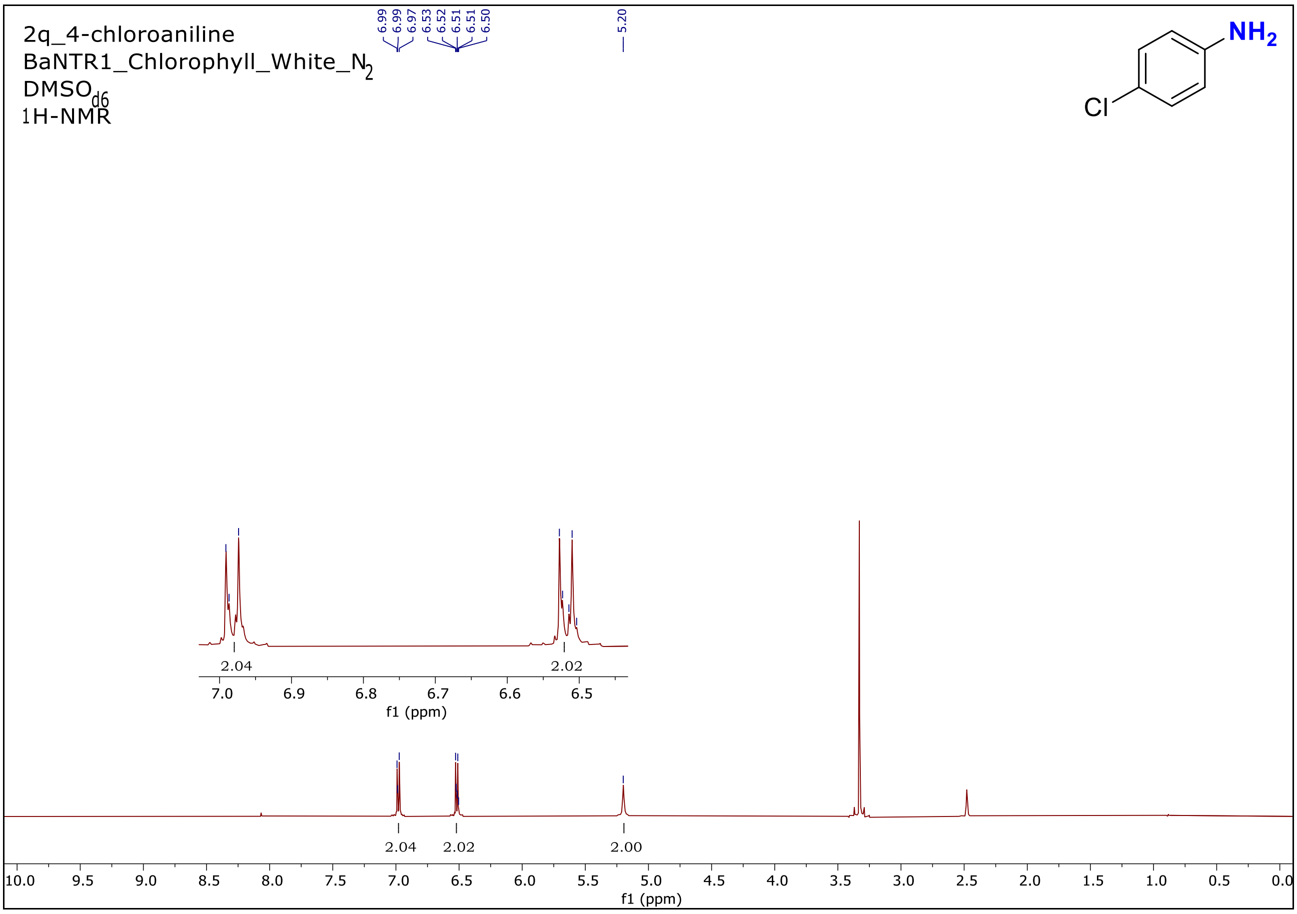

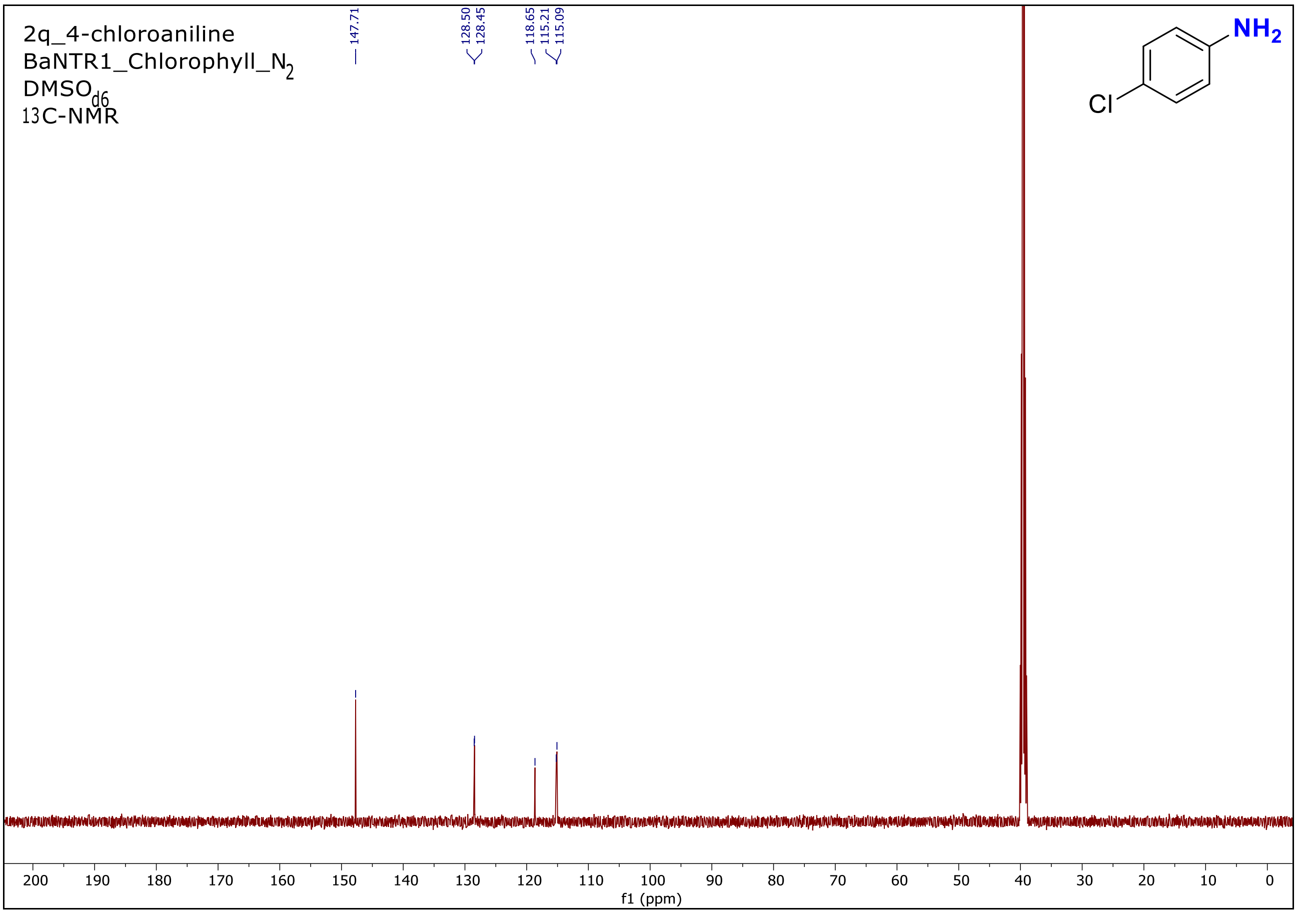

Supplementary Figure 68. ^{1}H-NMR (top) and ^{13}C-NMR (bottom) spectrum of the photoenzymatic product **9**.

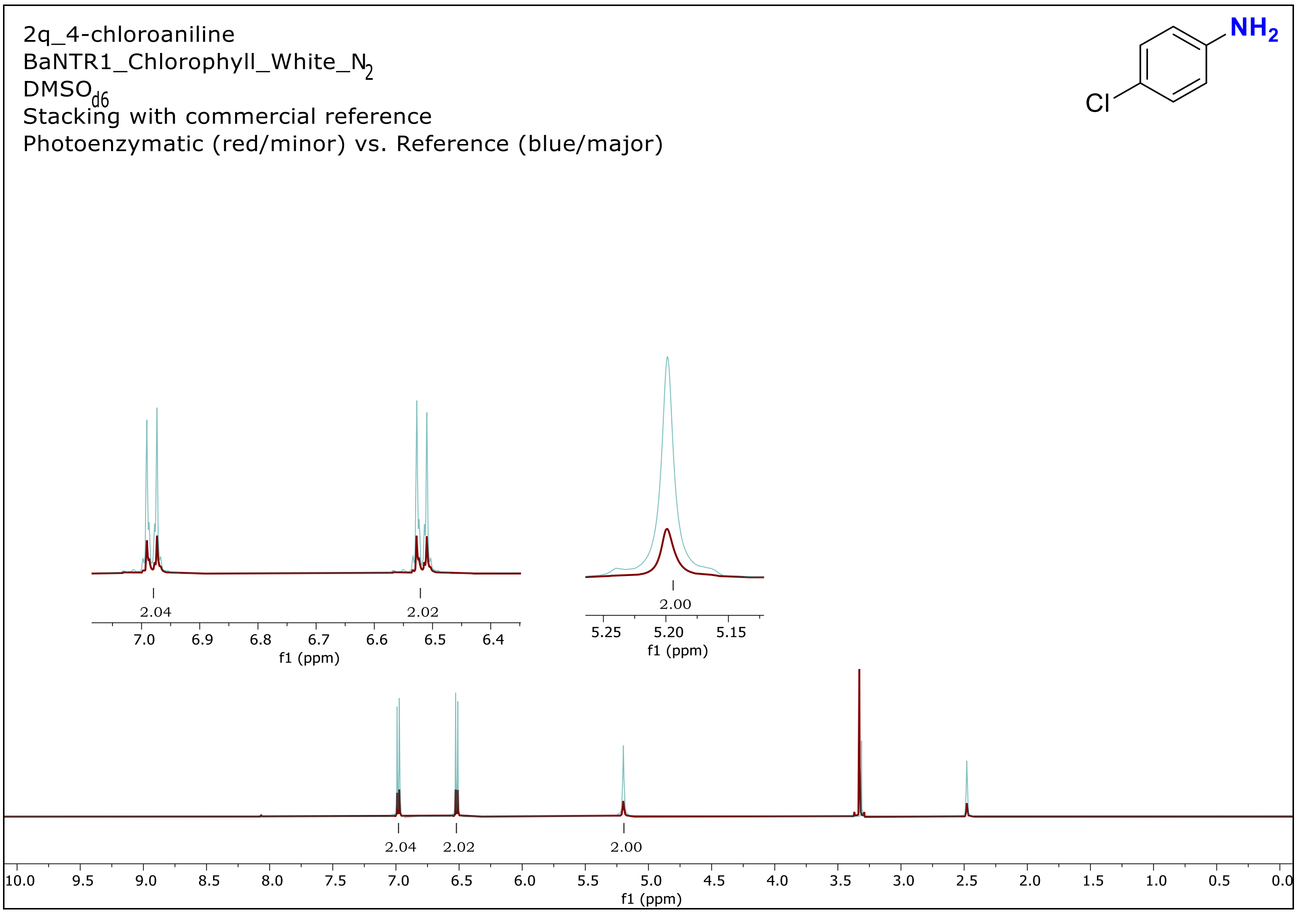

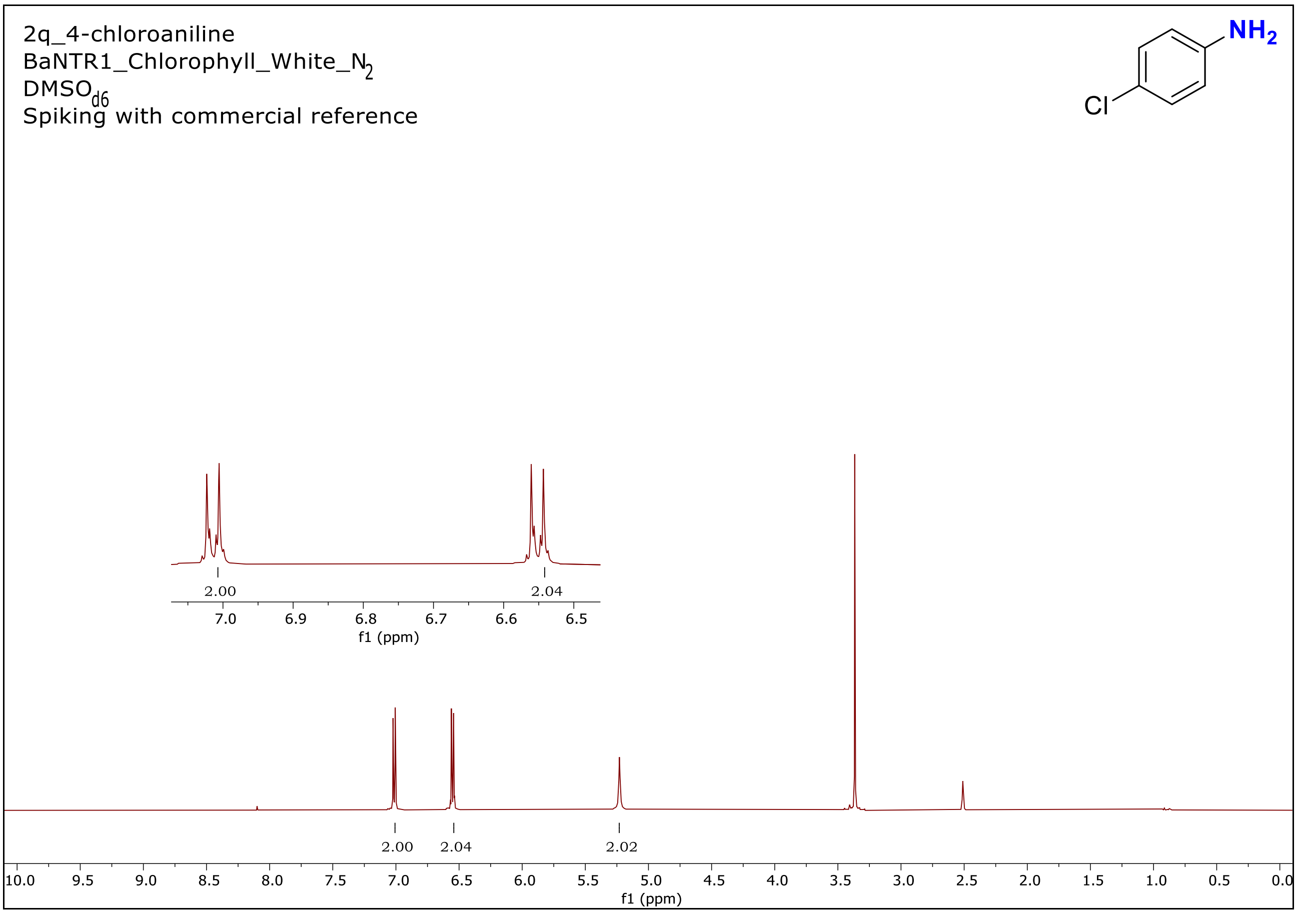

Supplementary Figure 69. Stacking ^{1}H-NMR (top) and spiking (bottom) spectrum of the photoenzymatic product **9** with a commercial reference.

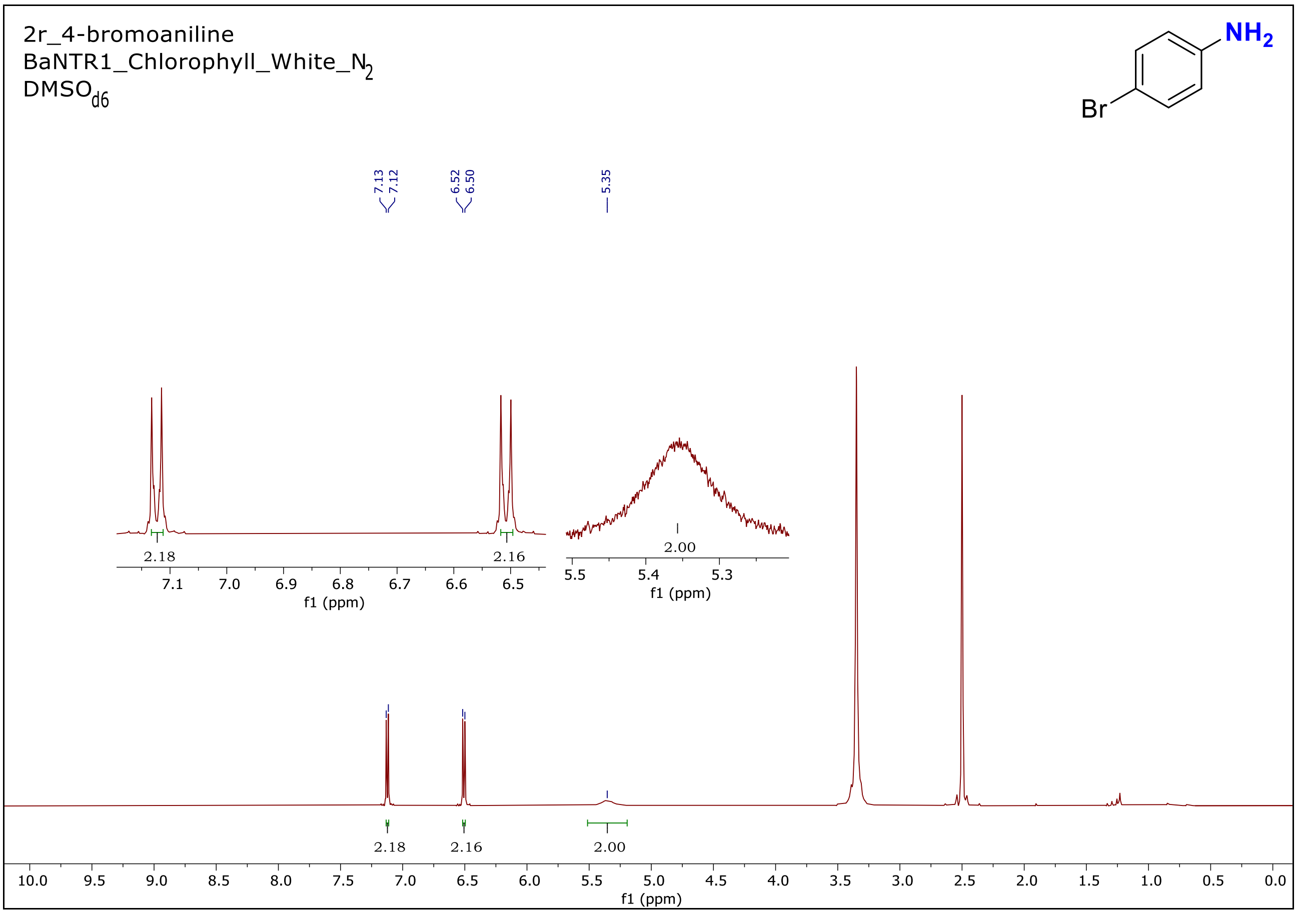

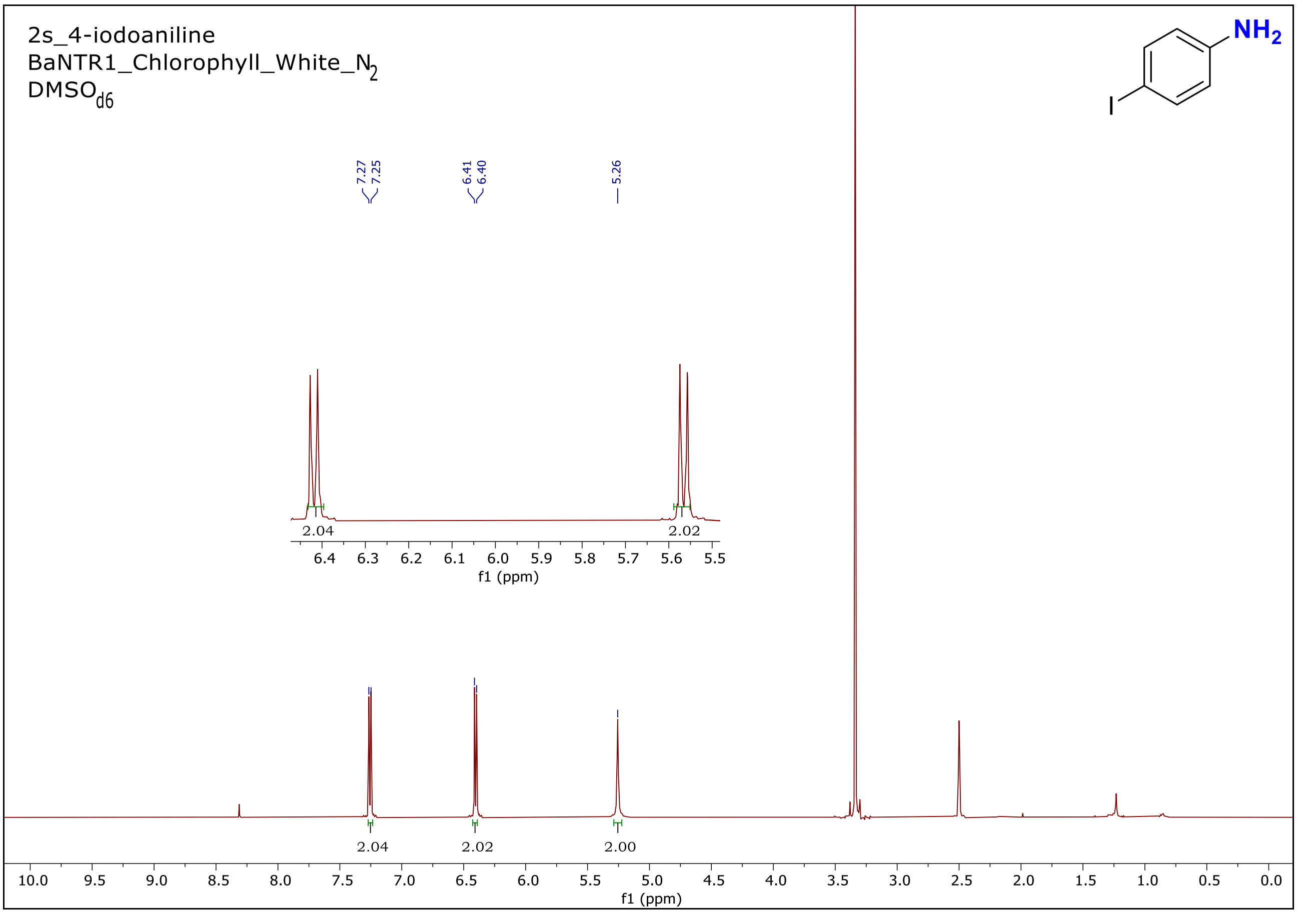

Supplementary Figure 70. ^{1}H-NMR spectrum of the photoenzymatic products **10** (top) and **11** (bottom).

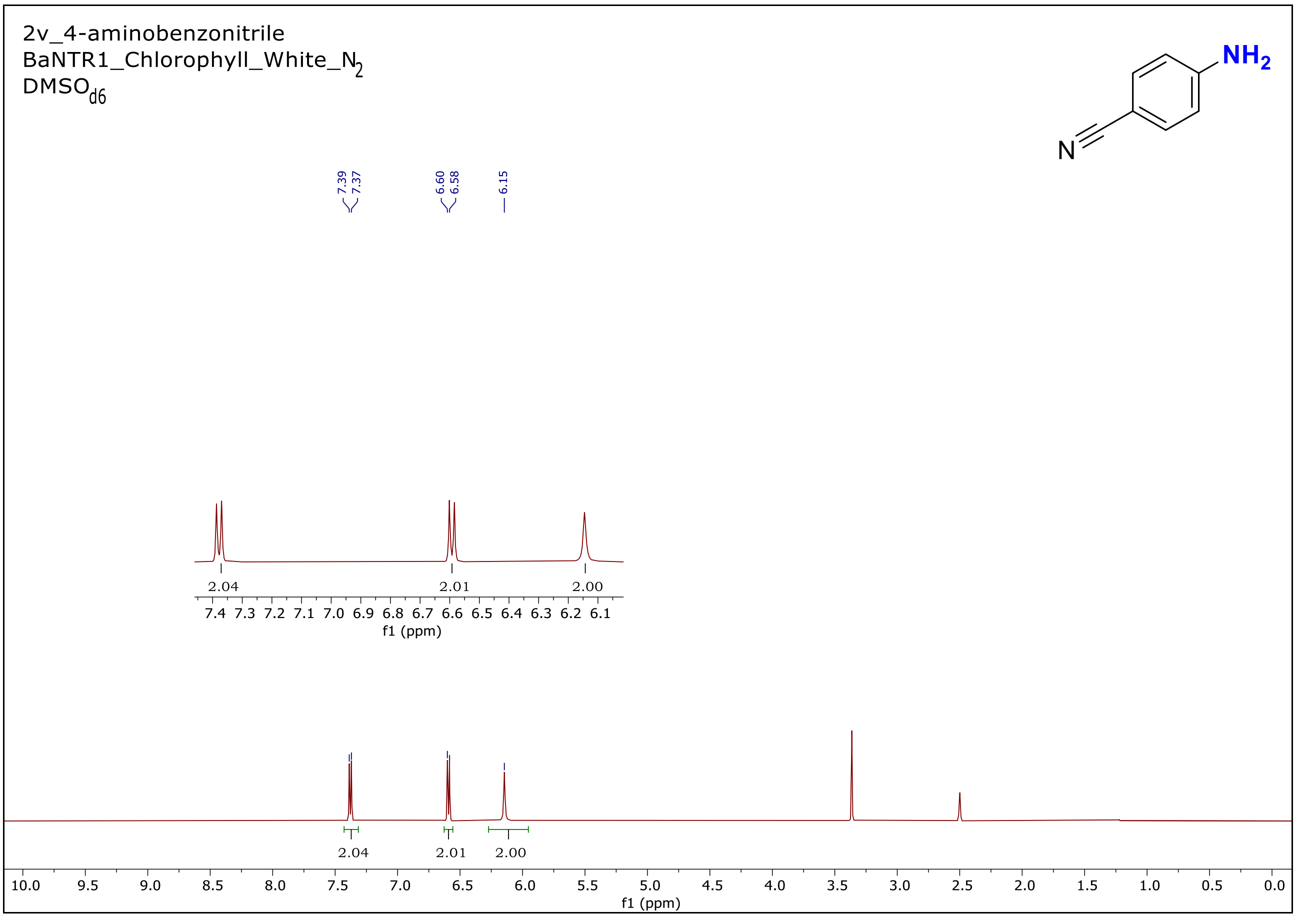

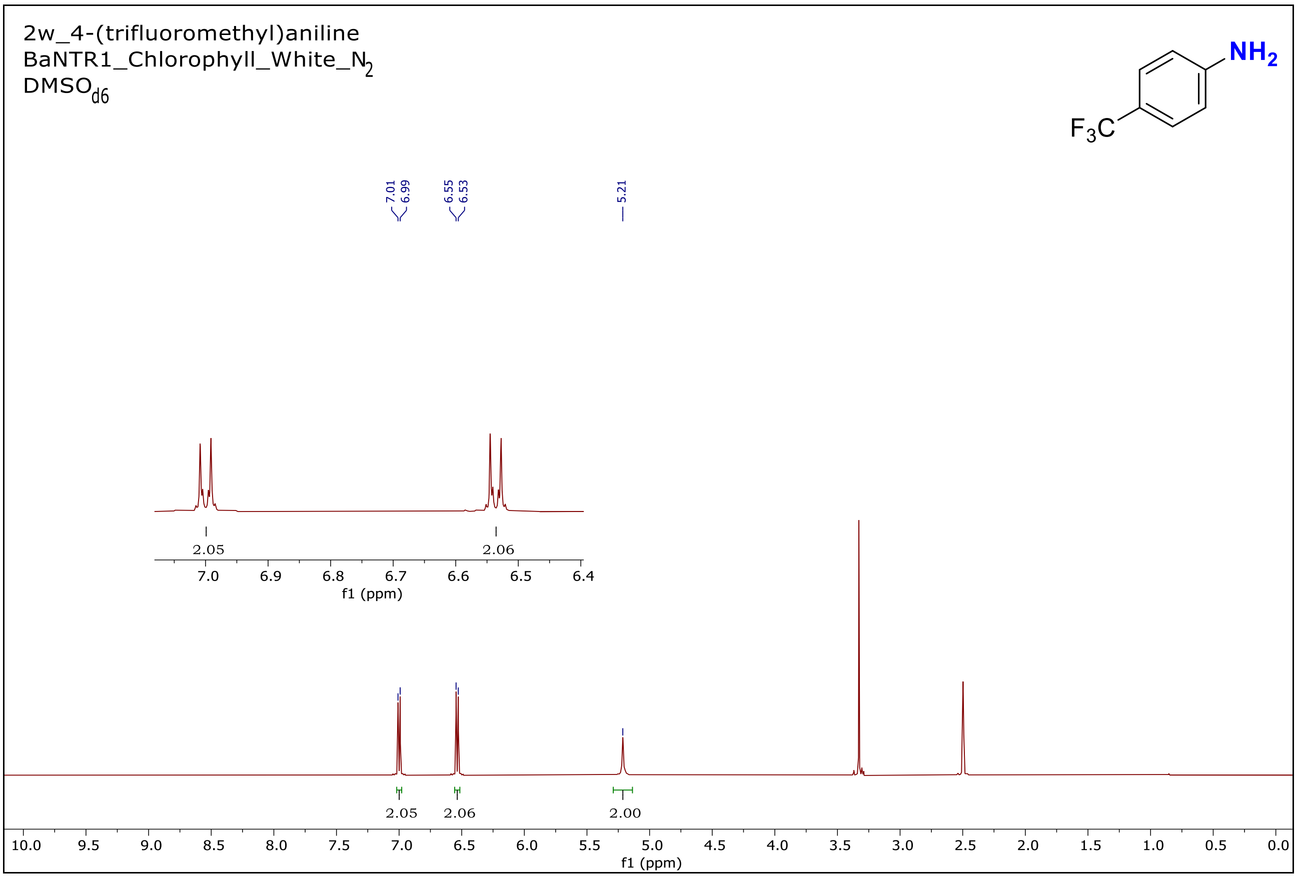

Supplementary Figure 71. ^{1}H-NMR spectrum of the photoenzymatic products **12** (top) and **13** (bottom).

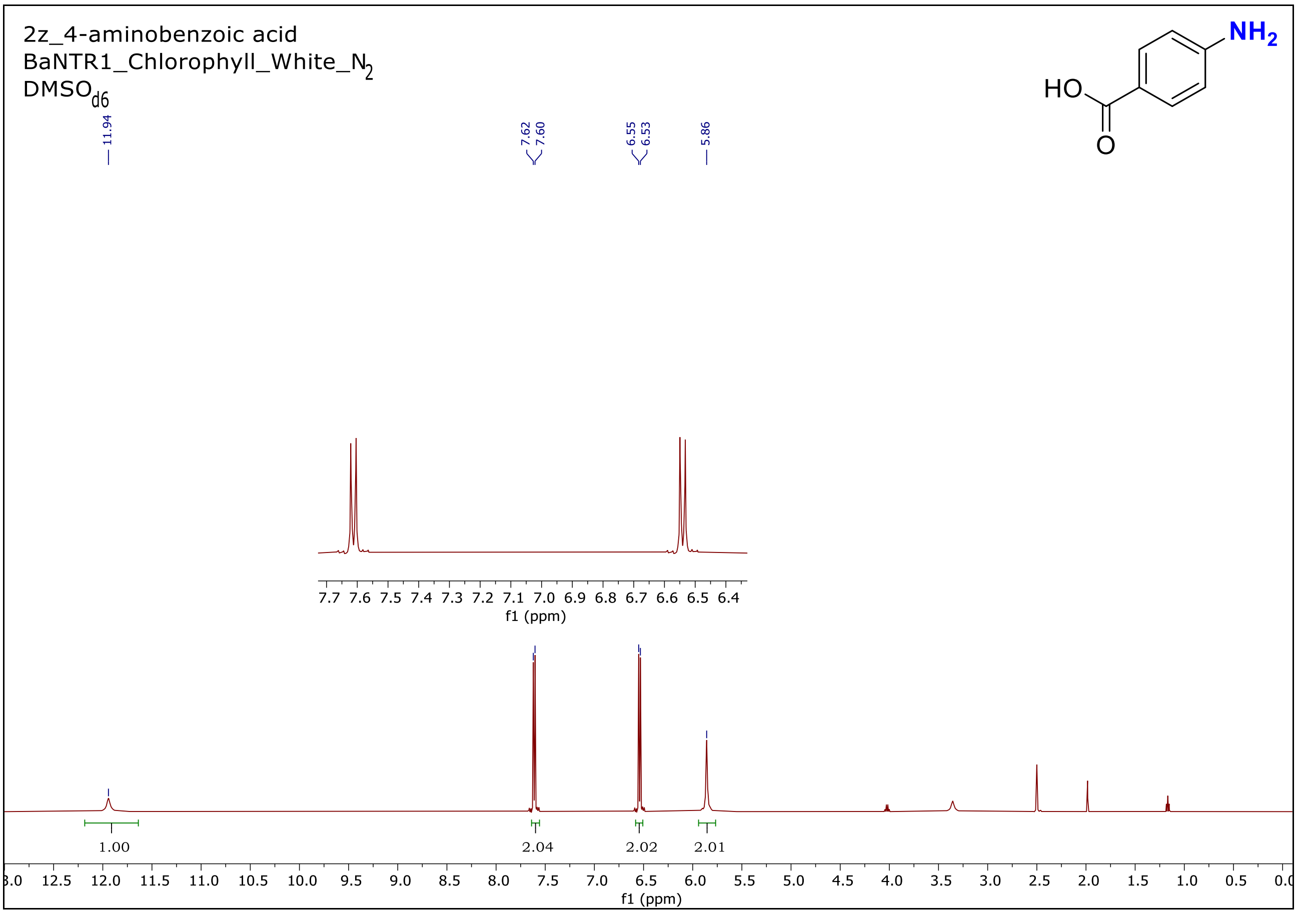

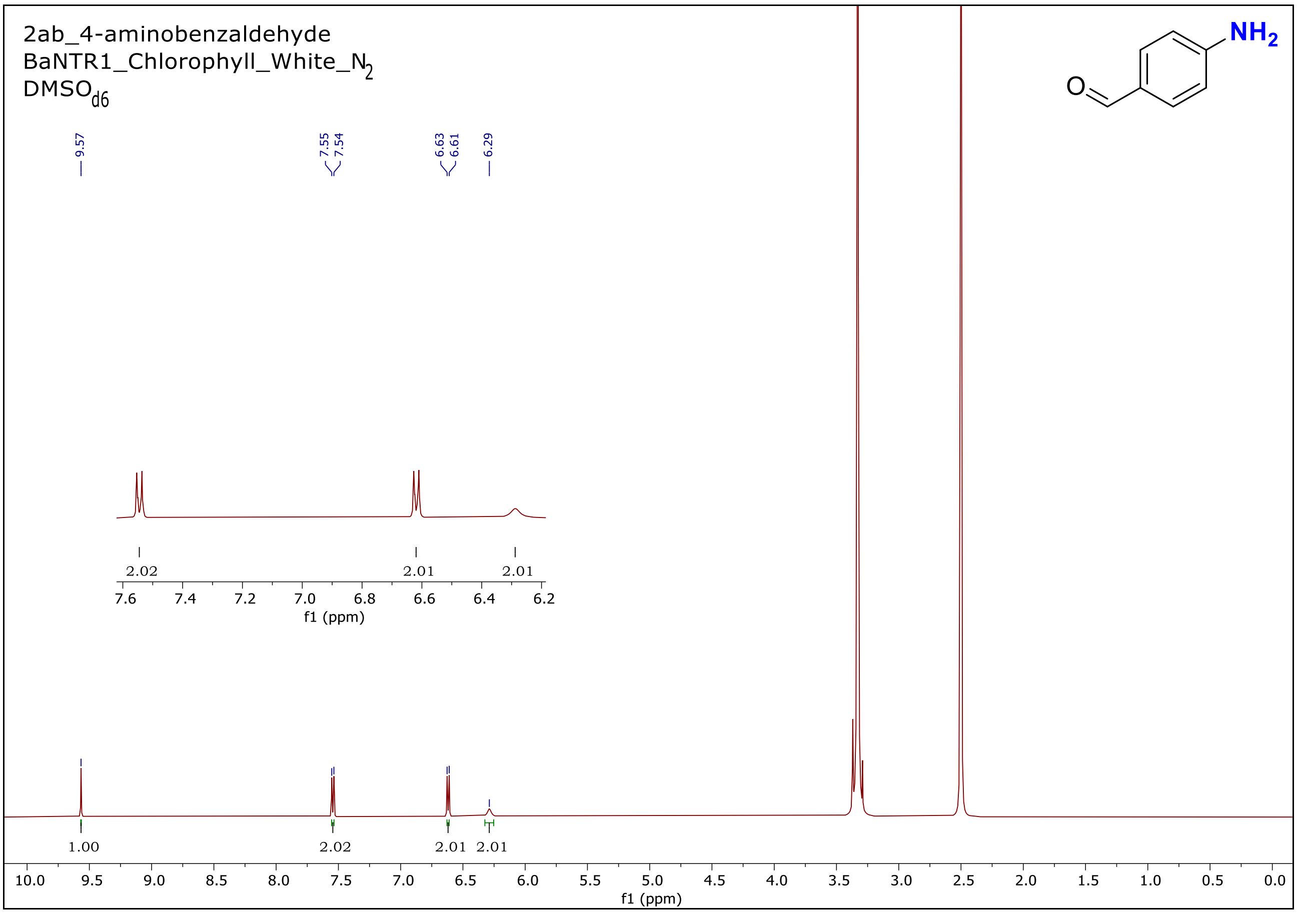

Supplementary Figure 72. ^{1}H-NMR spectrum of the photoenzymatic products **14** (top) and **15** (bottom).

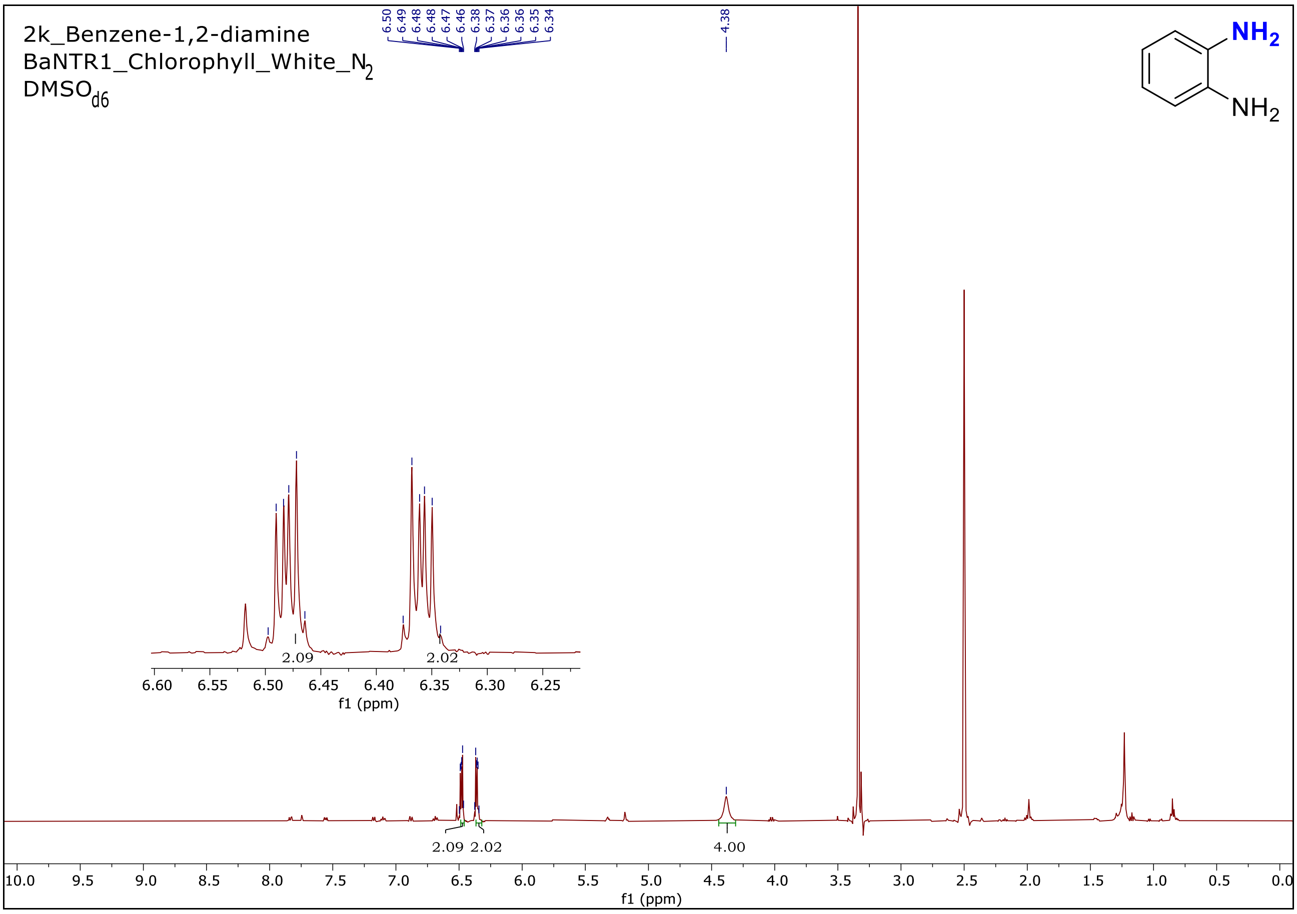

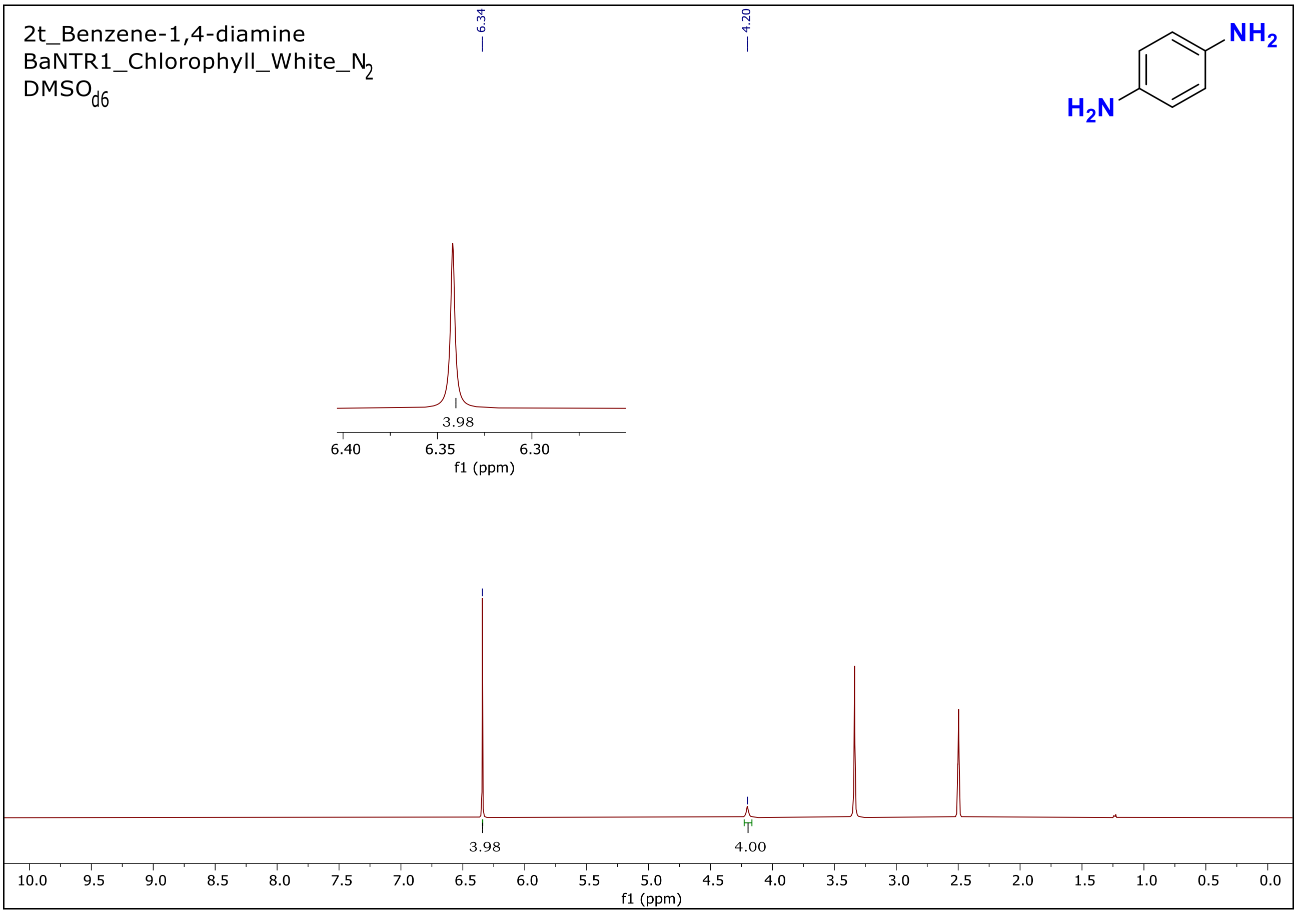

Supplementary Figure 73. ^{1}H-NMR spectrum of the photoenzymatic products **16** (top) and **17** (bottom).

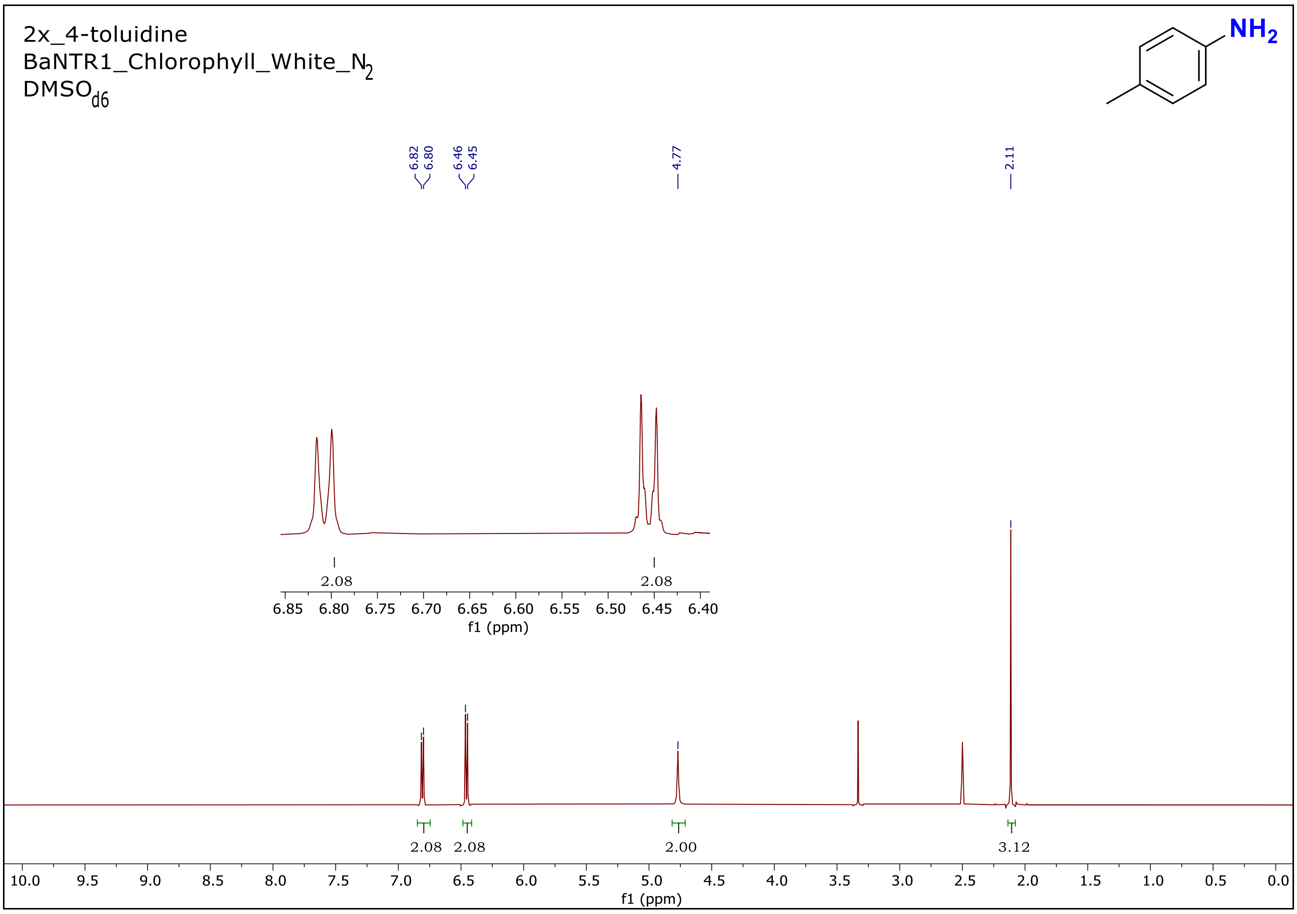

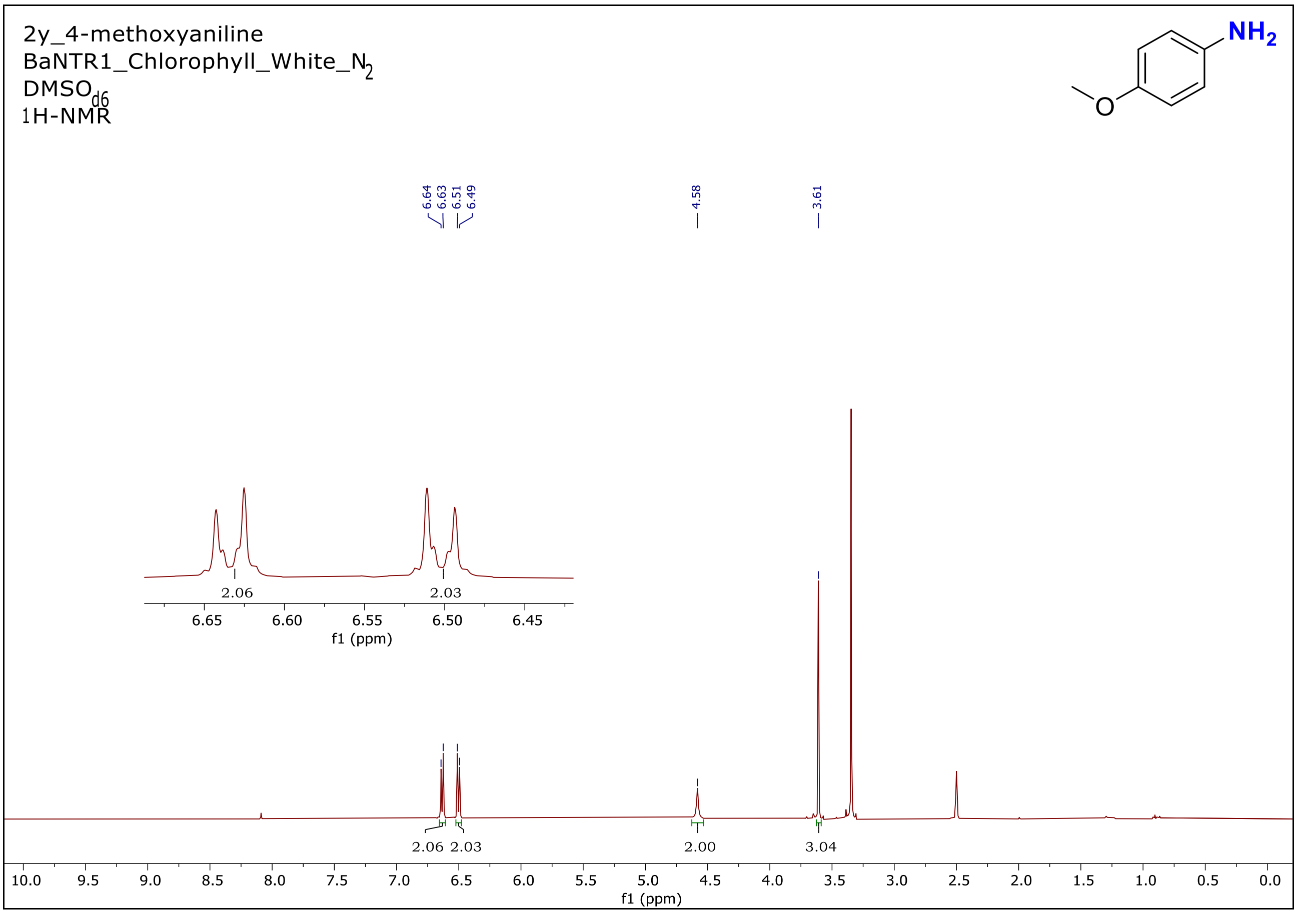

Supplementary Figure 74. ^{1}H-NMR spectrum of the photoenzymatic products **18** (top) and **19** (bottom).

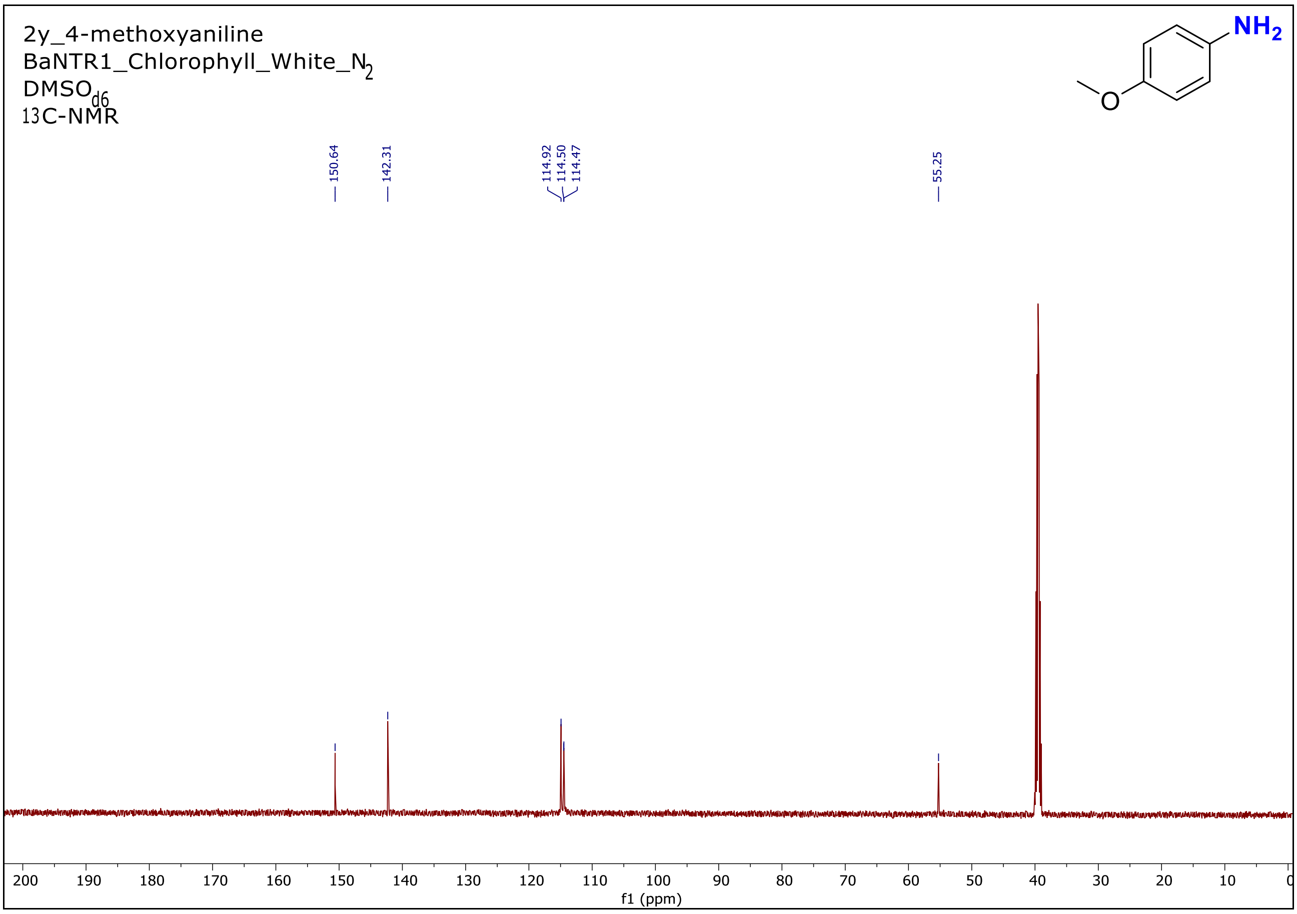

Supplementary Figure 75. ^{13}C-NMR spectrum of the photoenzymatic product **19**.

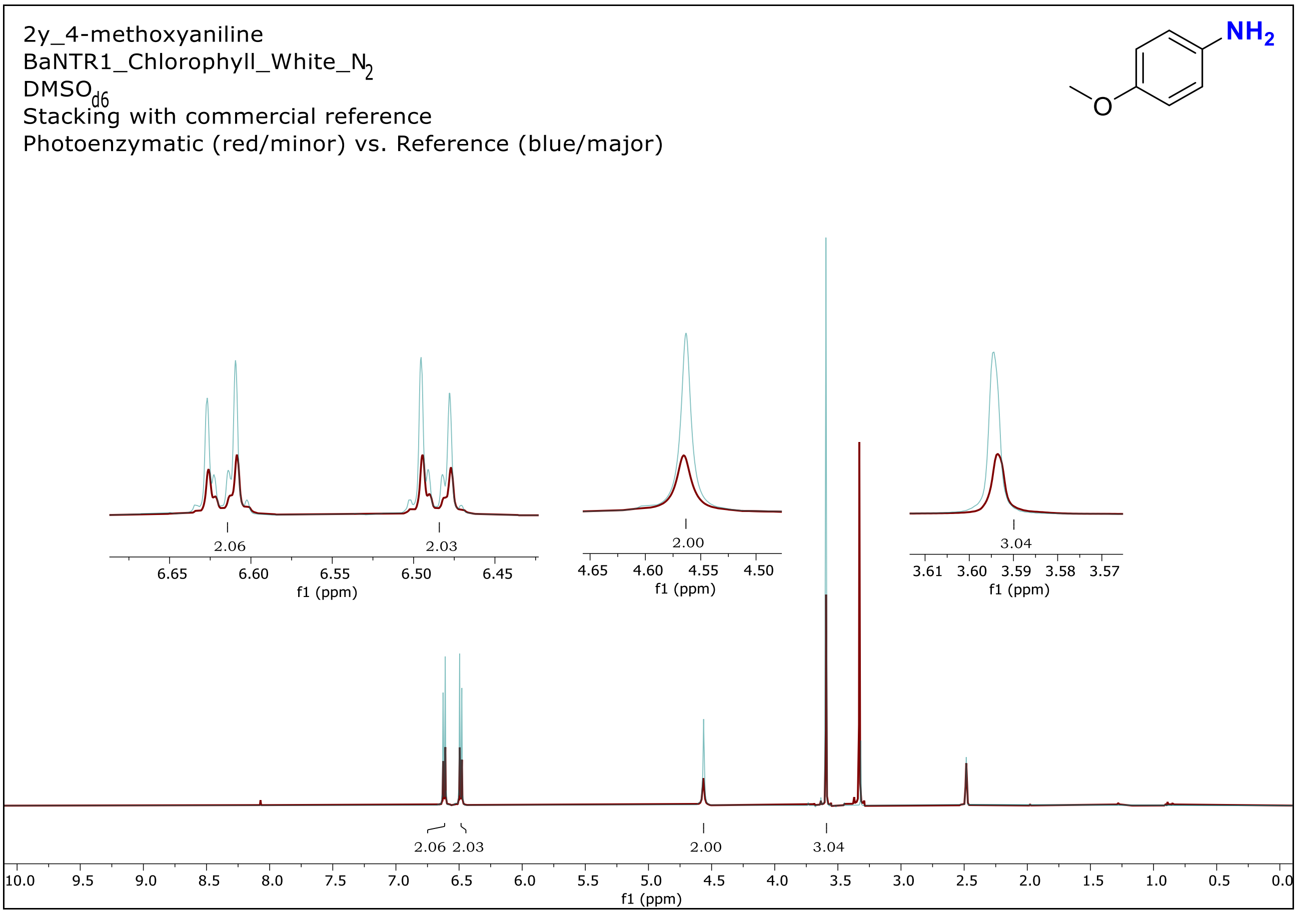

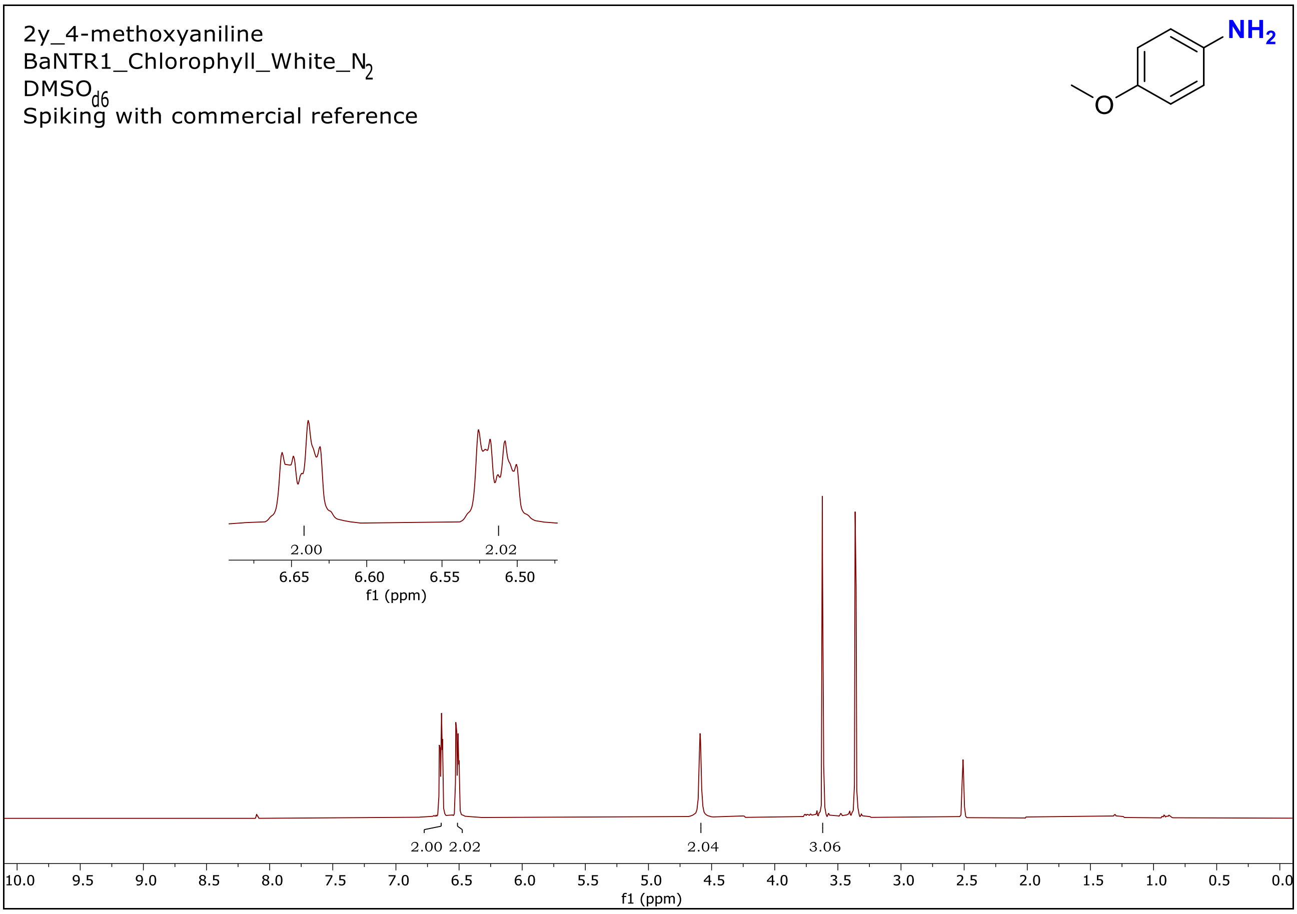

Supplementary Figure 76. Stacking ^{1}H-NMR (top) and spiking (bottom) spectrum of the photoenzymatic product **19** with a commercial reference.

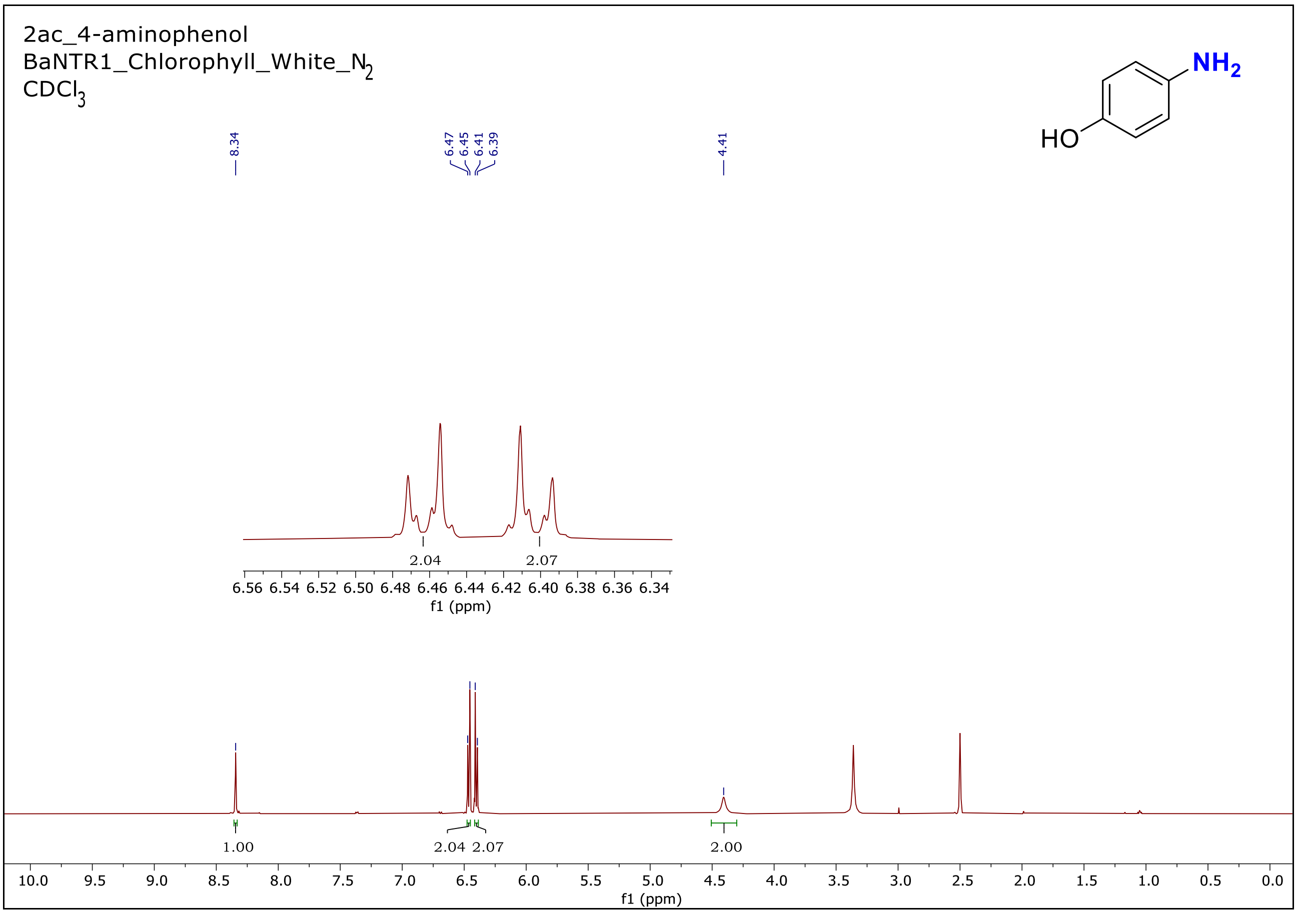

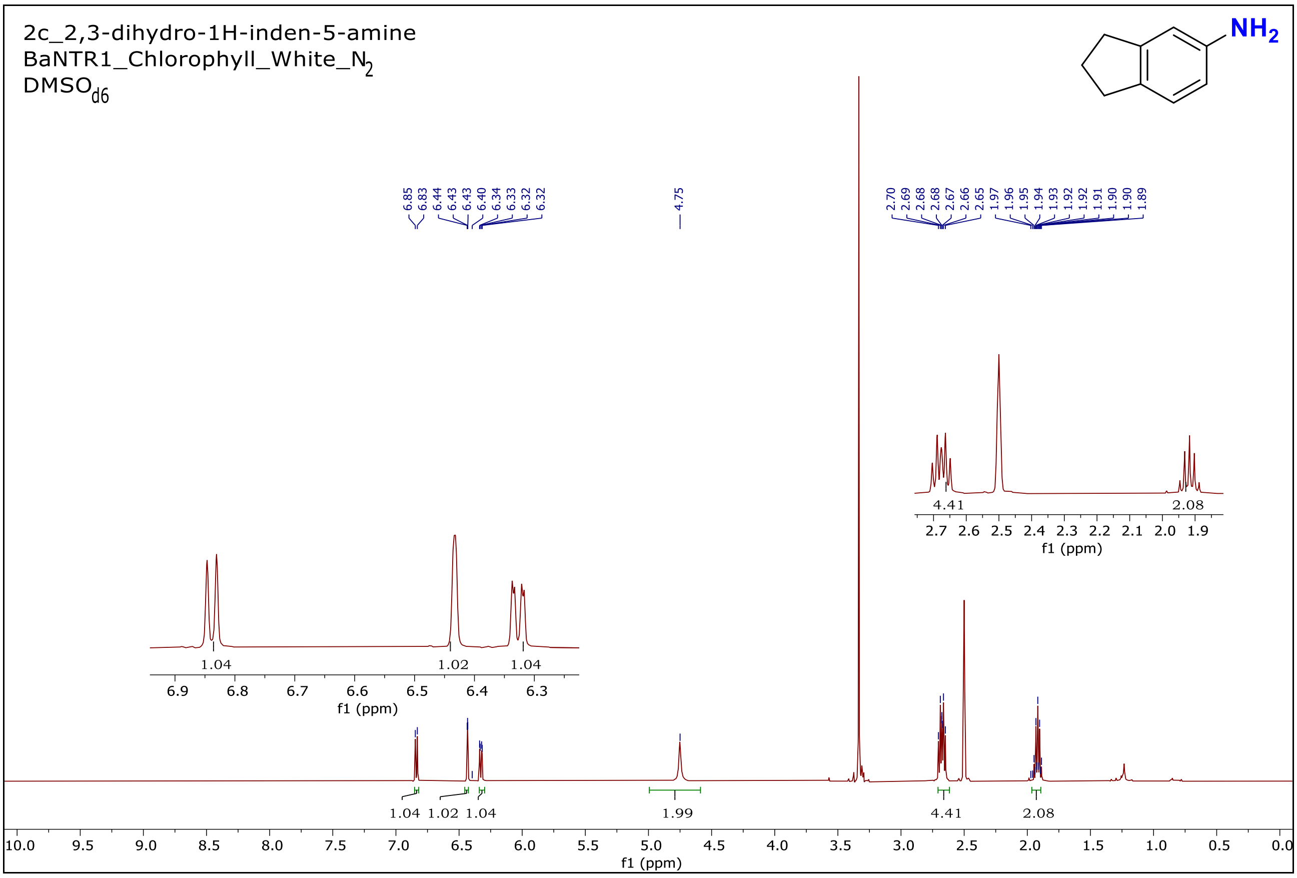

Supplementary Figure 77. ^{1}H-NMR spectrum of the photoenzymatic products **20** (top) and **21** (bottom).

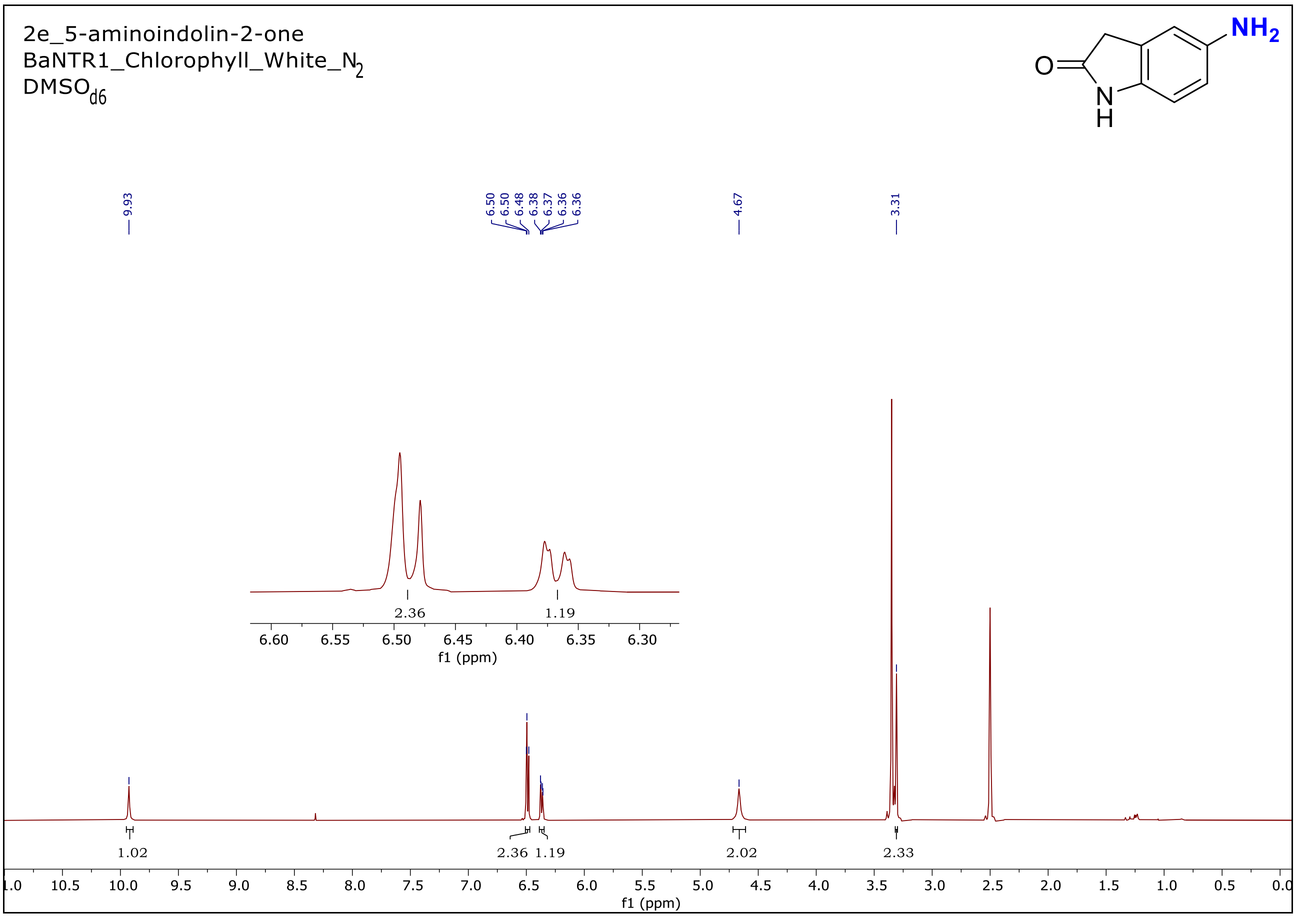

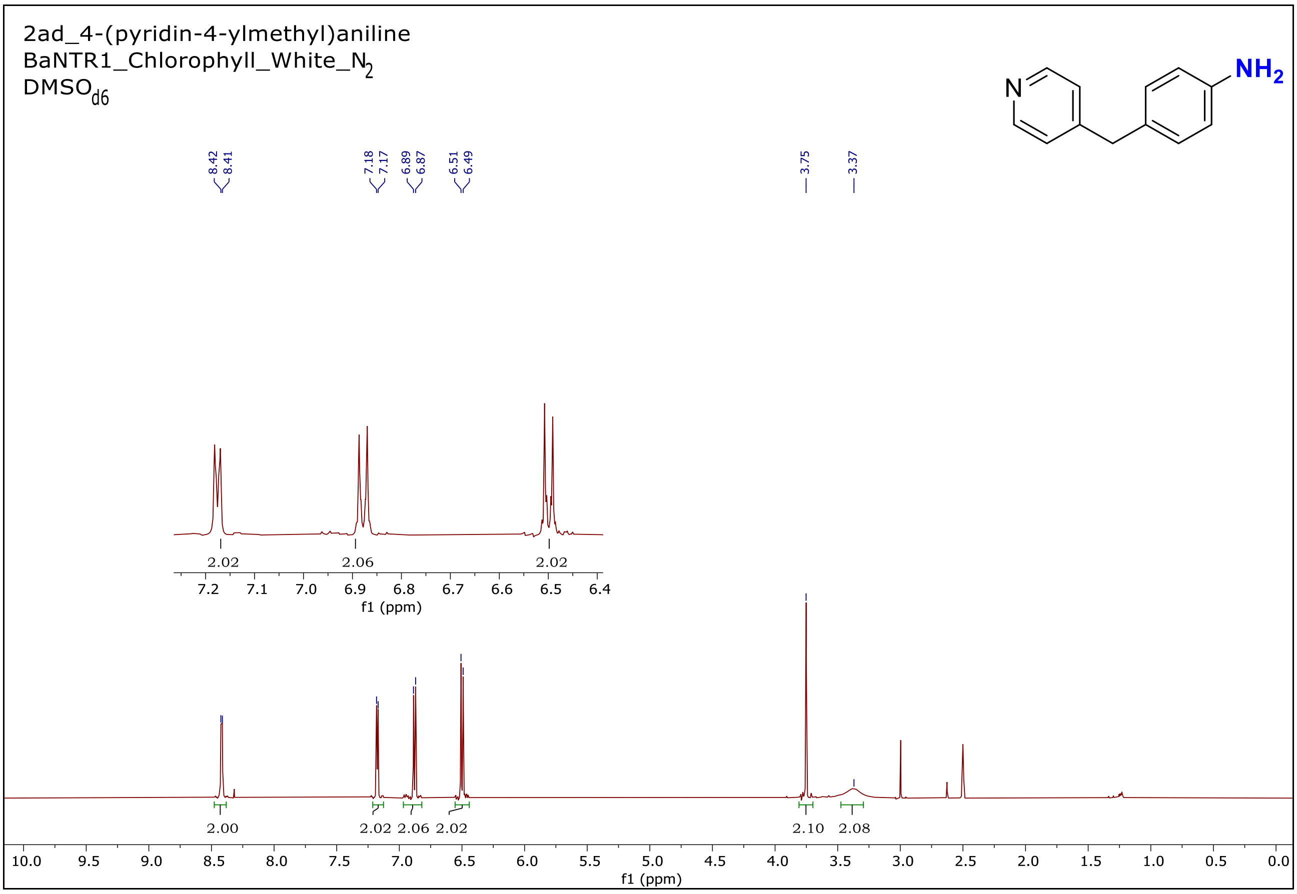

Supplementary Figure 78. ^{1}H-NMR spectrum of the photoenzymatic products **22** (top) and **23** (bottom).

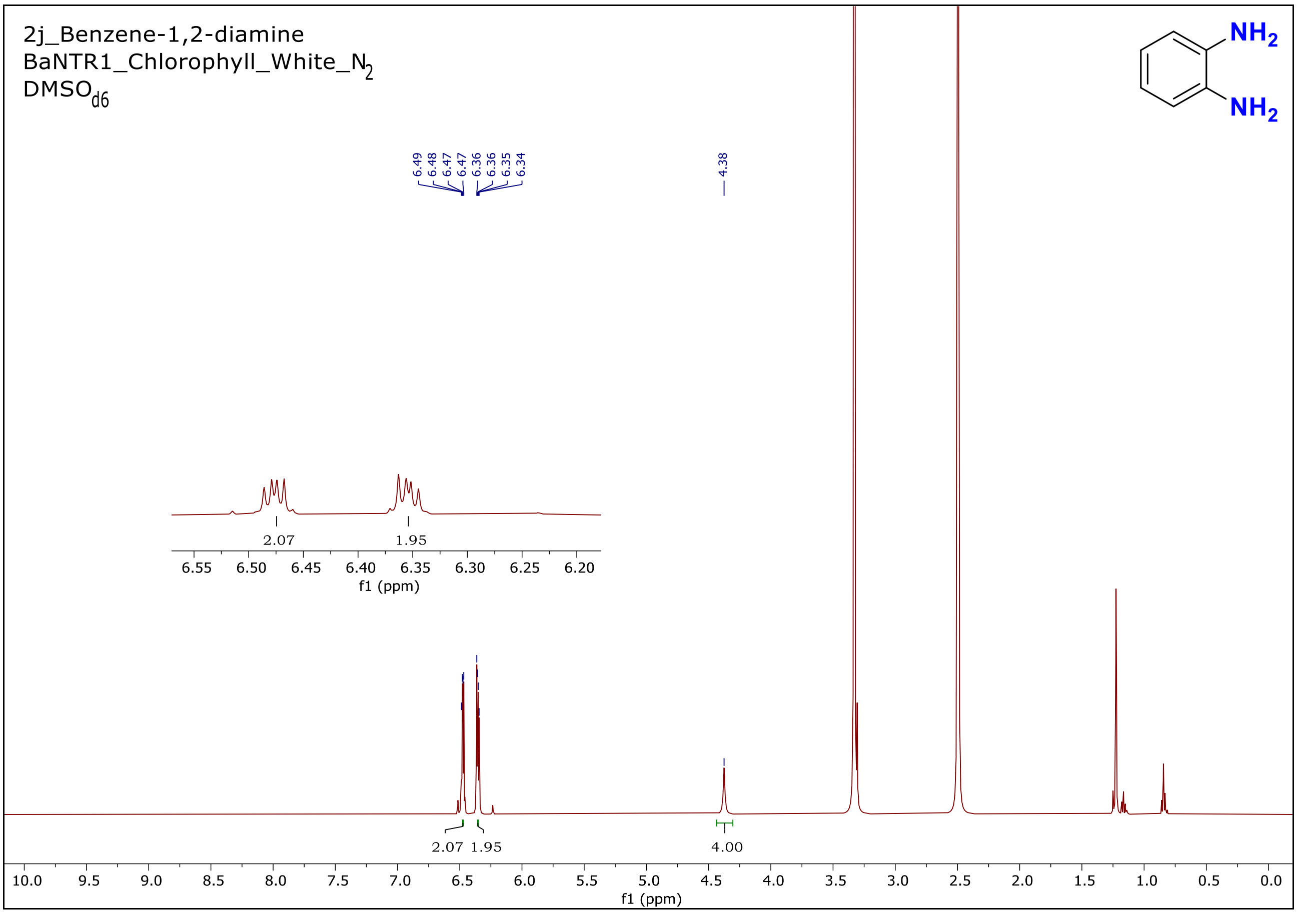

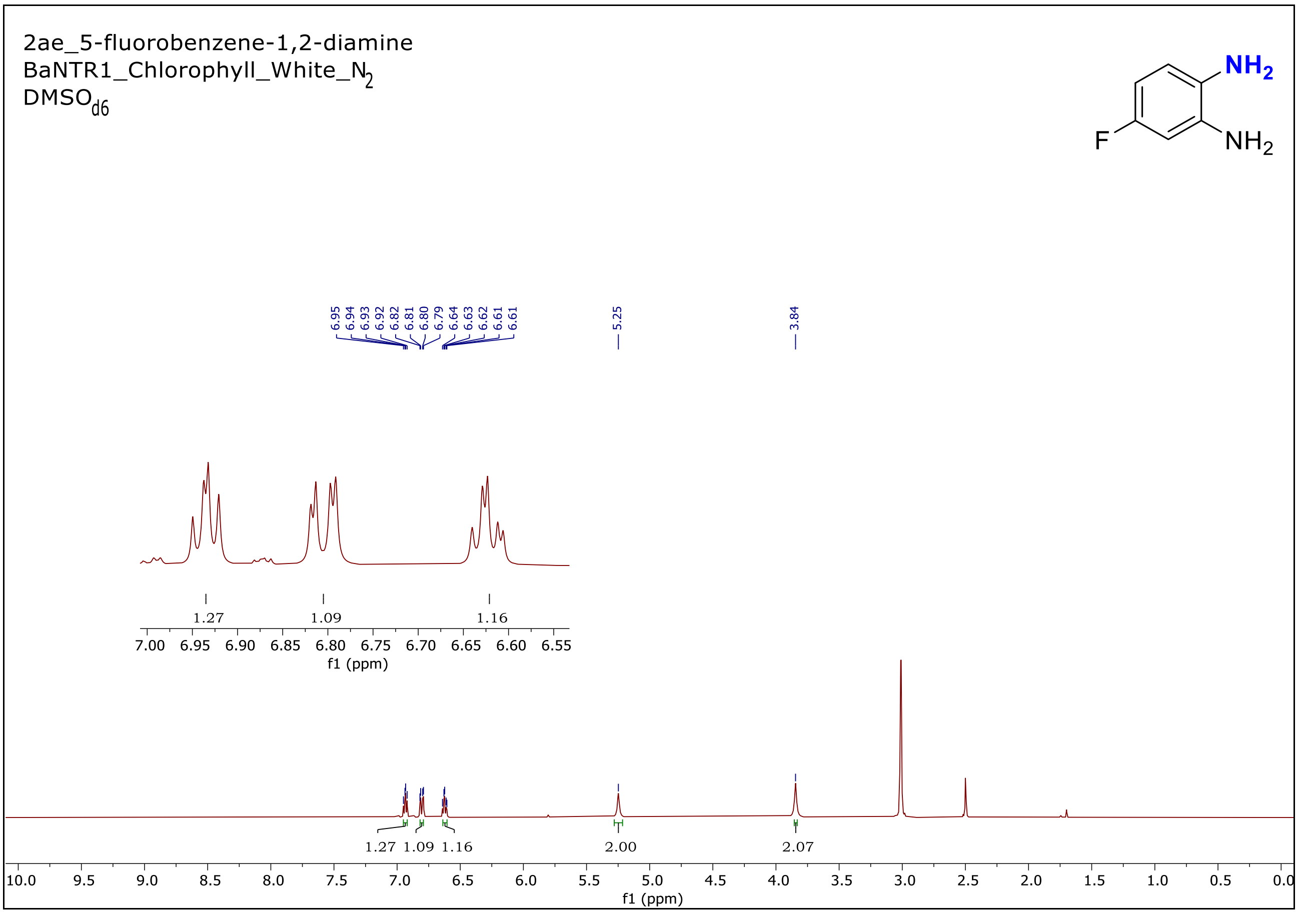

Supplementary Figure 79. ^{1}H-NMR spectrum of the photoenzymatic products **24** (top) and **25** (bottom).

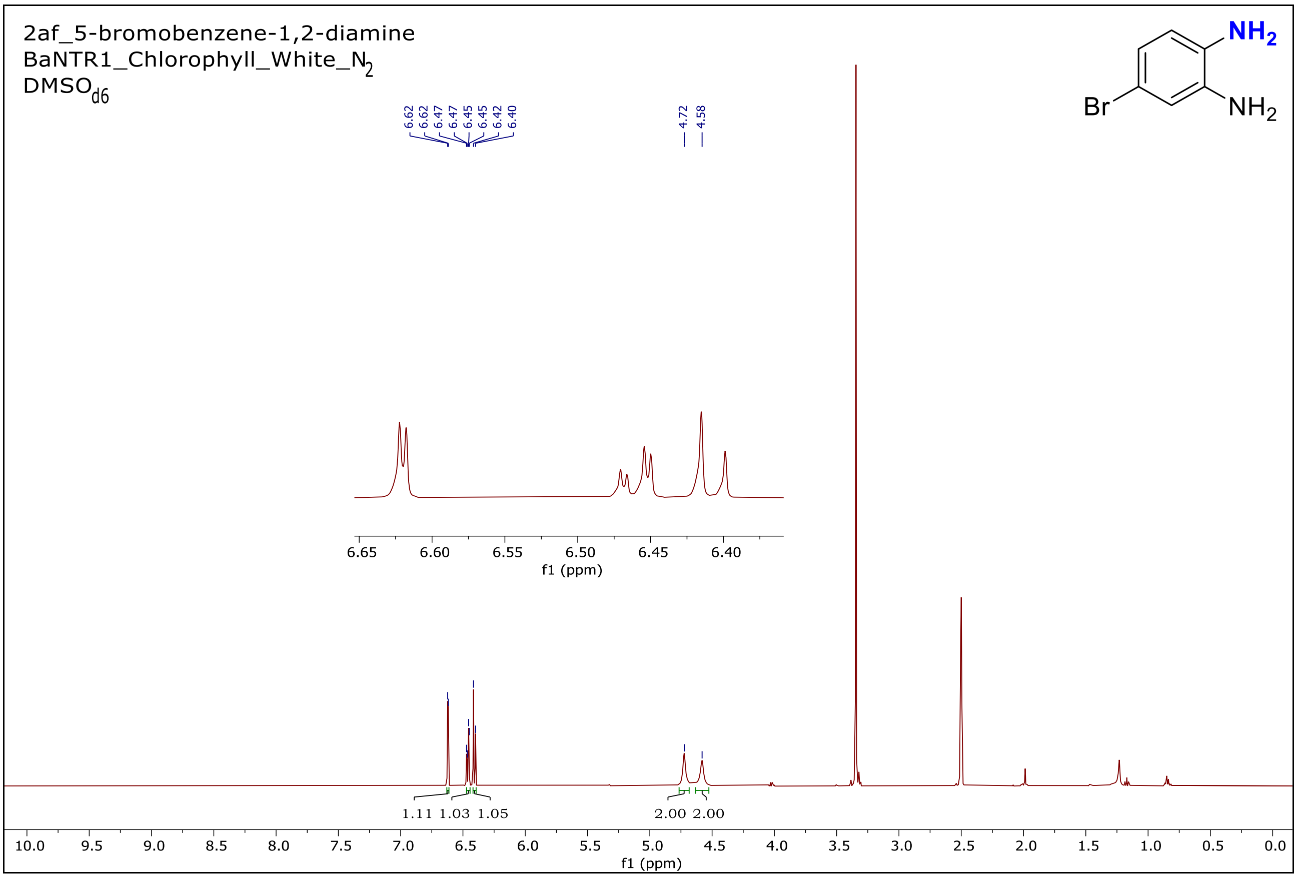

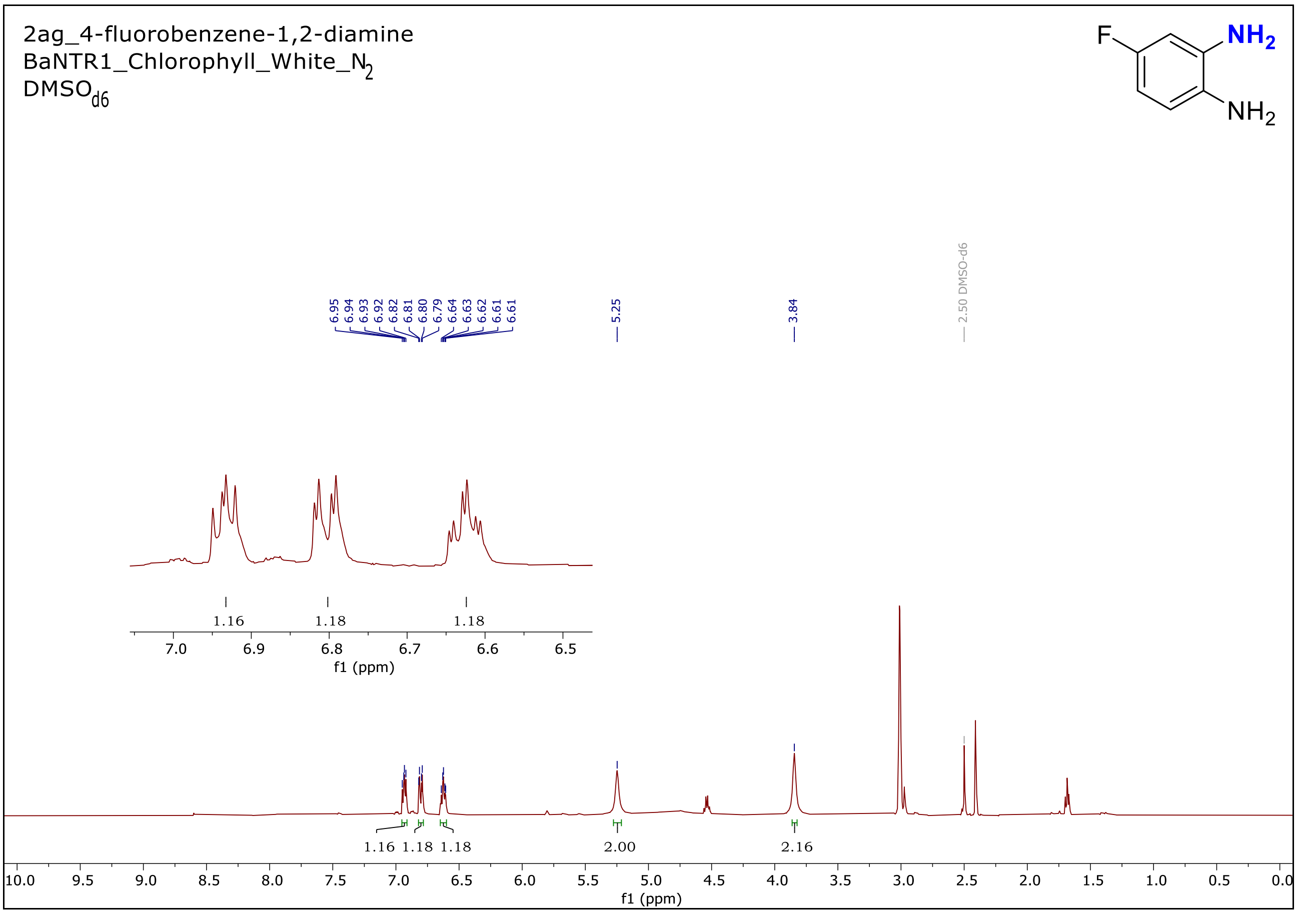

Supplementary Figure 80. ^{1}H-NMR spectrum of the photoenzymatic products **26** (top) and **27** (bottom).

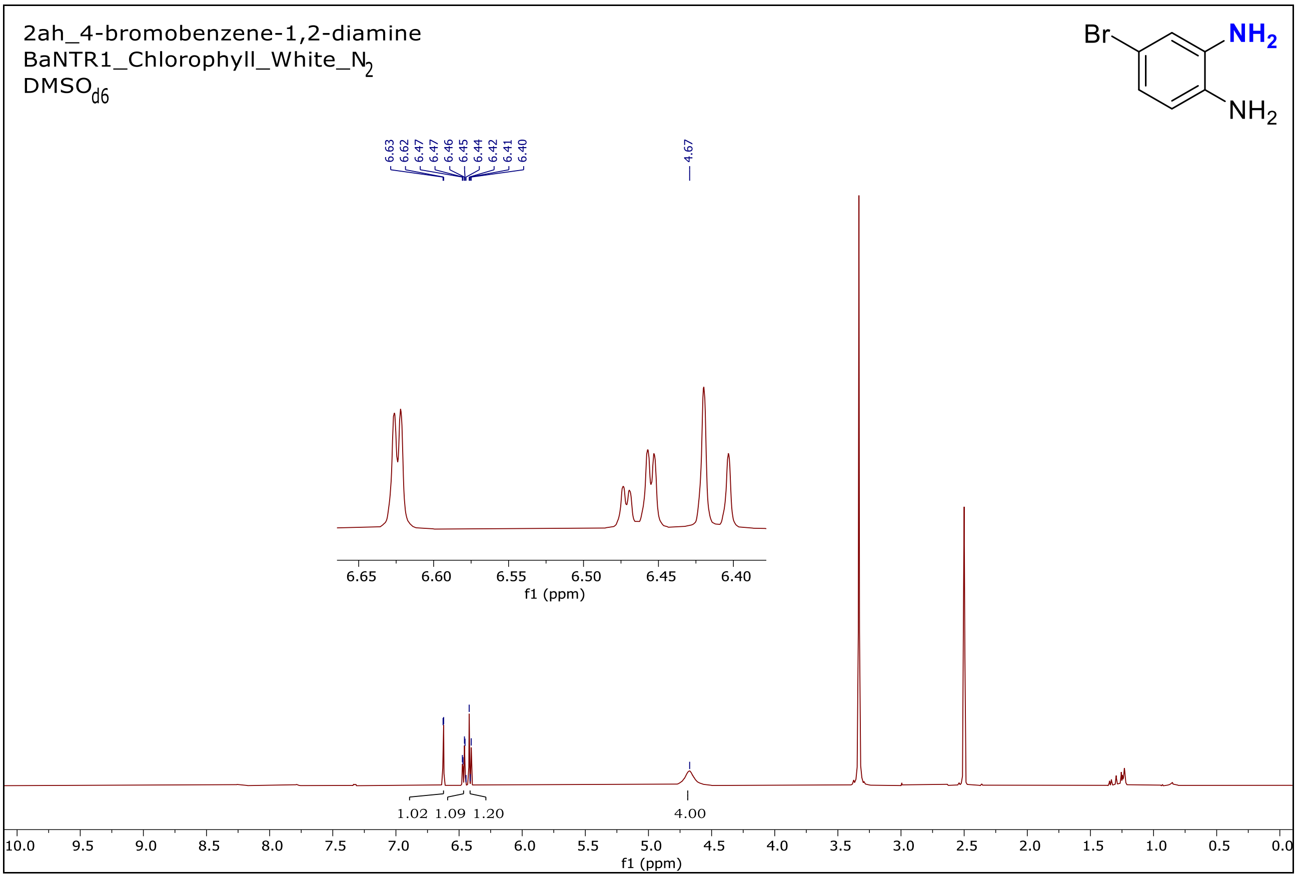

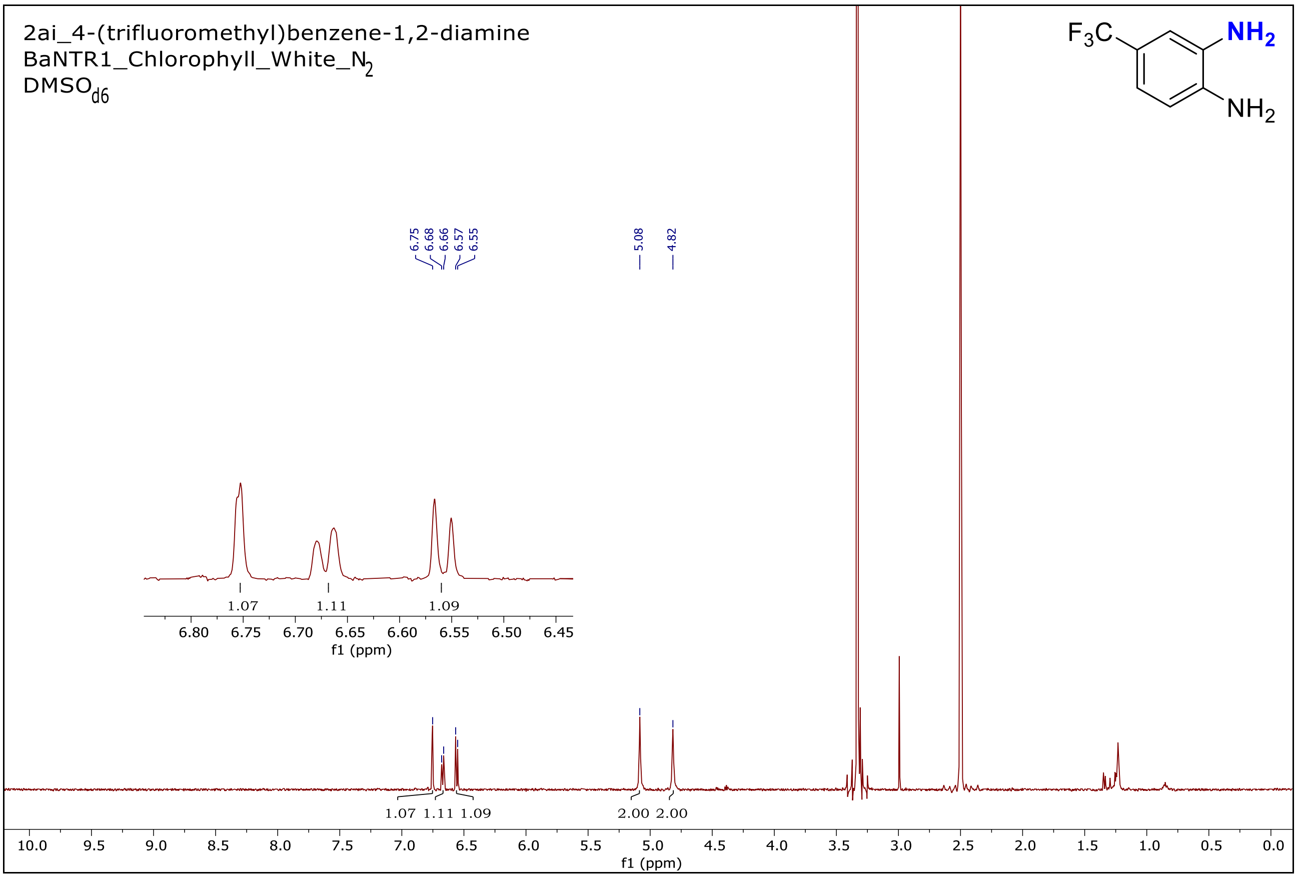

Supplementary Figure 81. ^{1}H-NMR spectrum of the photoenzymatic products **28** (top) and **29** (bottom).

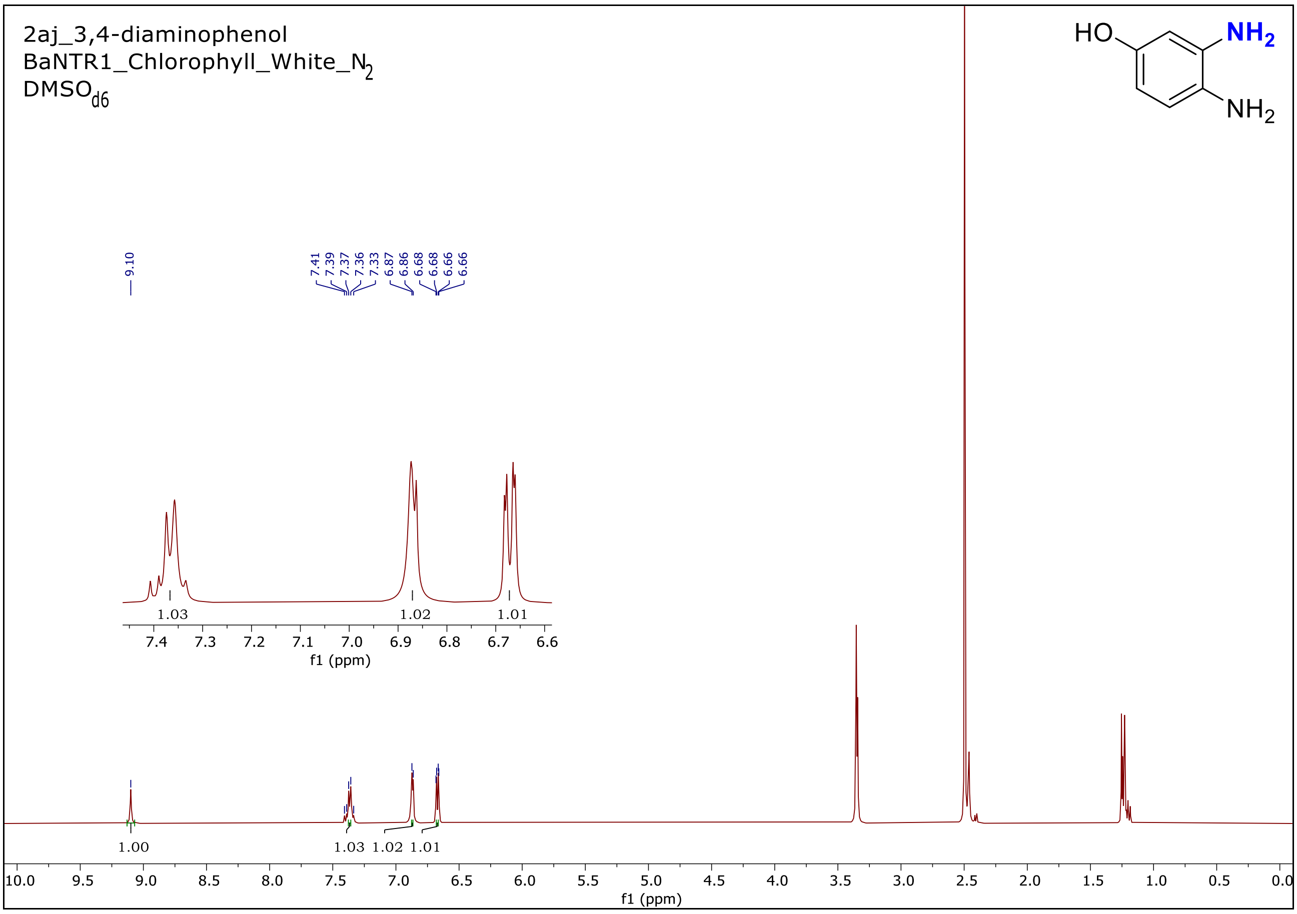

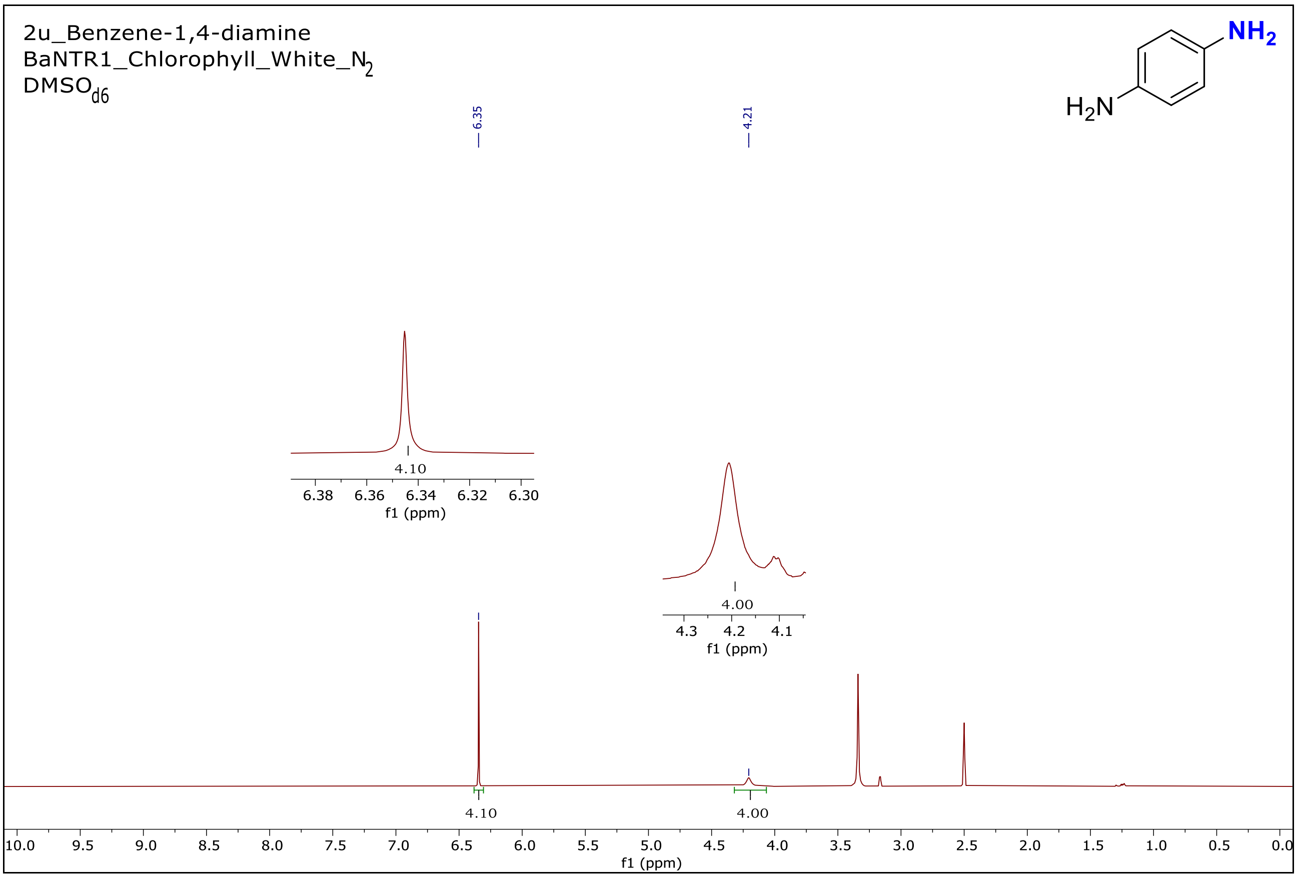

Supplementary Figure 82. ^{1}H-NMR spectrum of the photoenzymatic products **30** (top) and **31** (bottom).

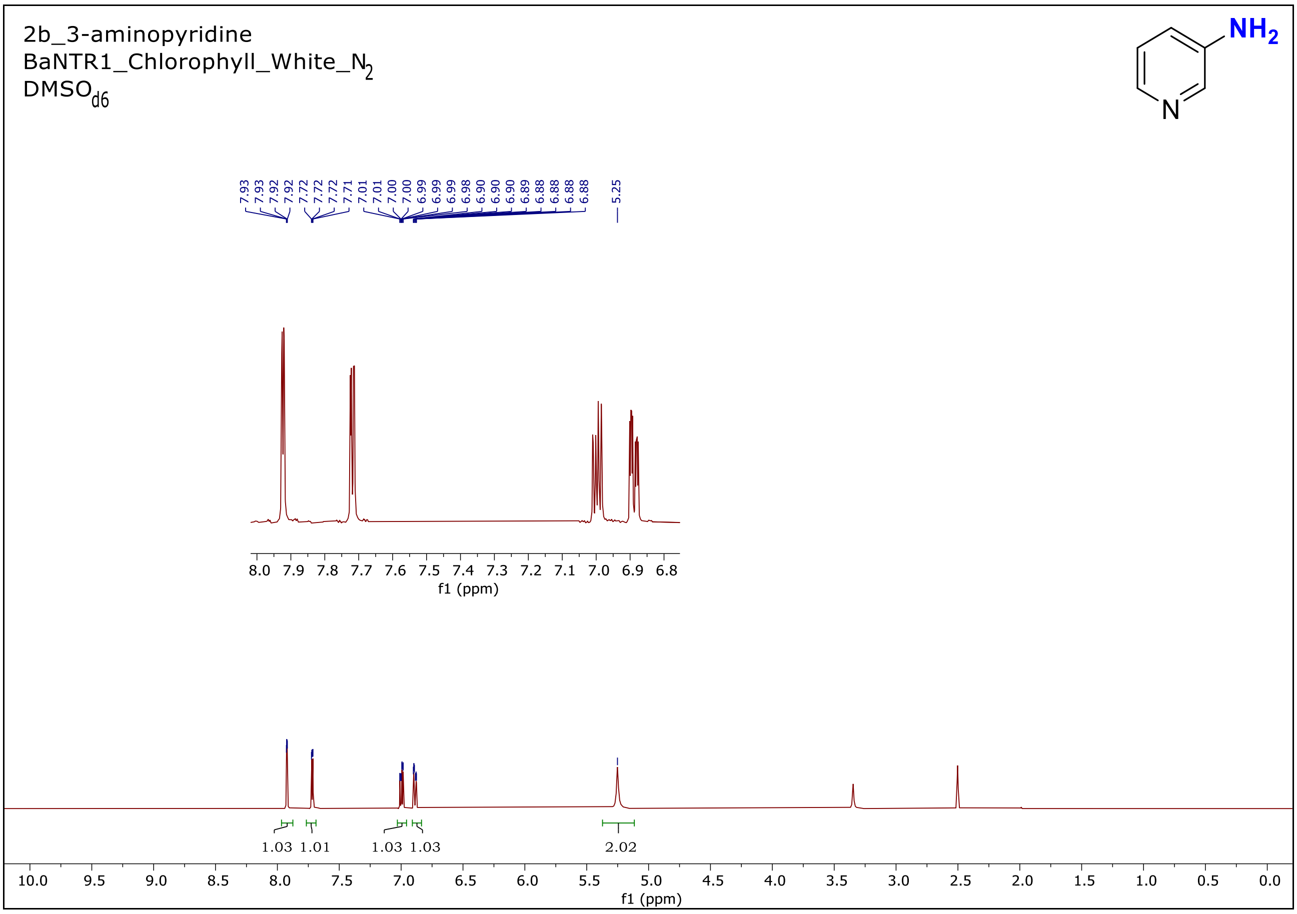

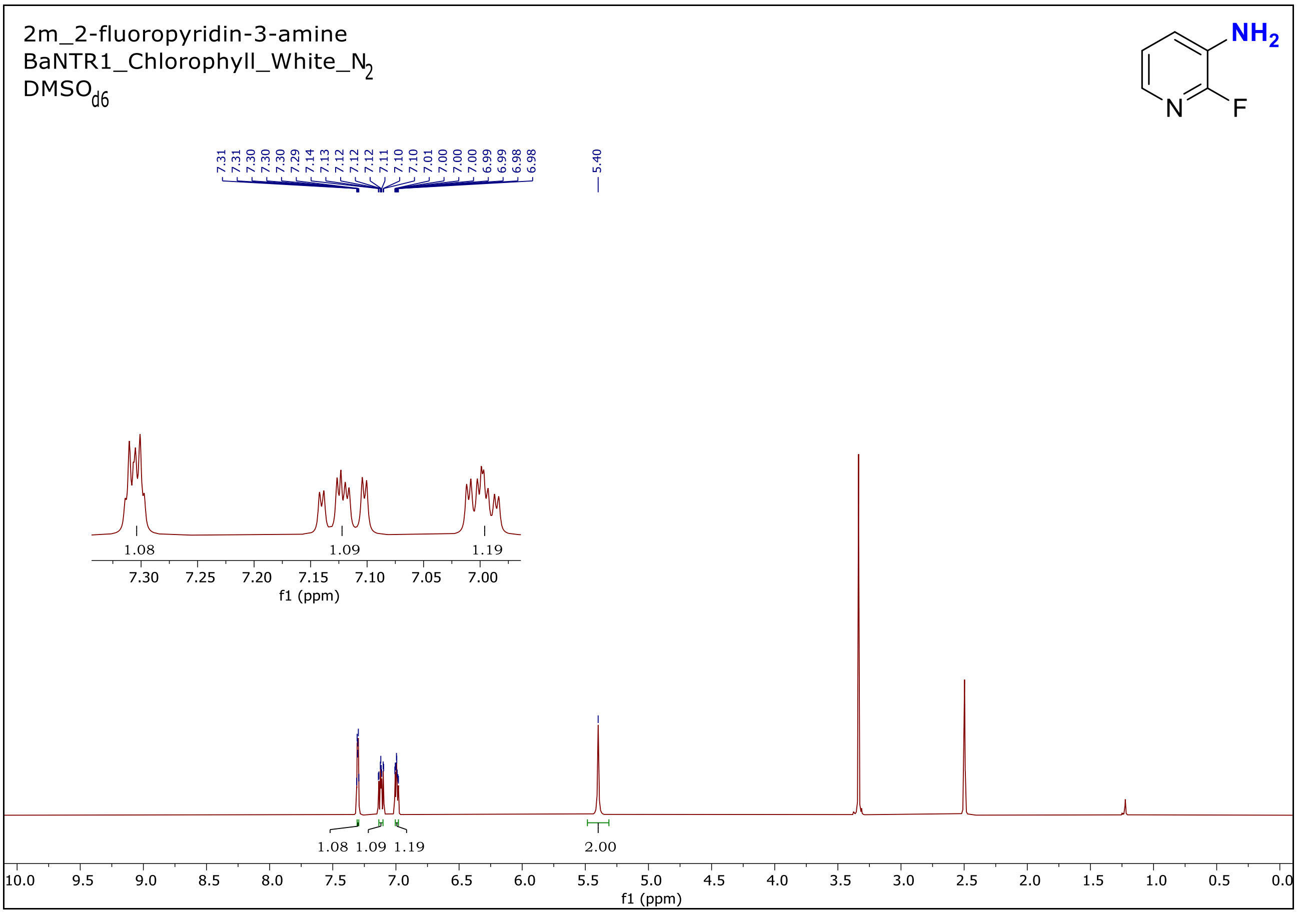

Supplementary Figure 83. ^{1}H-NMR spectrum of the photoenzymatic products **32** (top) and **33** (bottom).

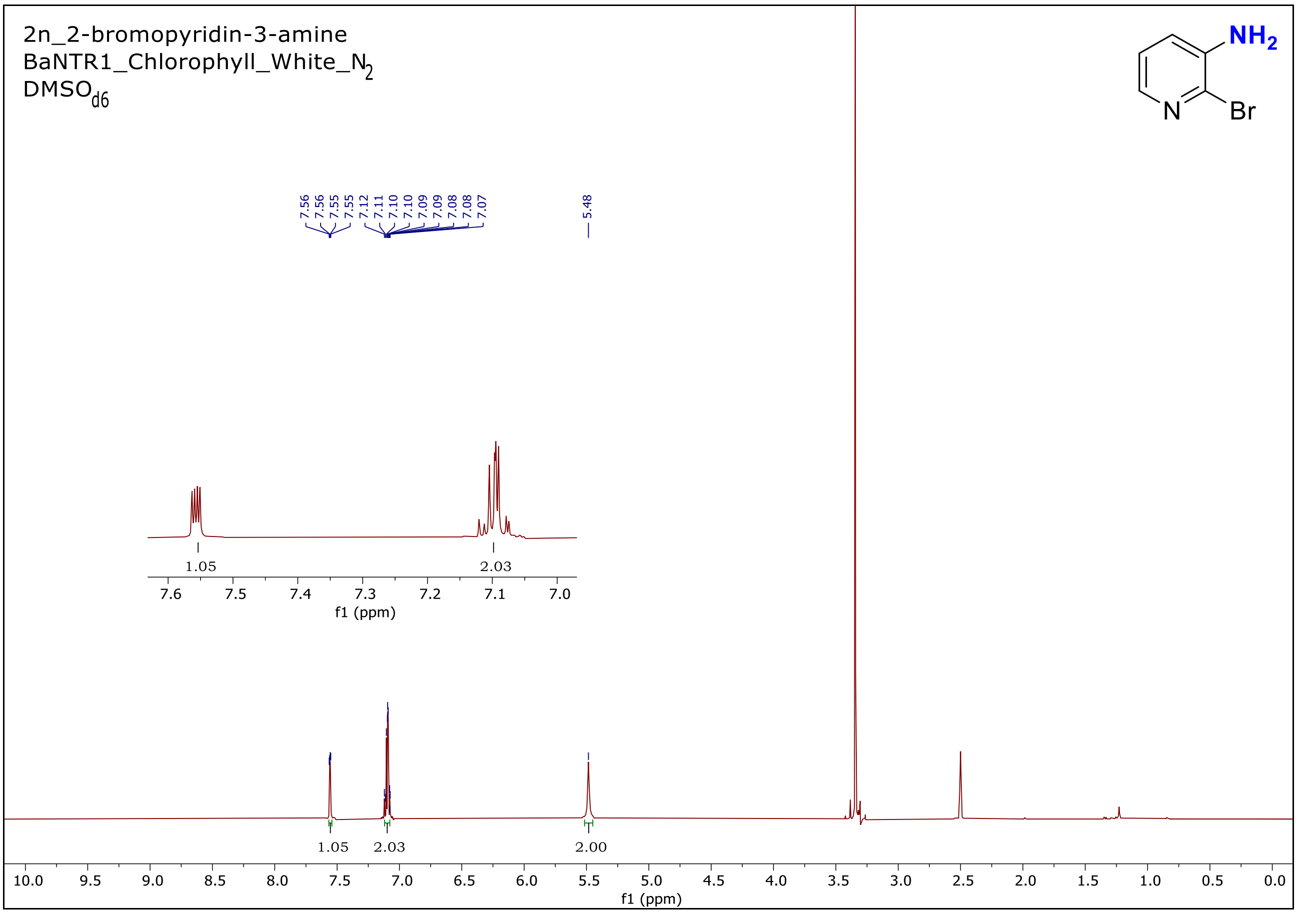

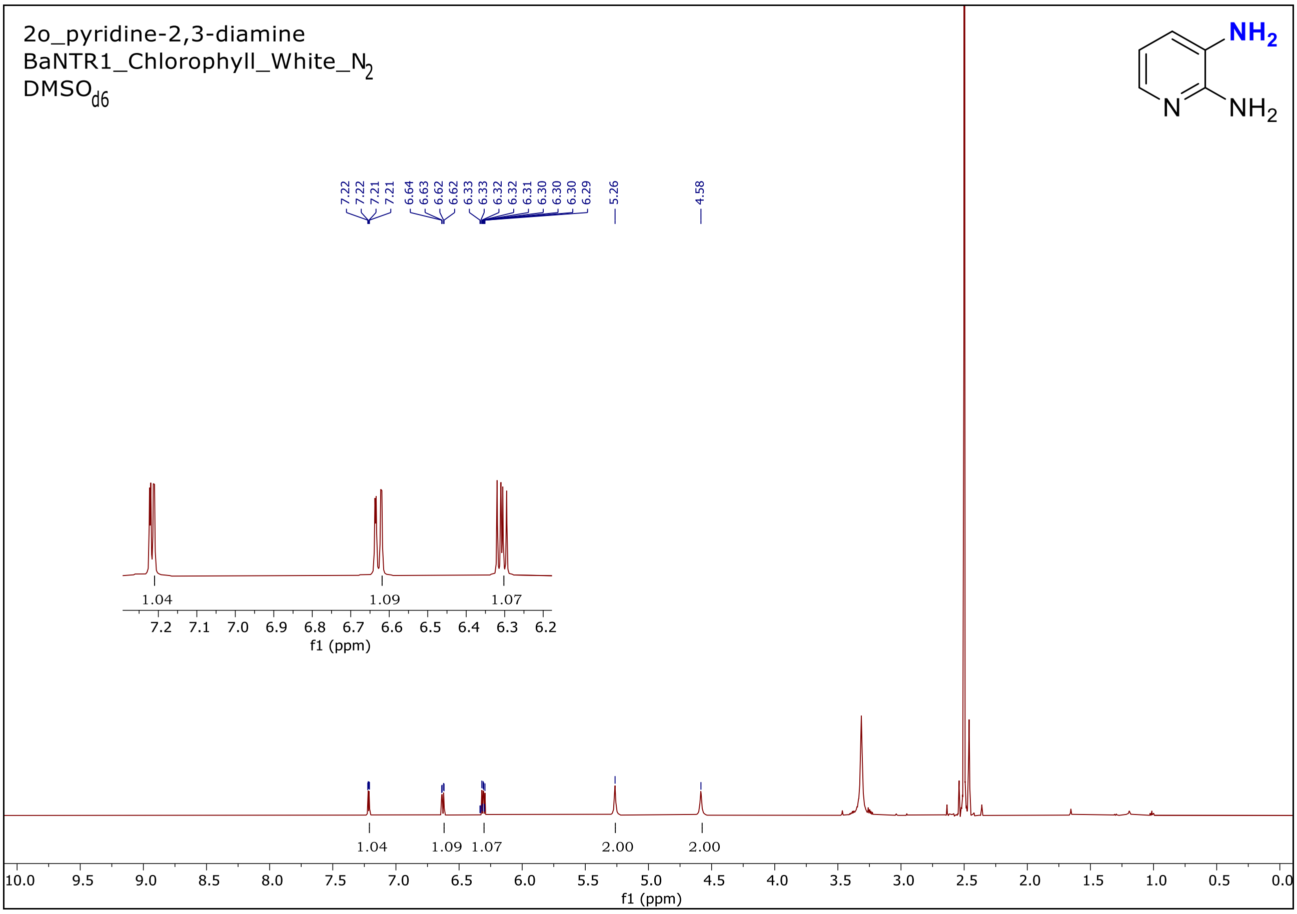

Supplementary Figure 84. ^{1}H-NMR spectrum of the photoenzymatic products **34** (top) and **35** (bottom).

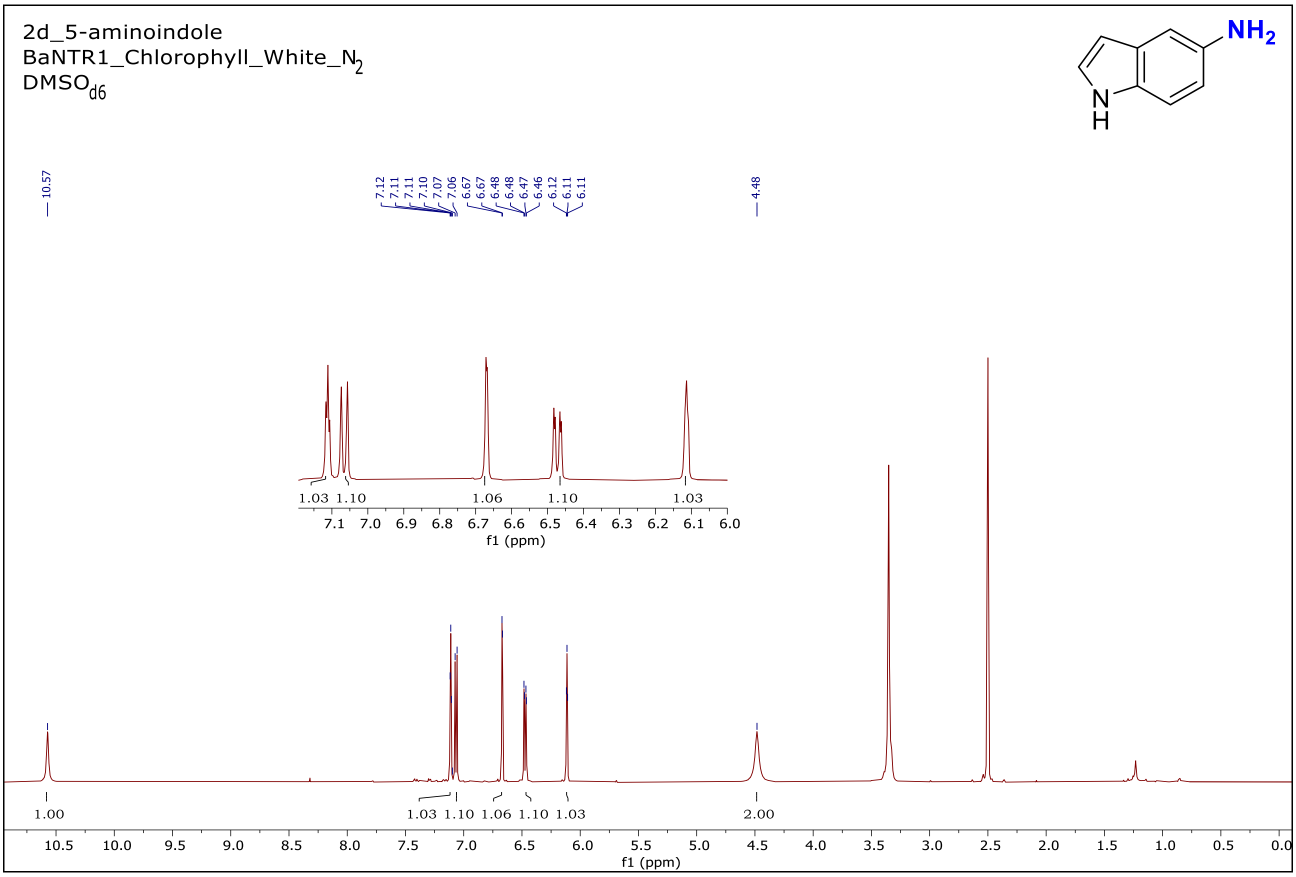

Supplementary Figure 85. ^{1}H-NMR spectrum of the photoenzymatic product **36.**

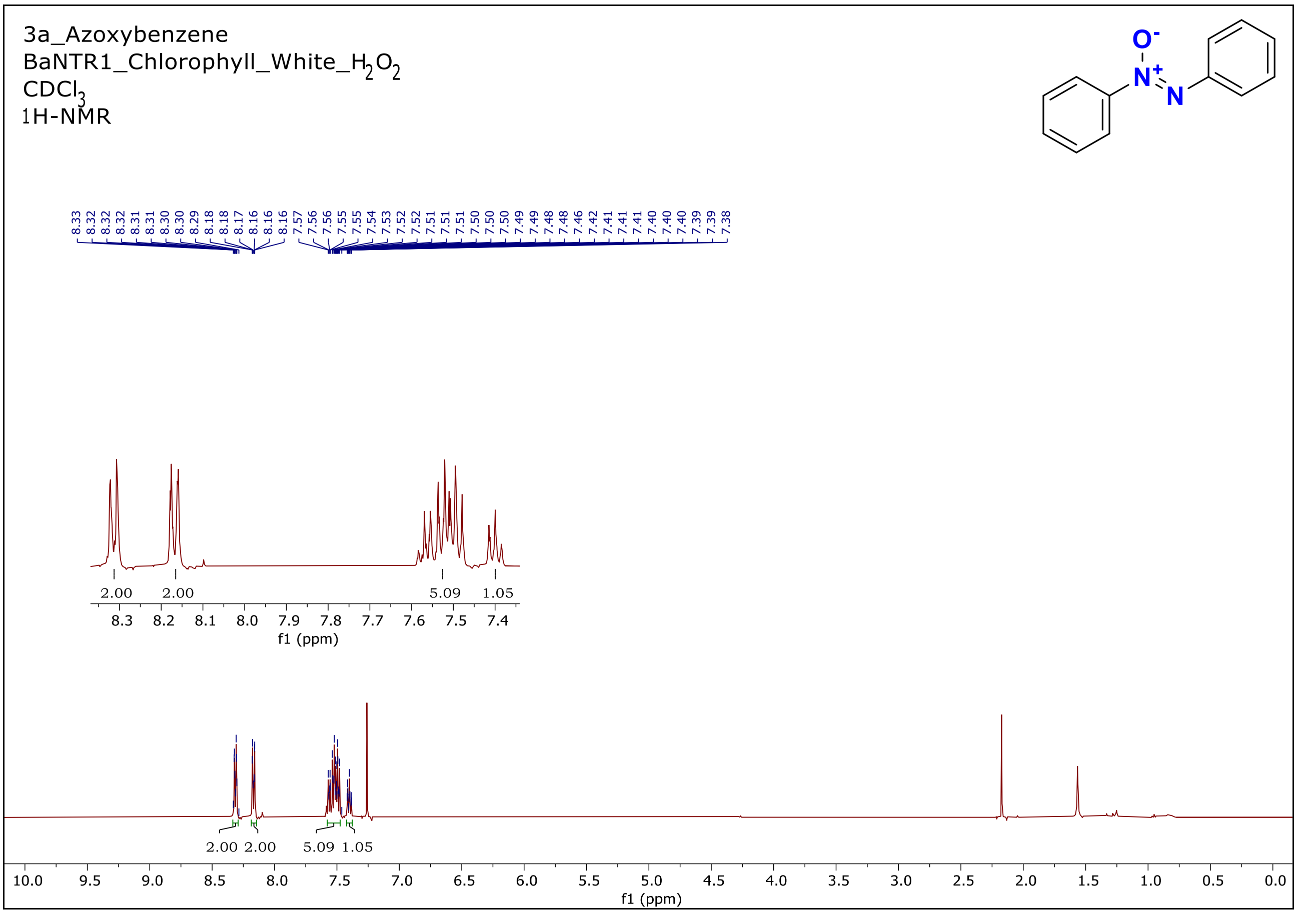

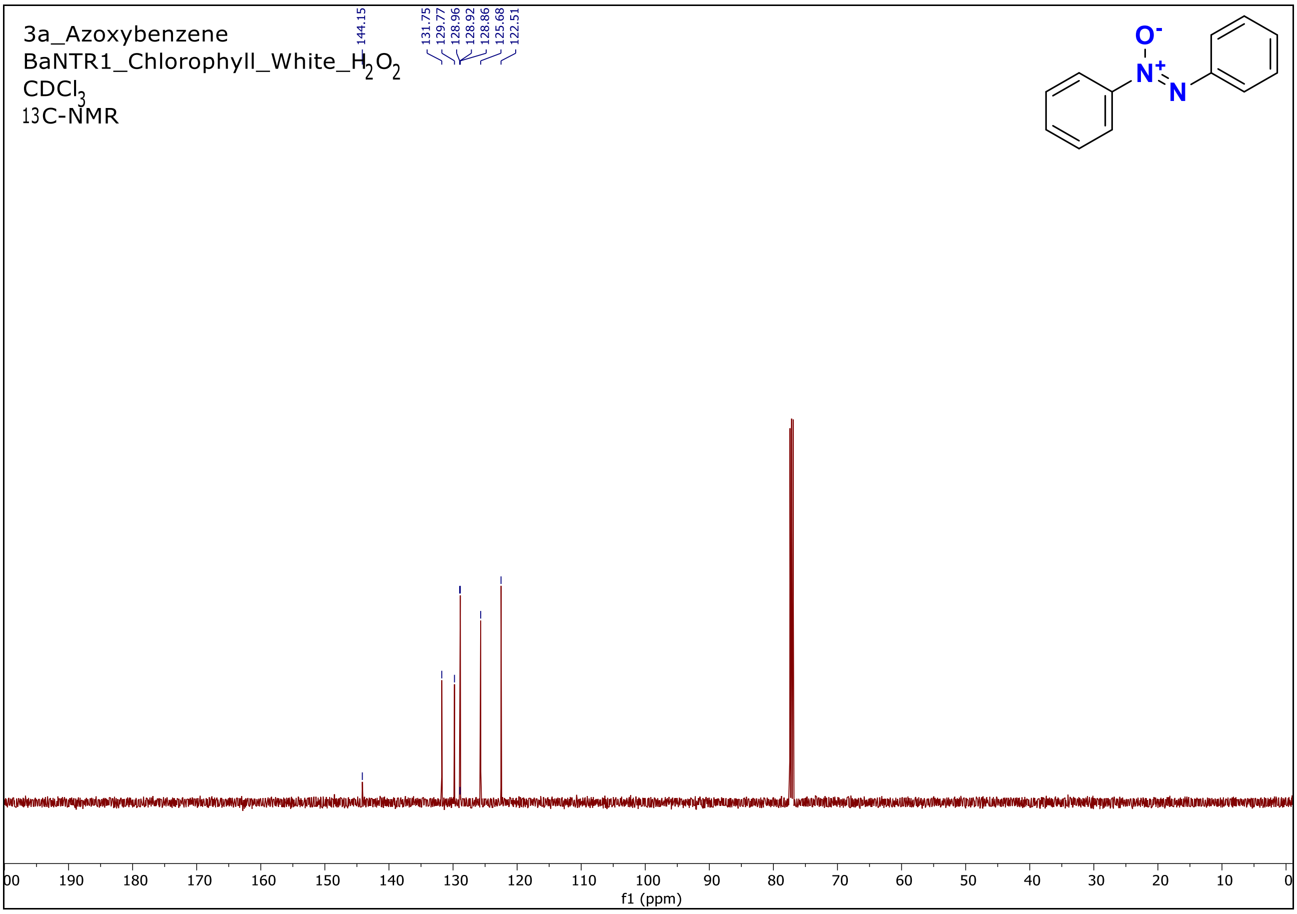

Supplementary Figure 86. ^{1}H-NMR (top) and ^{13}C-NMR (bottom) spectrum of the photoenzymatic product **37**.

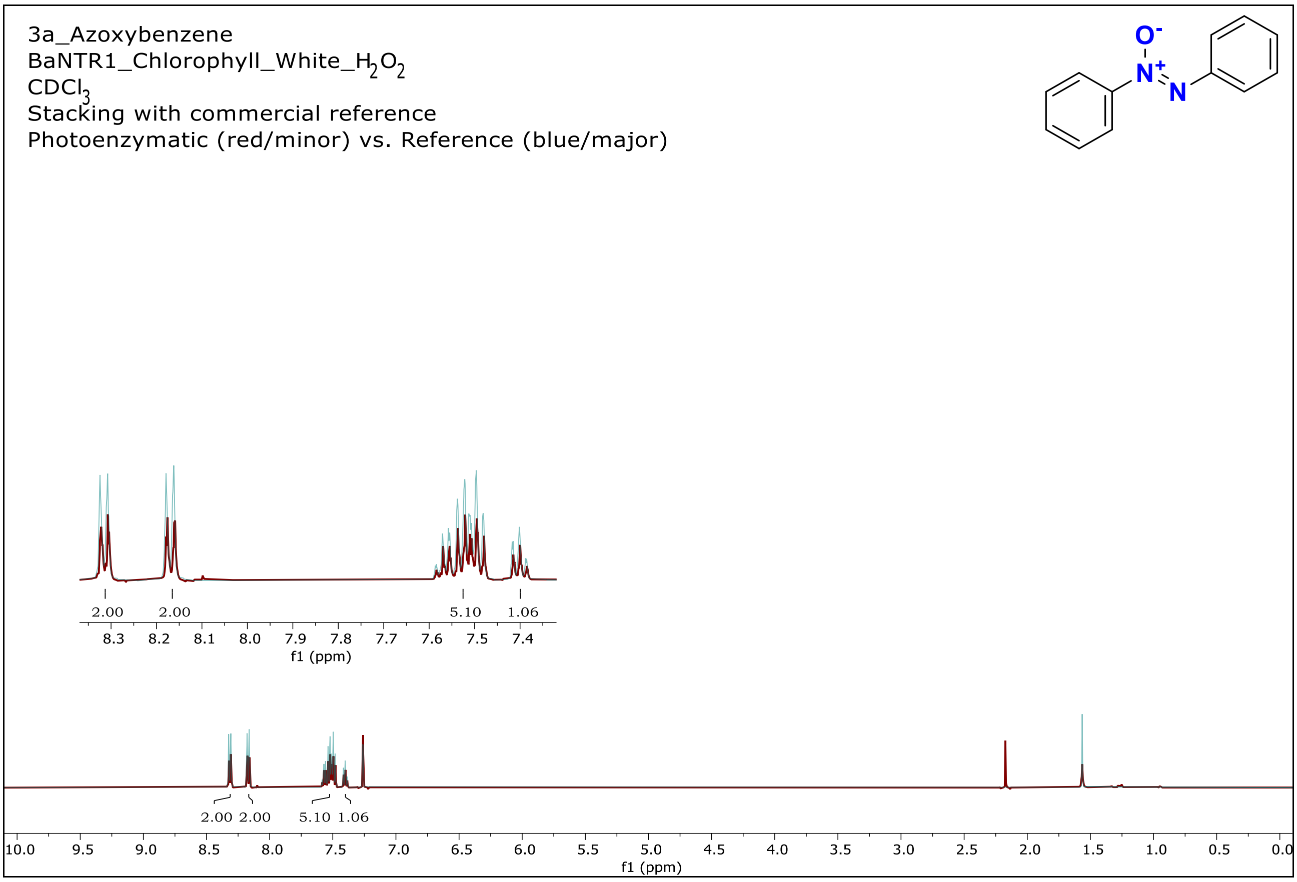

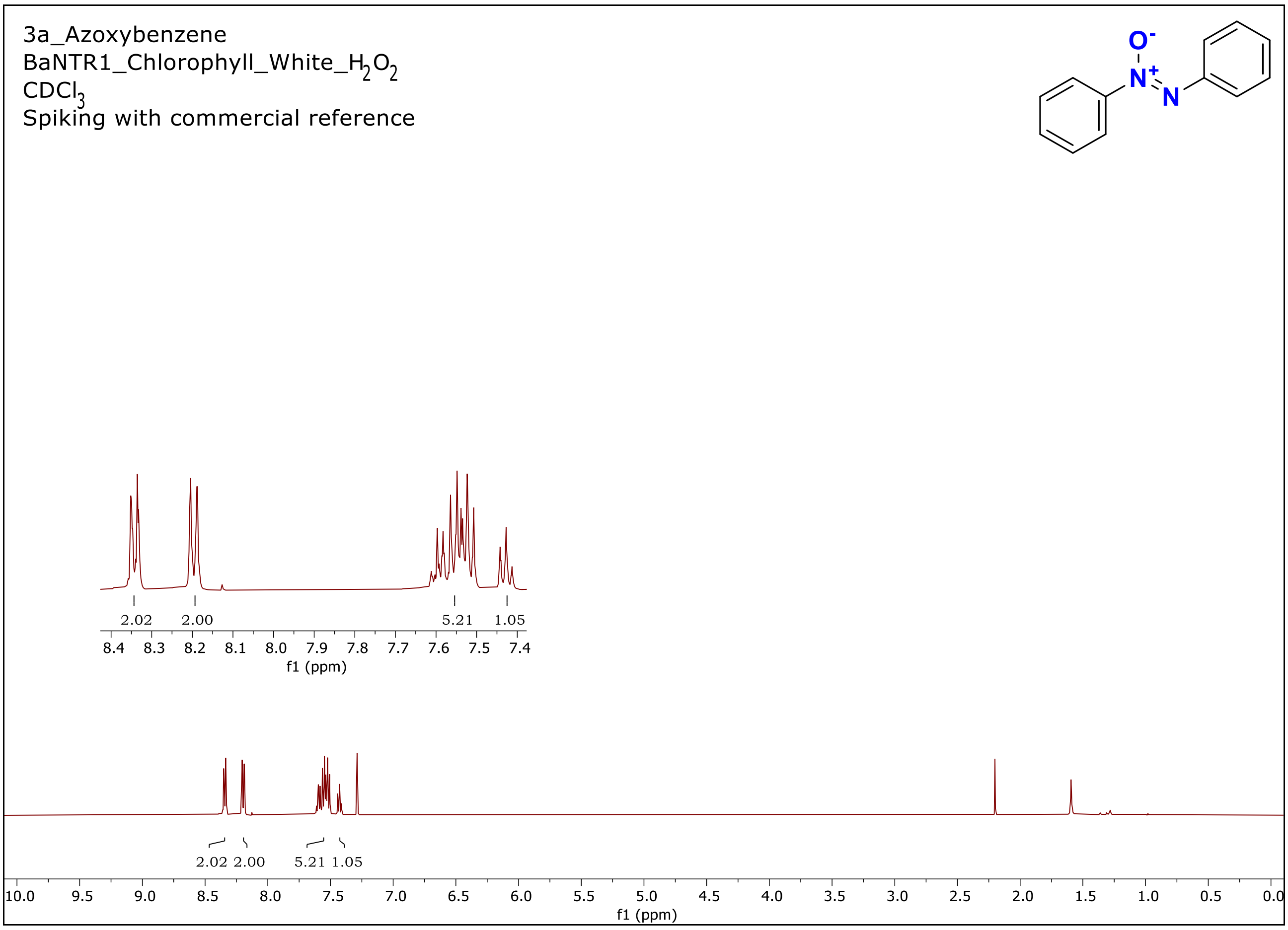

Supplementary Figure 87. Stacking (top) and spiking (bottom) ^{1}H-NMR spectrum of the photoenzymatic product **37** with a commercial reference.

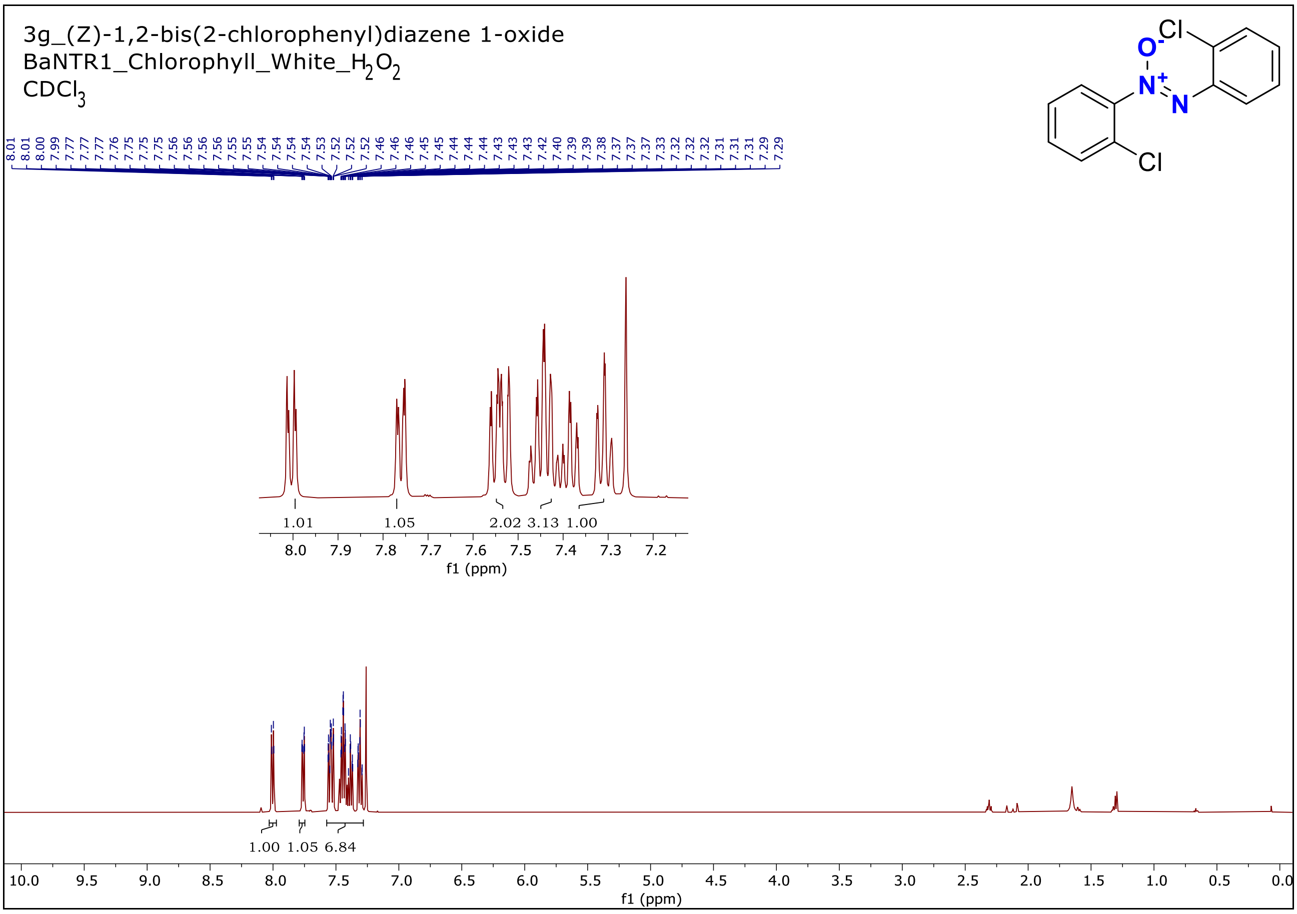

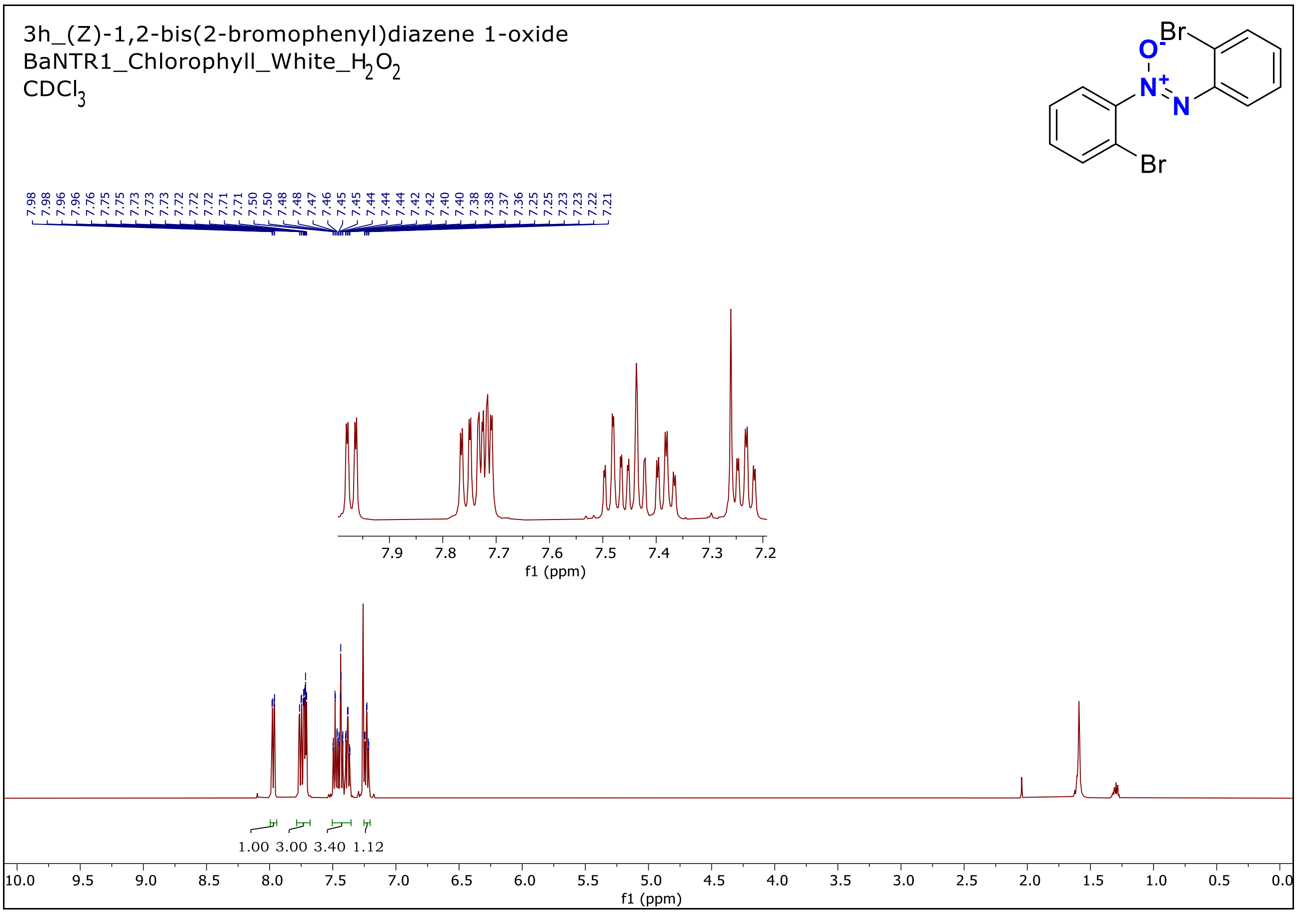

Supplementary Figure 88. ^{1}H-NMR spectrum of the photoenzymatic products **38** (top) and **39** (bottom).

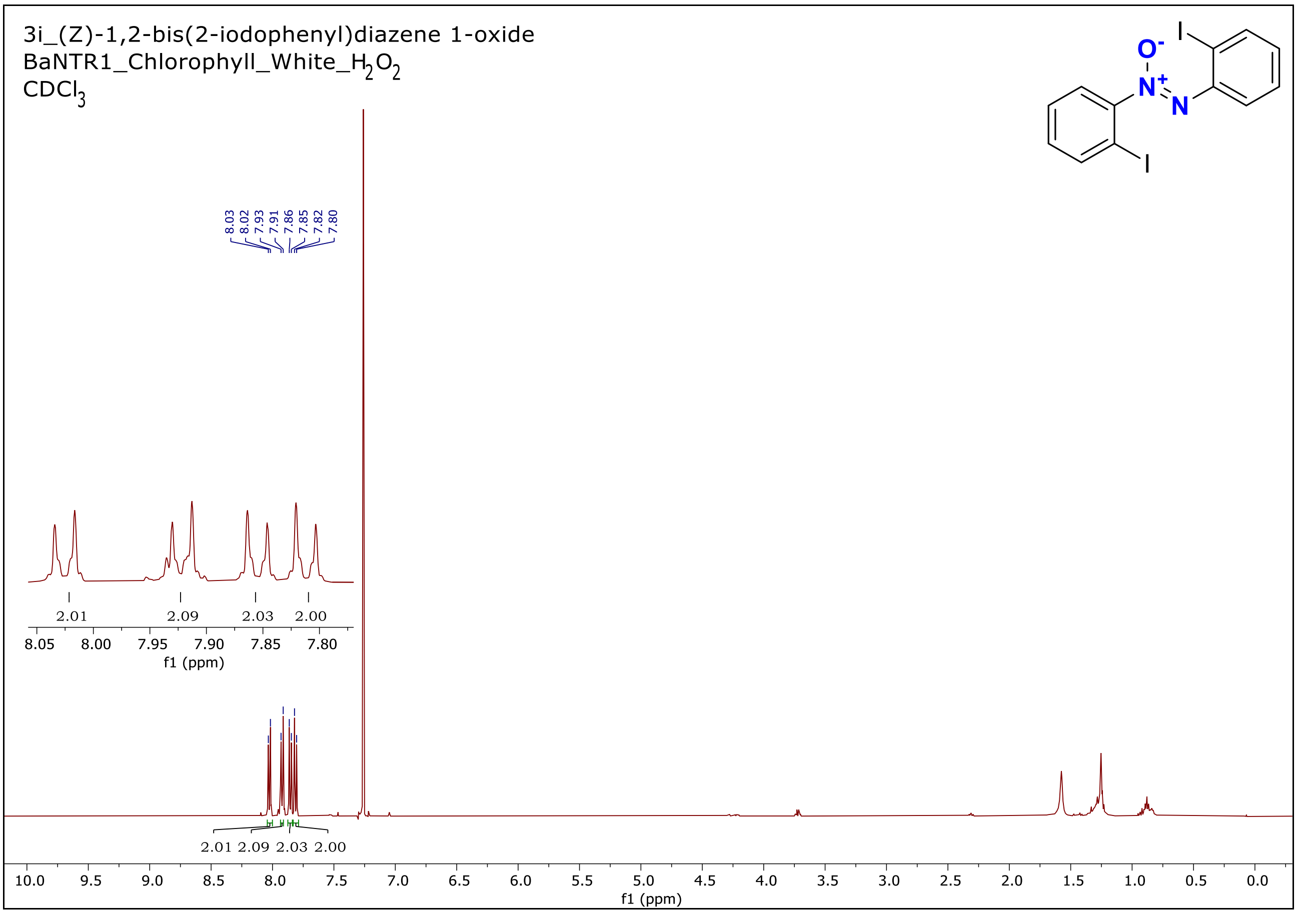

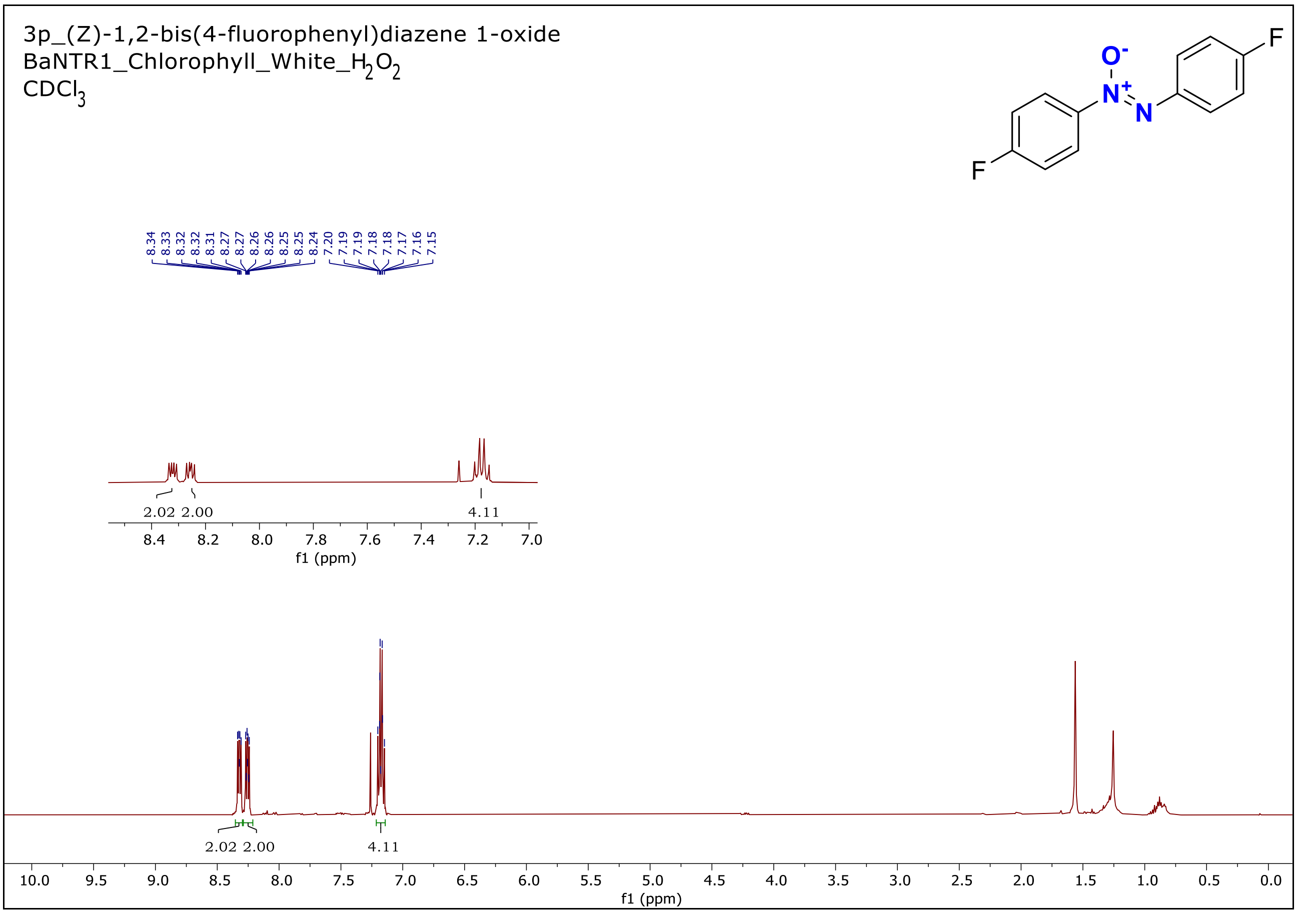

Supplementary Figure 89. ^{1}H-NMR spectrum of the photoenzymatic products **40** (top) and **41** (bottom).

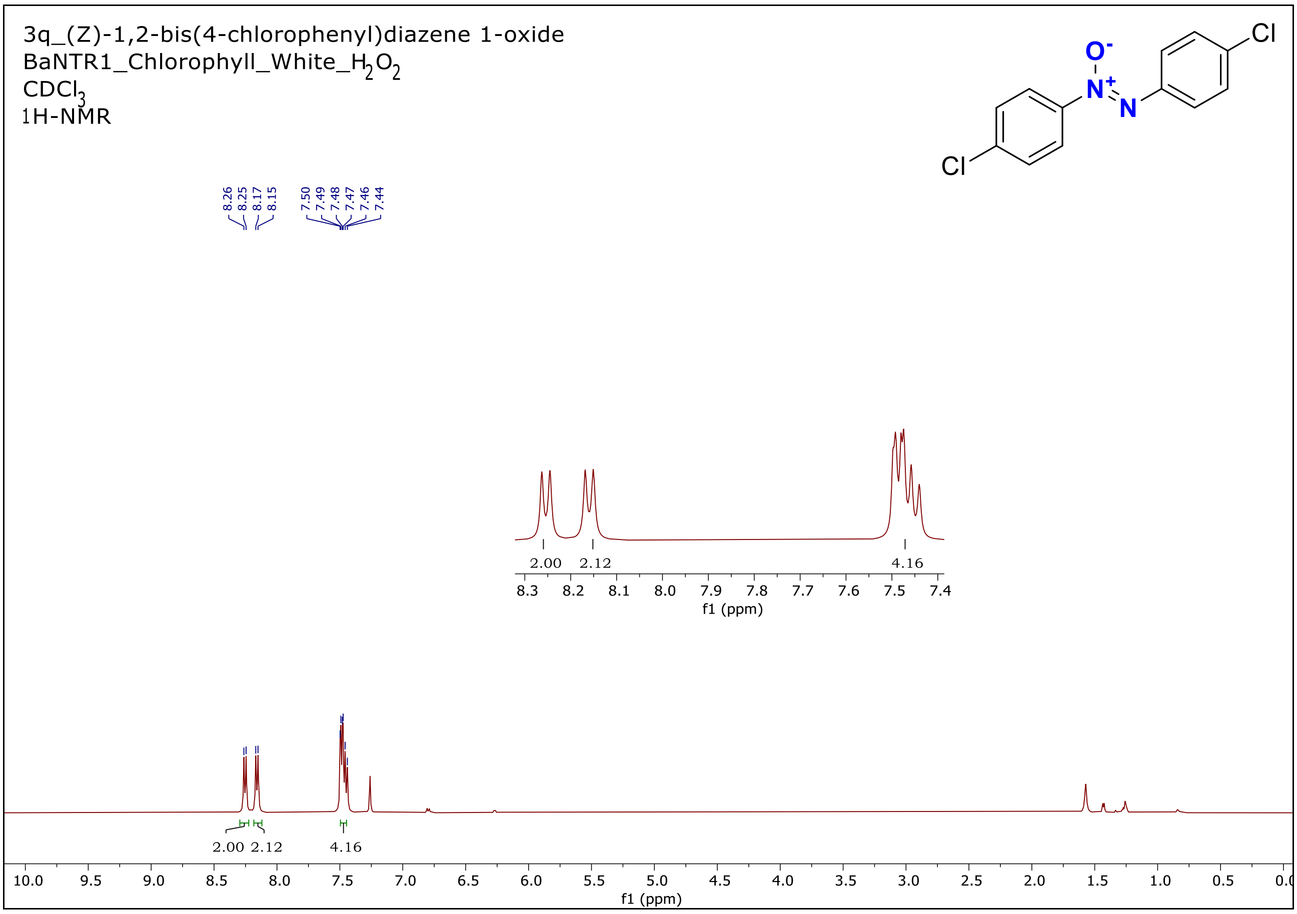

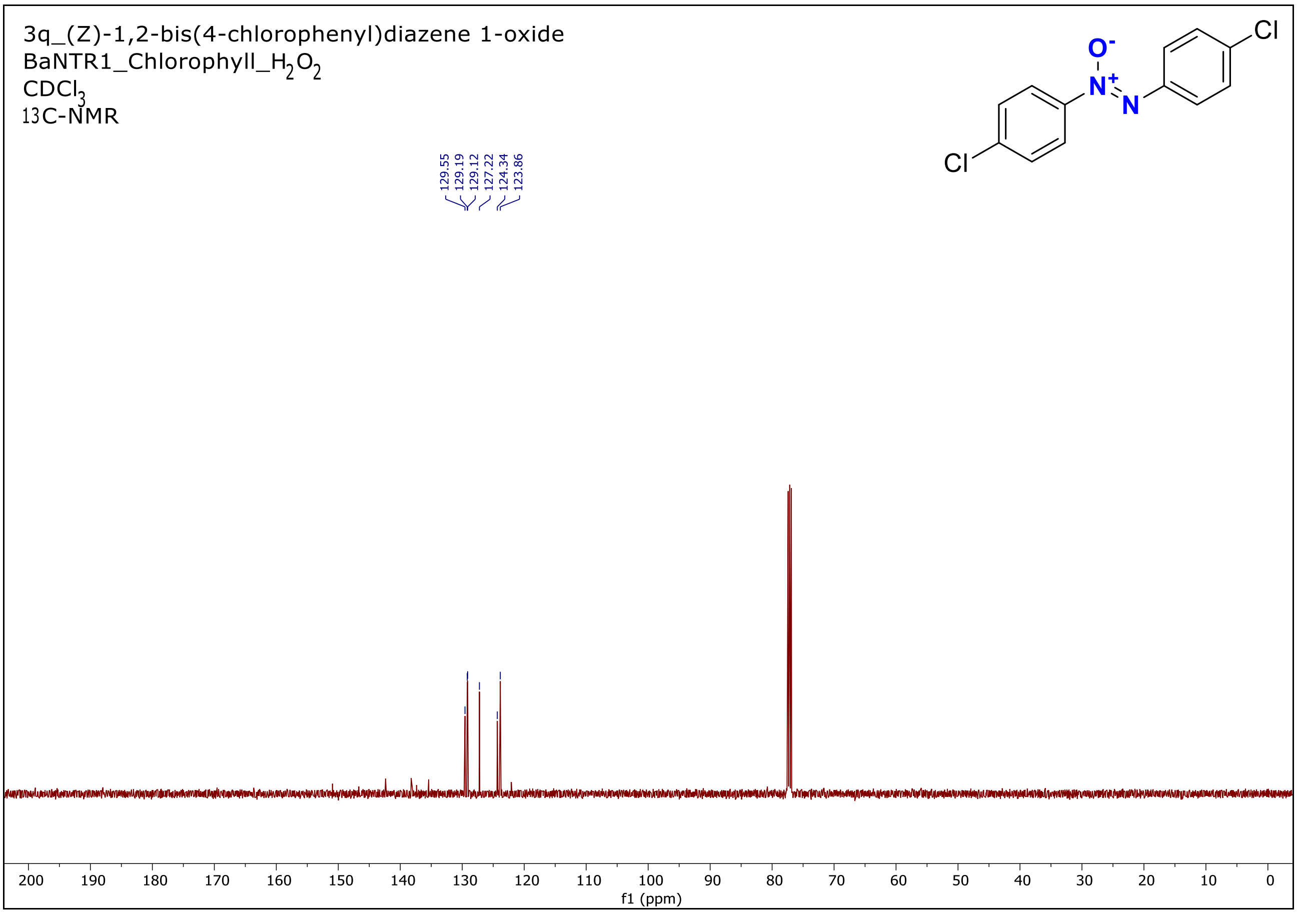

Supplementary Figure 90. ^{1}H-NMR (top) and ^{13}C-NMR (bottom) spectrum of the photoenzymatic product **42**.

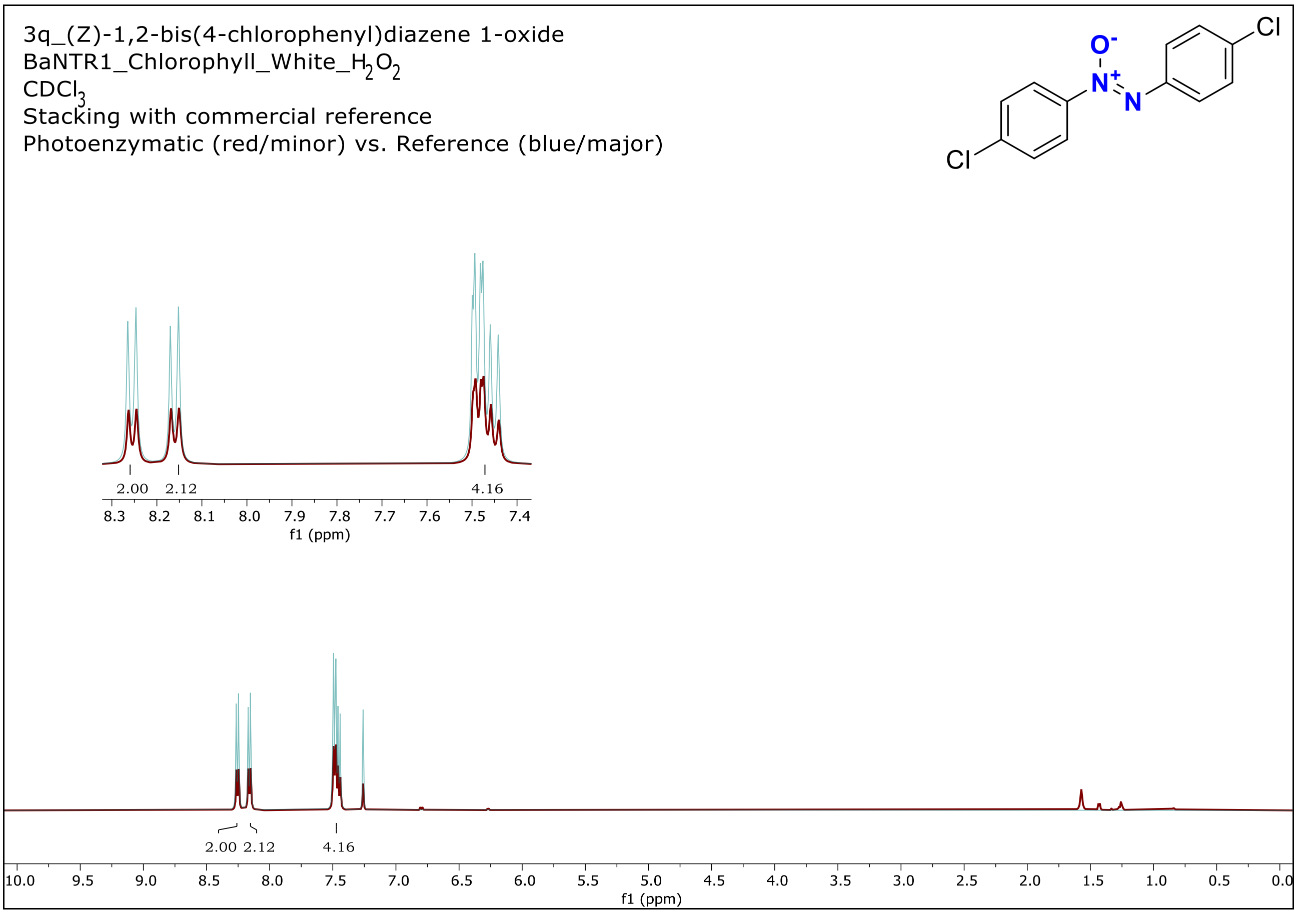

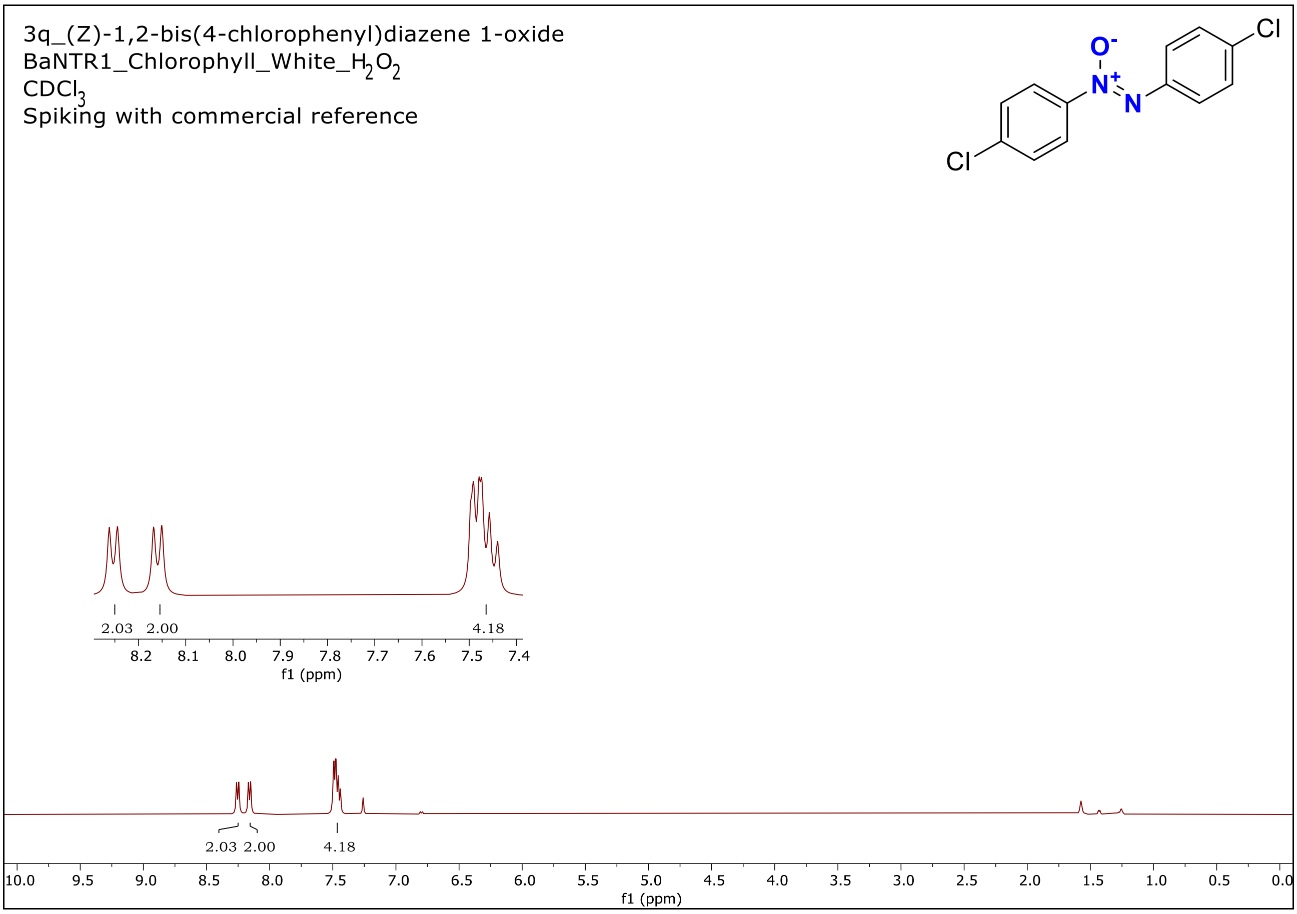

Supplementary Figure 91. Stacking (top) and spiking (bottom) ^{1}H-NMR of the photoenzymatic product **42** with a commercial reference.

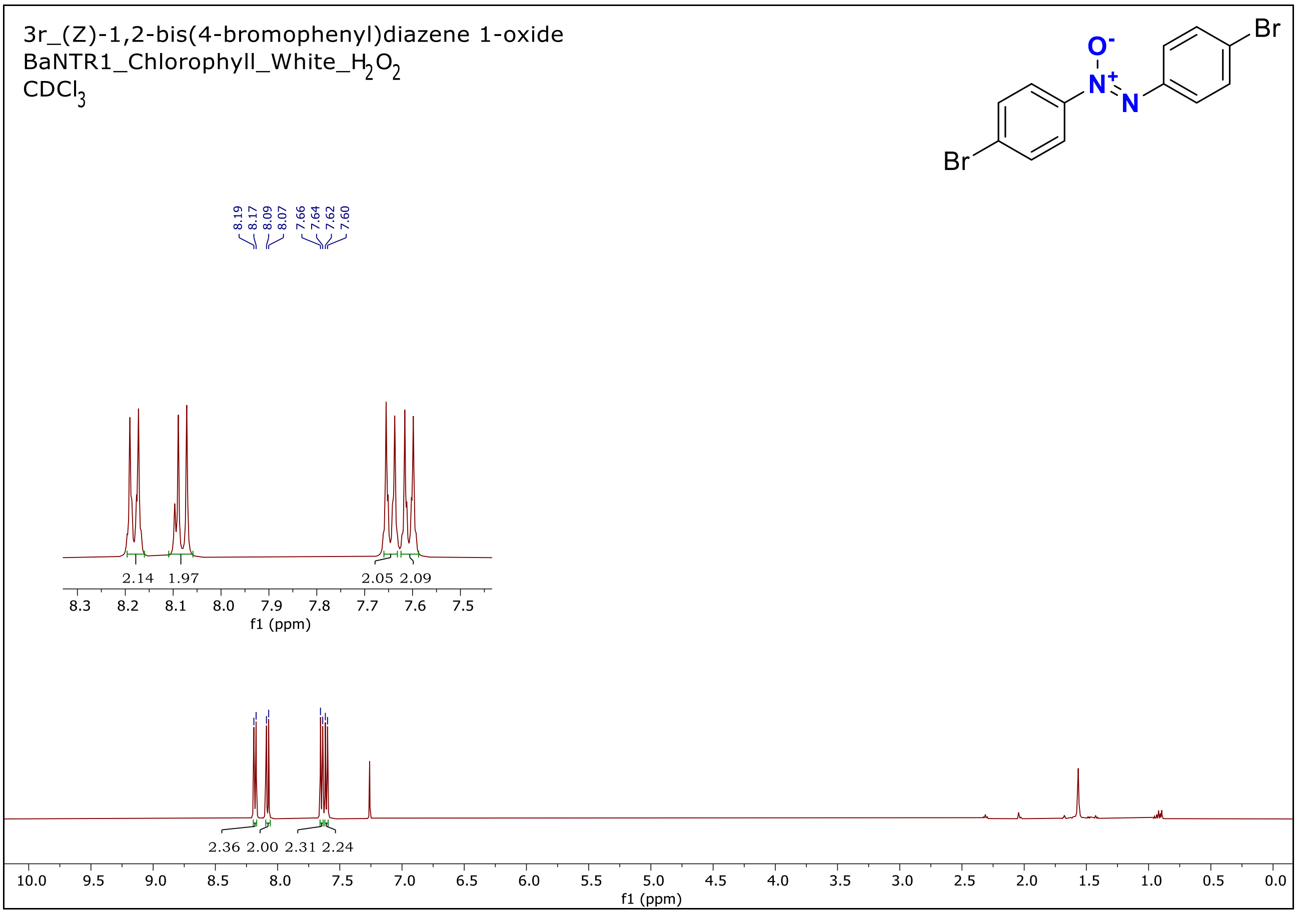

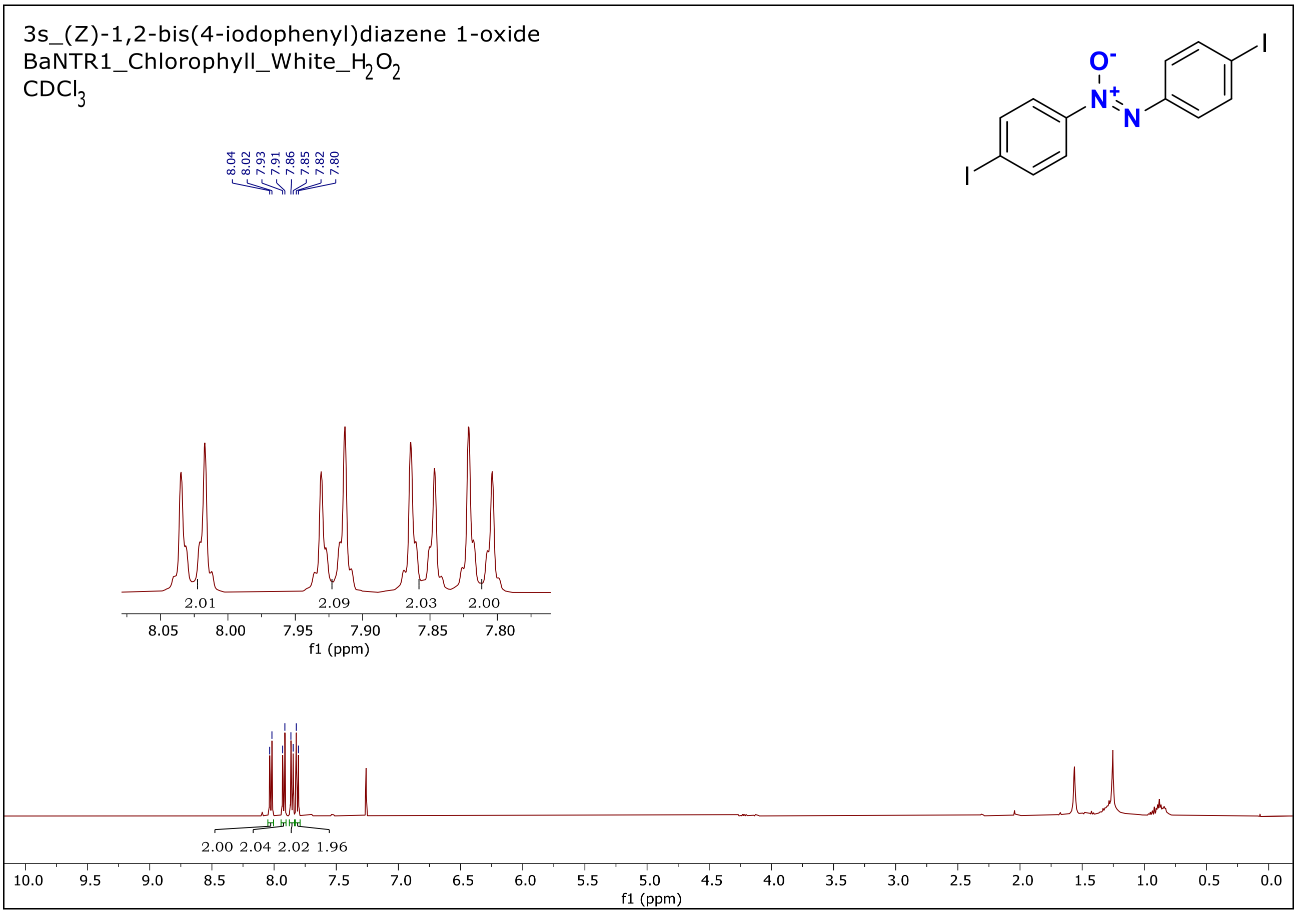

Supplementary Figure 92. ^{1}H-NMR spectrum of the photoenzymatic products **43** (top) and **44** (bottom).

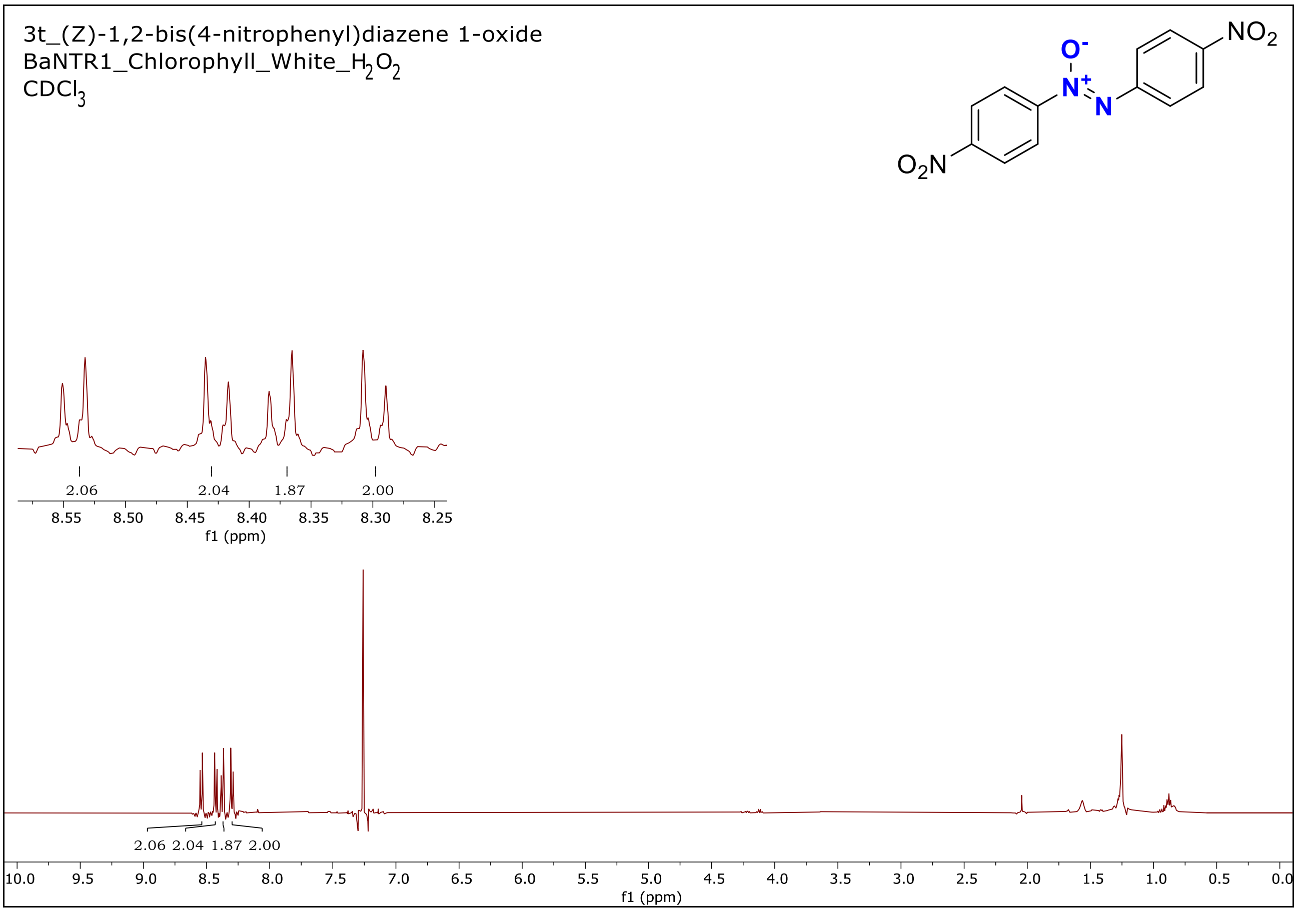

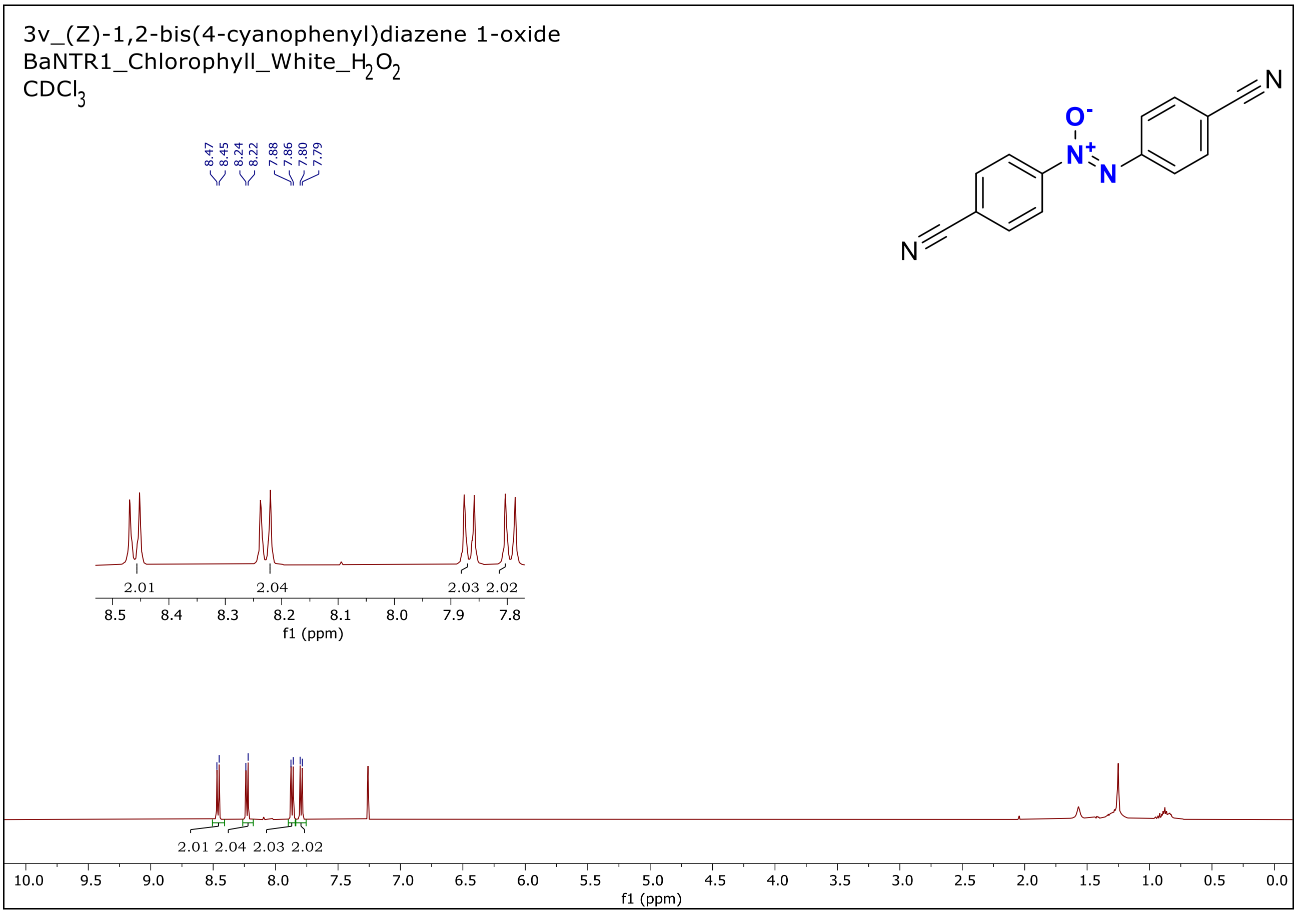

Supplementary Figure 93. ^{1}H-NMR spectrum of the photoenzymatic products **45** (top) and **46** (bottom).

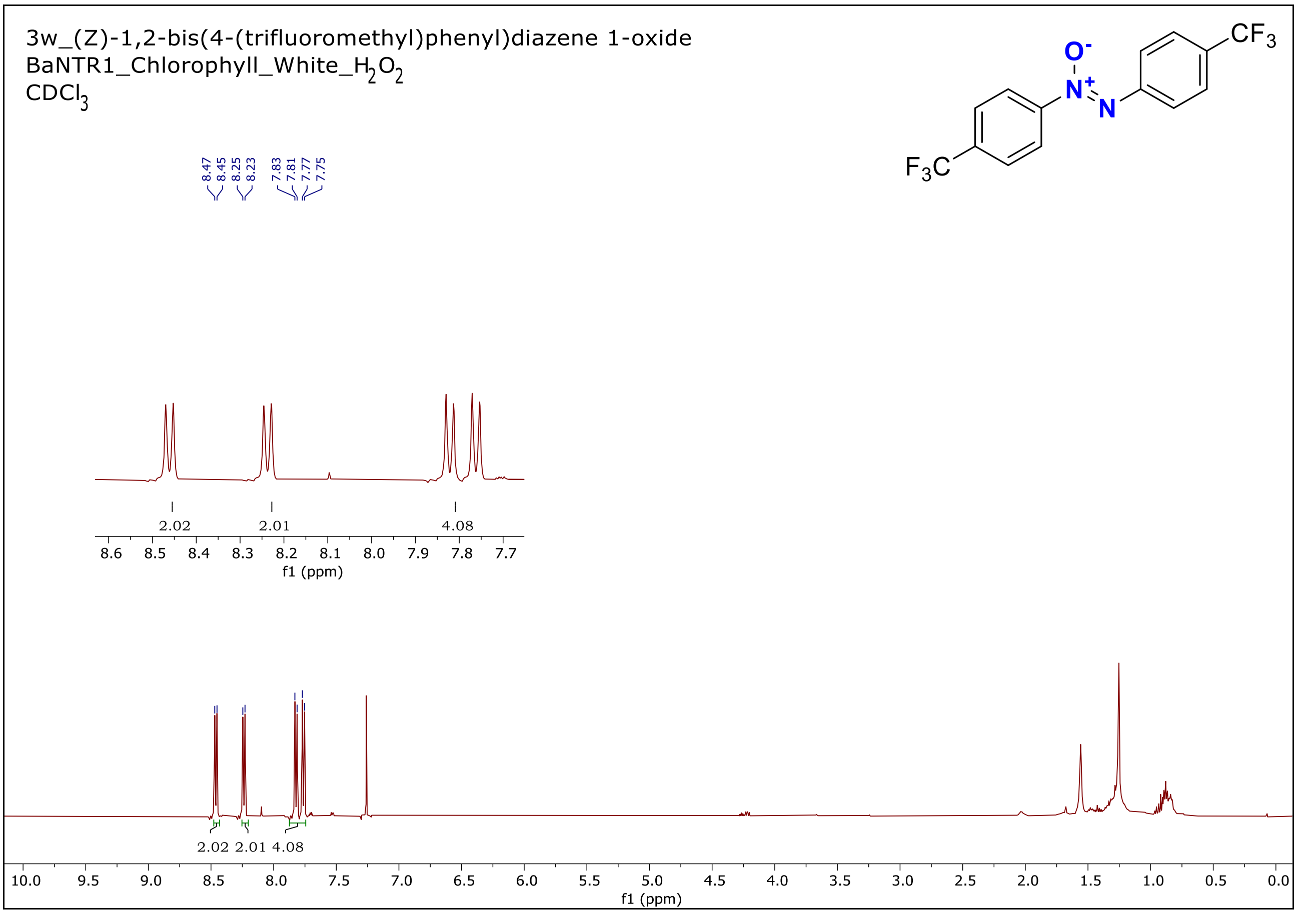

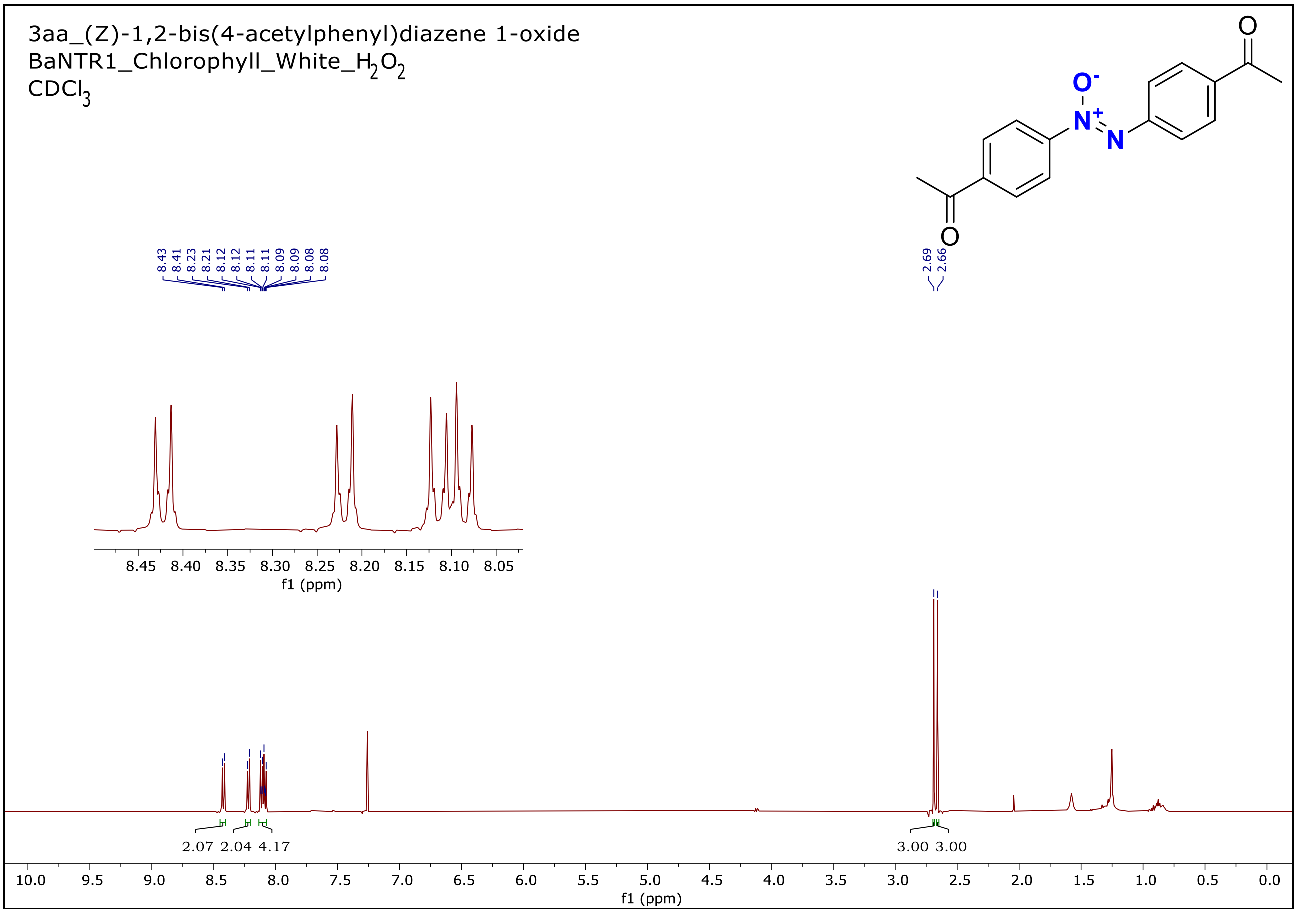

Supplementary Figure 94. ^{1}H-NMR spectrum of the photoenzymatic products **47** (top) and **48** (bottom).

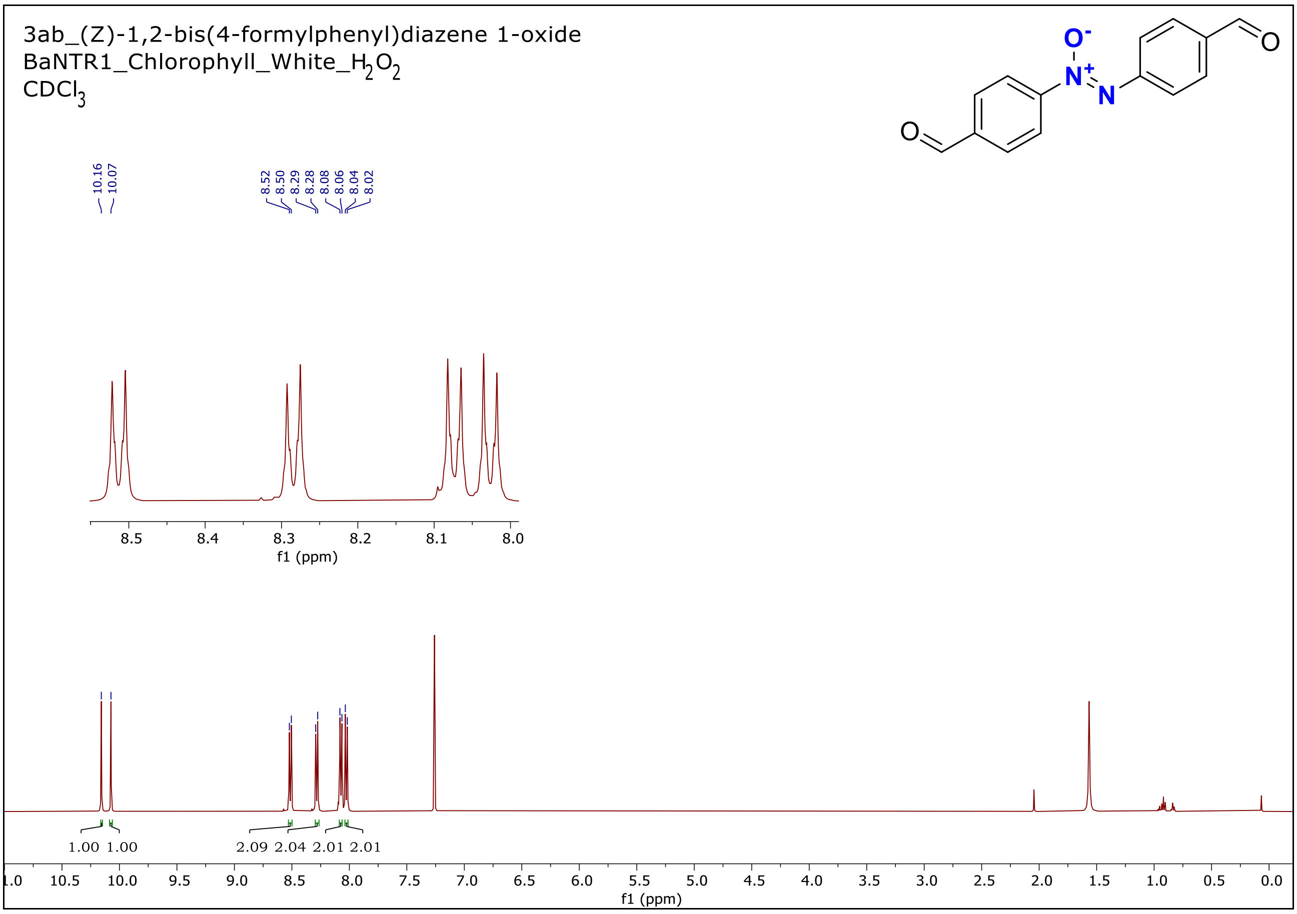

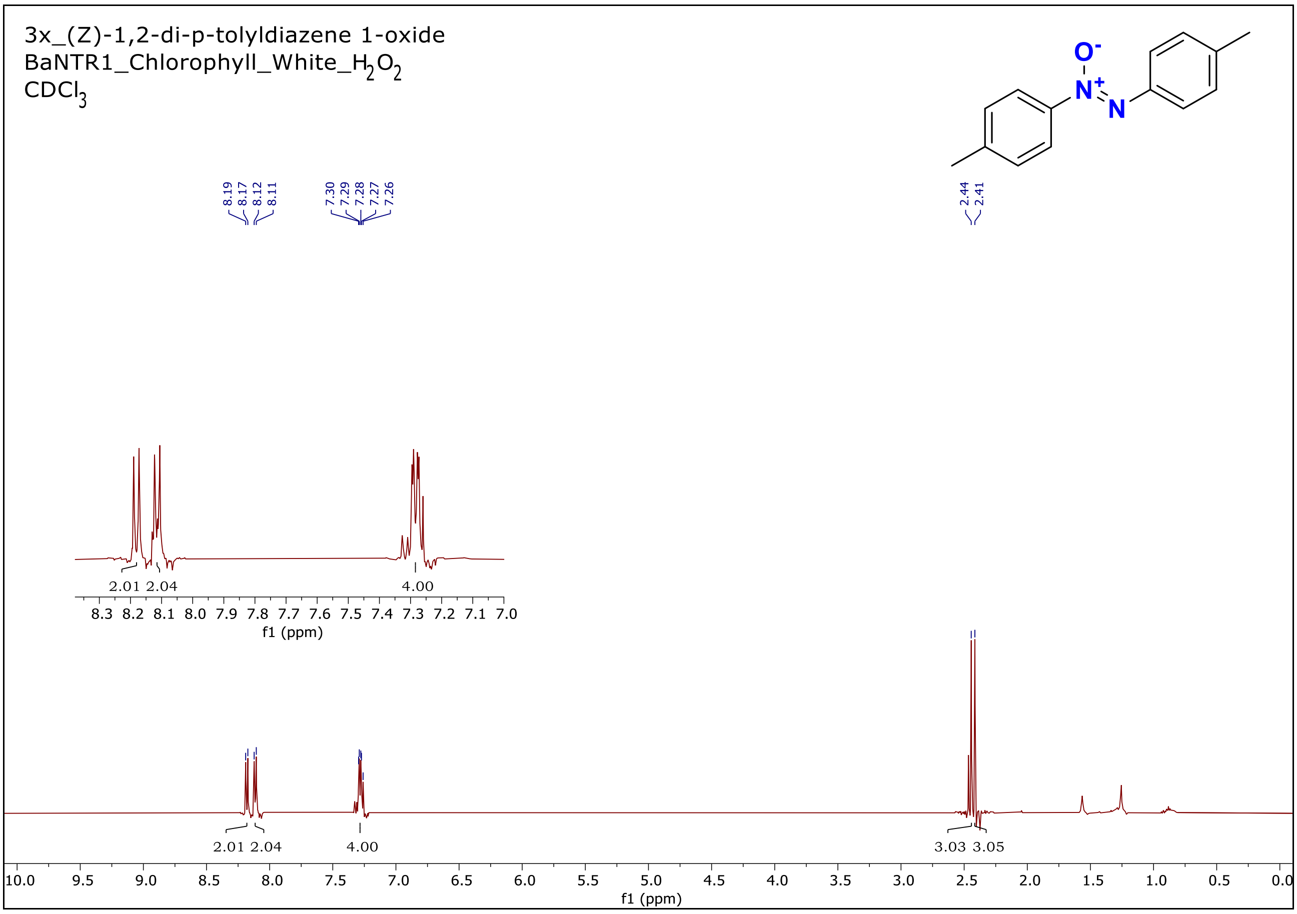

Supplementary Figure 95. ^{1}H-NMR spectrum of the photoenzymatic product **49** (top) and **50** (bottom).

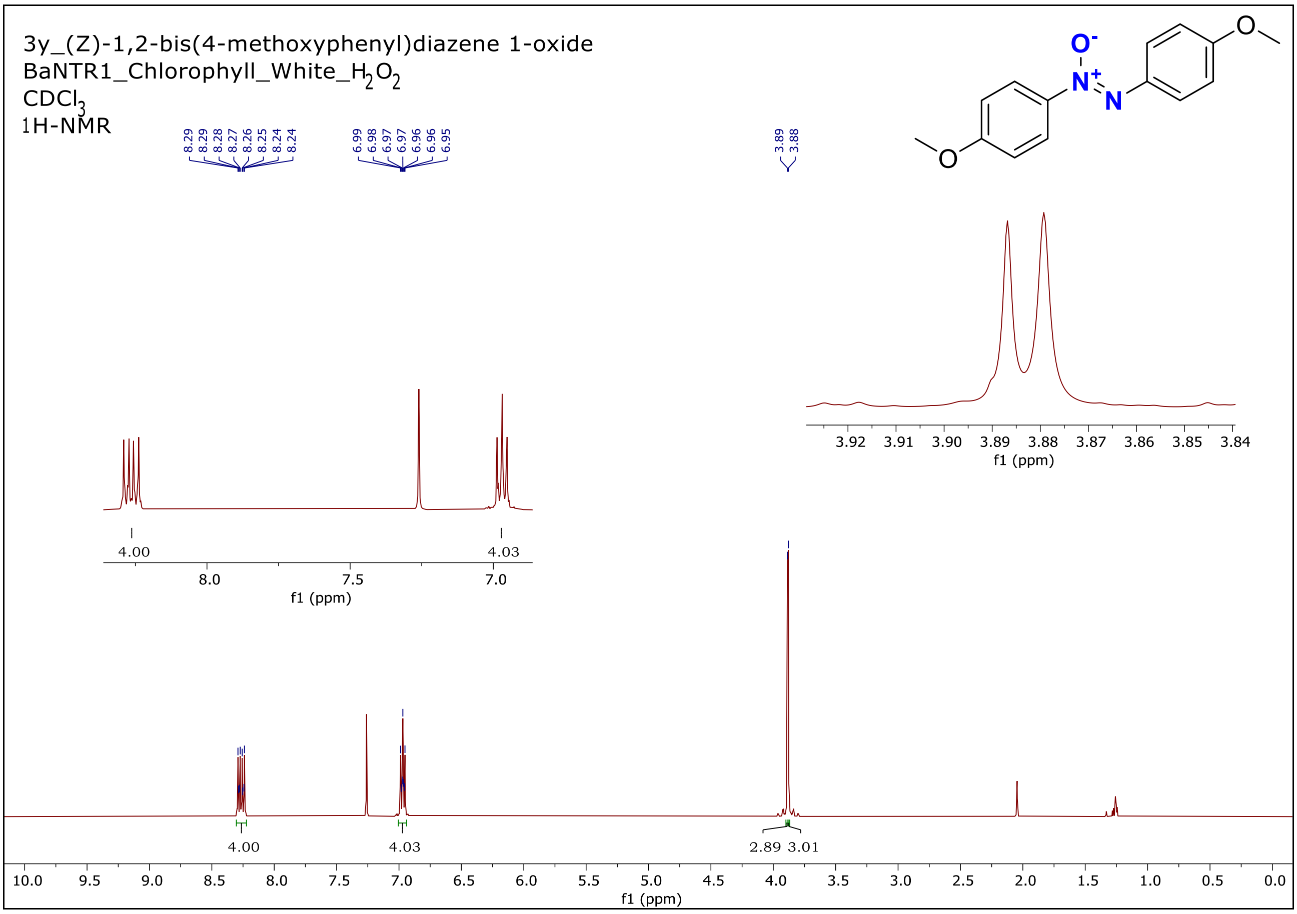

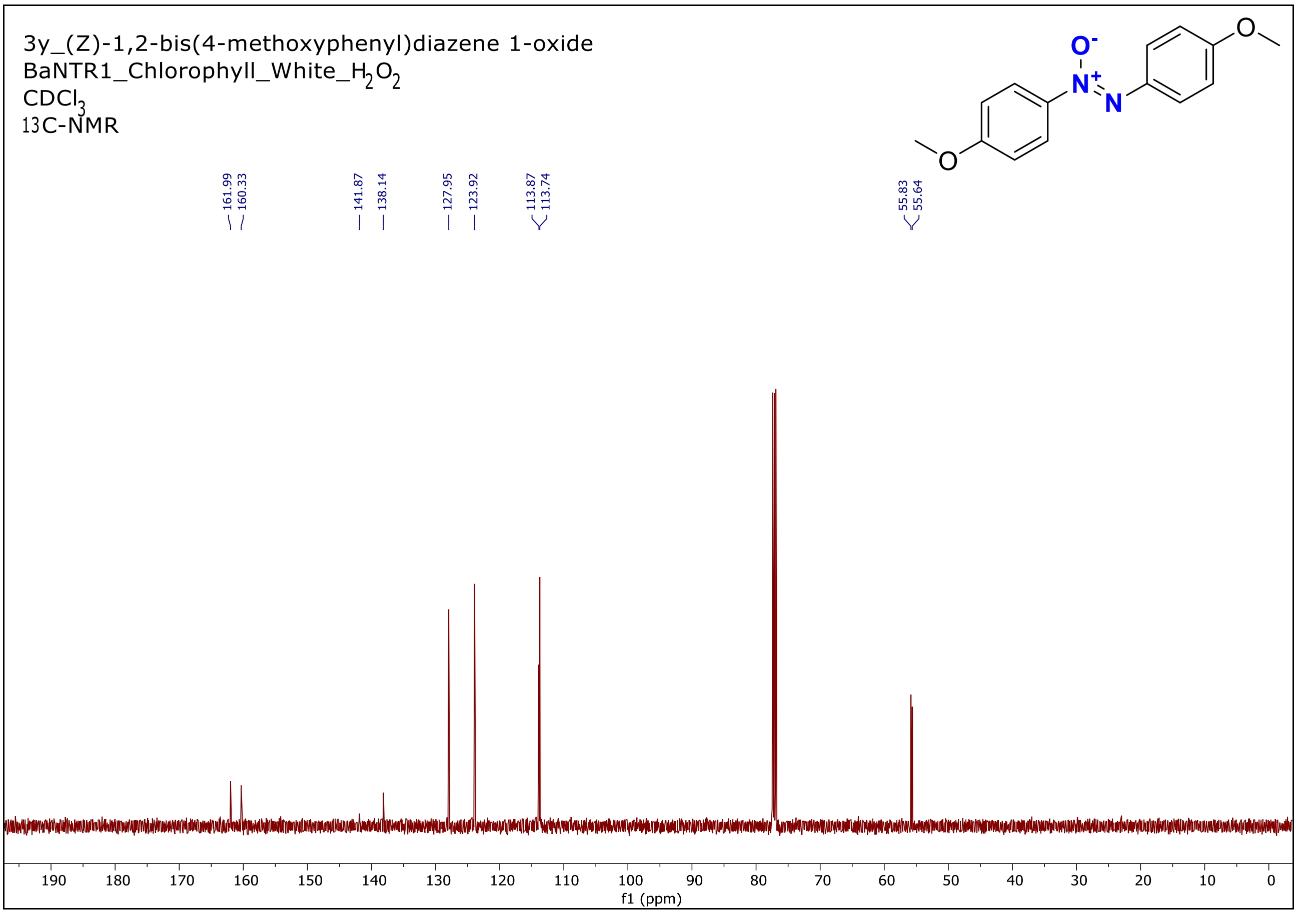

Supplementary Figure 96. ^{1}H-NMR (top) and ^{13}C-NMR (bottom) spectrum of the photoenzymatic product **51**.

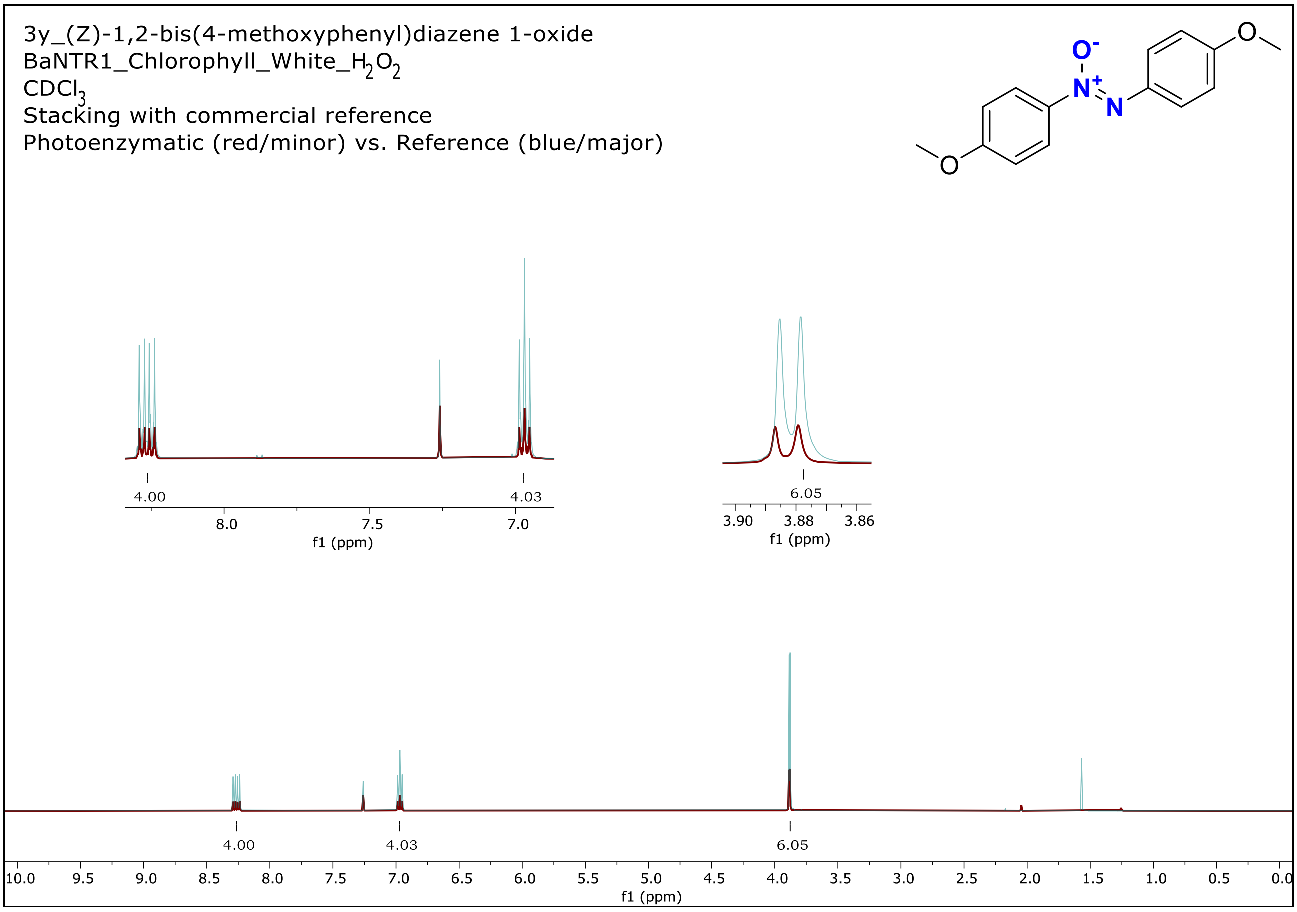

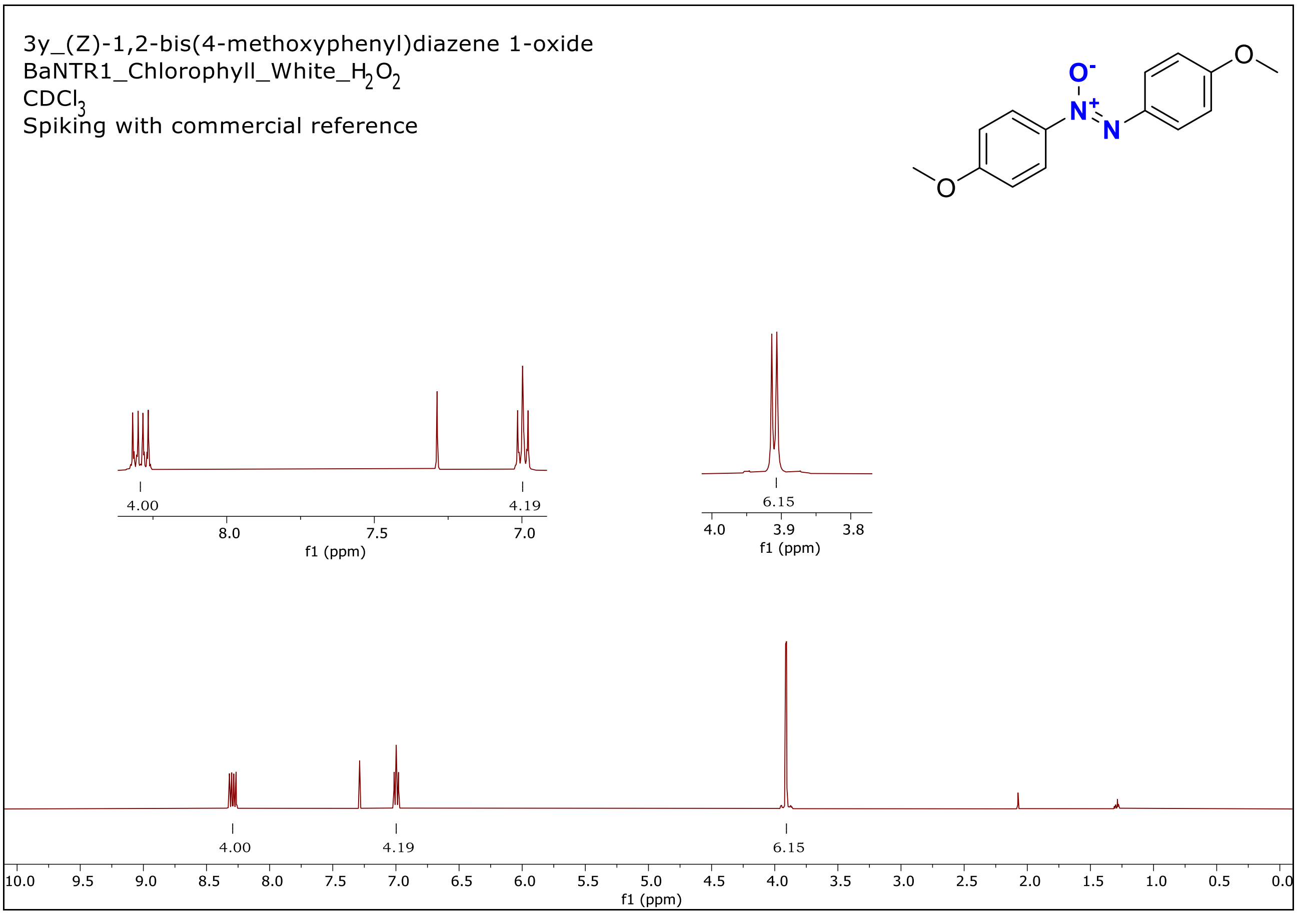

Supplementary Figure 97. Stacking (top) and spiking (bottom) ^{1}H-NMR spectrum of the photoenzymatic product **51** with a commercial reference.

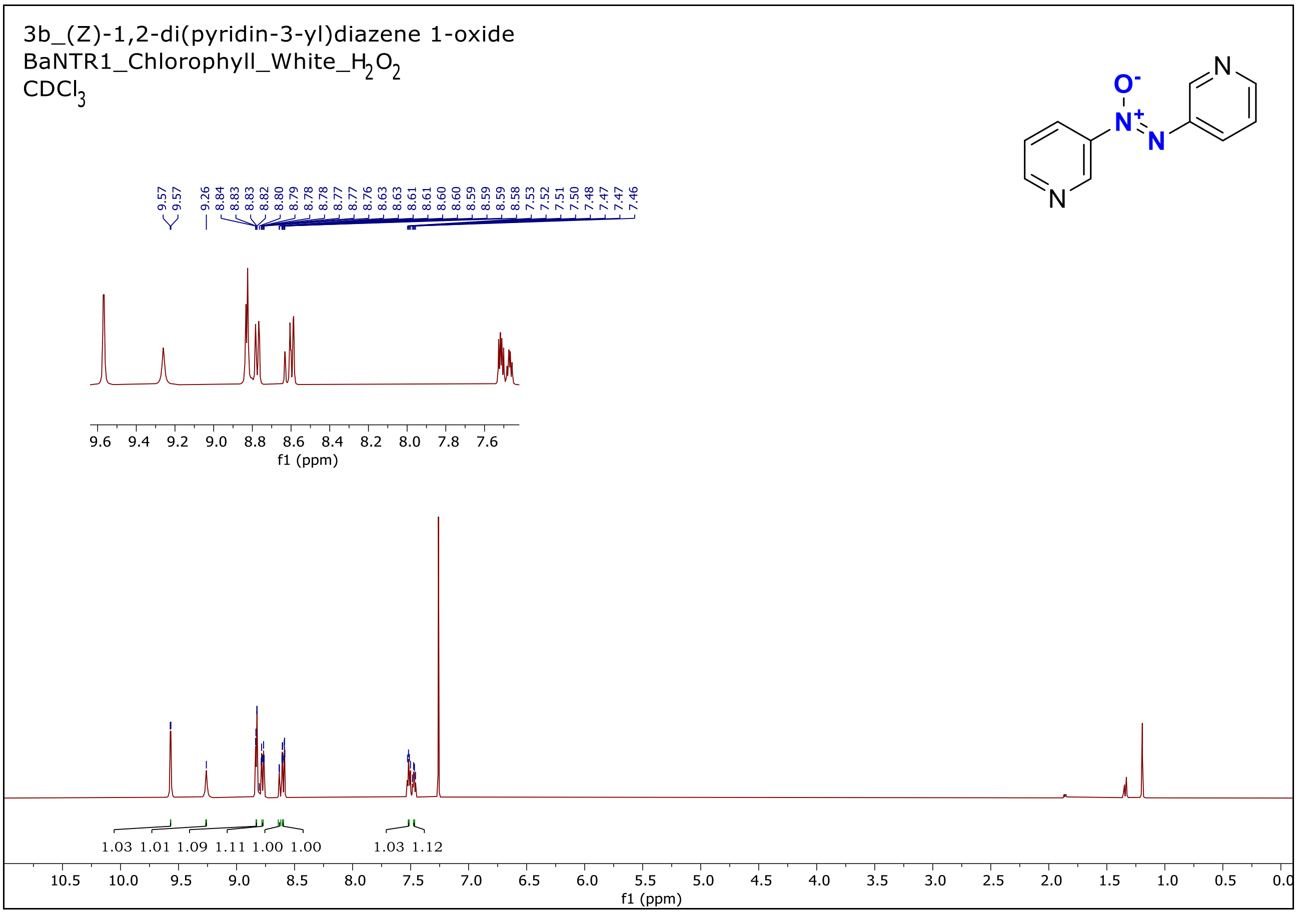

Supplementary Figure 98. ^{1}H-NMR spectrum of the photoenzymatic product **52**.

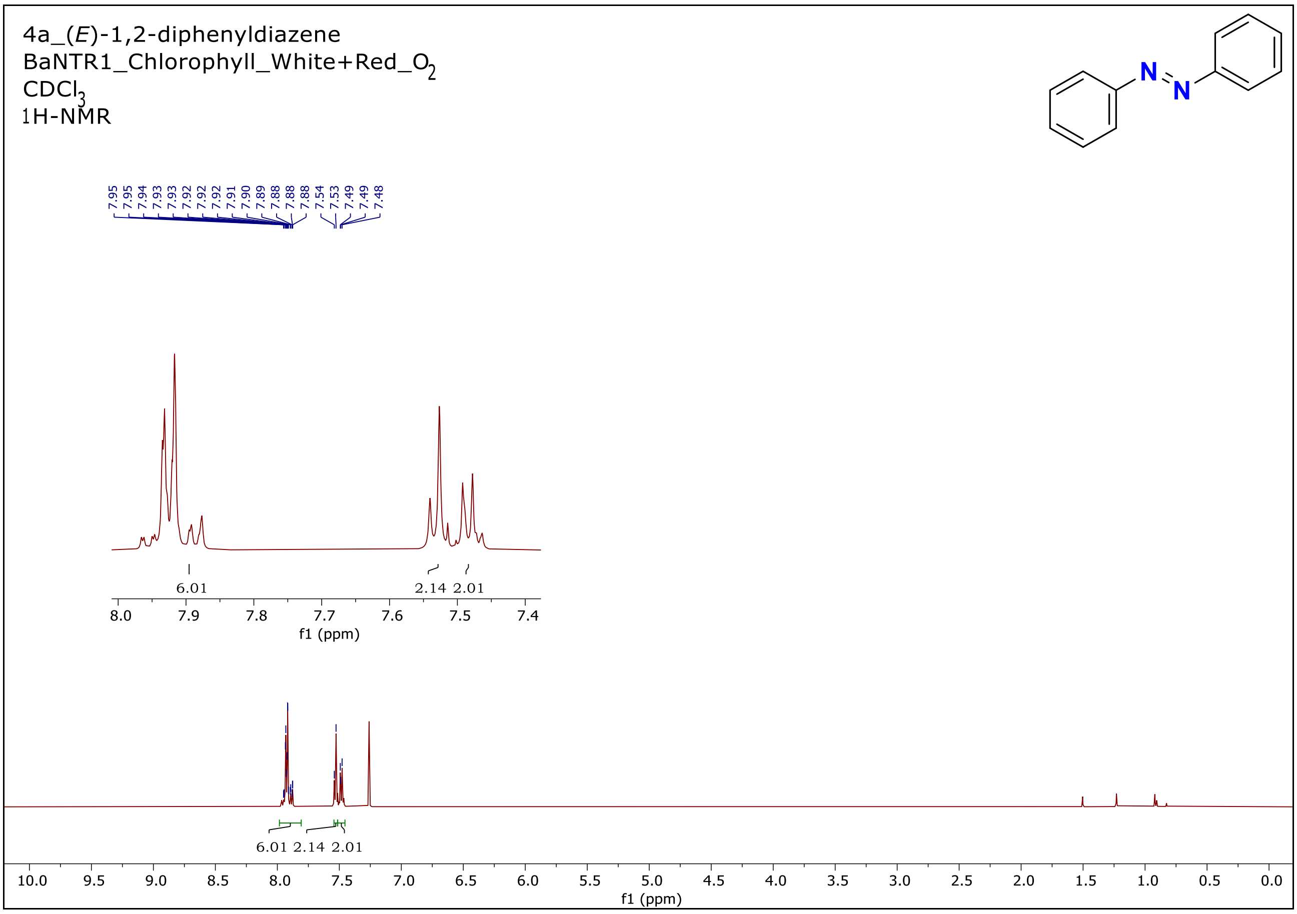

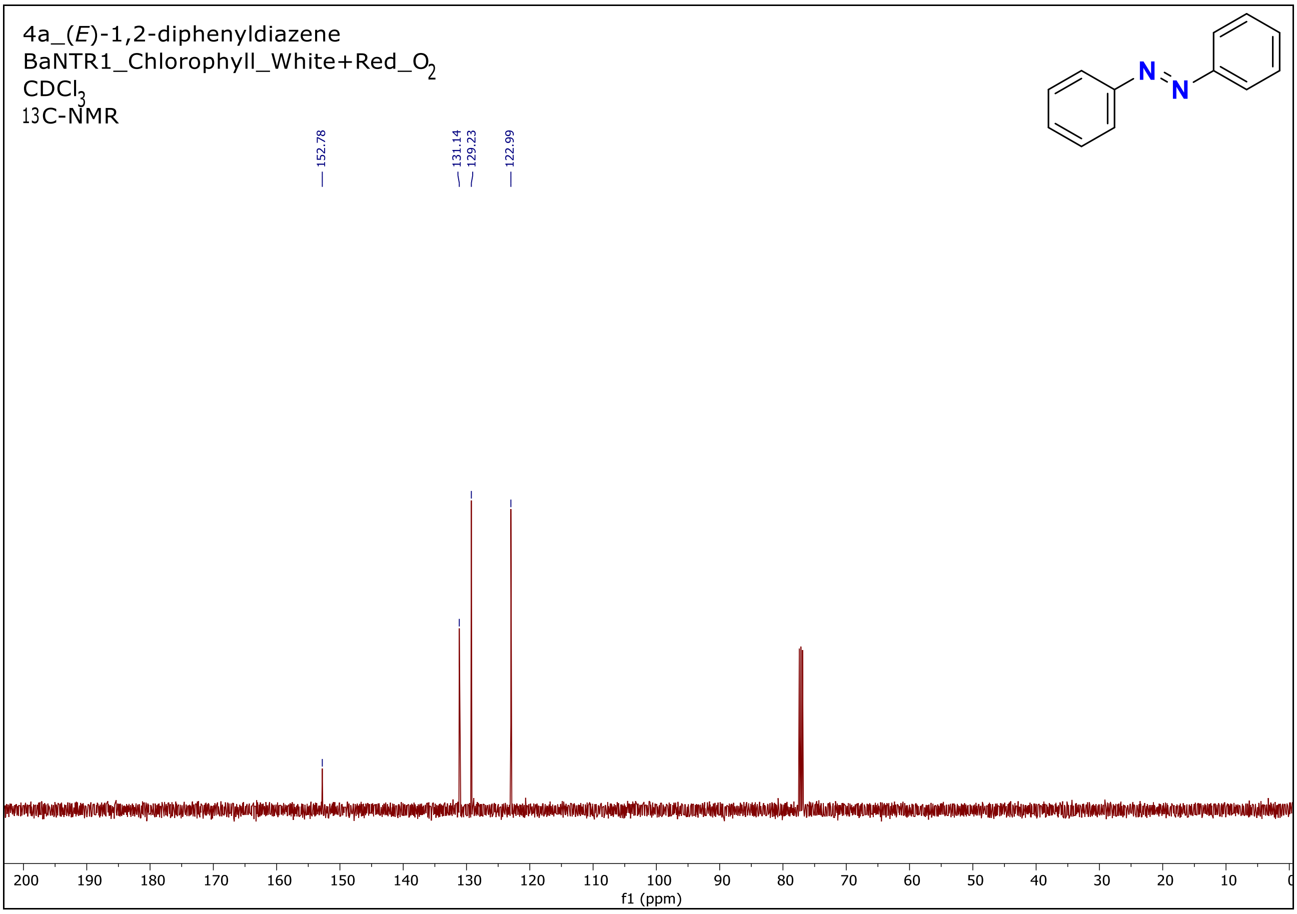

Supplementary Figure 99. ^{1}H-NMR (top) and ^{13}C-NMR (bottom) spectrum of the photoenzymatic product **53**.

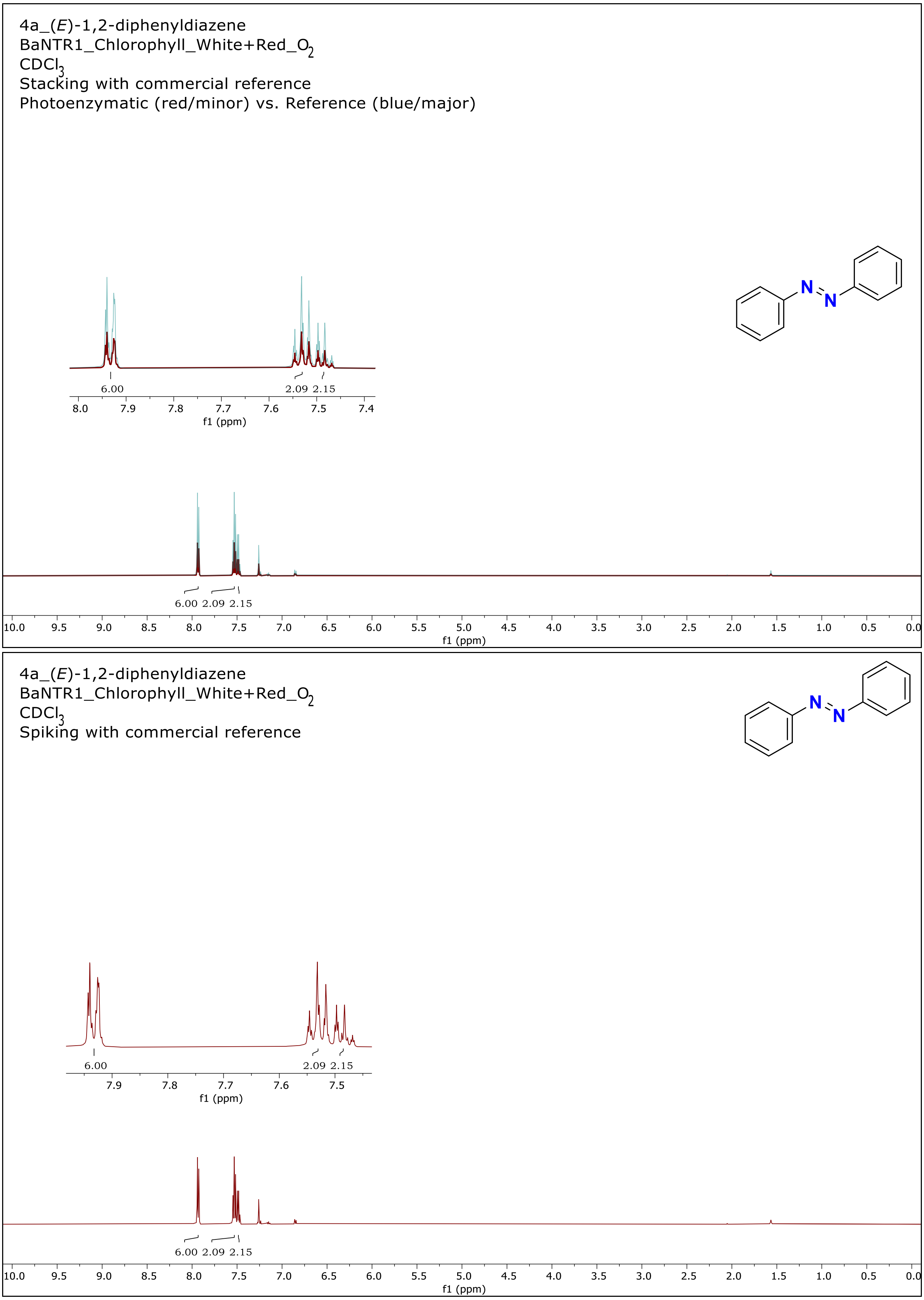

Supplementary Figure 100. Stacking (top) and spiking (bottom) ^{1}H-NMR of the photoenzymatic product **53** with a commercial reference.

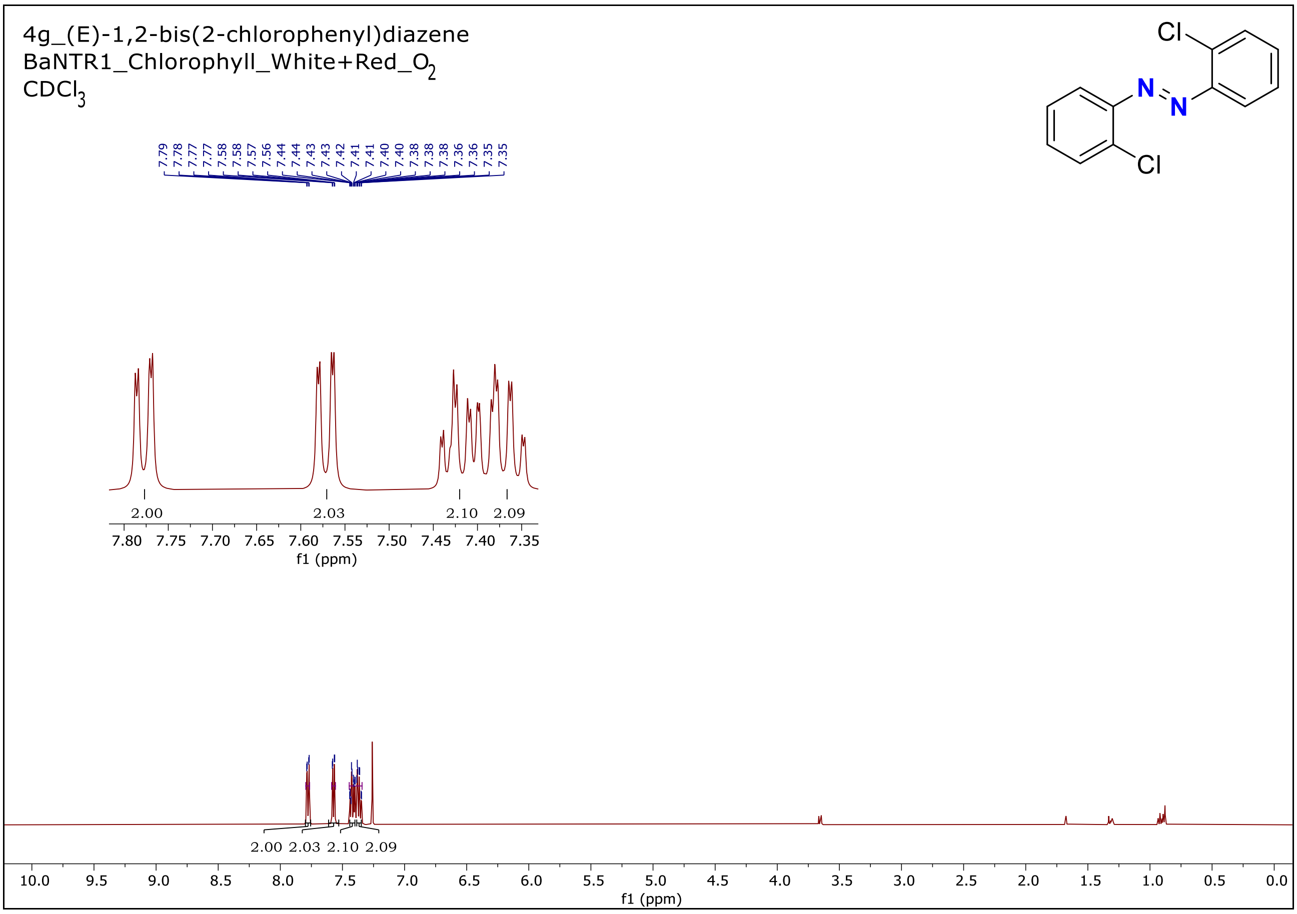

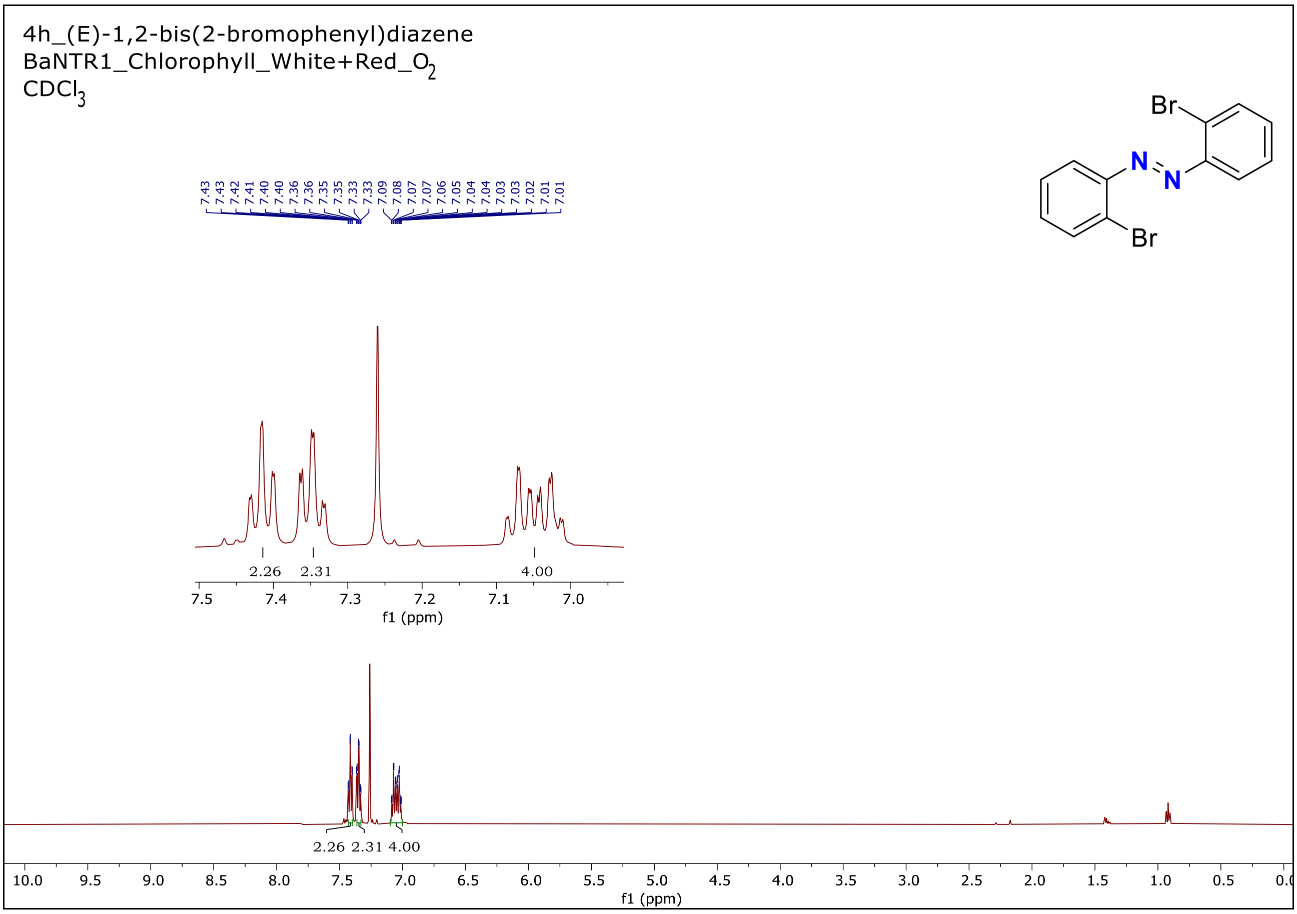

Supplementary Figure 101. ^{1}H-NMR of the photoenzymatic products **54** (top) and **55** (bottom).

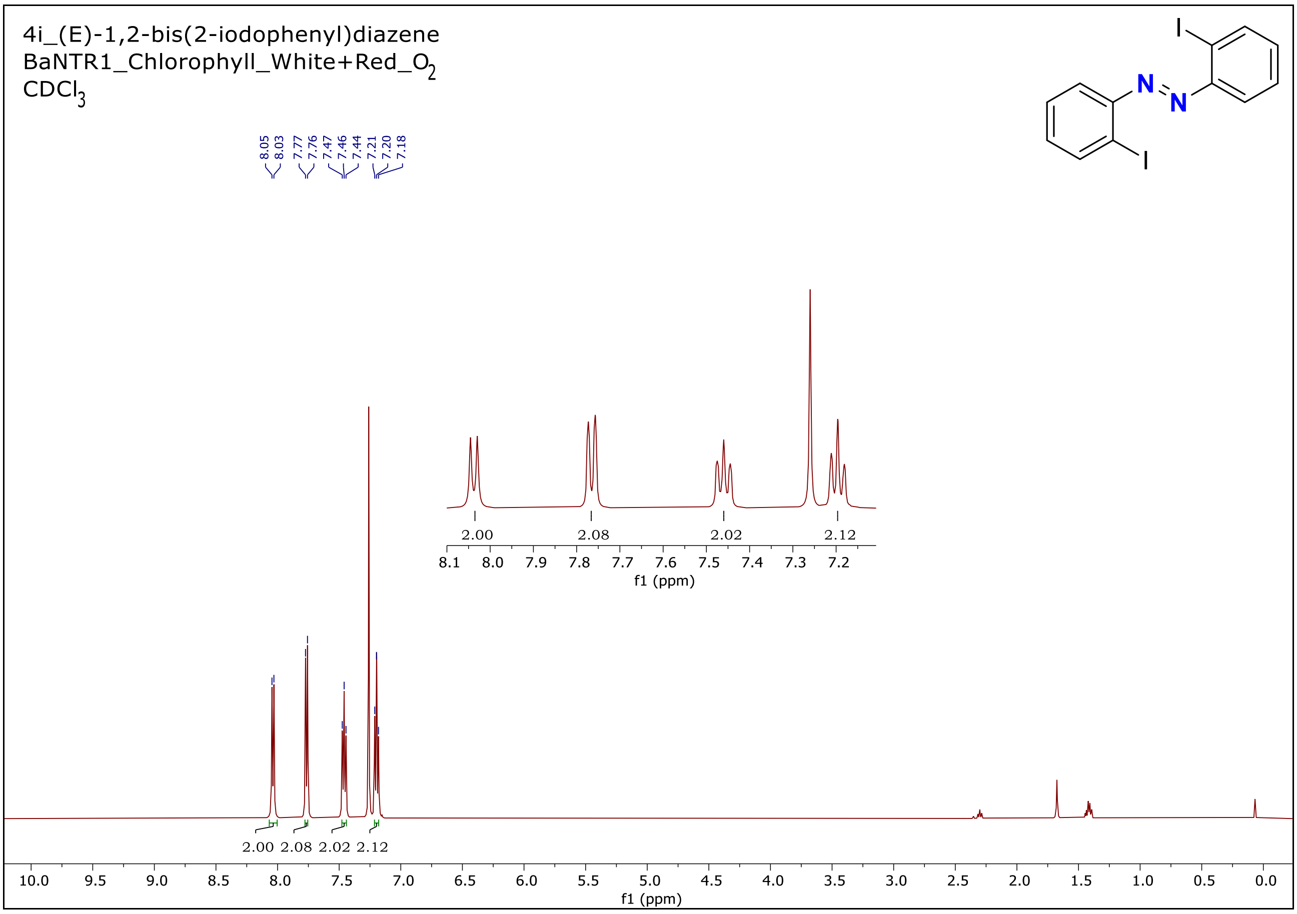

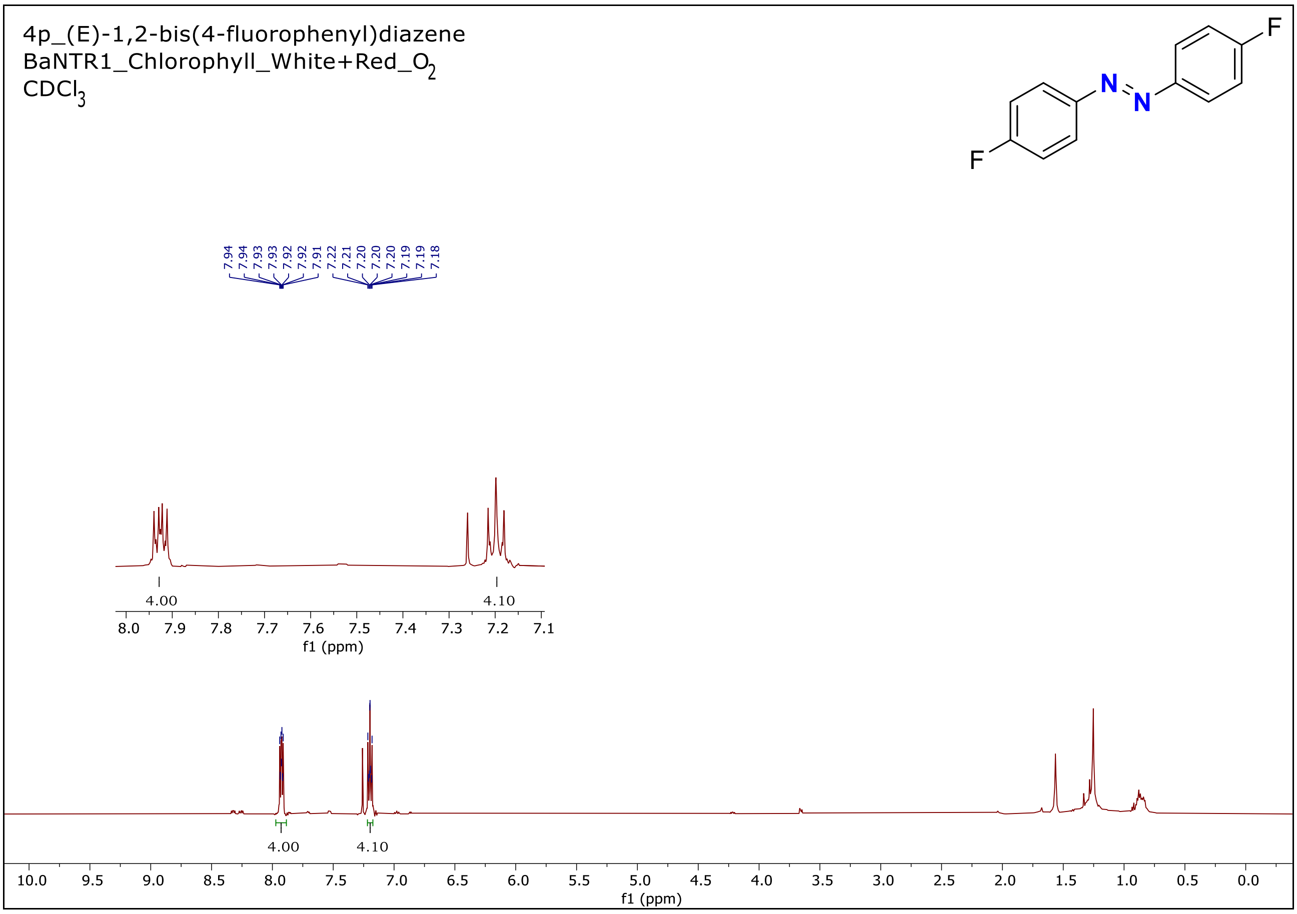

Supplementary Figure 102. ^{1}H-NMR of the photoenzymatic products **56** (top) and **57** (bottom).

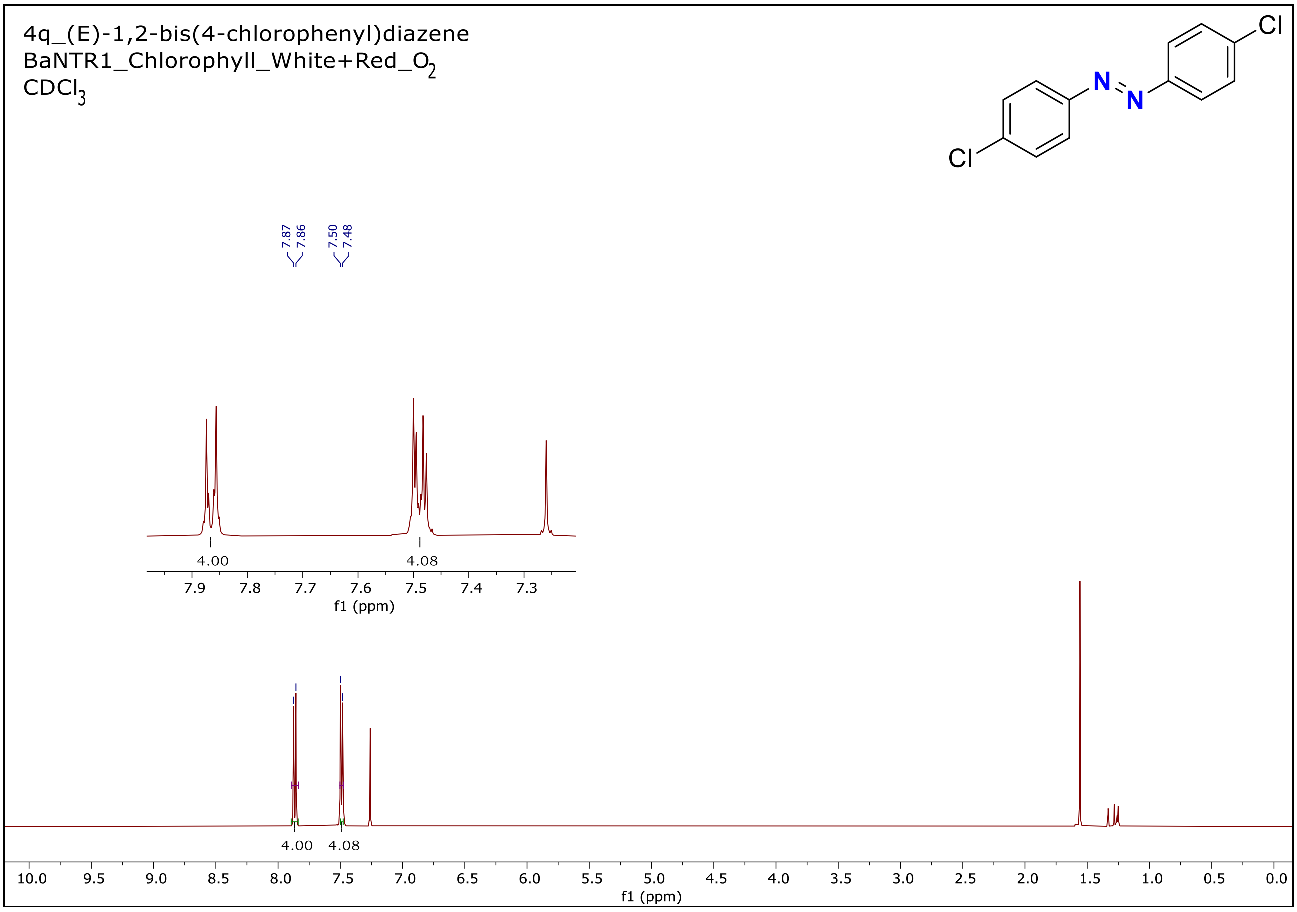

Supplementary Figure 103. ^{1}H-NMR spectrum of the photoenzymatic products **58**.

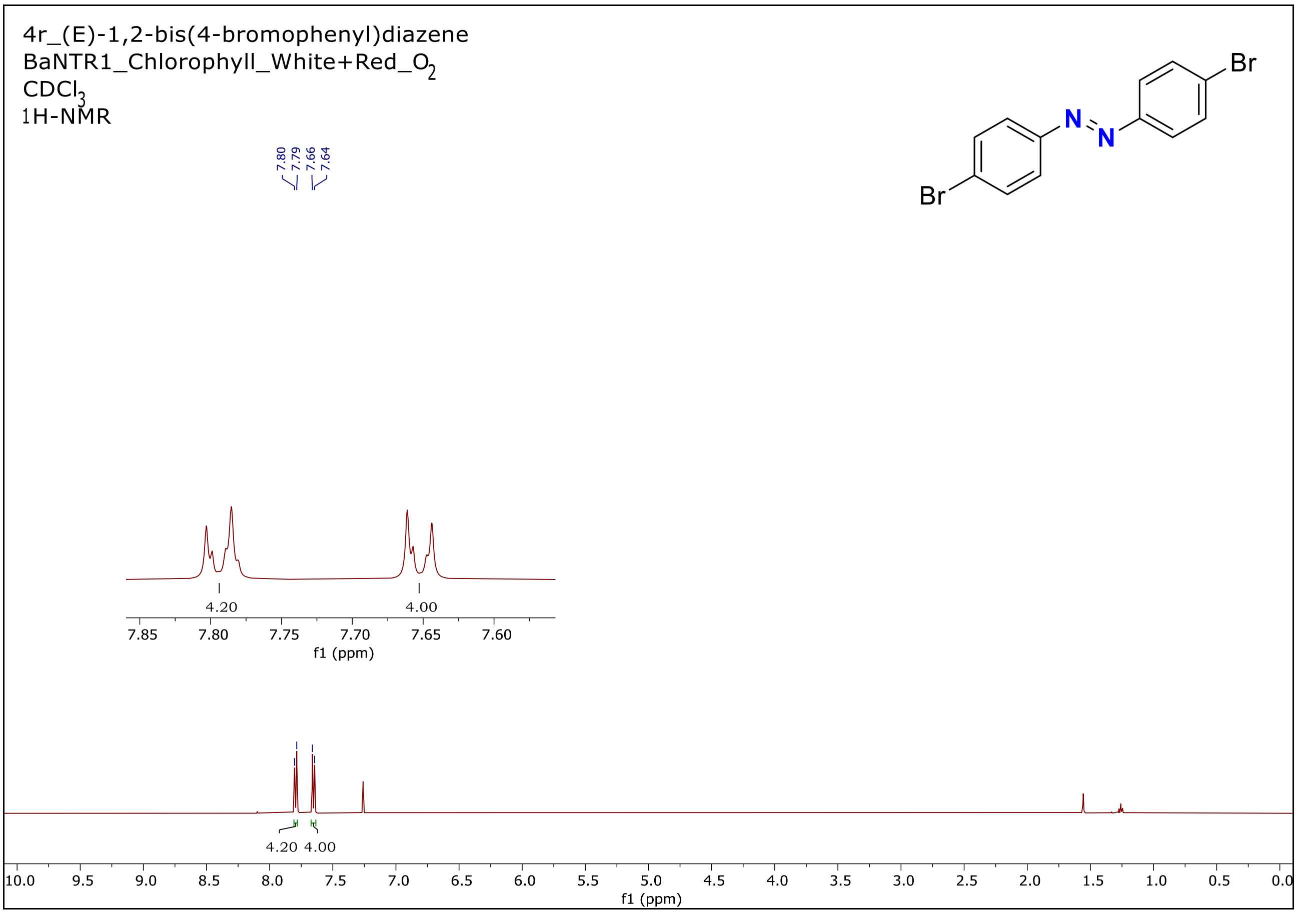

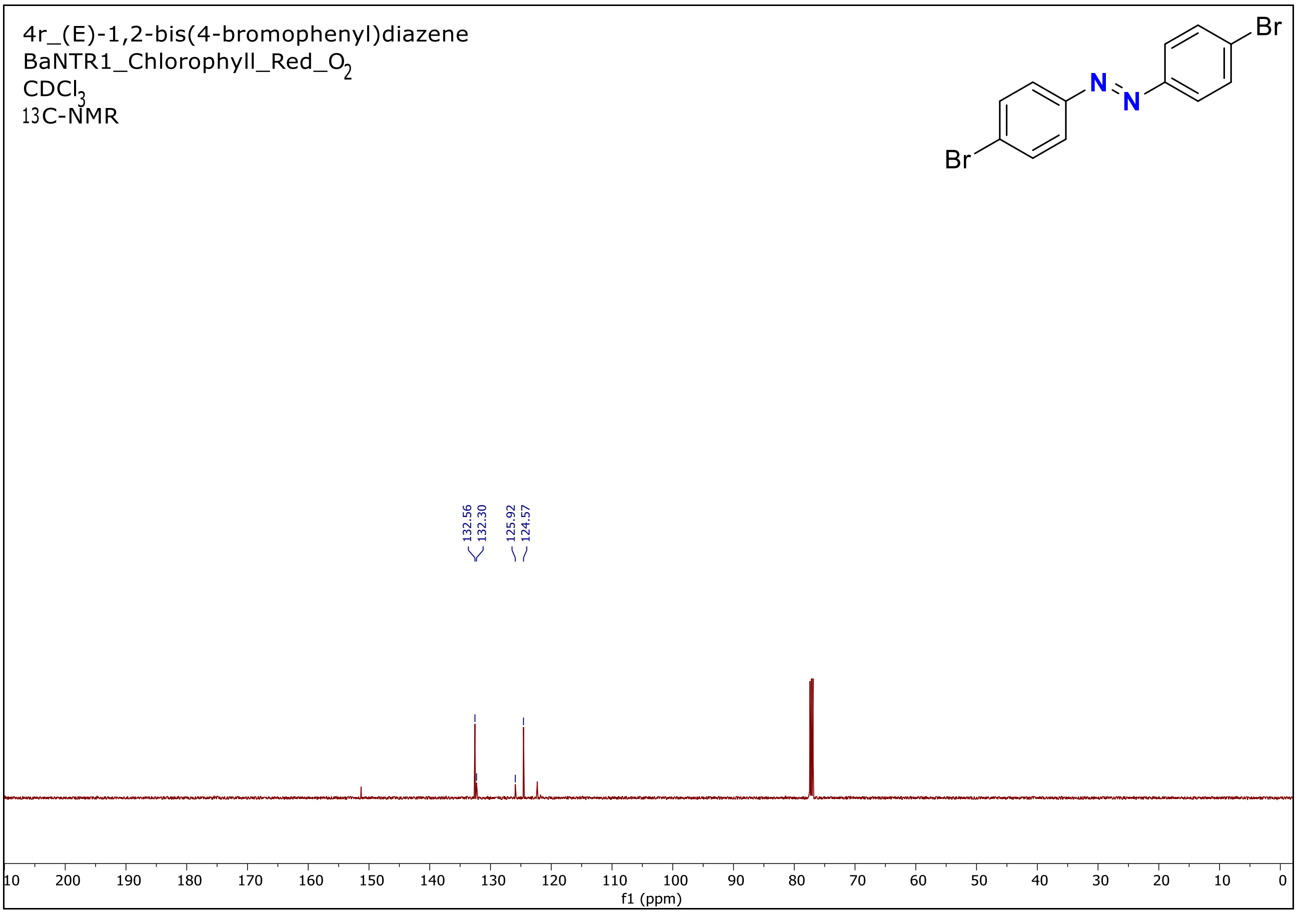

Supplementary Figure 104. ^{1}H-NMR (top) and ^{13}C-NMR (bottom) of the photoenzymatic product **59**.

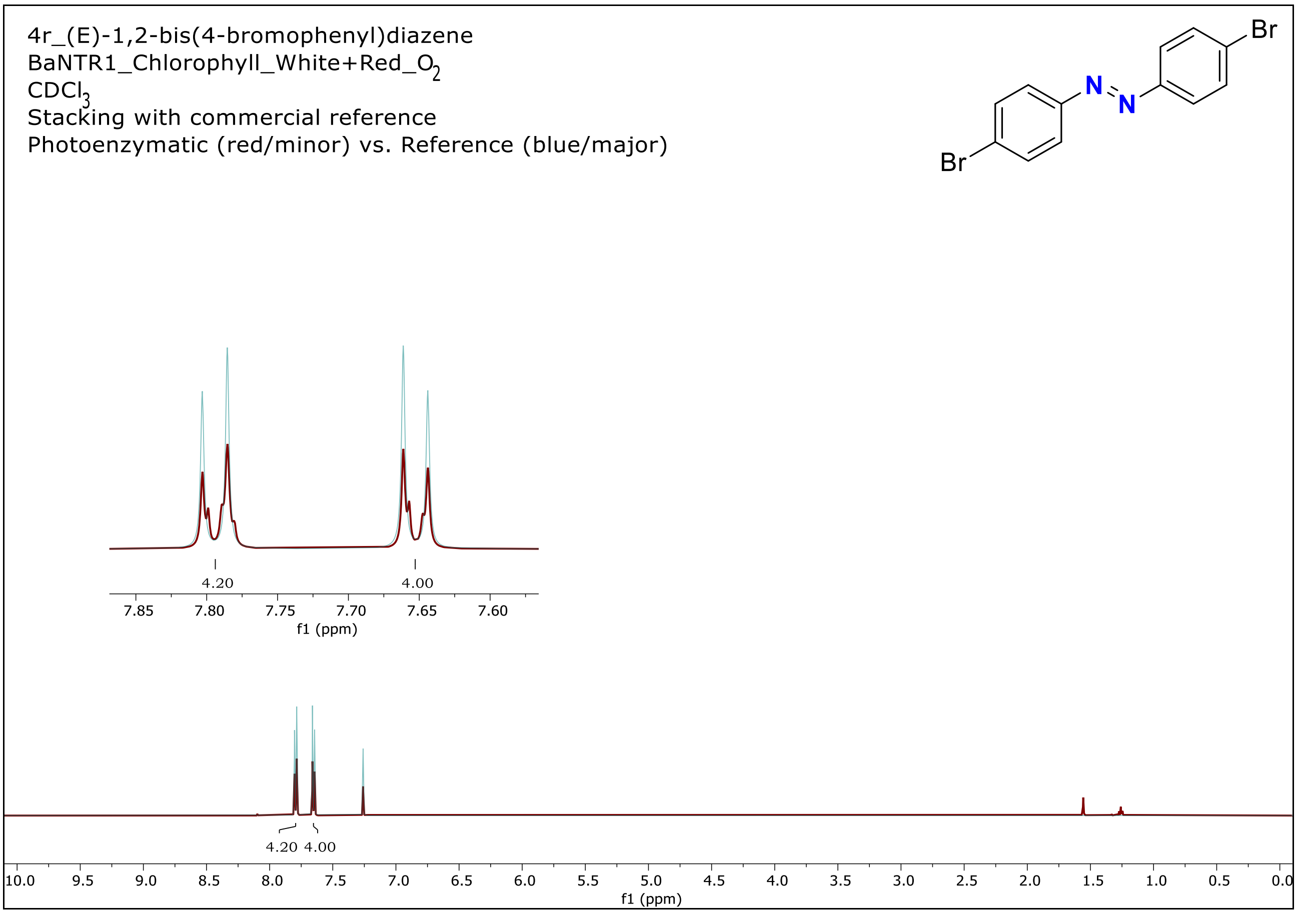

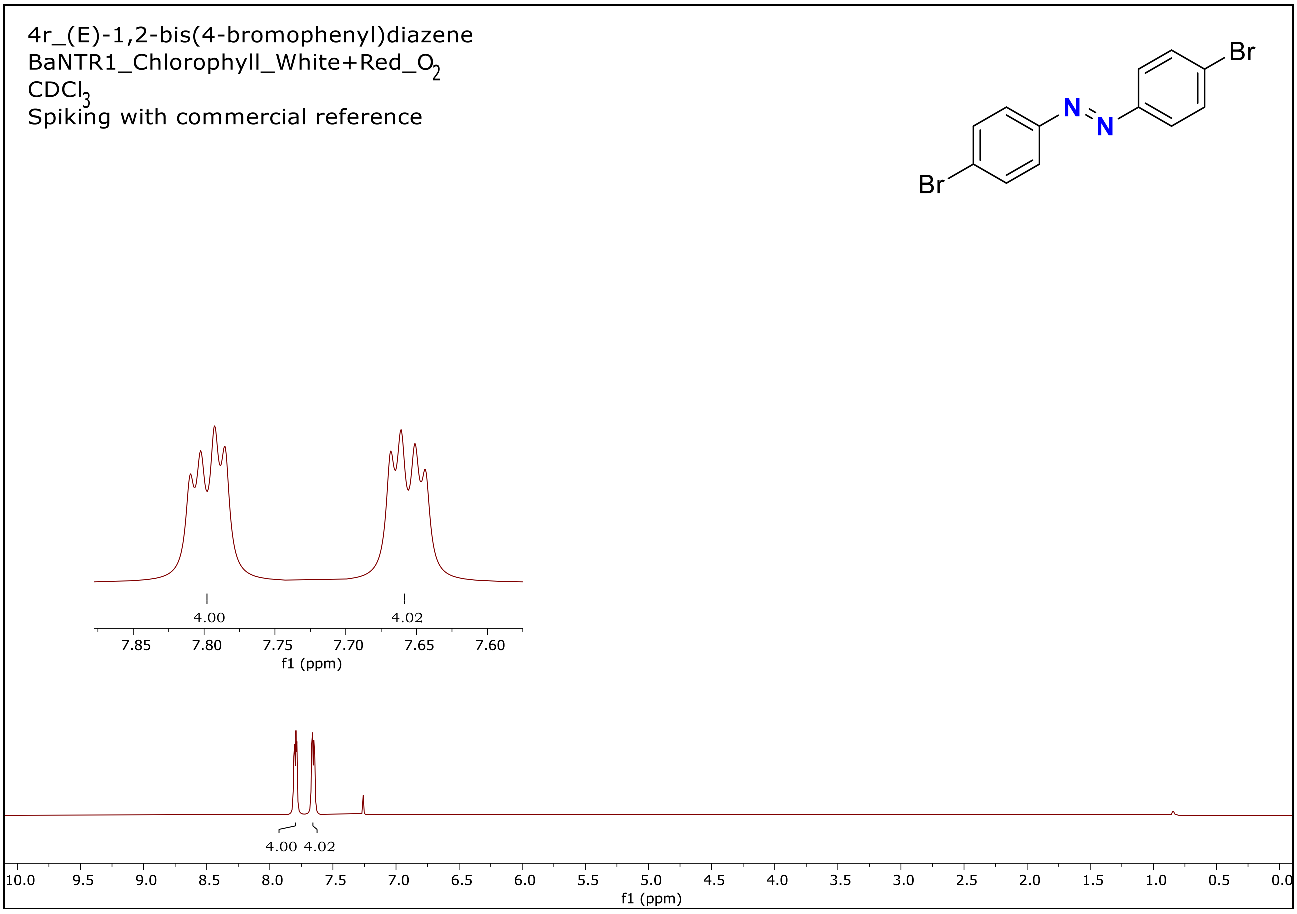

Supplementary Figure 105. Stacking (top) and spiking (bottom) ^{1}H-NMR of the photoenzymatic product **59** with a commercial reference.

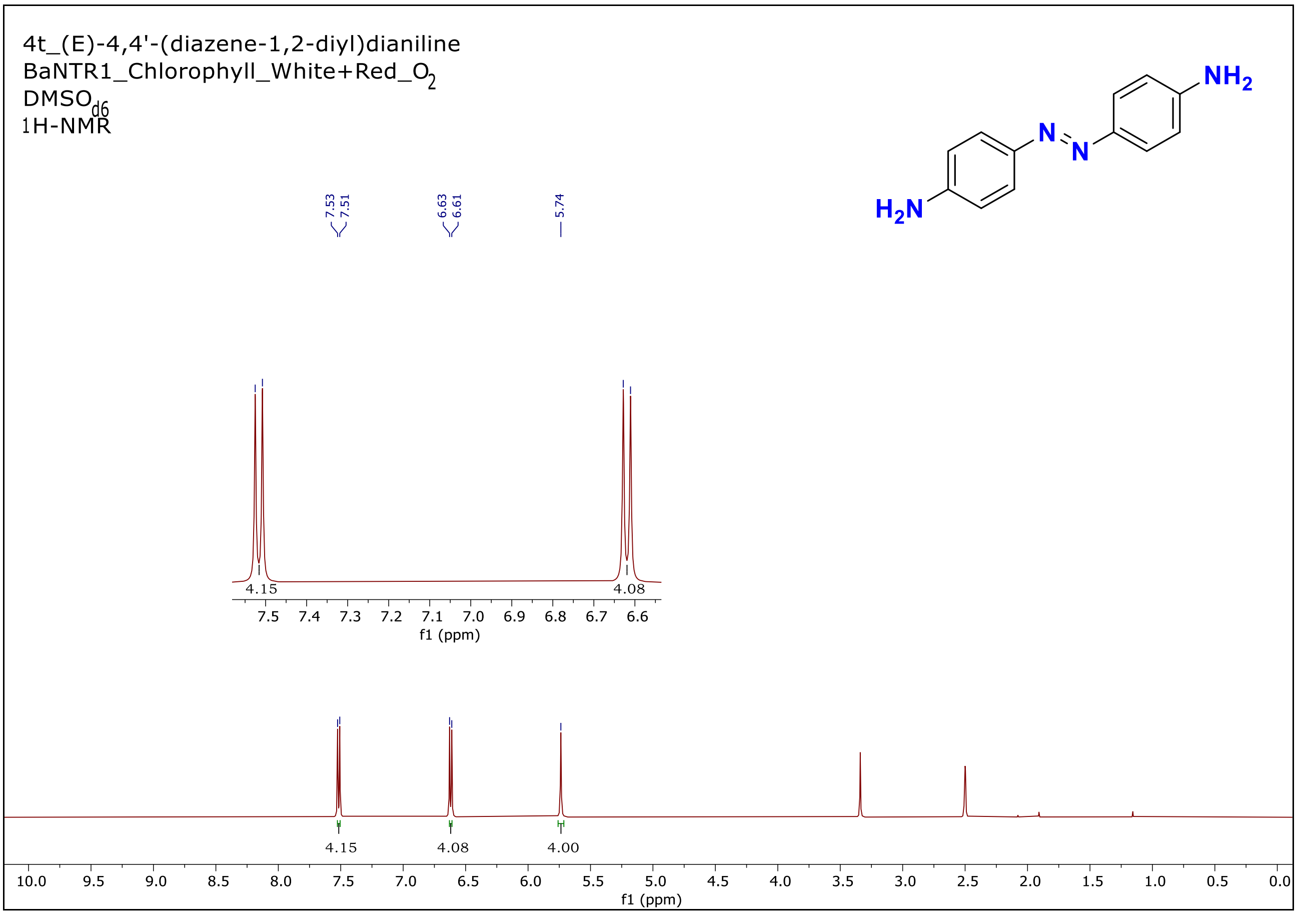

Supplementary Figure 106. ^{1}H-NMR spectrum of the photoenzymatic product **60**.

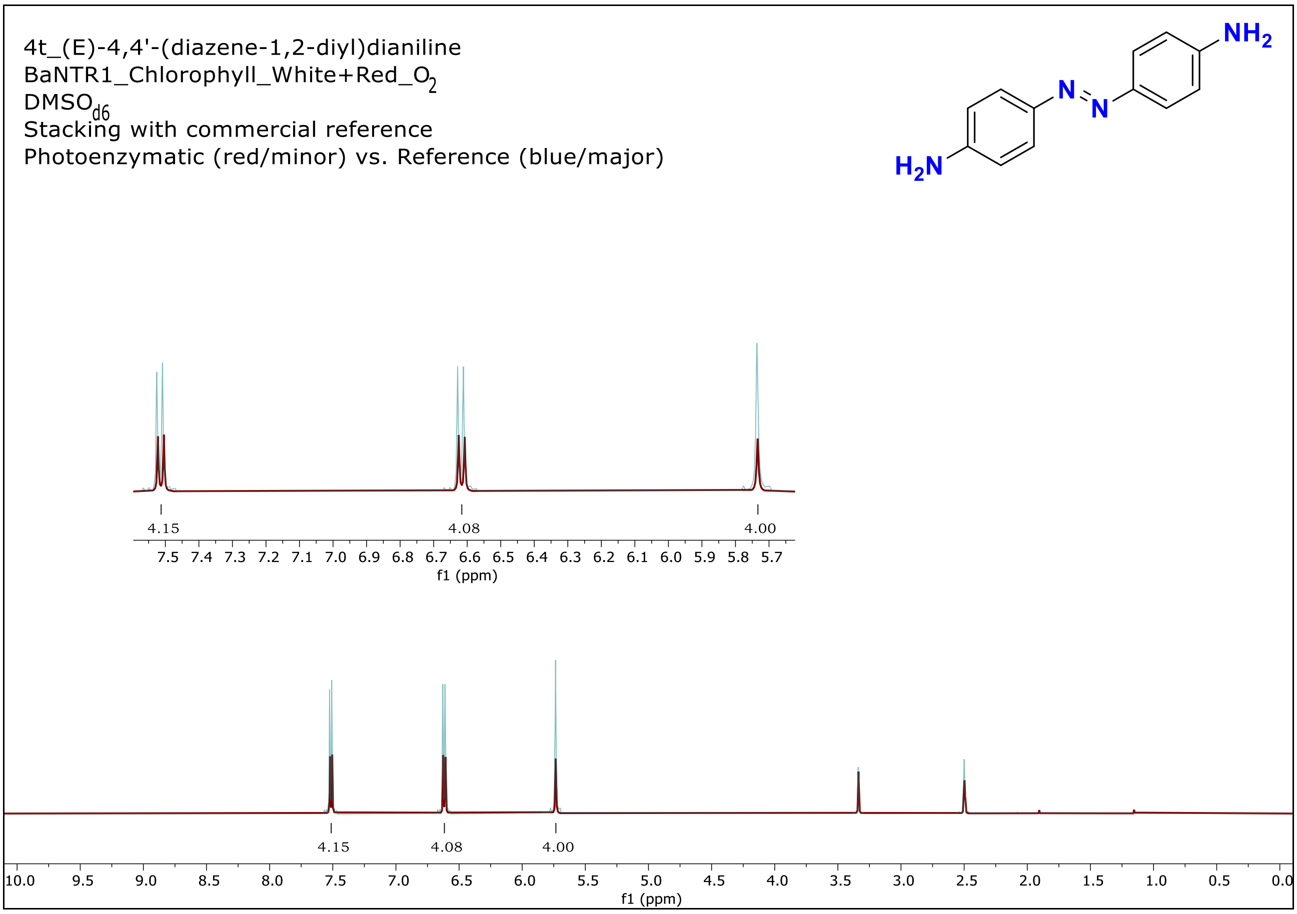

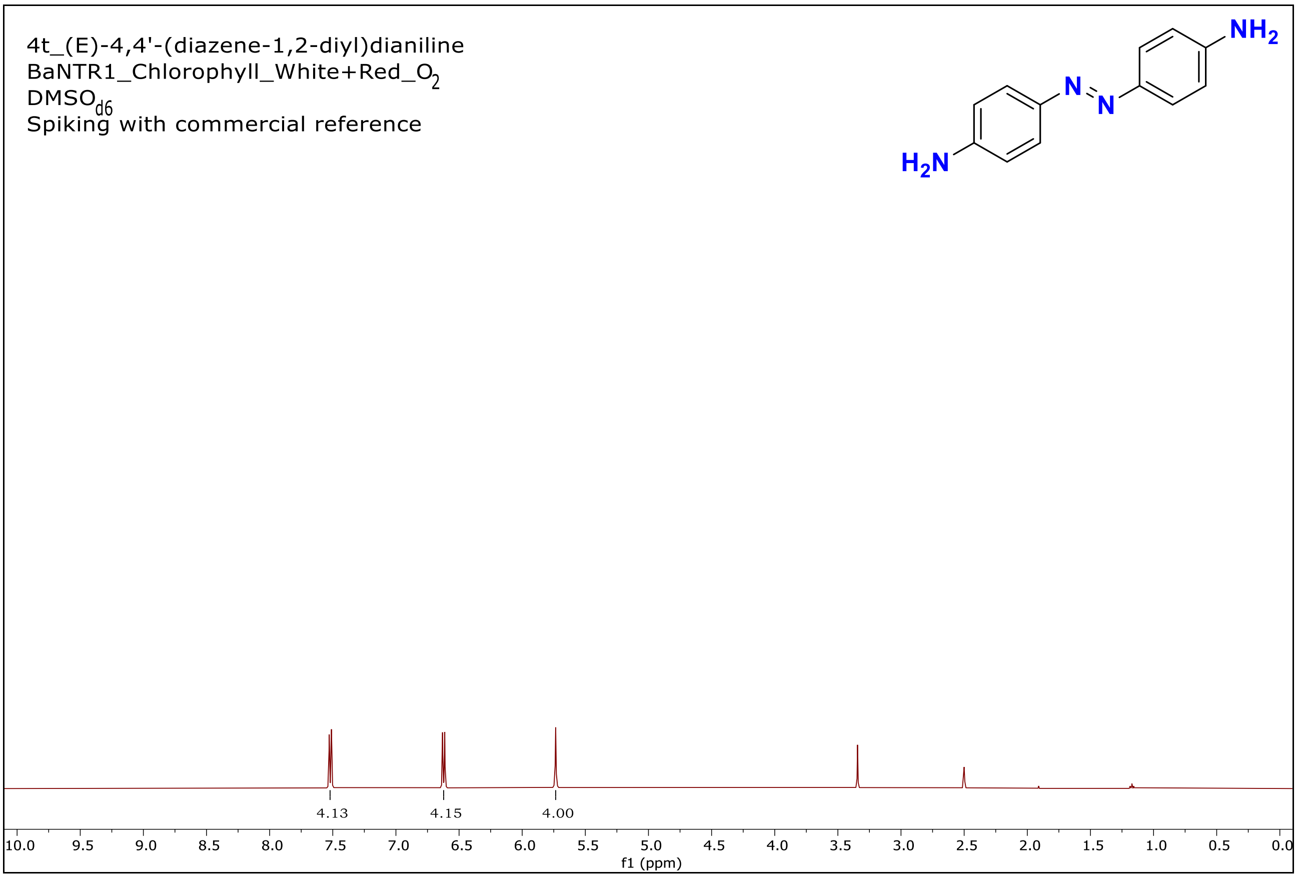

Supplementary Figure 107. Stacking (top) and spiking (bottom) ^{1}H-NMR spectrum of the photoenzymatic product **60** with a commercial reference.

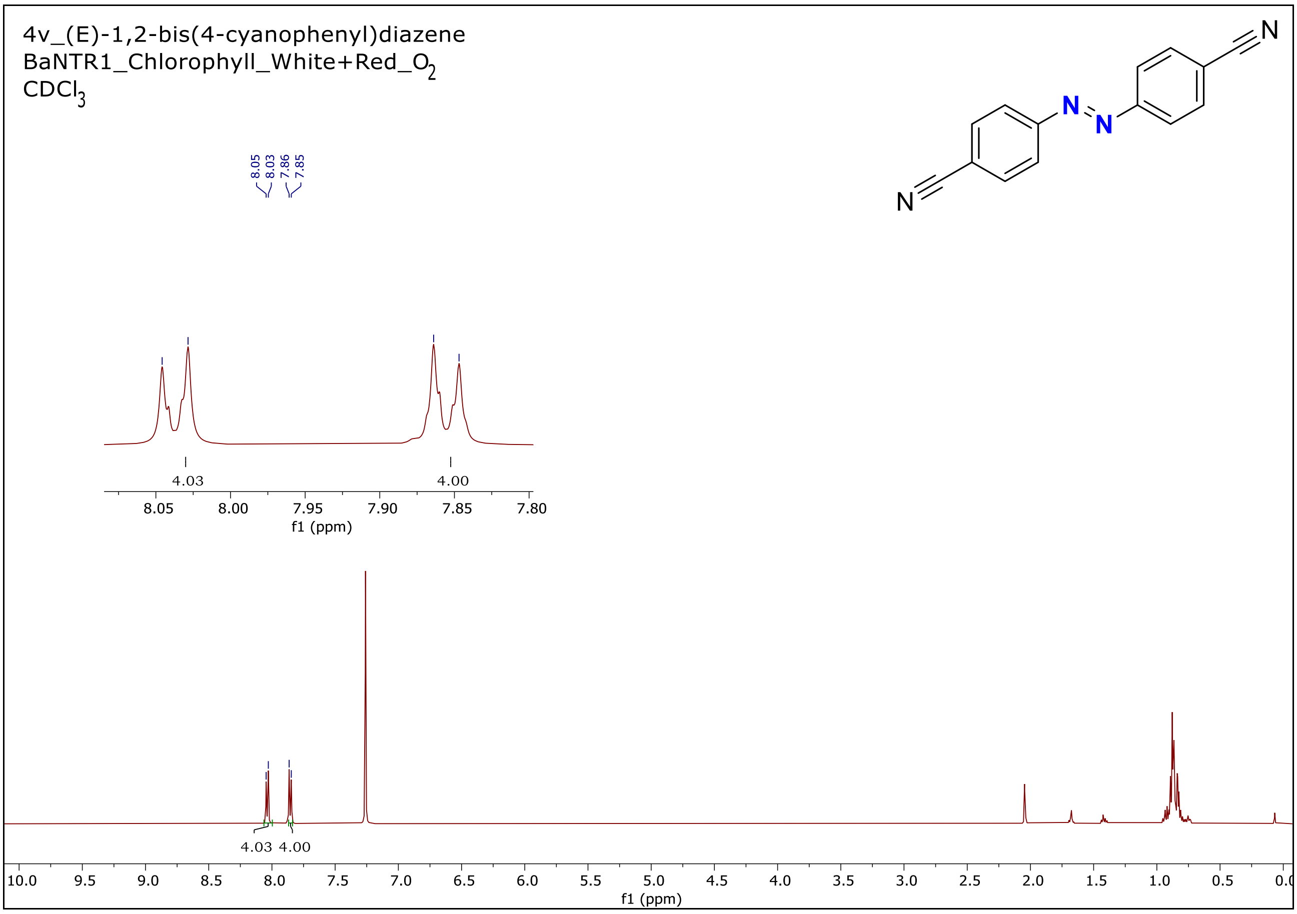

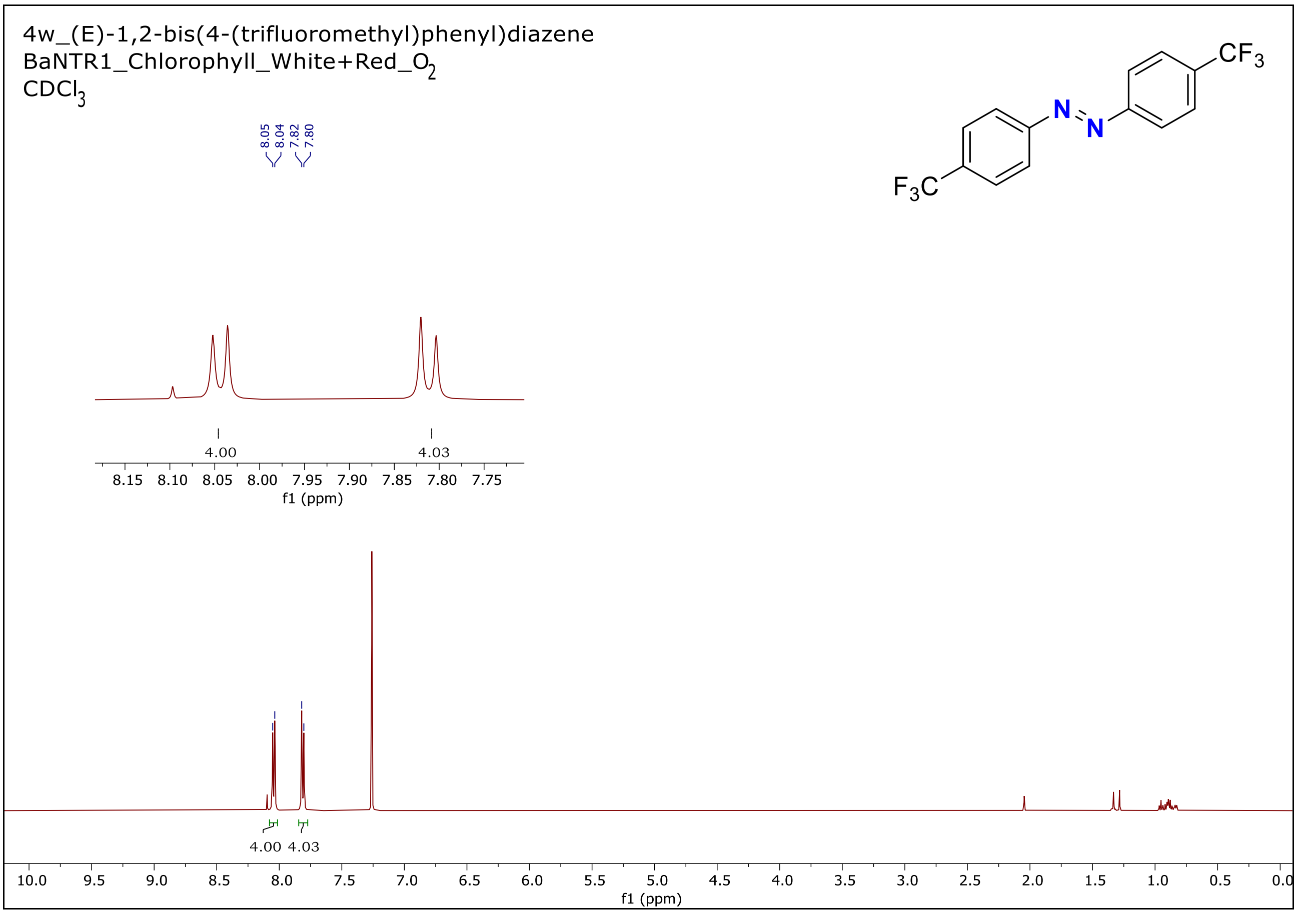

Supplementary Figure 108. ^{1}H-NMR of the photoenzymatic products **61** (top) and **62** (bottom).

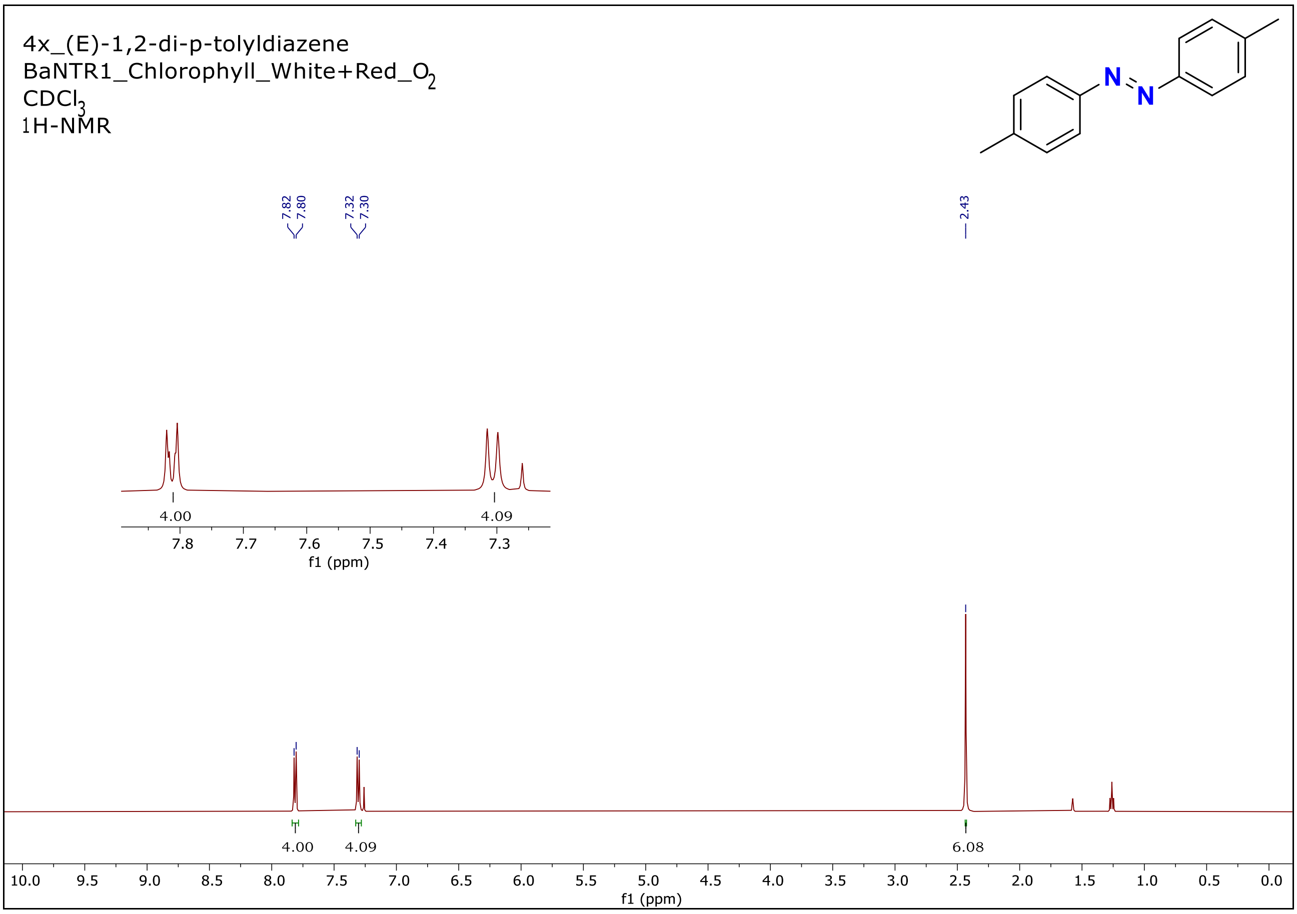

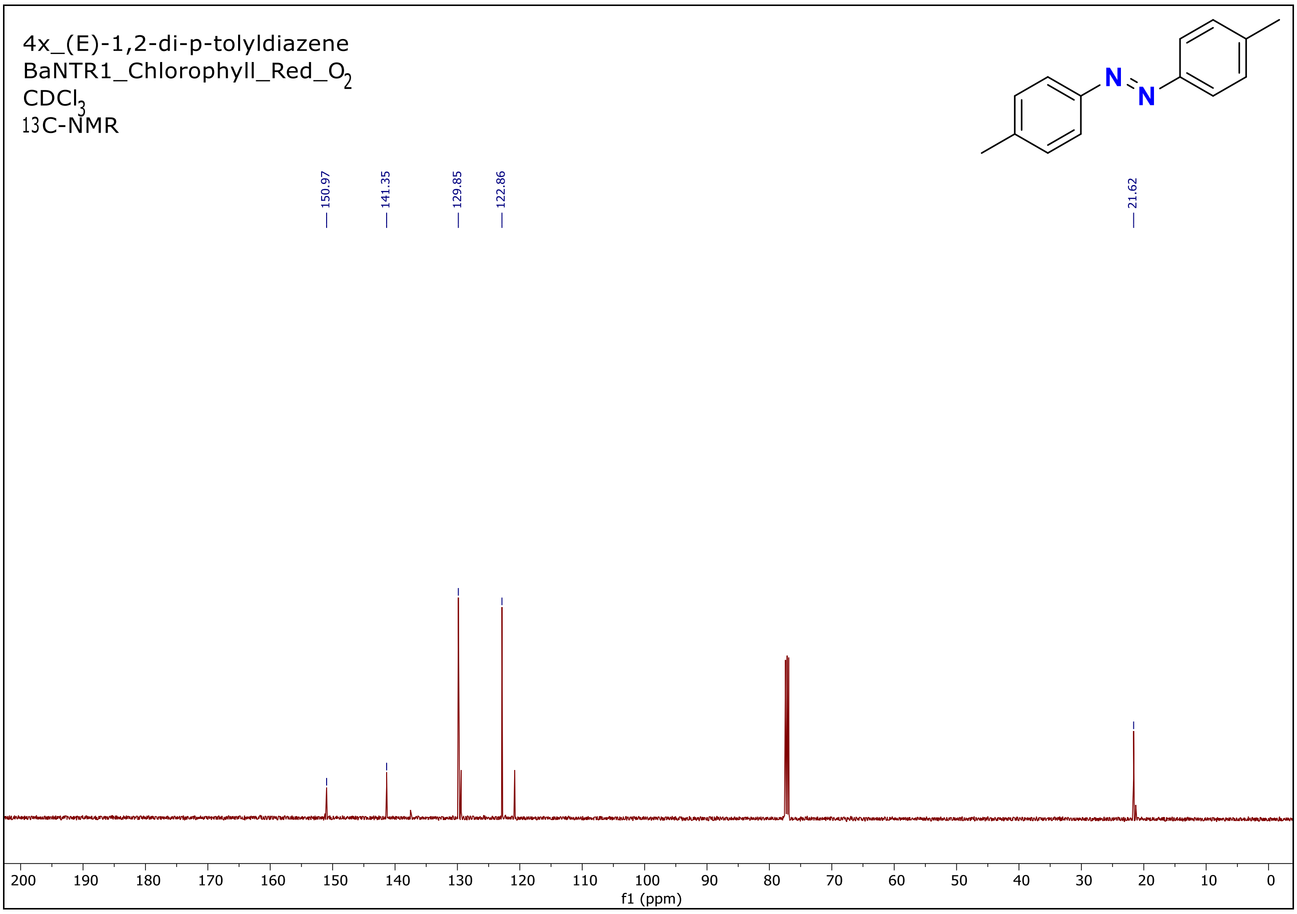

Supplementary Figure 109. ^{1}H-NMR (top) and ^{13}C-NMR (bottom) of the photoenzymatic product **63**.

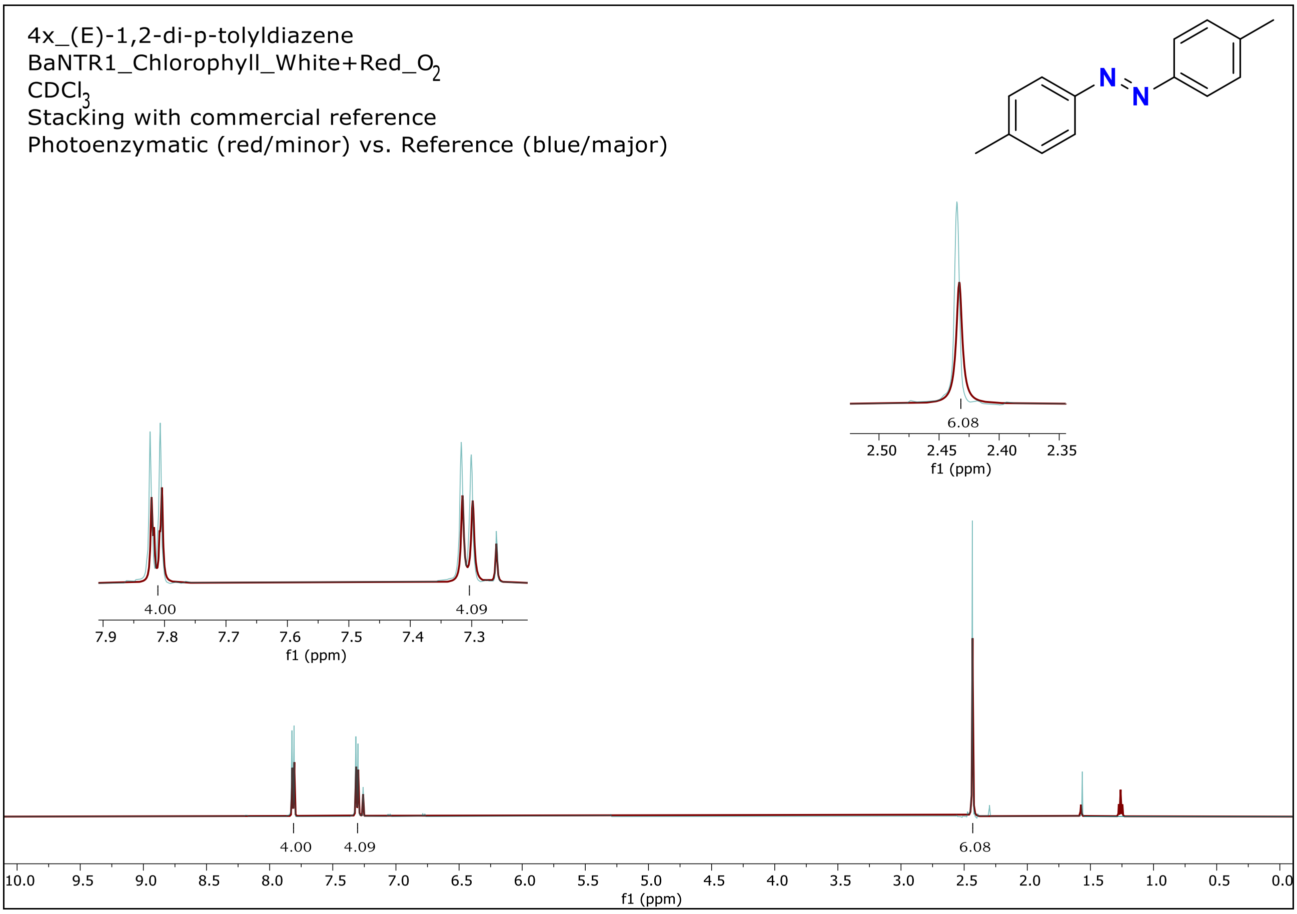

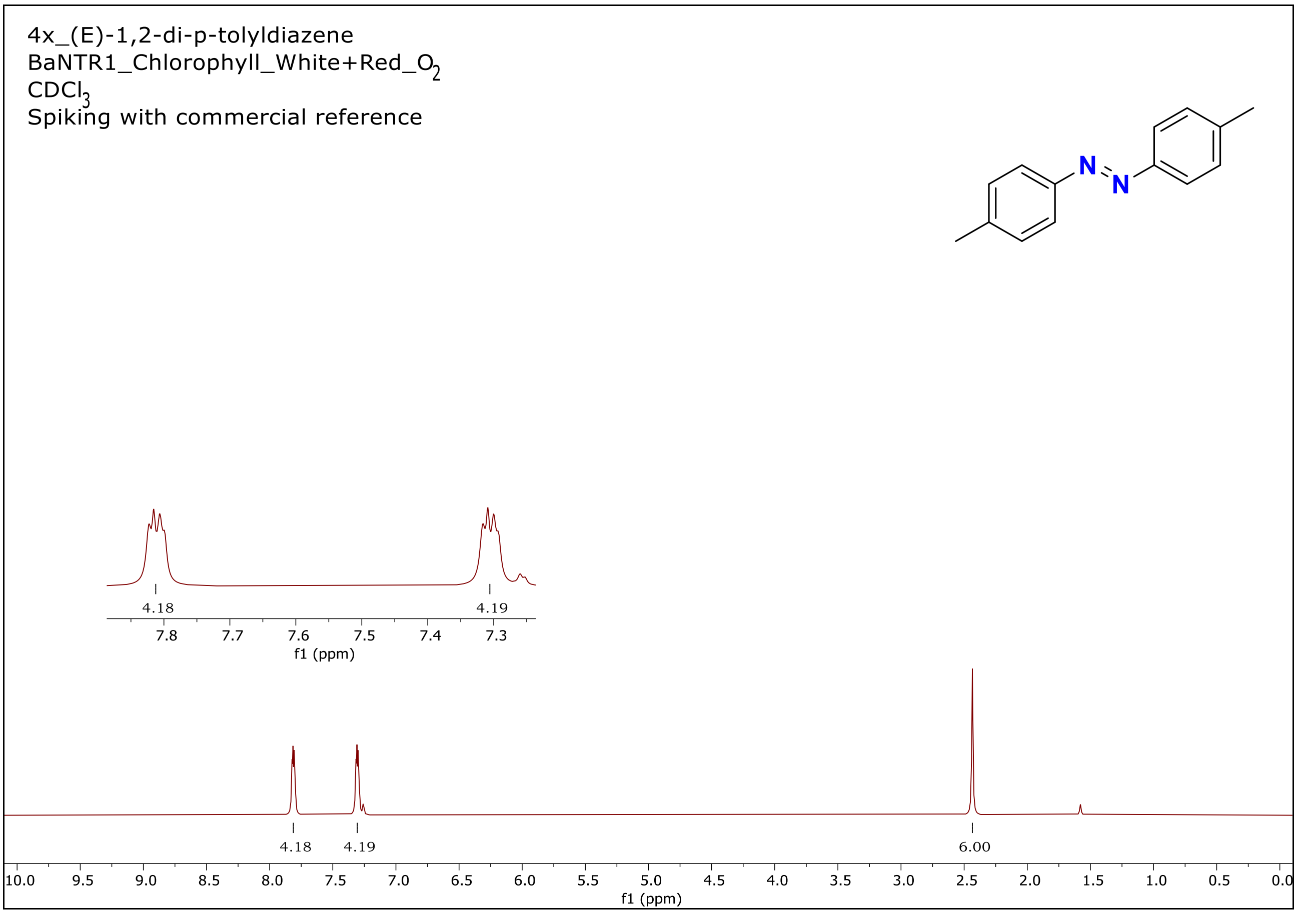

Supplementary Figure 110. Stacking (top) and spiking (bottom) ^{1}H-NMR spectrum of the photoenzymatic product **63** with a commercial reference.

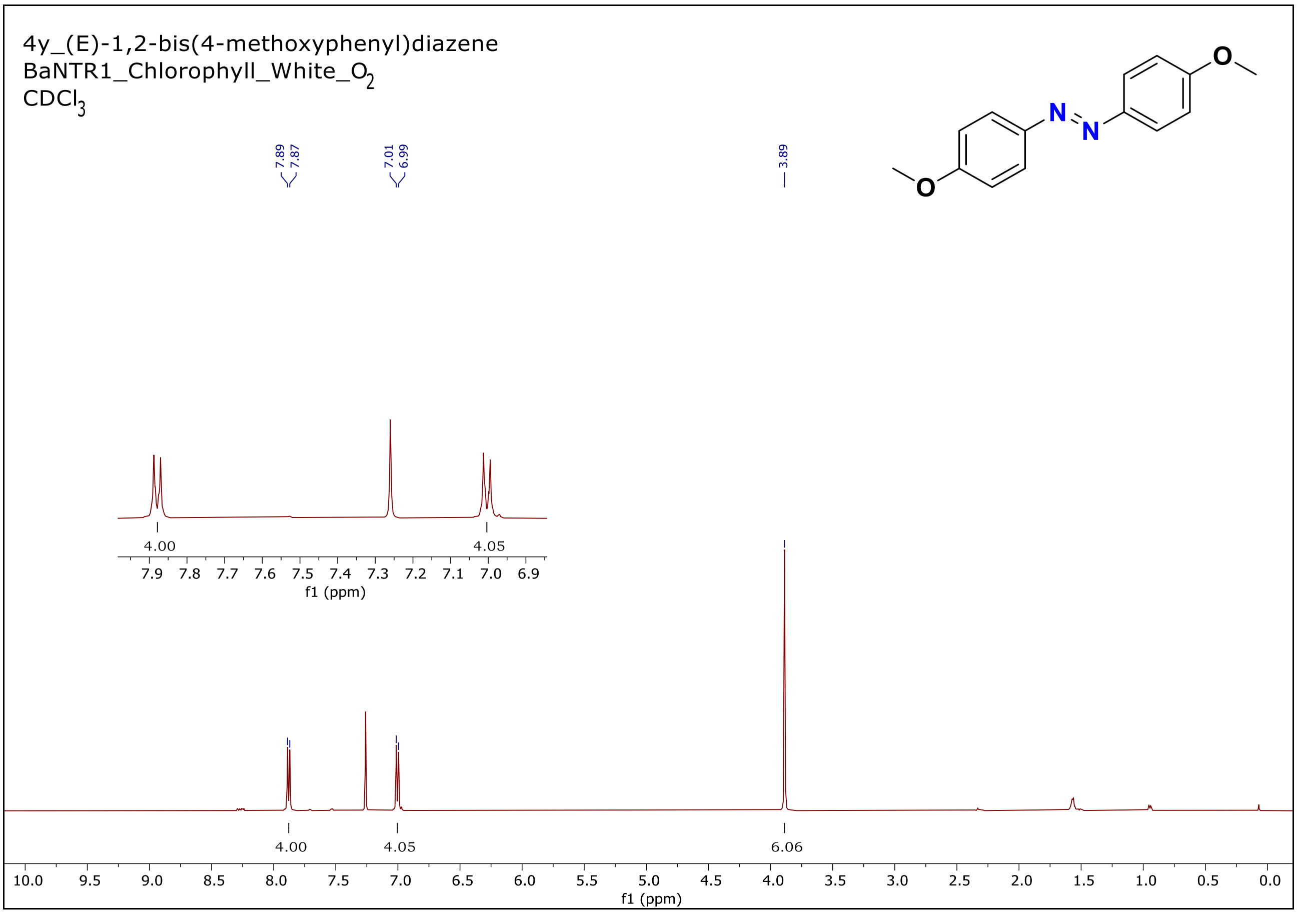

Supplementary Figure 111. ^{1}H-NMR of the photoenzymatic product **64.**

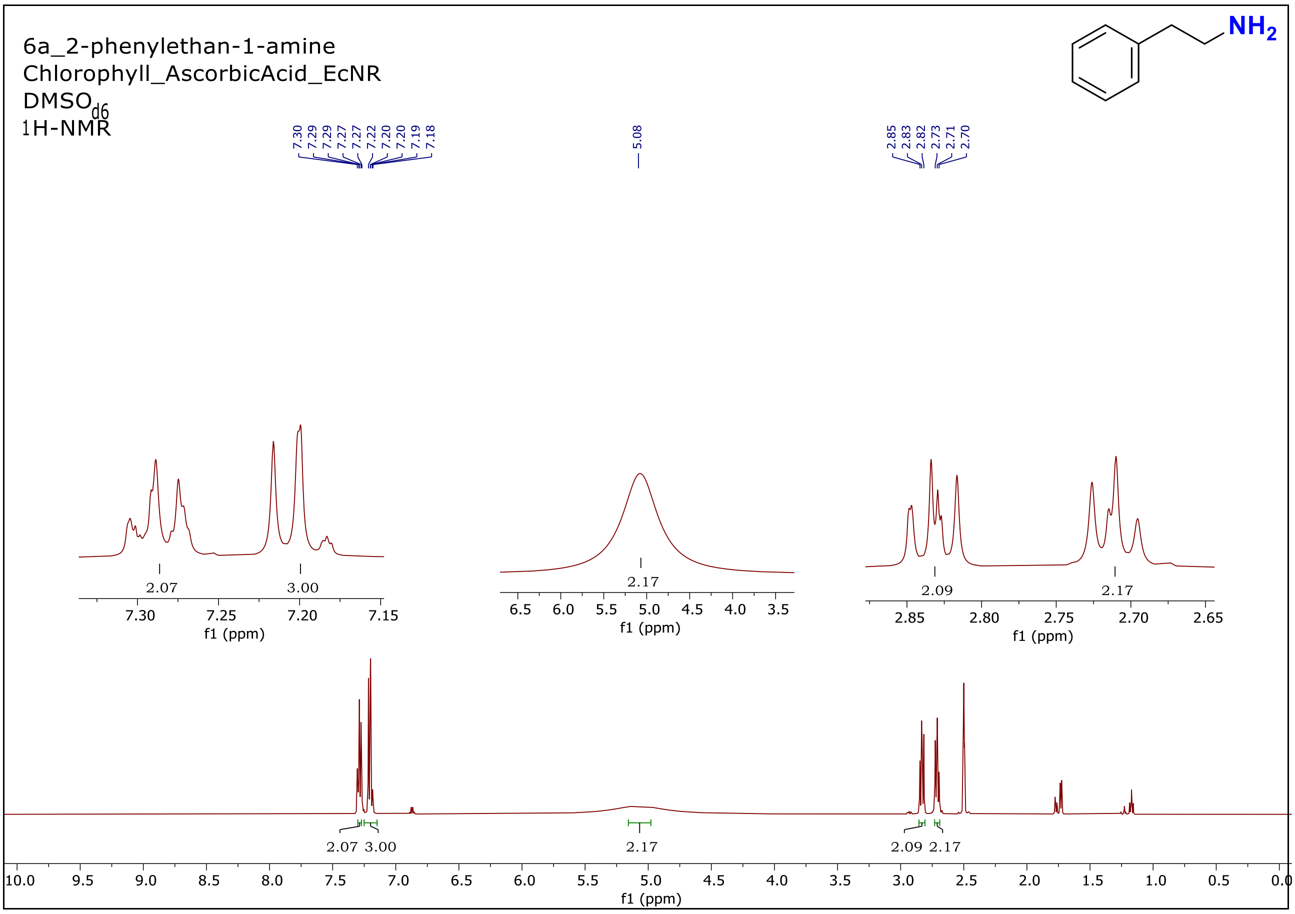

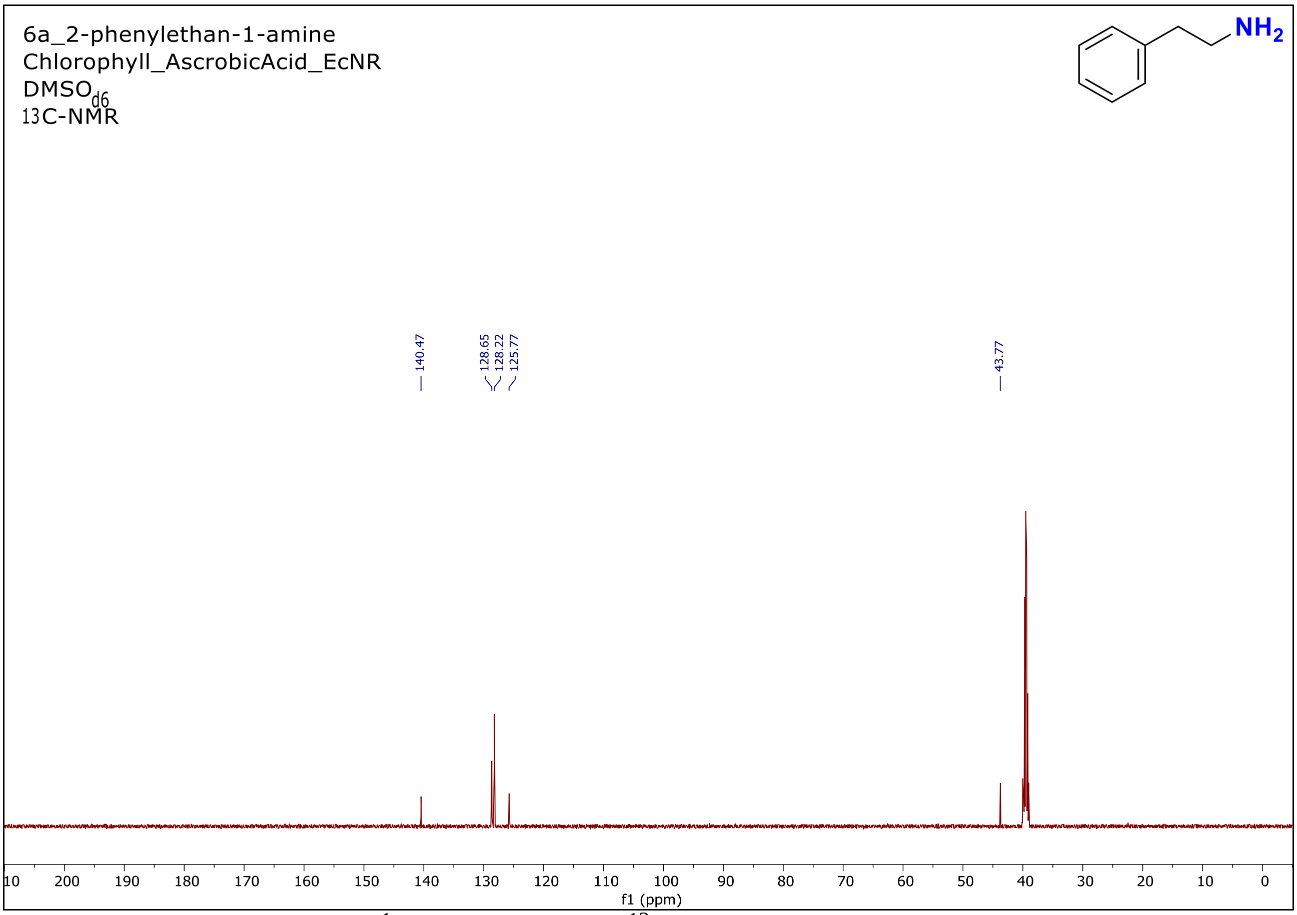

Supplementary Figure 112. ^{1}H-NMR (top) and ^{13}C-NMR (bottom) spectrum of the photoenzymatic product **68**.

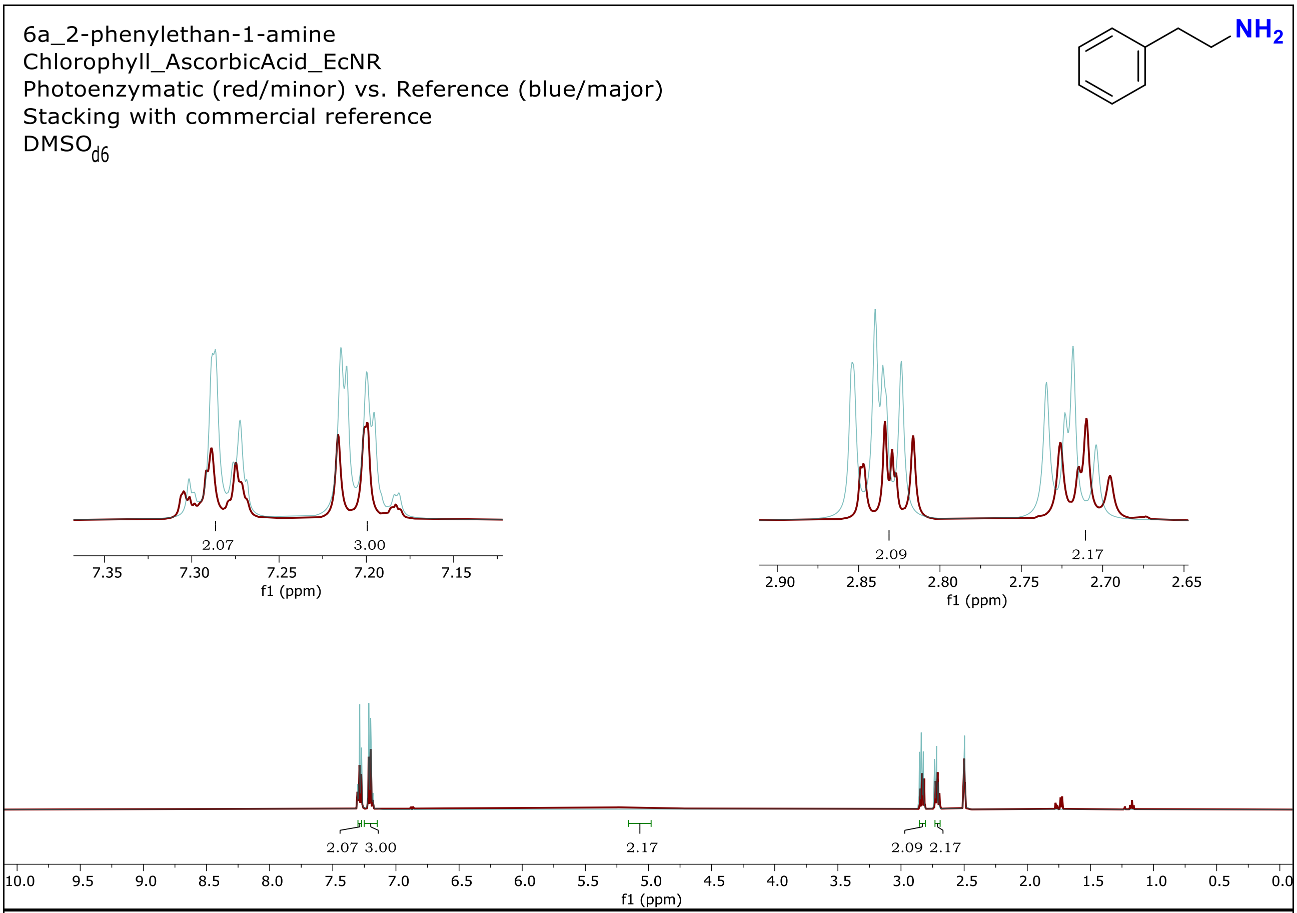

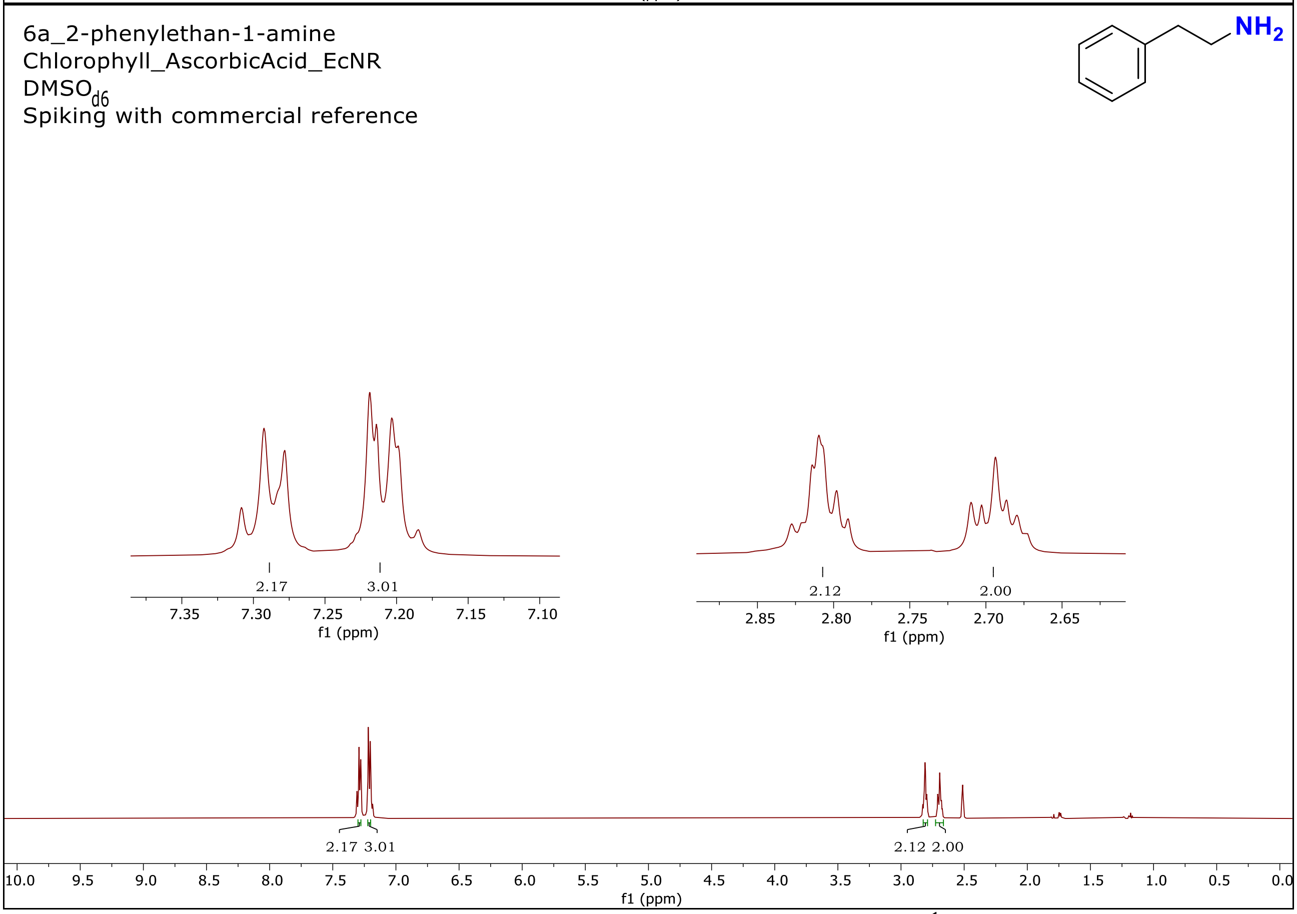

Supplementary Figure 113. Stacking (top) and spiking (bottom) ^{1}H-NMR spectrum with a commercial reference of the photoenzymatic product **68**.

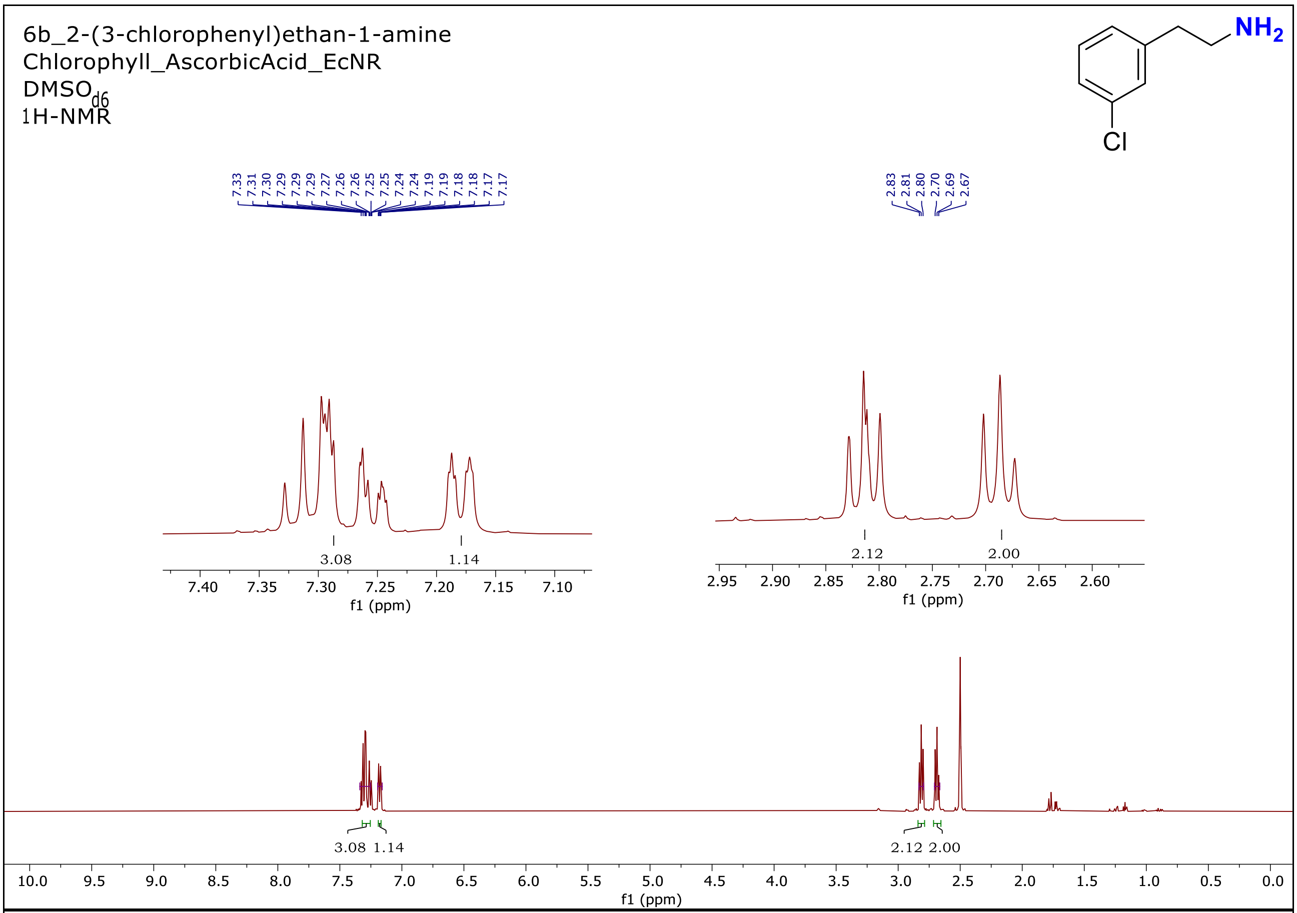

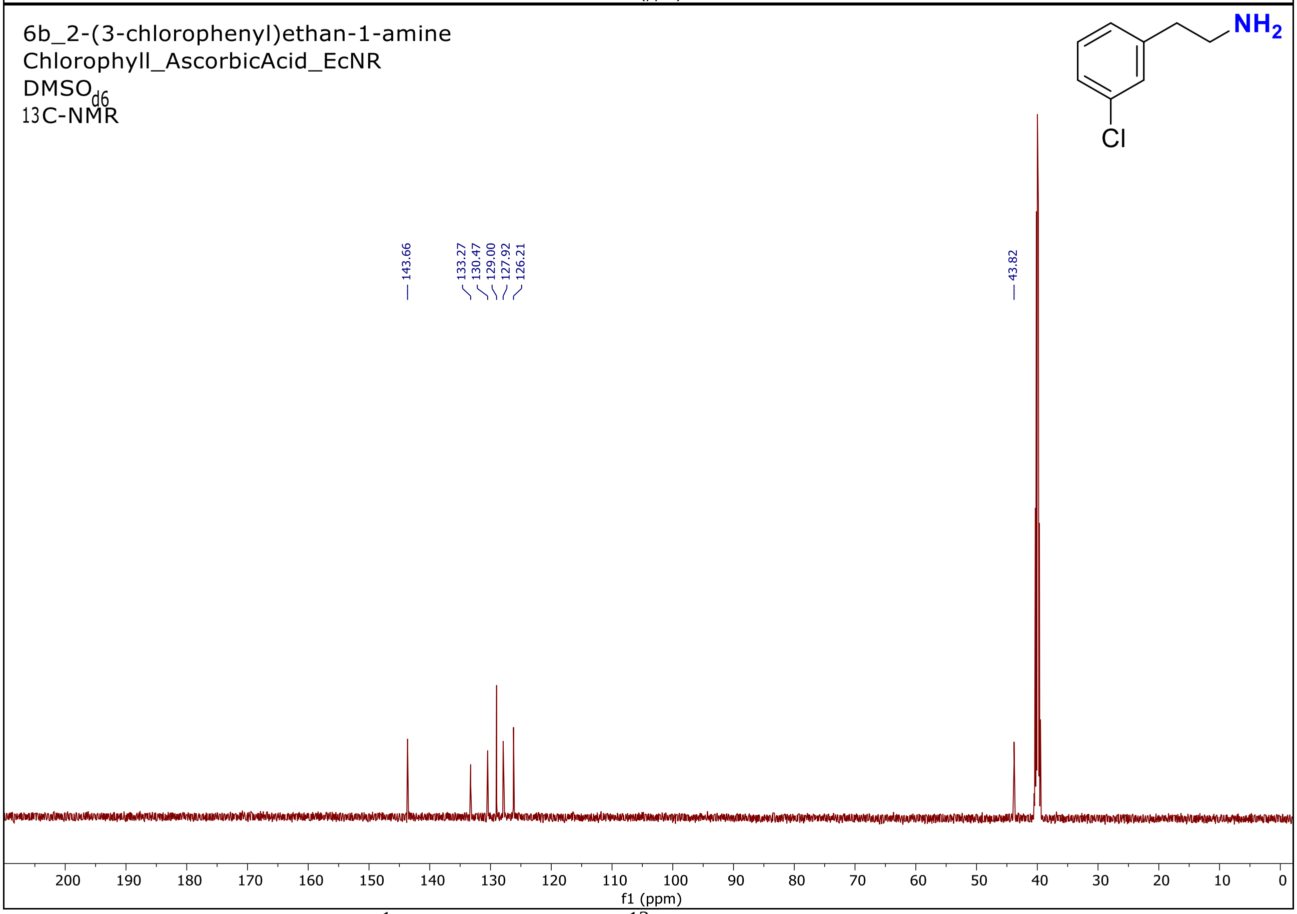

Supplementary Figure 114. ^{1}H-NMR (top) and ^{13}C-NMR (bottom) spectrum of the photoenzymatic product **69**.

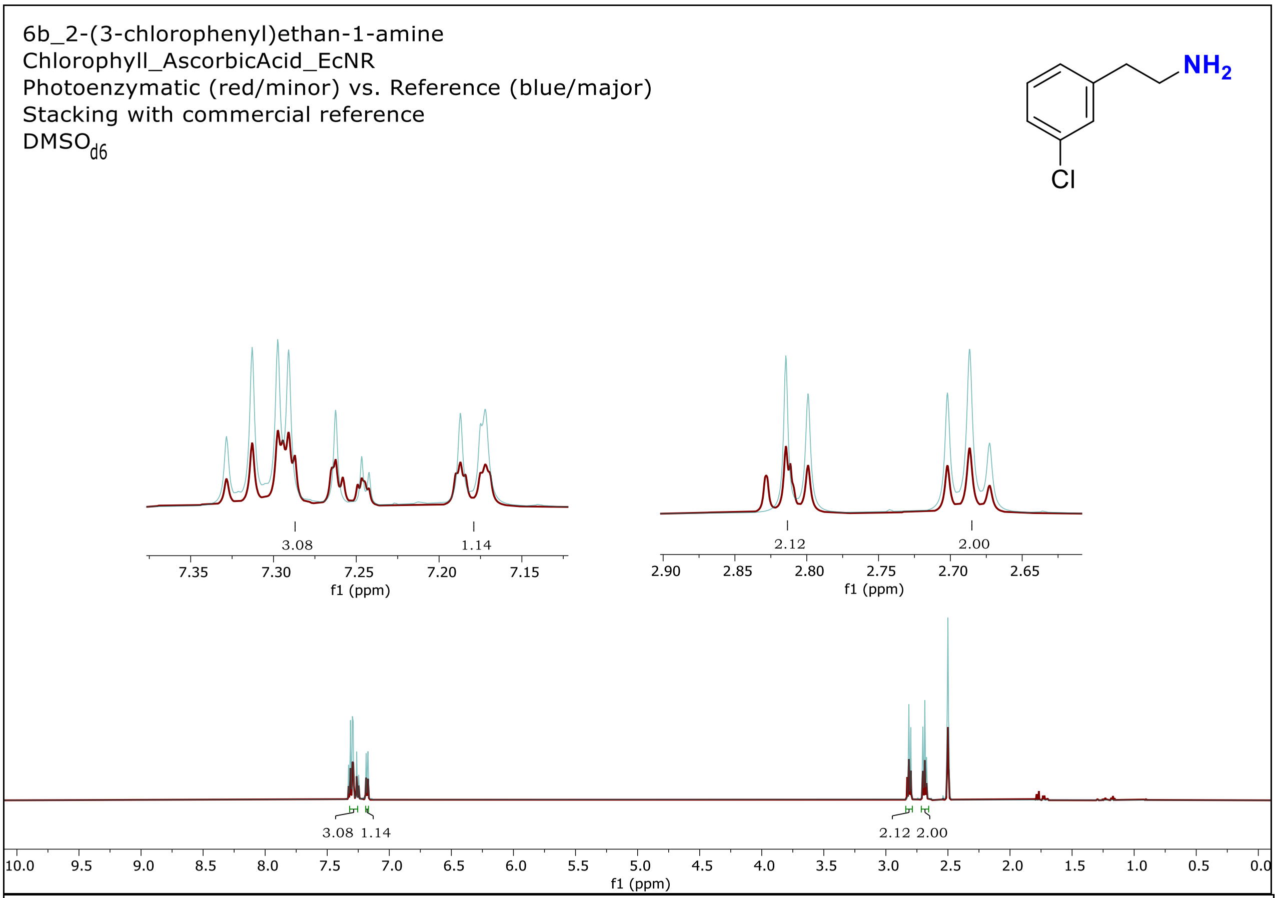

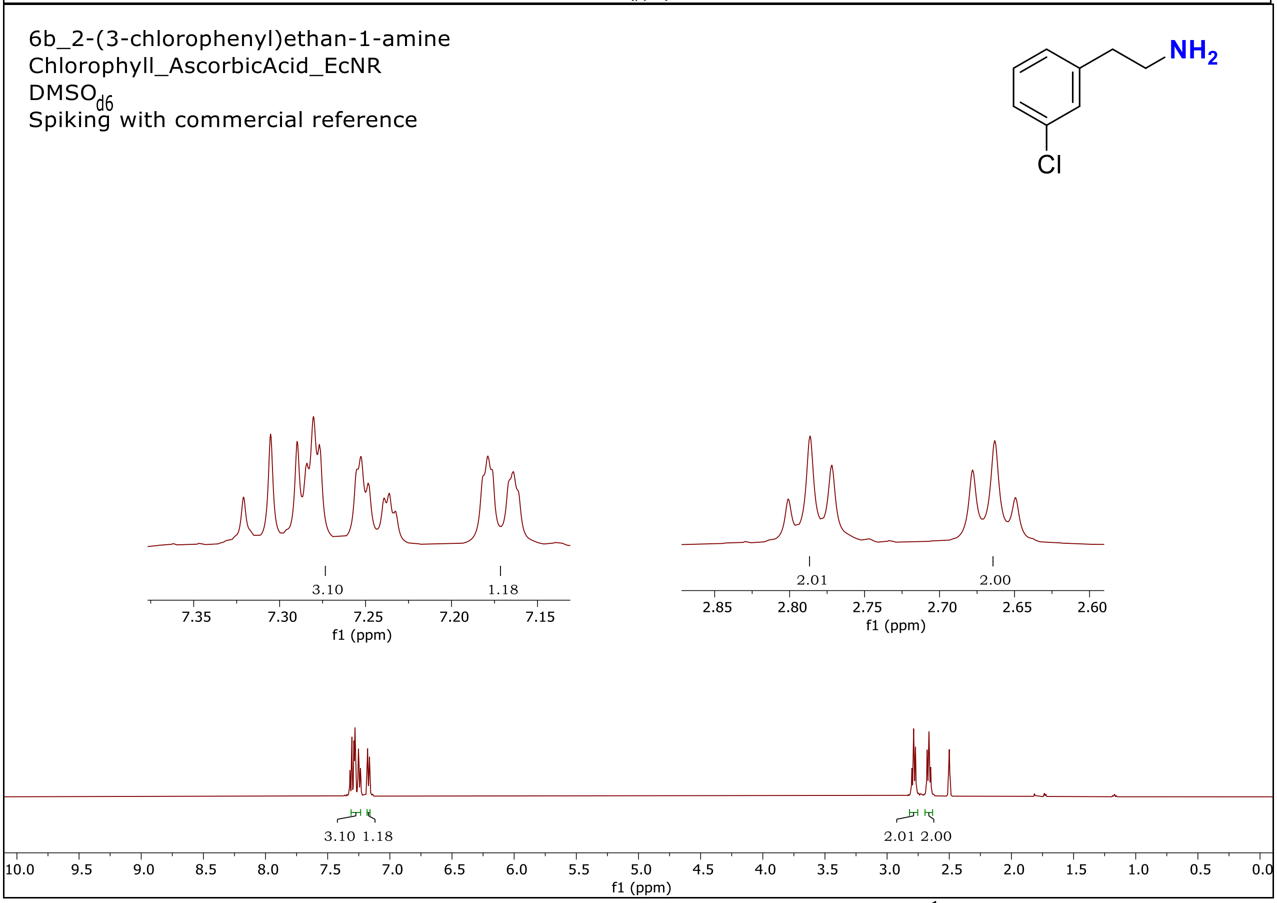

Supplementary Figure 115. Stacking (top) and spiking (bottom) ^{1}H-NMR spectrum with a commercial reference of the photoenzymatic product **69**.

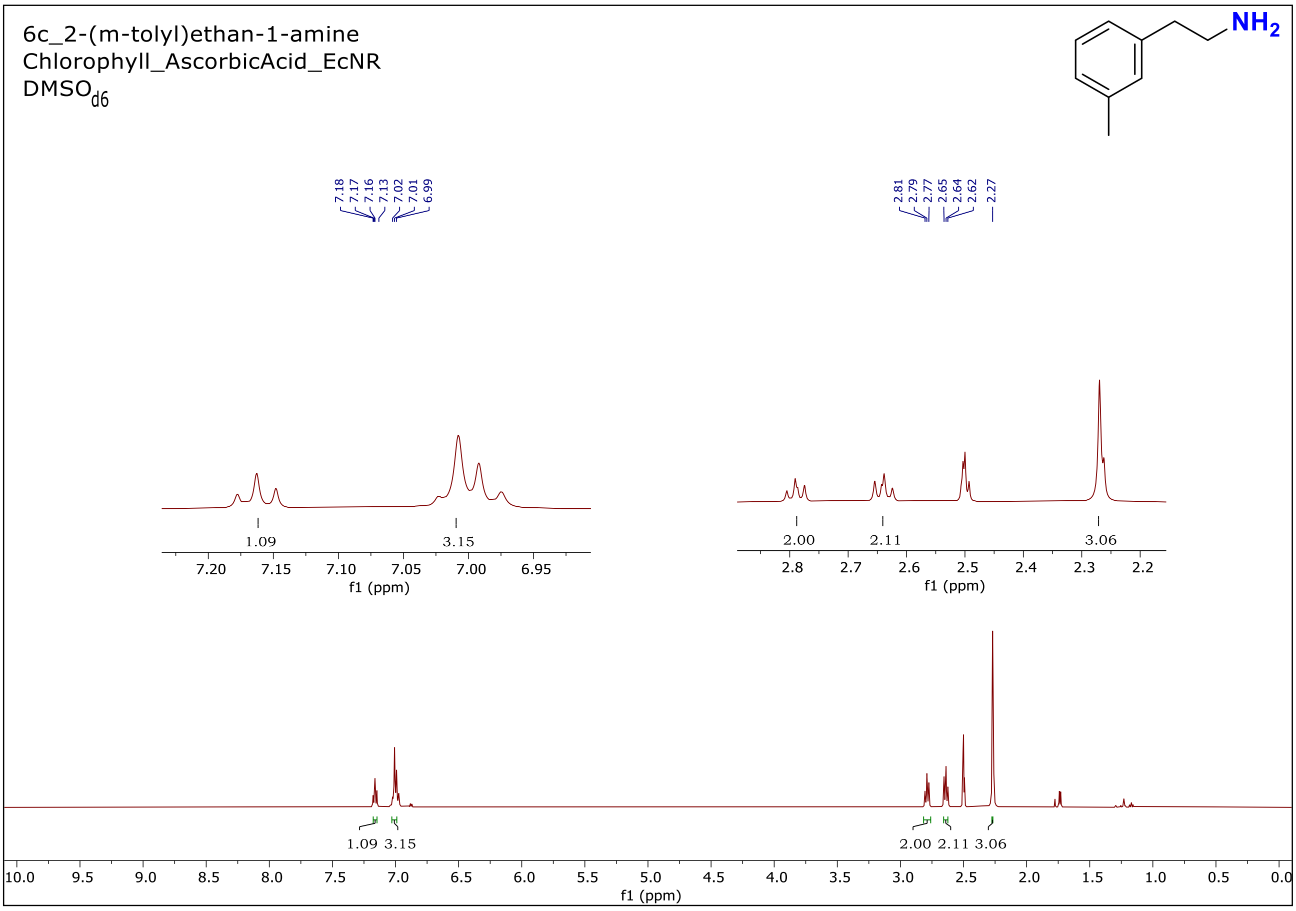

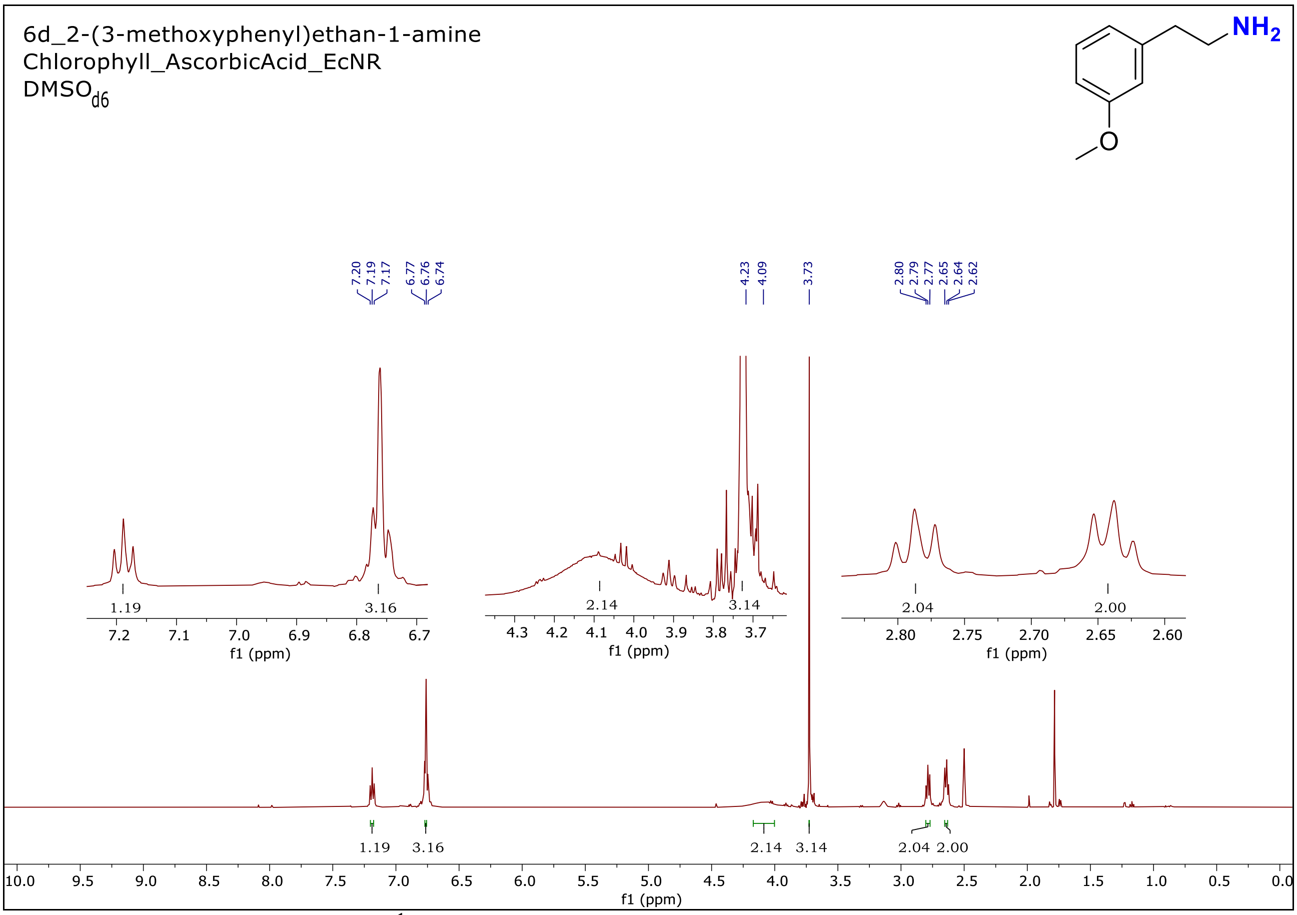

Supplementary Figure 116. ^{1}H-NMR spectrum of the photoenzymatic products **70** (top) and **71** (bottom).

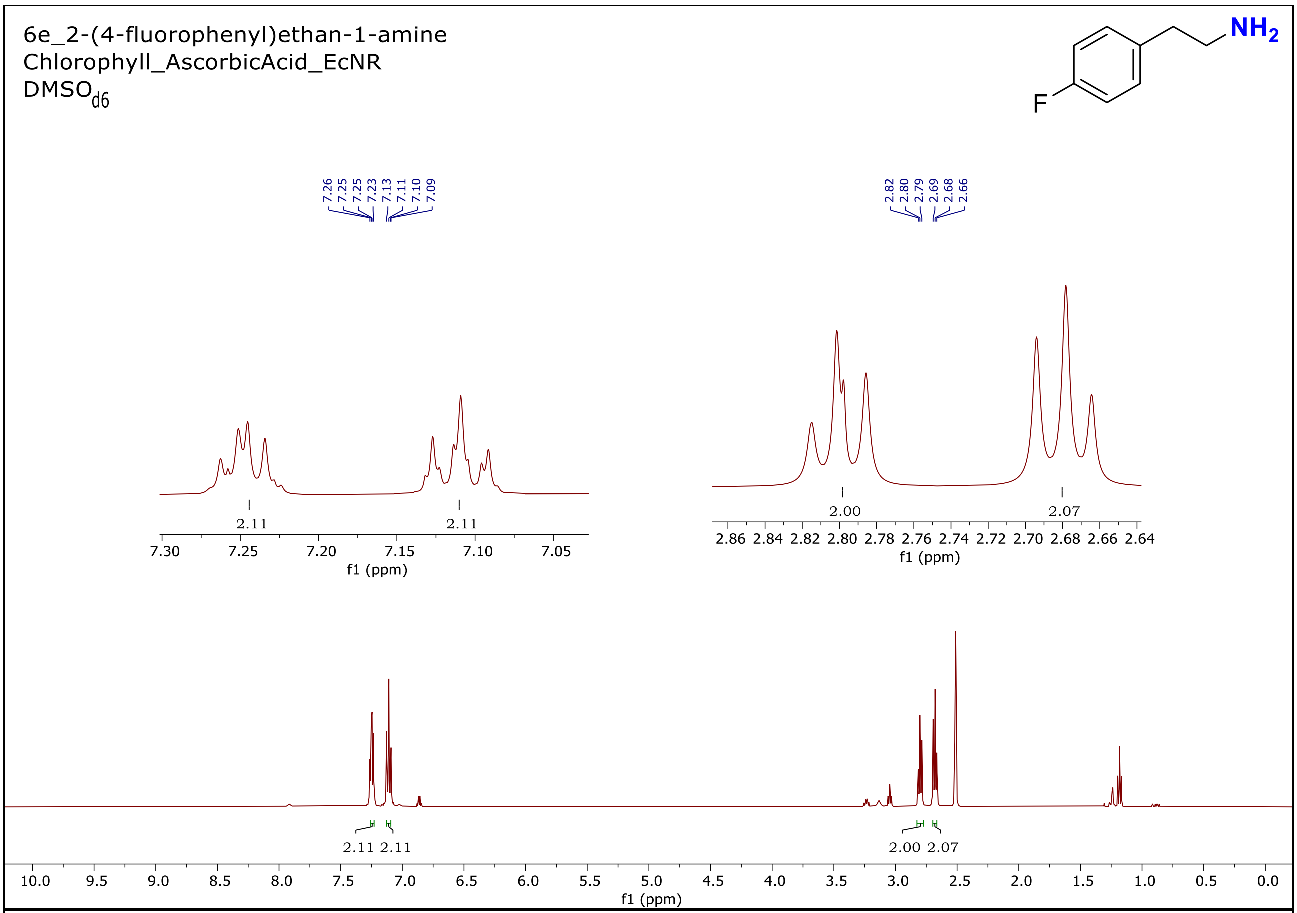

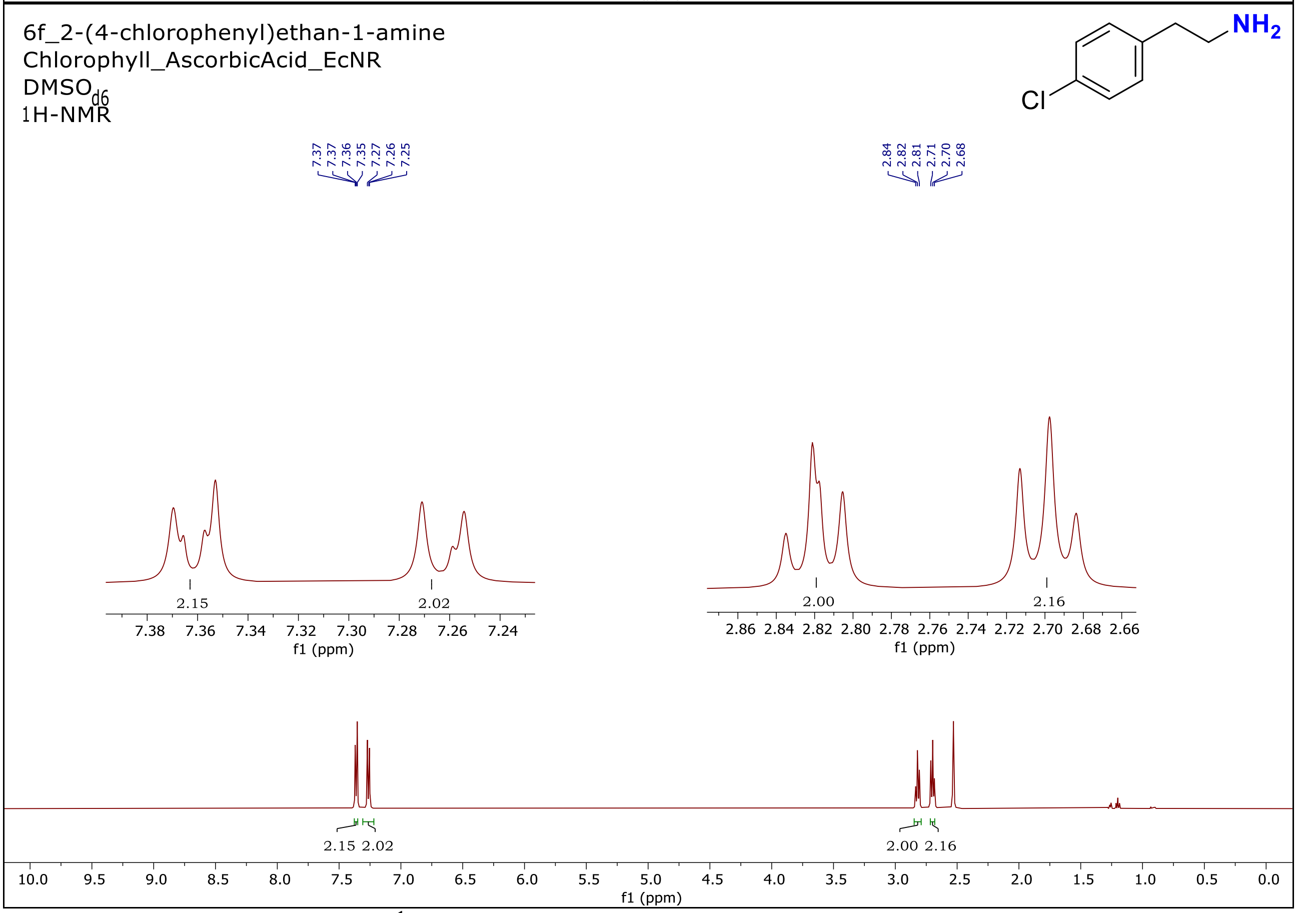

Supplementary Figure 117. ^{1}H-NMR spectrum of the photoenzymatic products **72** (top) and **73** (bottom).

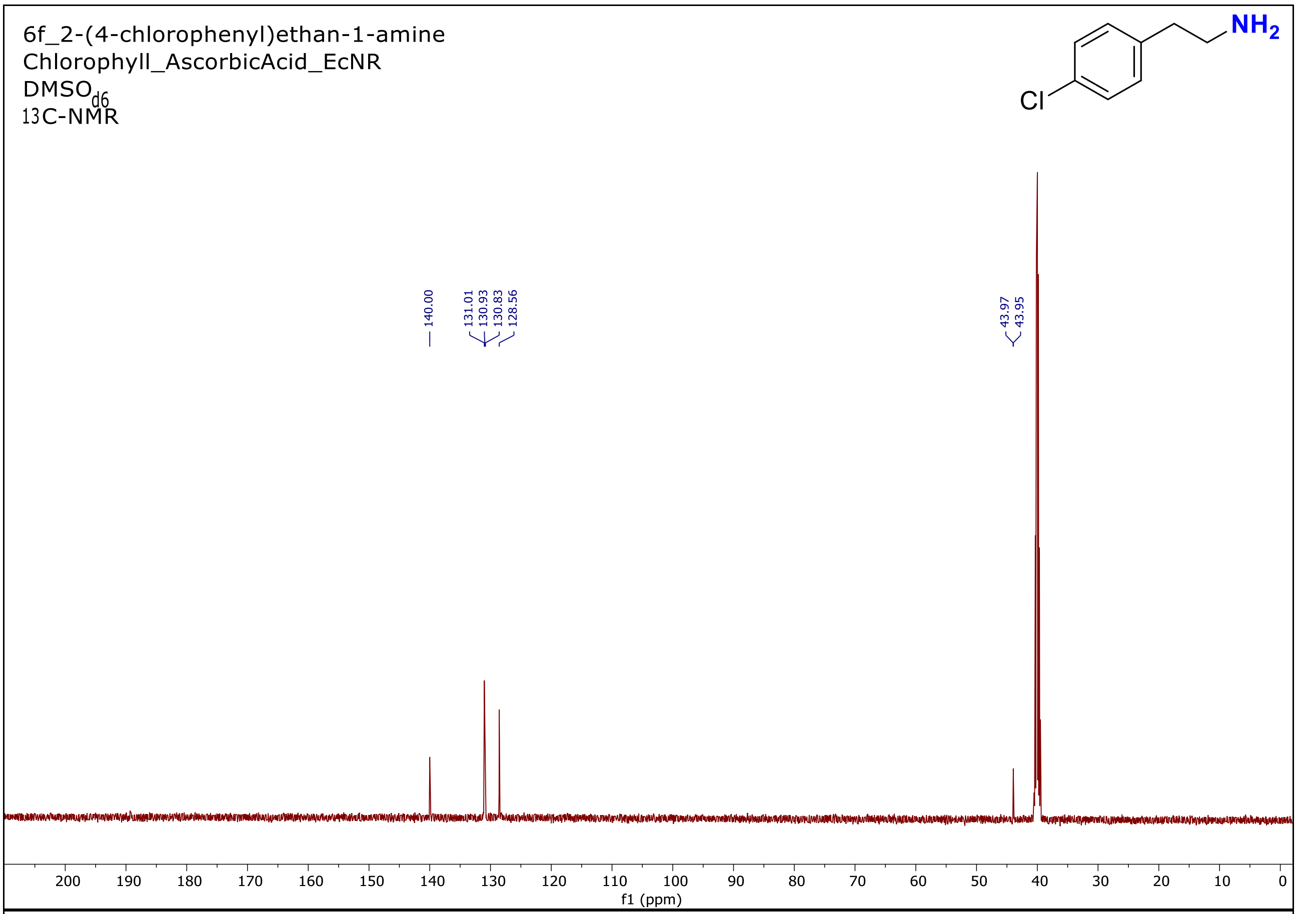

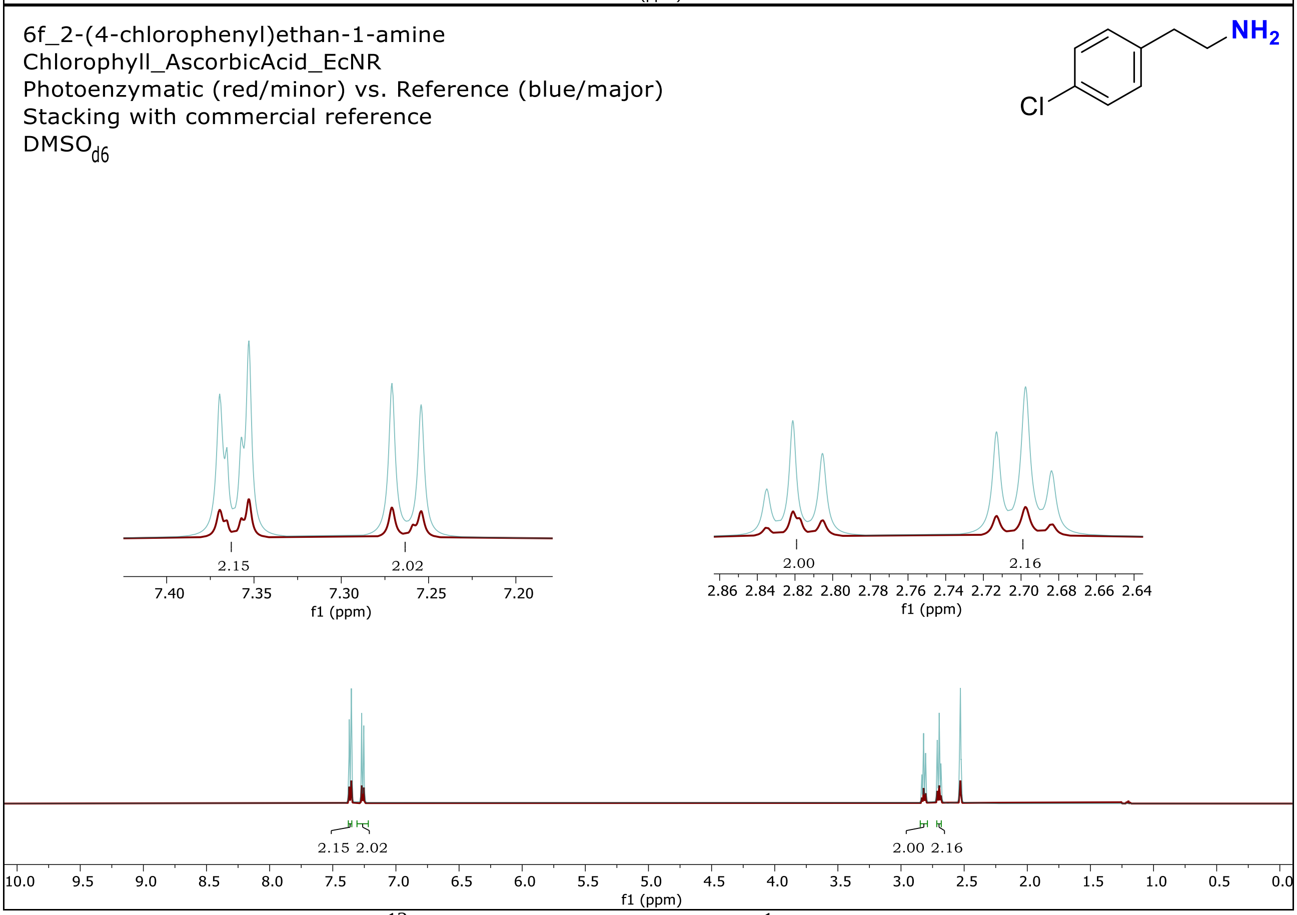

Supplementary Figure 118. ^{13}C-NMR (top) and stacking ^{1}H-NMR spectrum against a commercial reference (bottom) of the photoenzymatic product **73**.

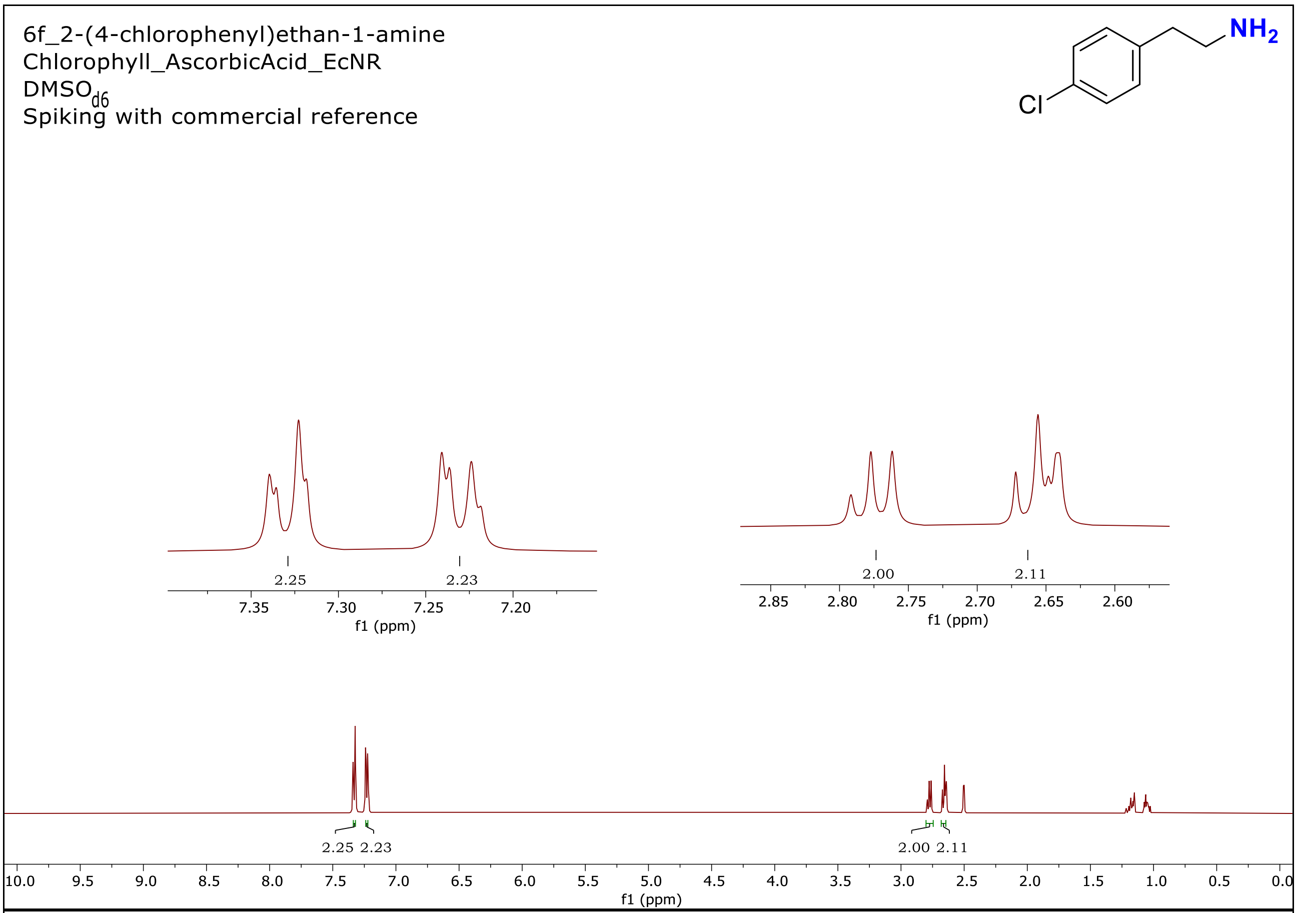

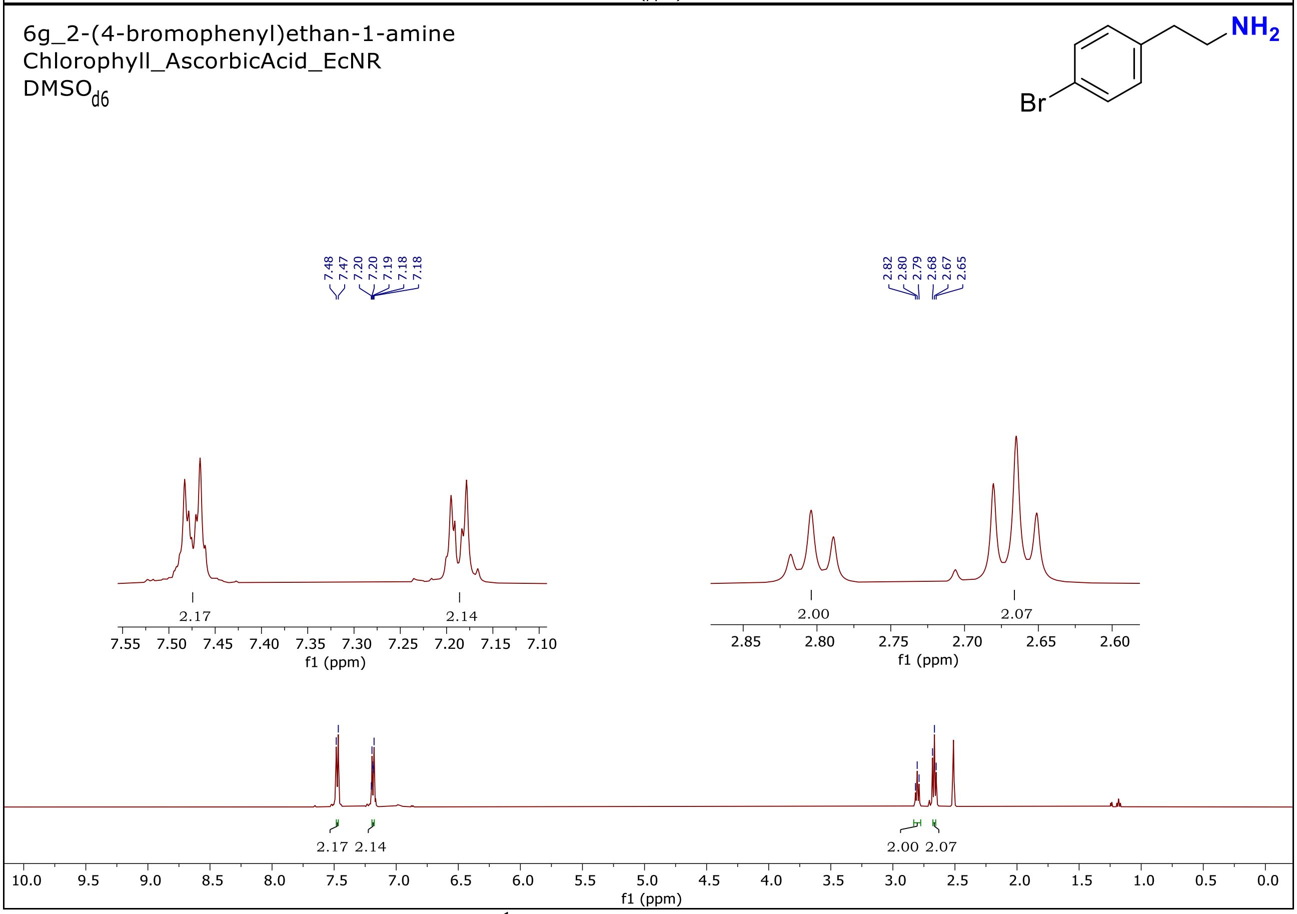

Supplementary Figure 119. Spiking ^{1}H-NMR spectrum with a commercial reference (top) of the photoenzymatic product **73** and ^{1}H-NMR (bottom) spectrum of the photoenzymatic product **74**.

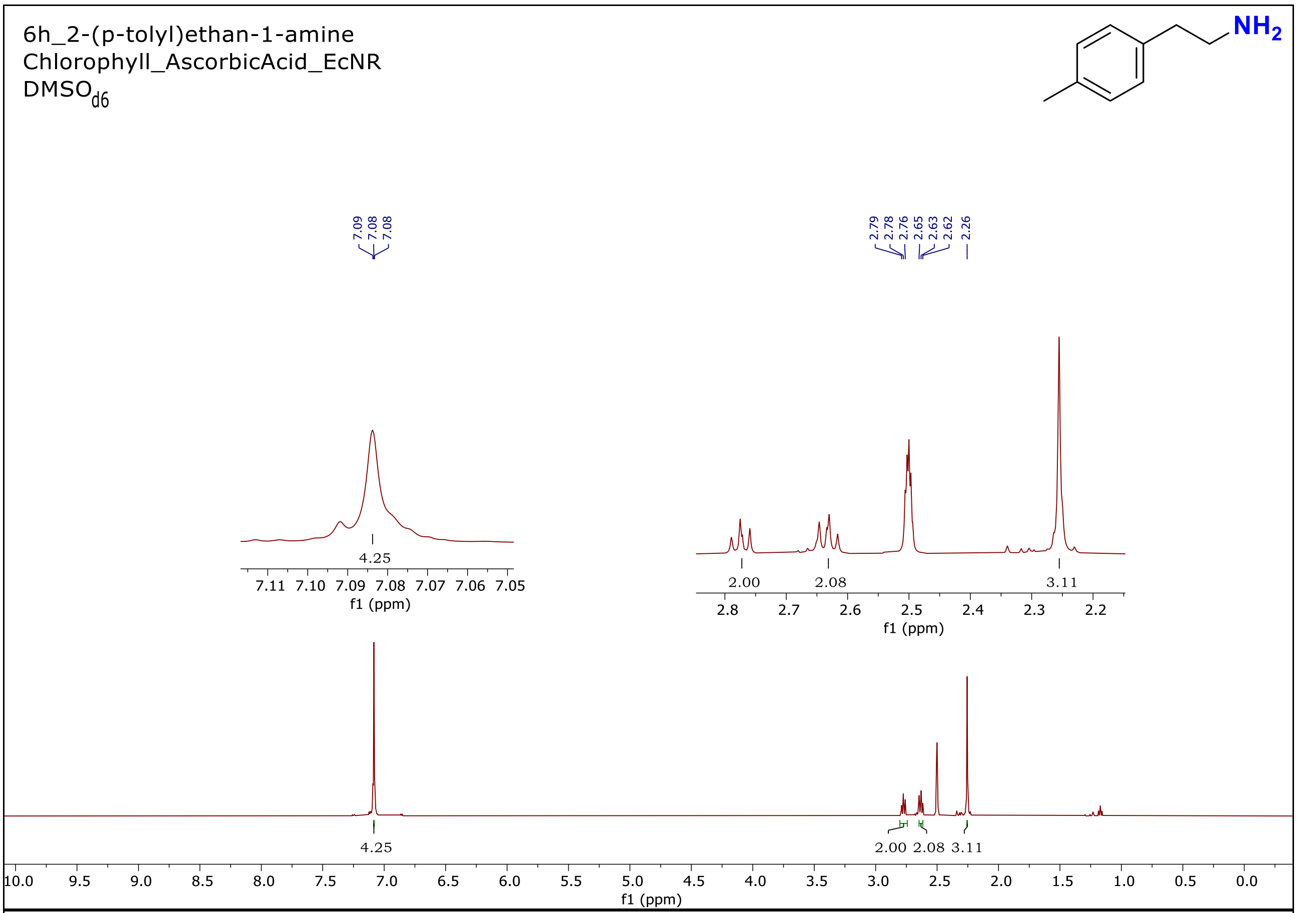

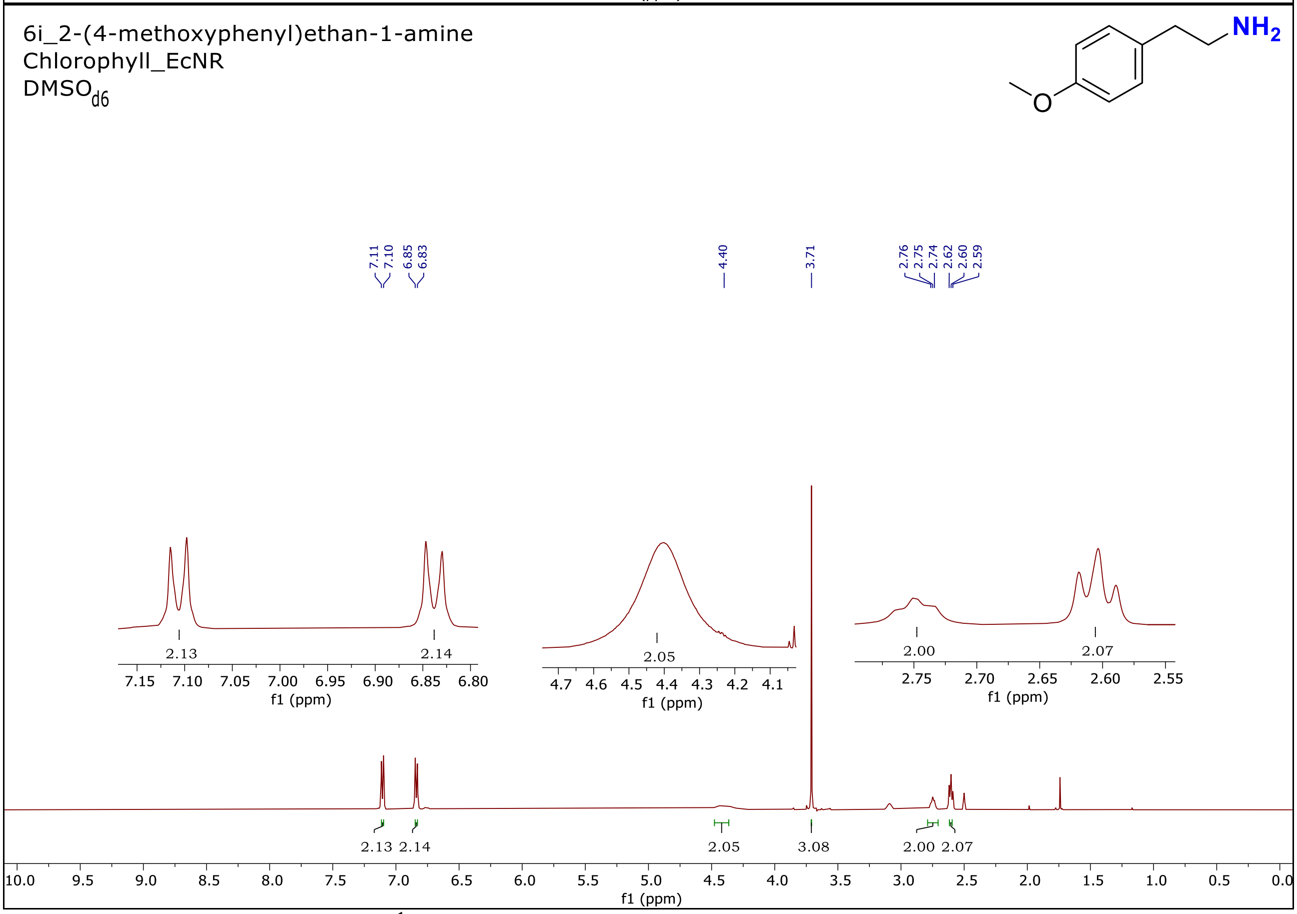

Supplementary Figure 120. ^{1}H-NMR spectrum of the photoenzymatic products **75** (top) and **76** (bottom).

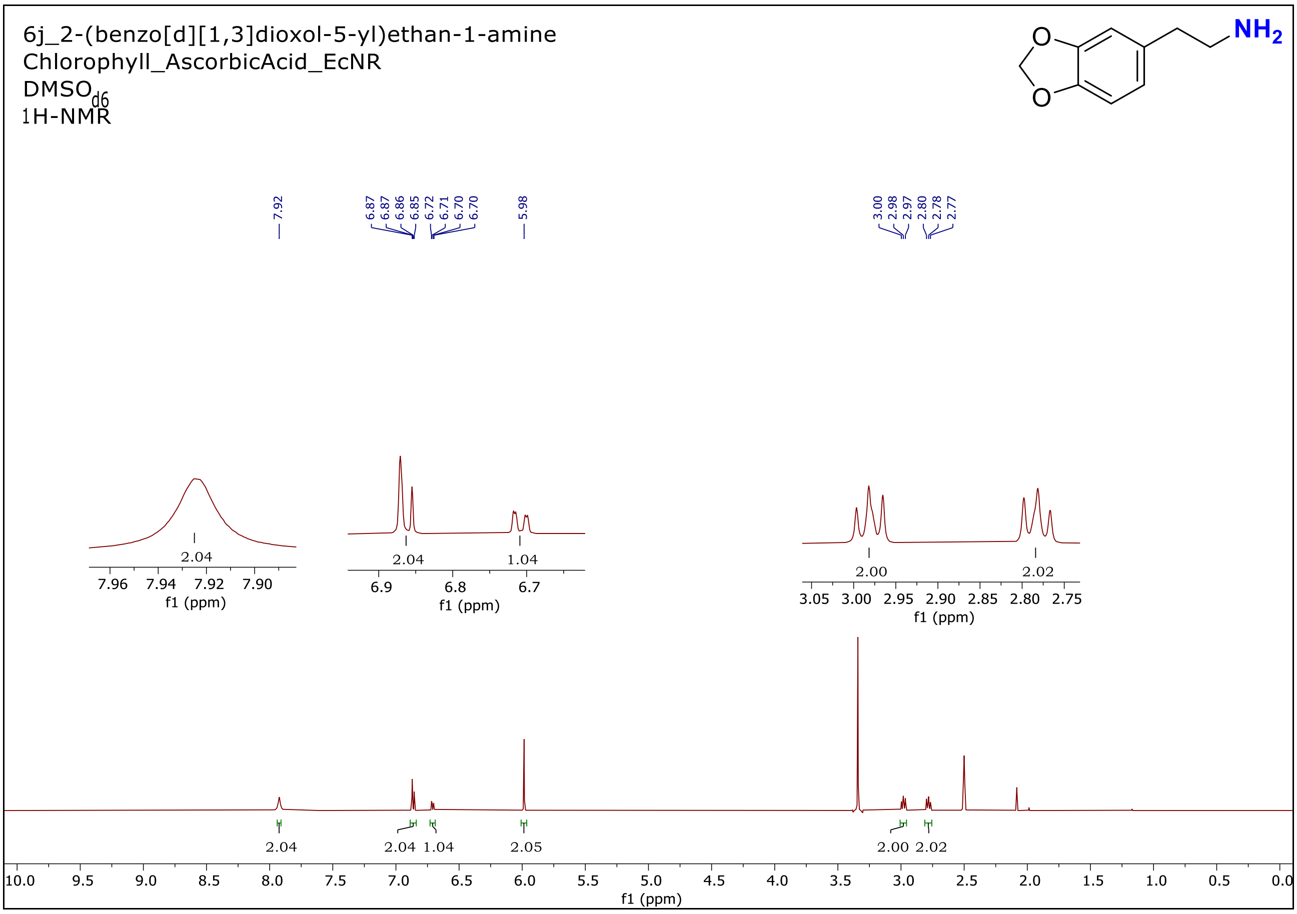

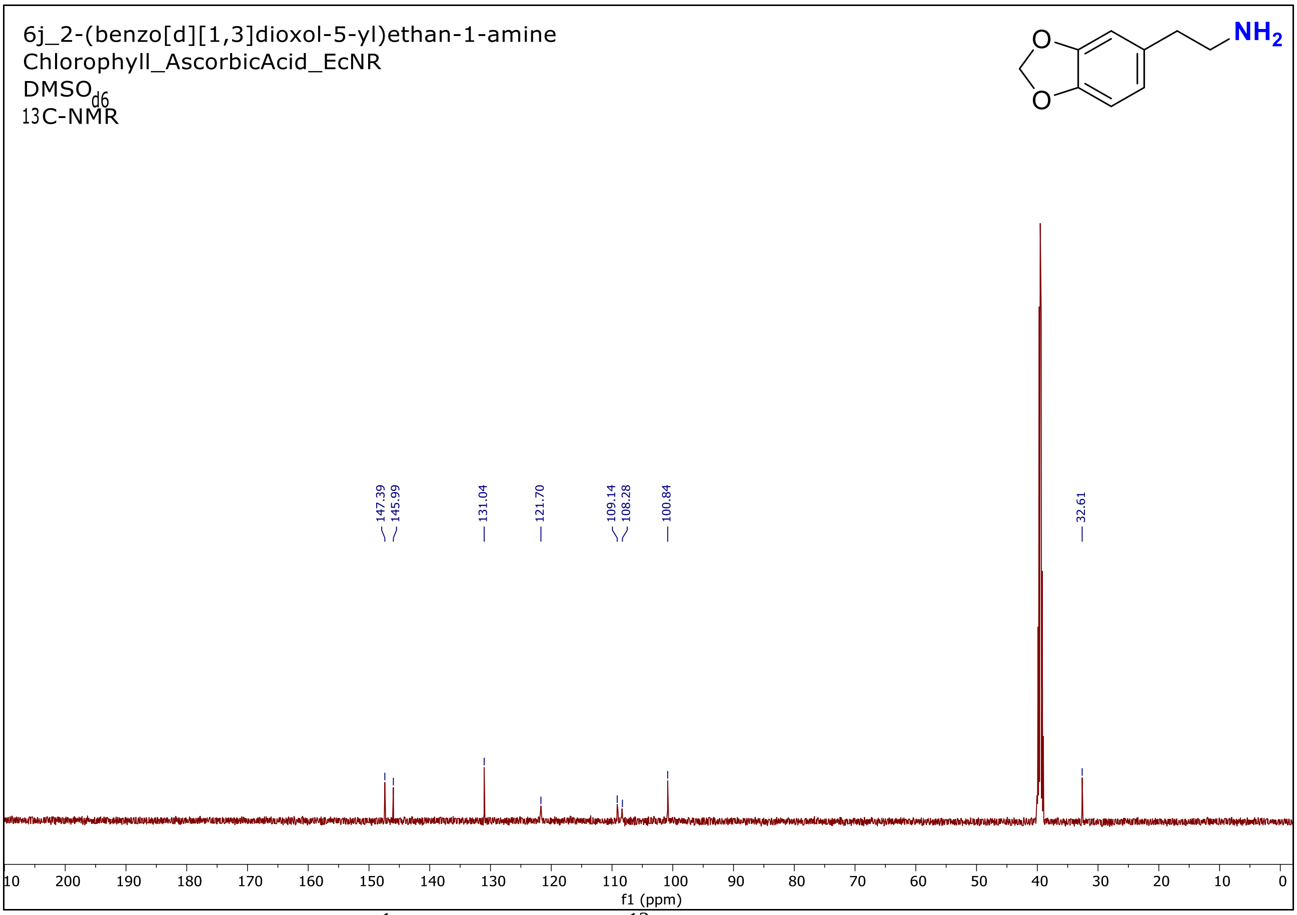

Supplementary Figure 121. ^{1}H-NMR (top) and ^{13}C-NMR (bottom) spectrum of the photoenzymatic product **77**.

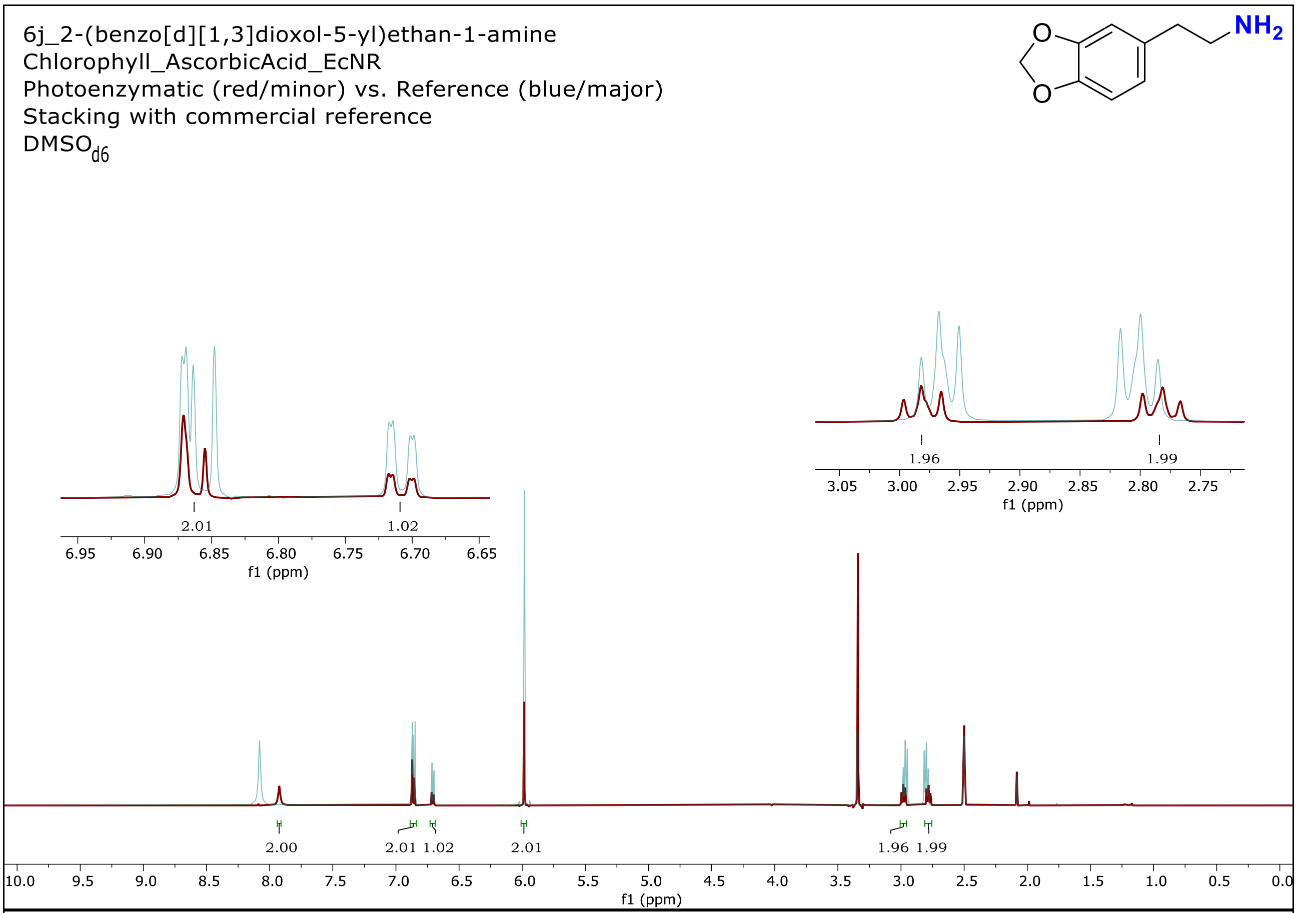

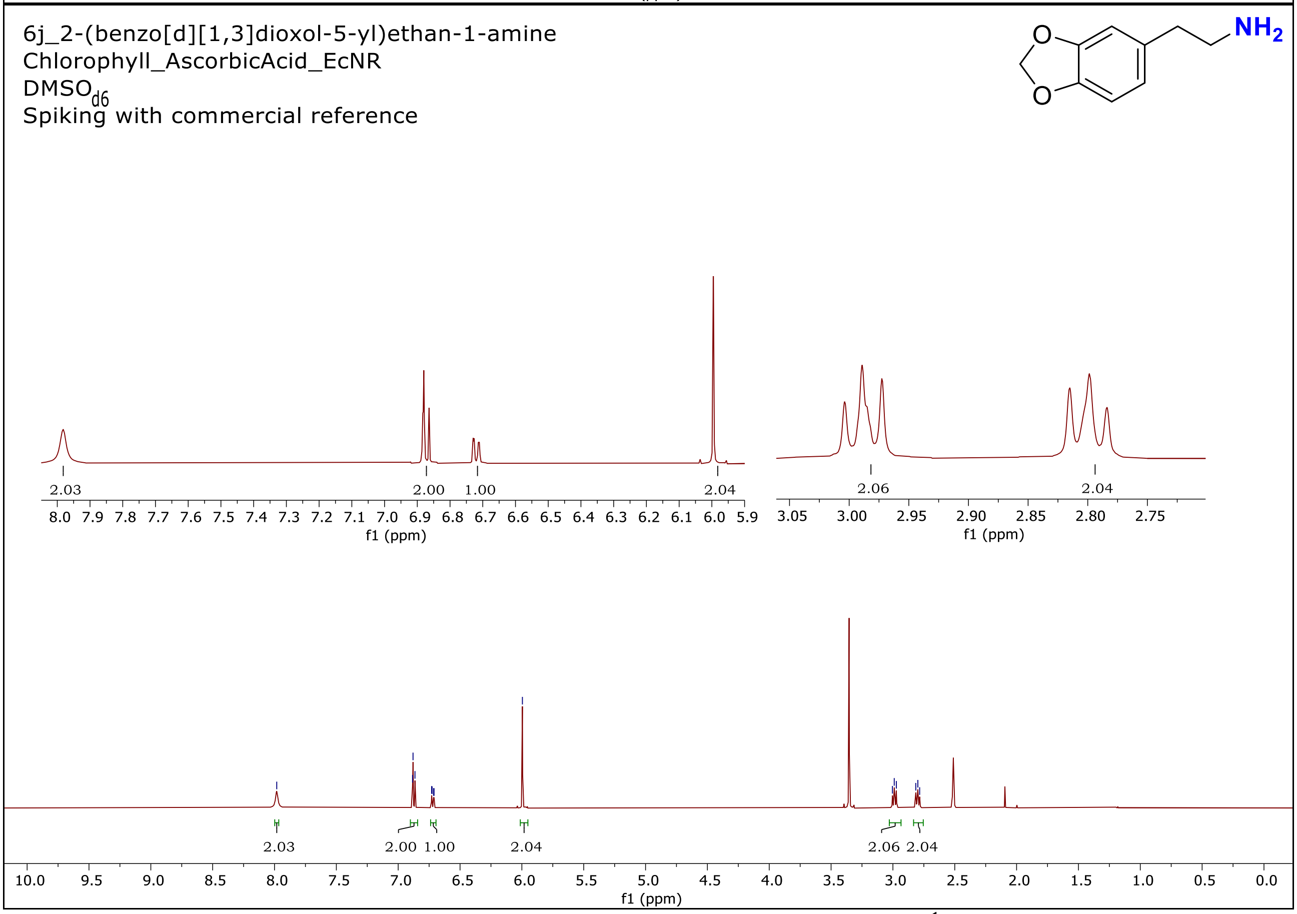

Supplementary Figure 122. Stacking (top) and spiking (bottom) ^{1}H-NMR spectrum with a commercial reference of the photoenzymatic product **77**.

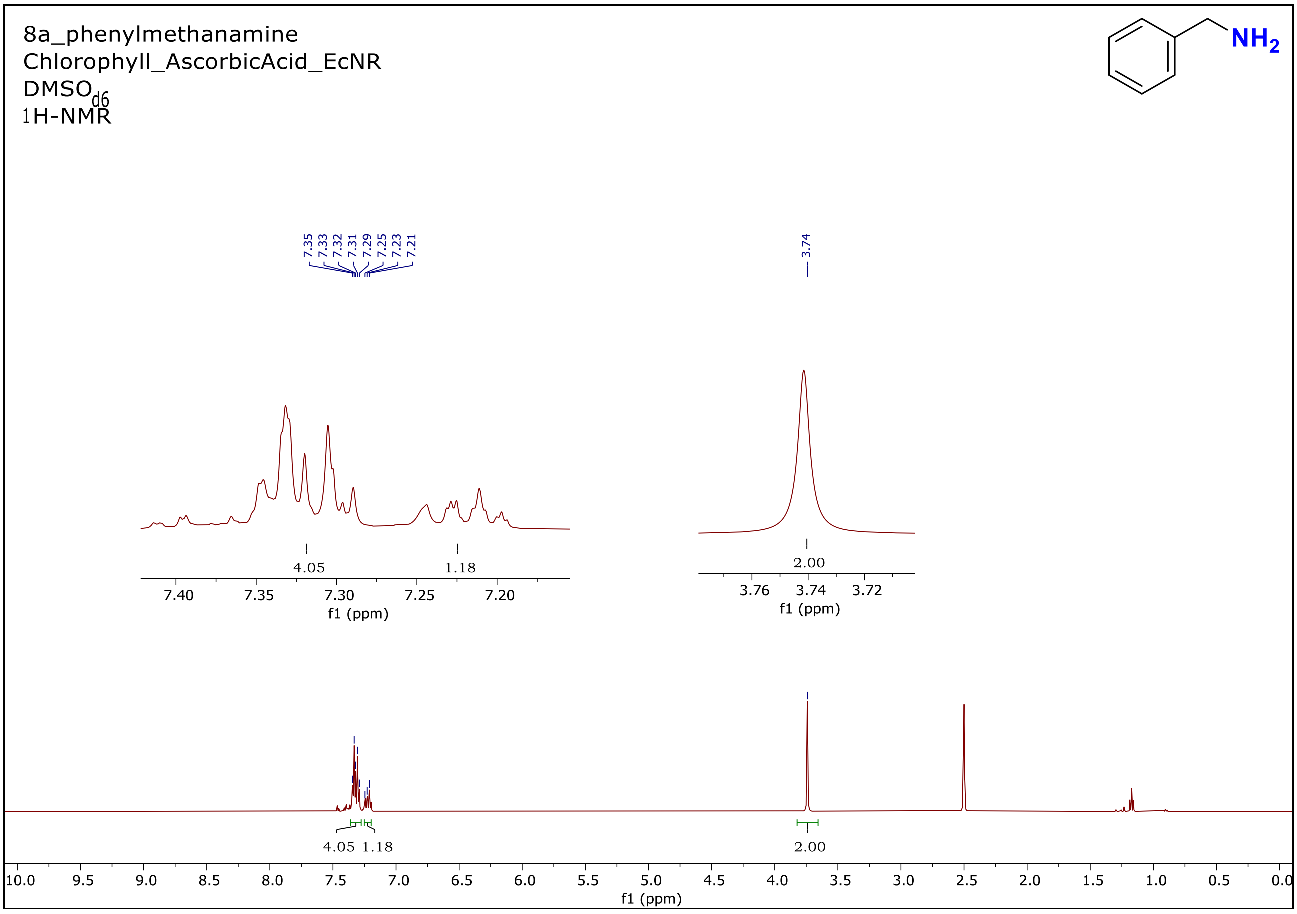

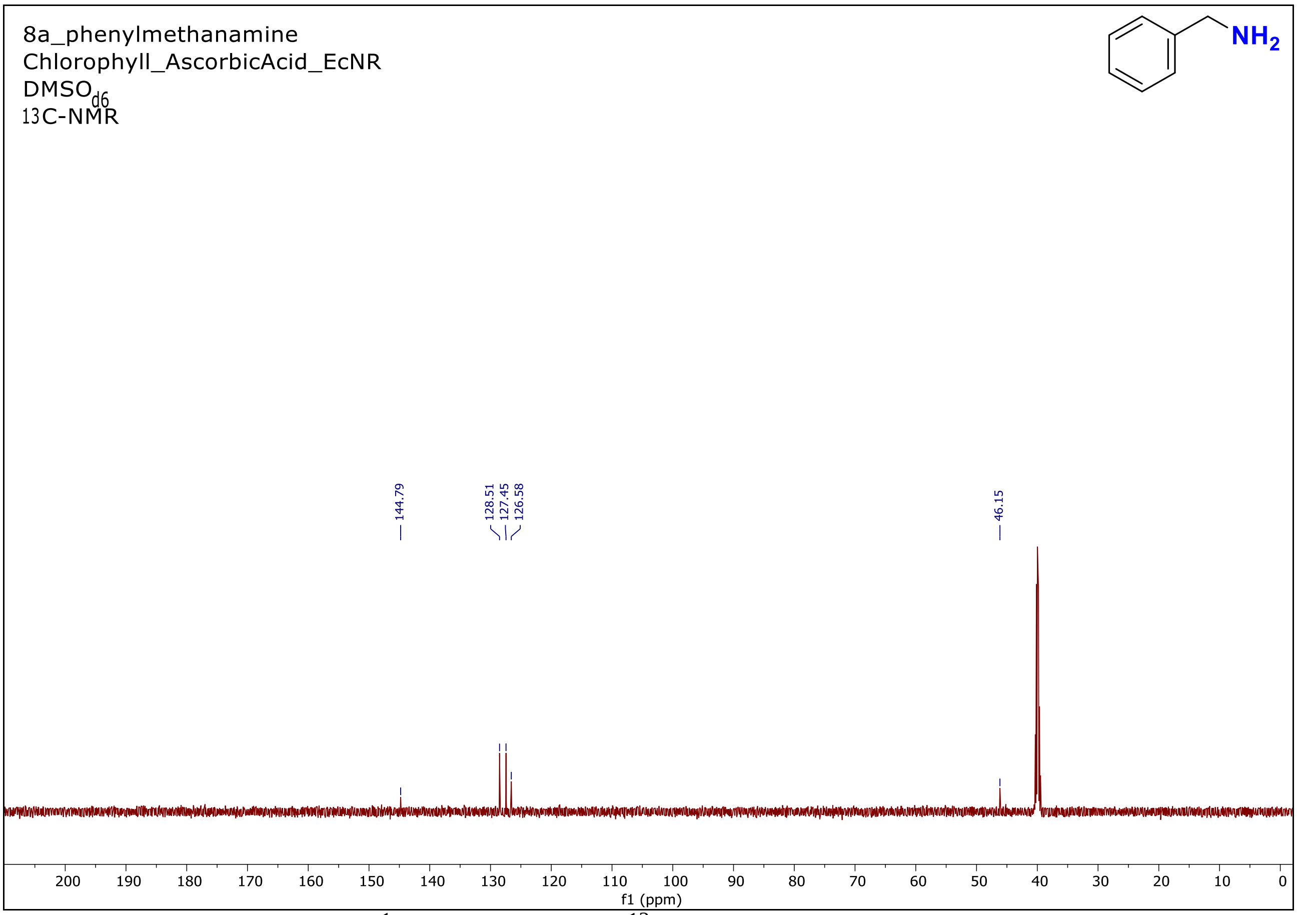

Supplementary Figure 123. ^{1}H-NMR (top) and ^{13}C-NMR (bottom) spectrum of the photoenzymatic product **78**.

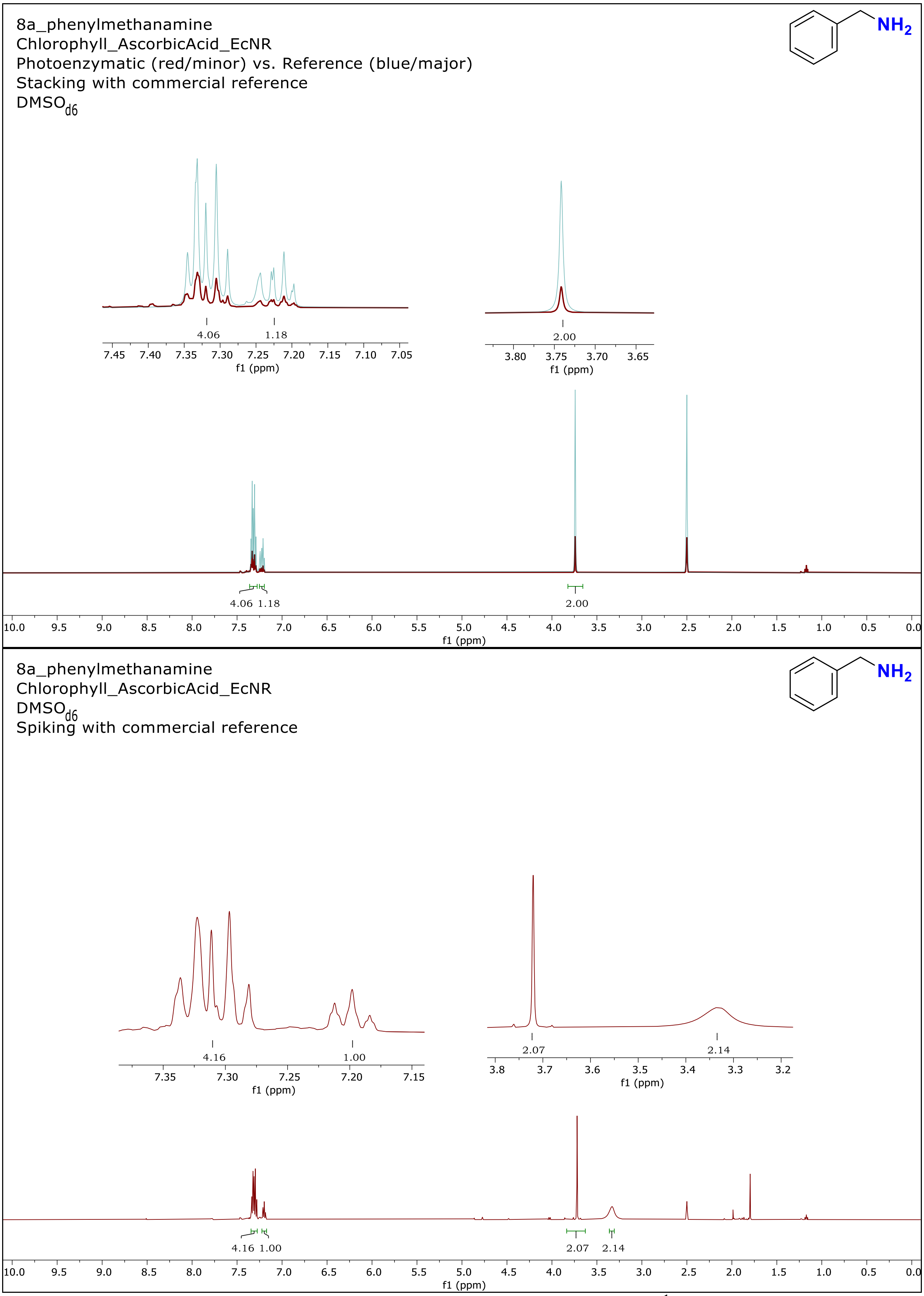

Supplementary Figure 124. Stacking (top) and spiking (bottom) ^{1}H-NMR spectrum with a commercial reference of the photoenzymatic product **78**.

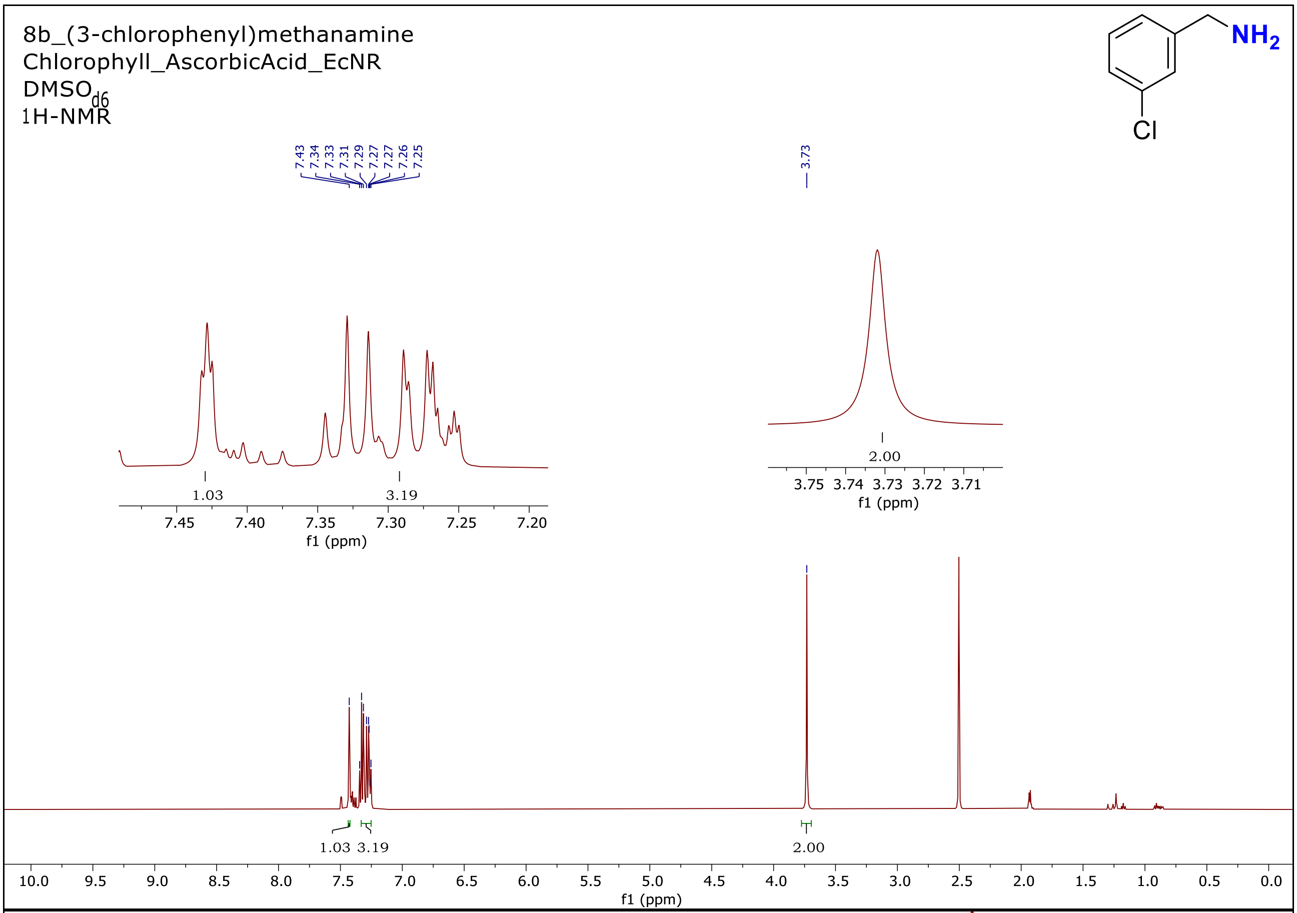

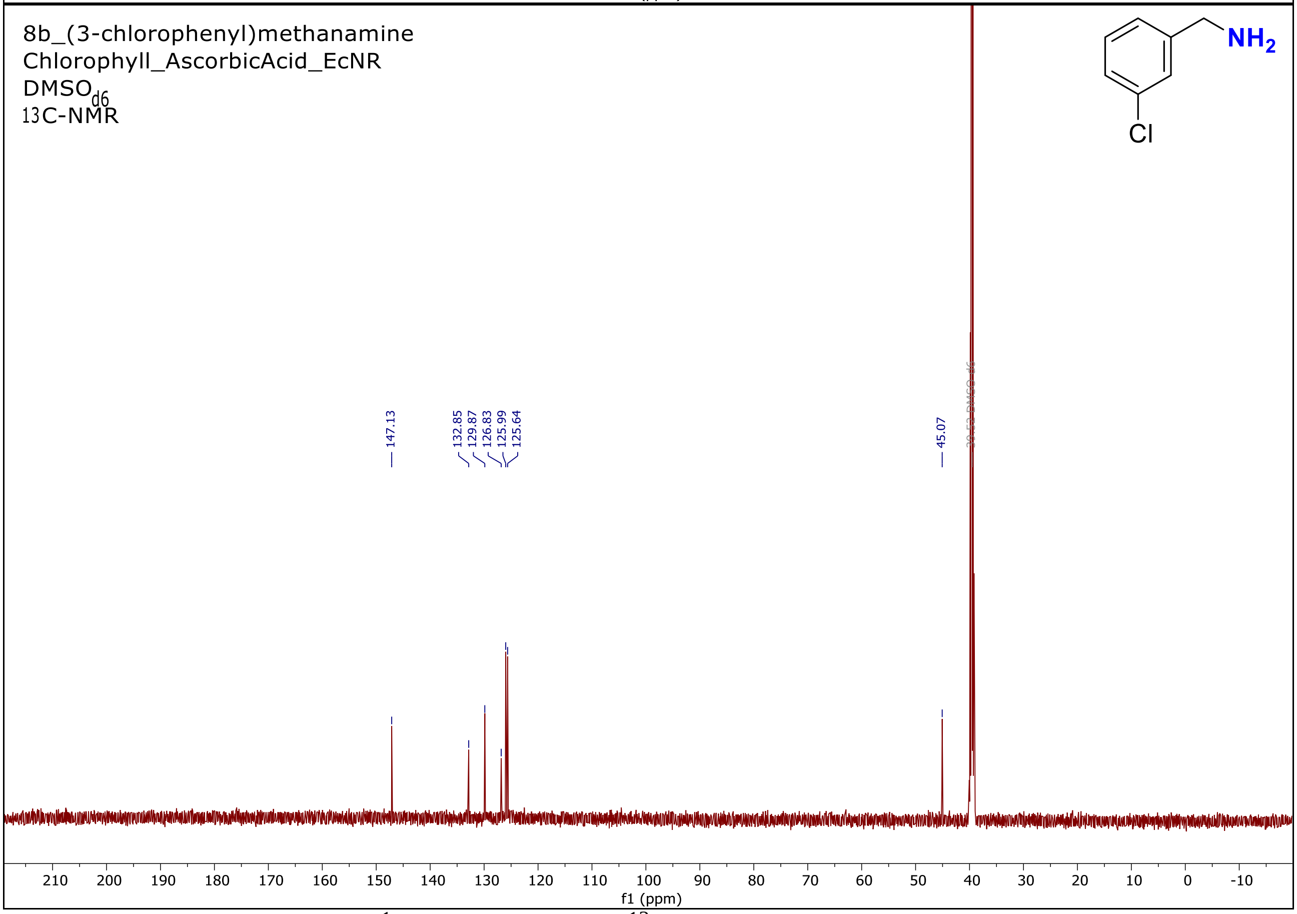

Supplementary Figure 125. ^{1}H-NMR (top) and ^{13}C-NMR (bottom) spectrum of the photoenzymatic product **79**.

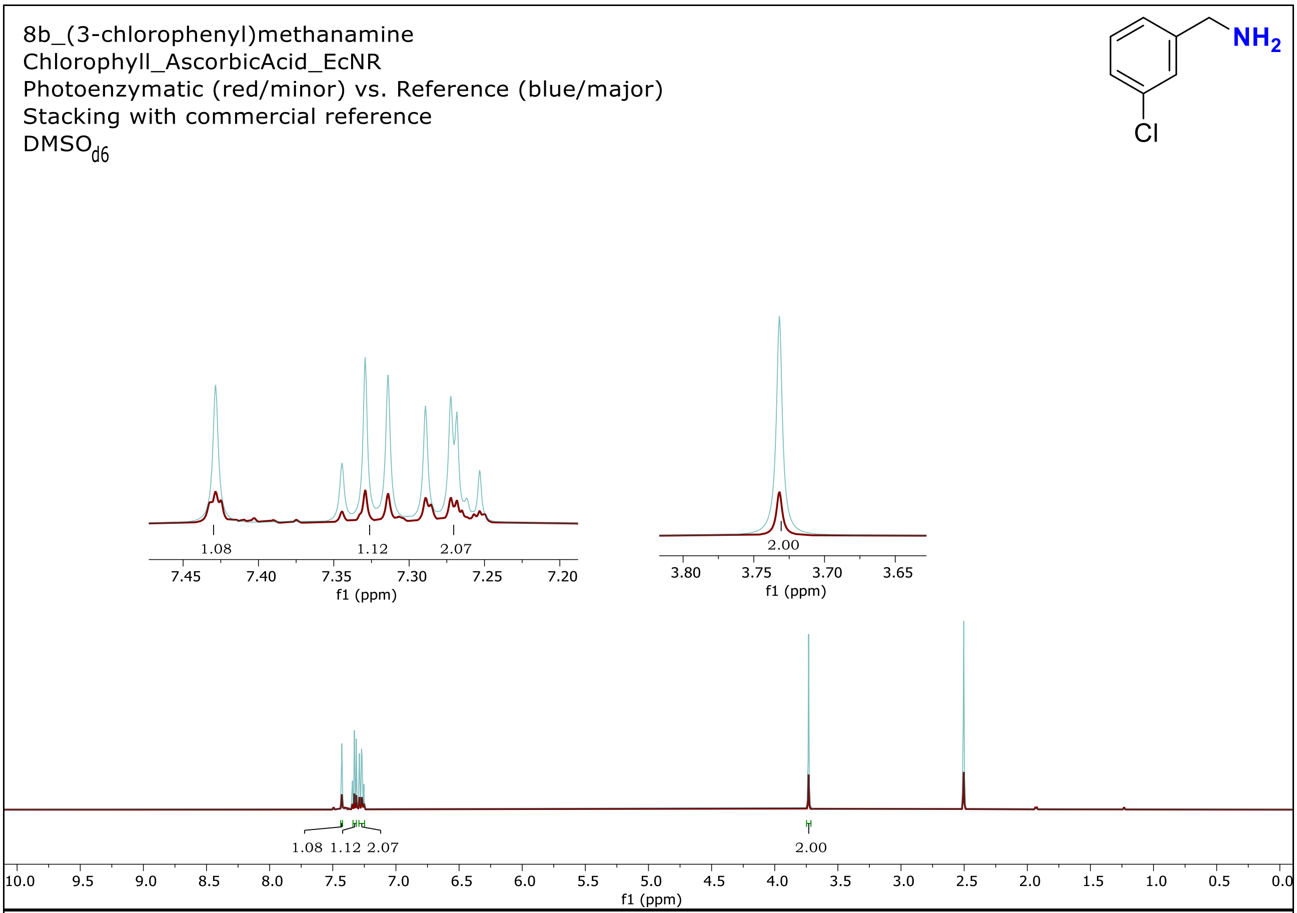

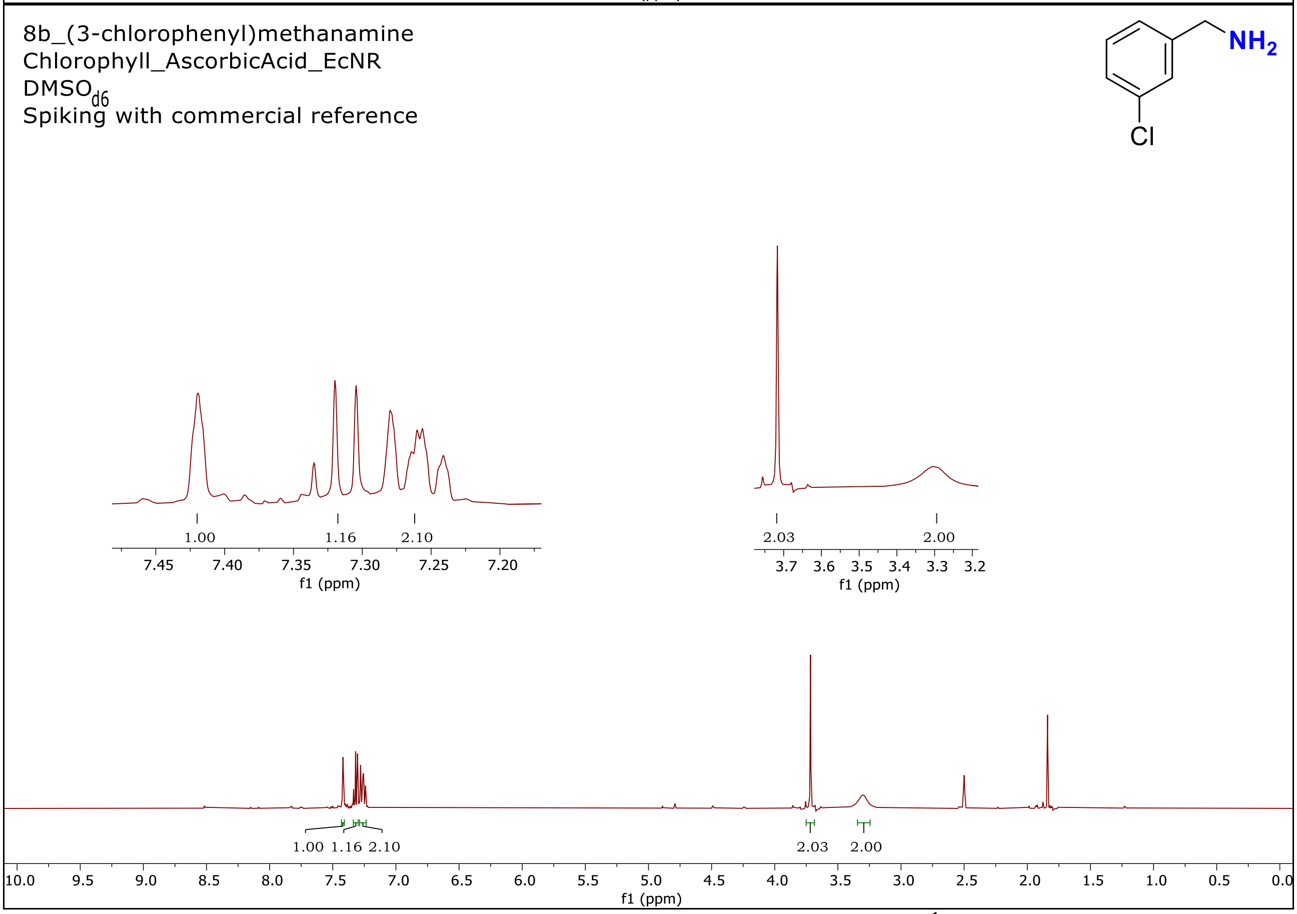

Supplementary Figure 126. Stacking (top) and spiking (bottom) ^{1}H-NMR spectrum with a commercial reference of the photoenzymatic product **79**.

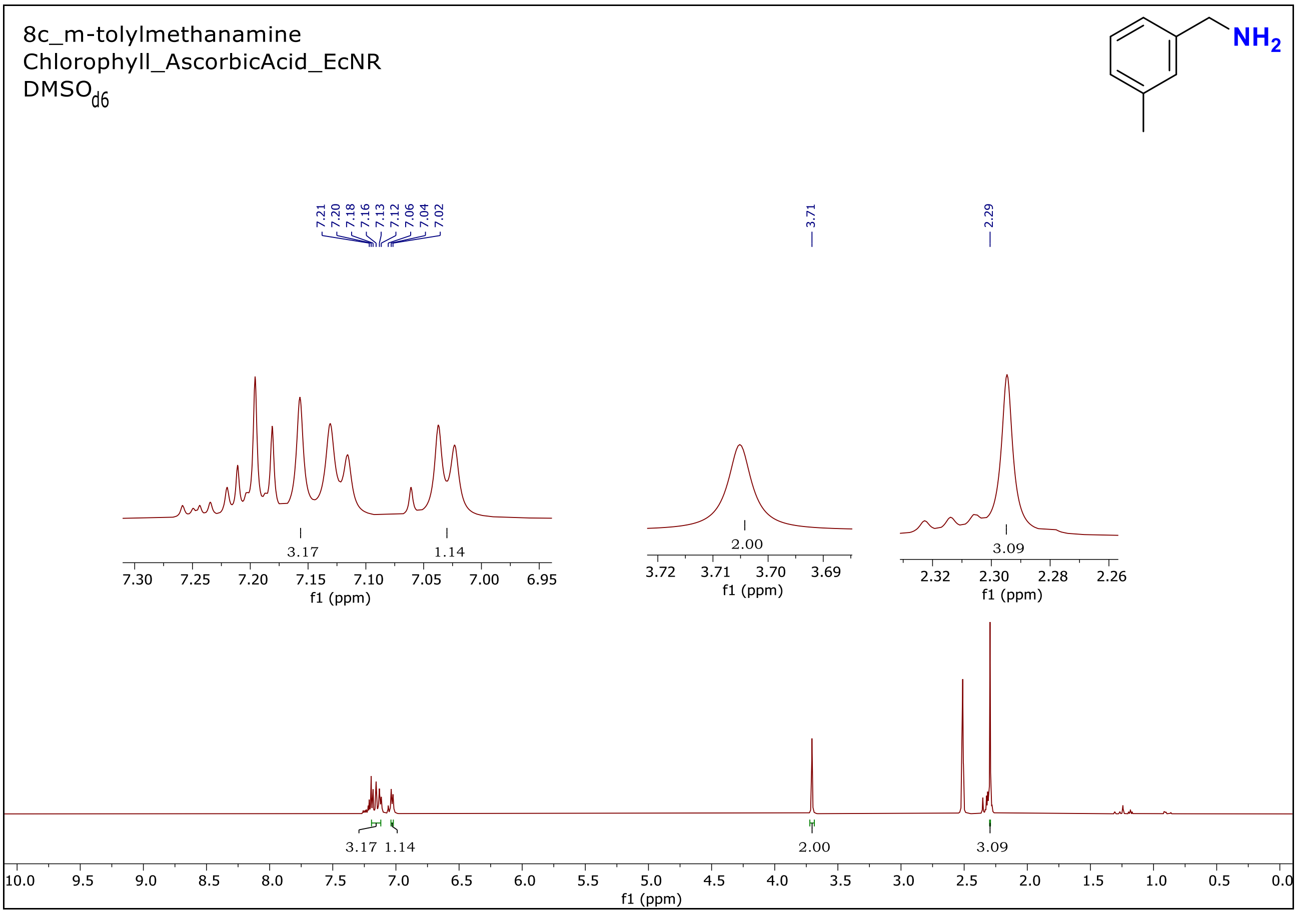

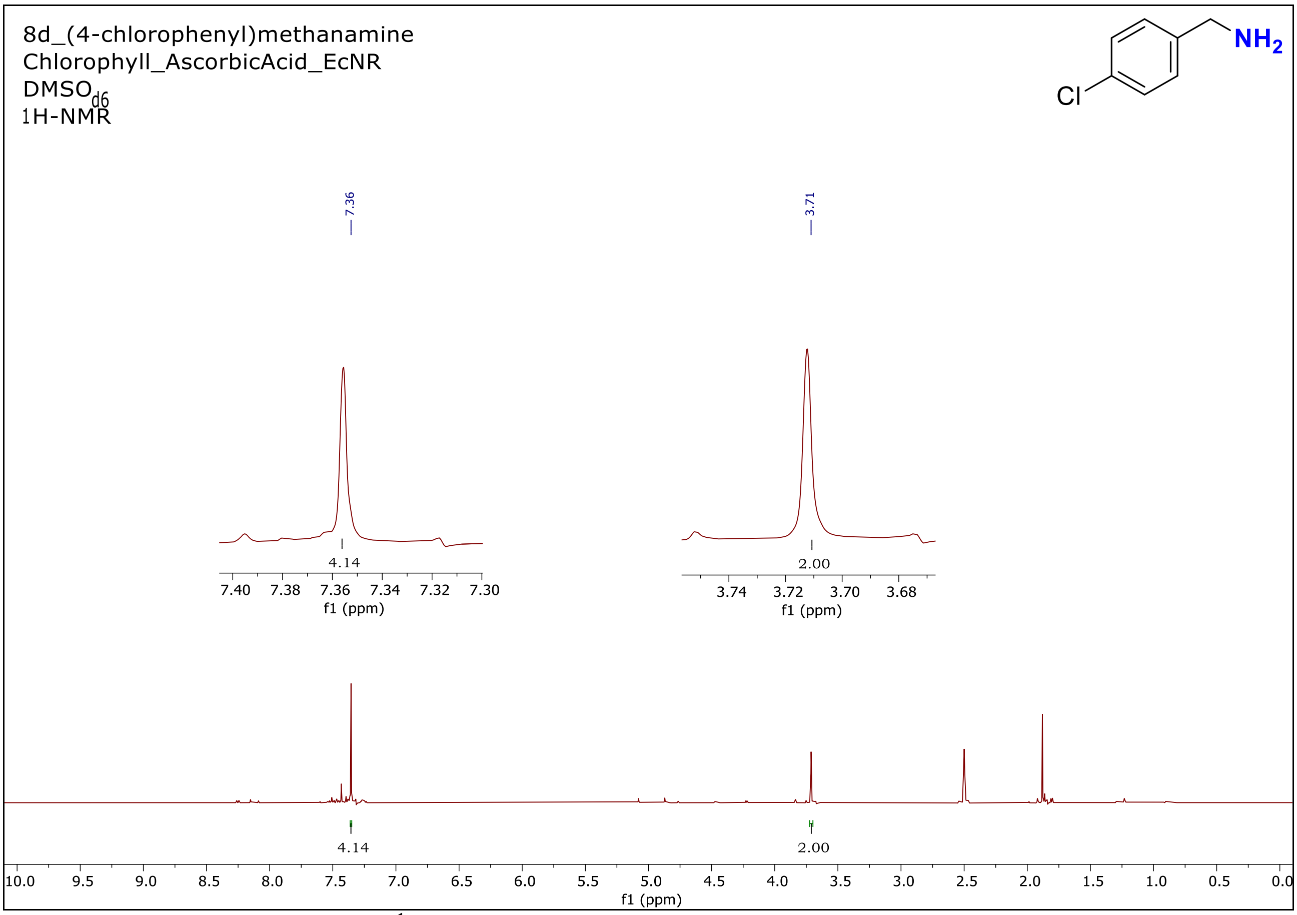

Supplementary Figure 127. ^{1}H-NMR spectrum of the photoenzymatic products **80** (top) and **81** (bottom).

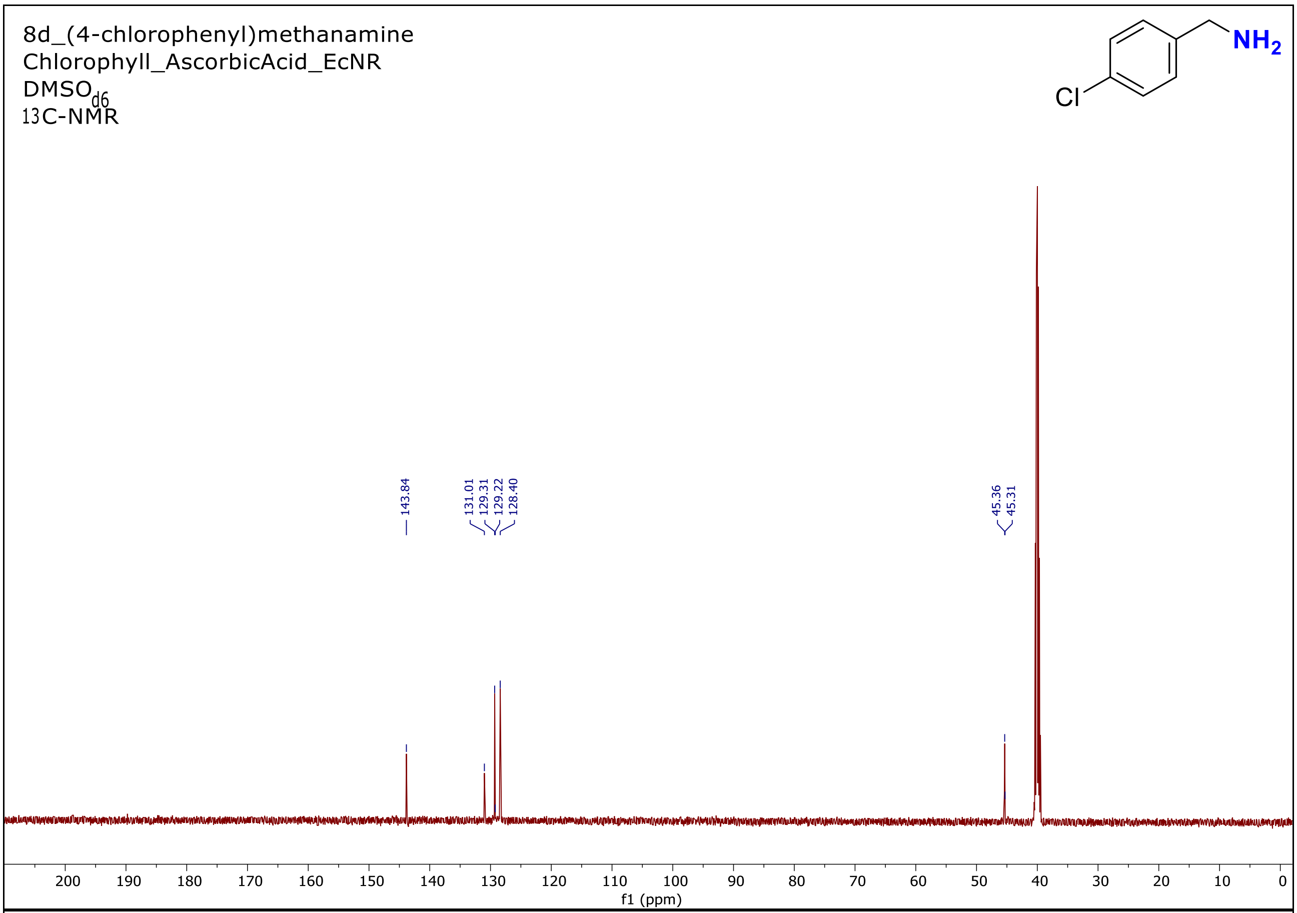

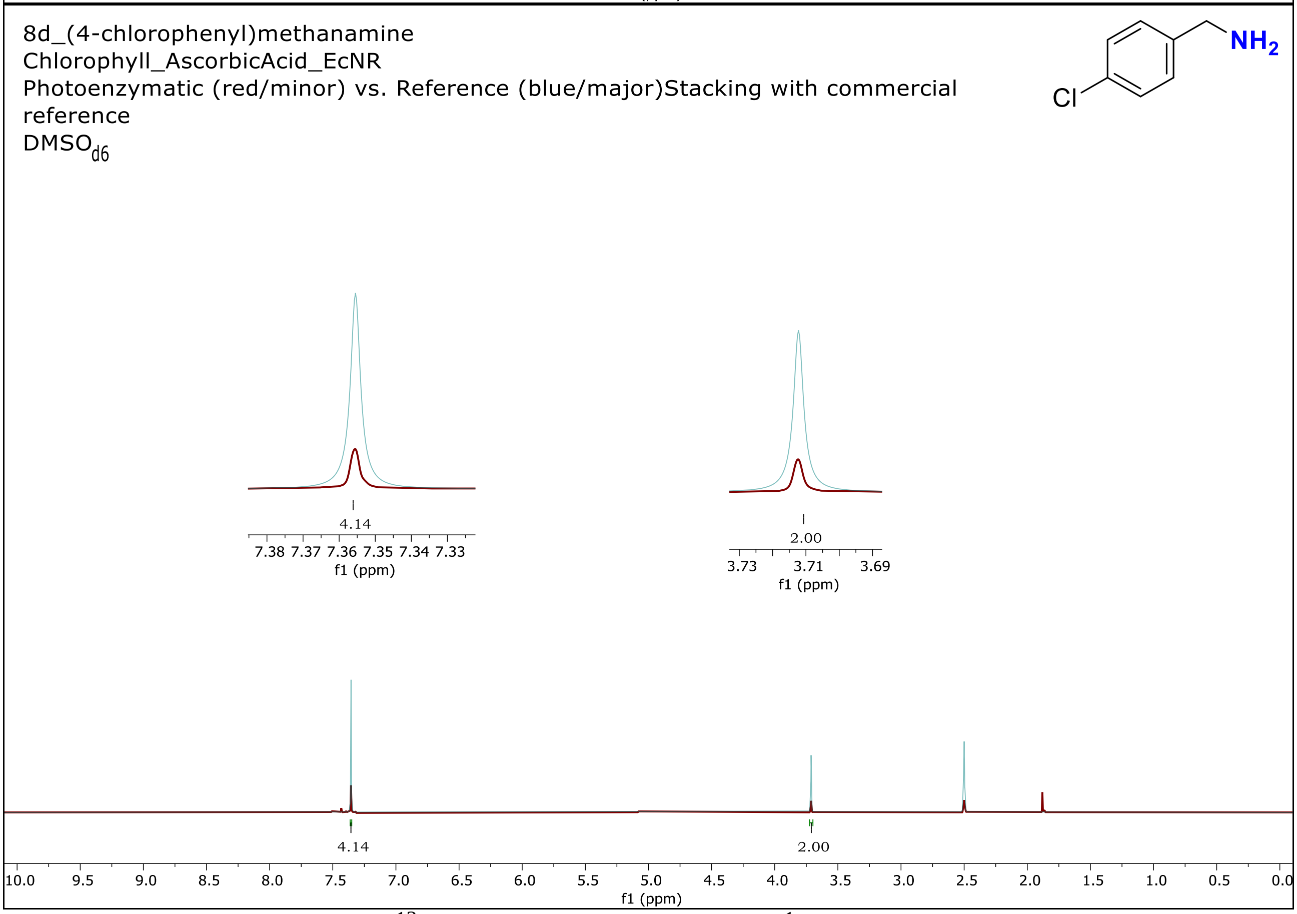

Supplementary Figure 128. ^{13}C-NMR (top) and stacking ^{1}H-NMR spectrum with a commercial reference (top) of the photoenzymatic product **81**.

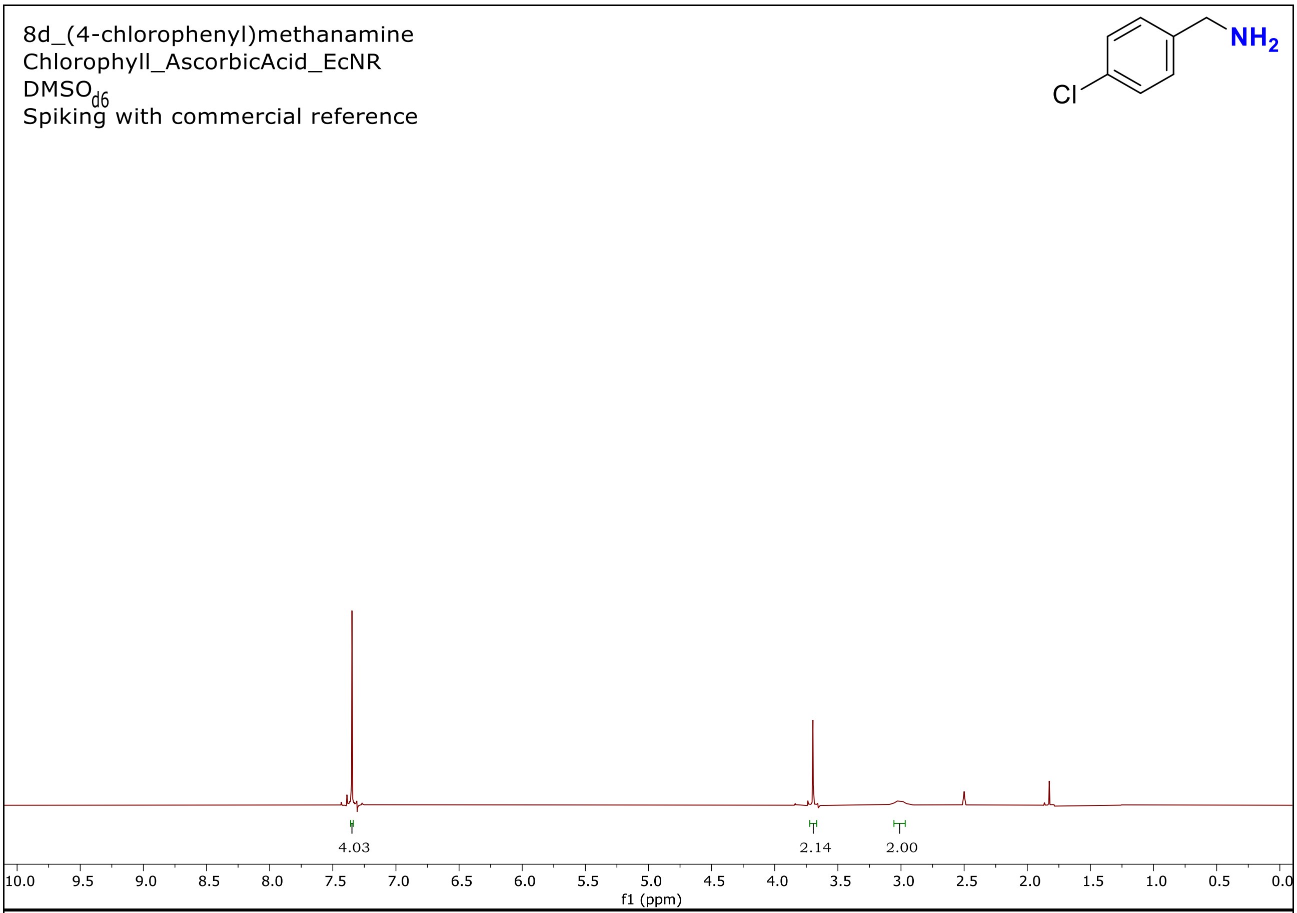

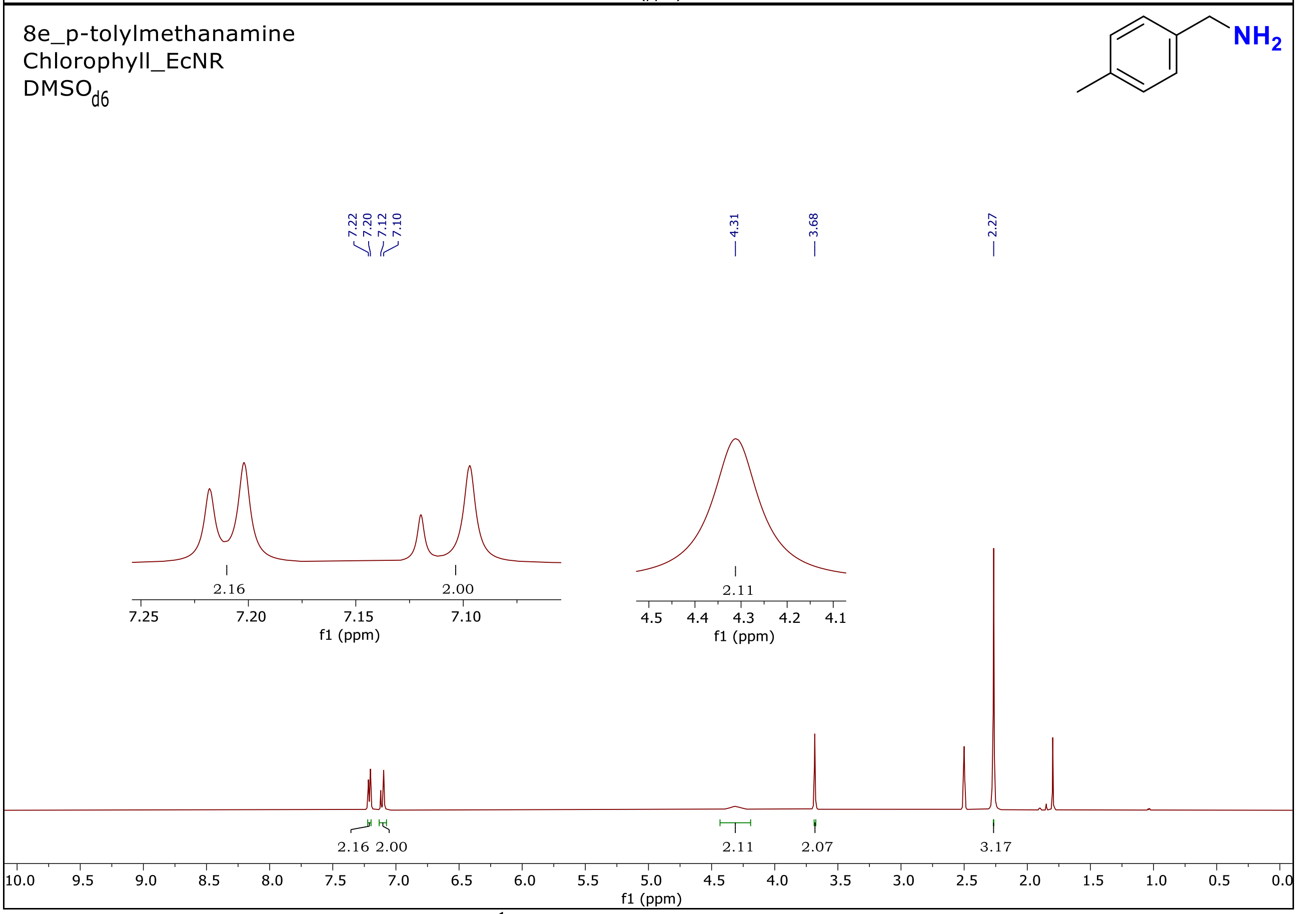

Supplementary Figure 129. Spiking ^{1}H-NMR with a commercial reference of the photoenzymatic product **81** (top) and ^{1}H-NMR of the photoenzymatic product **82** (bottom).

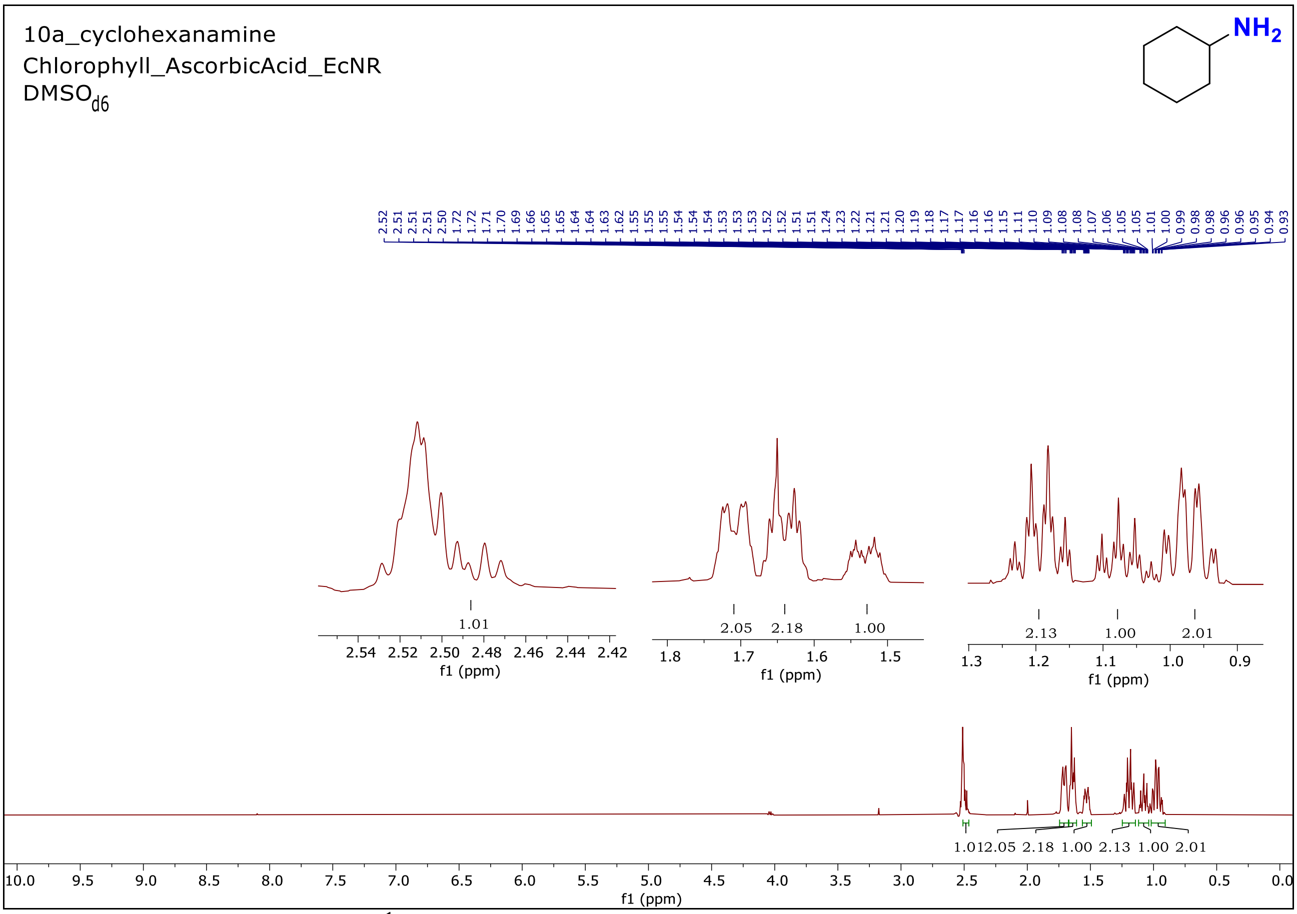

Supplementary Figure 130. ^{1}H-NMR of the photoenzymatic product **83**.

Supplementary References

1. Nguyen-Tran, H. H., Zheng, G. W., Qian, X. H. & Xu, J. H. Highly selective and controllable synthesis of arylhydroxylamines by the reduction of nitroarenes with an electron-withdrawing group using a new nitroreductase BaNTR1. *Chem. Commun.* **50**, 2861–2864 (2014).
2. Luján, A. P., Bhat, F., Saravanan, T. & Poelarends, G. J. Chemo- and enantioselective photoenzymatic ketone reductions using a promiscuous flavin-dependent nitroreductase. *ChemCatChem* **202200043**, 1–6 (2022).
3. Isayev, O., Crespo-Hernández, C. E., Gorb, L., Hill, F. C. & Leszczynski, J. In silico structure-function analysis of E. cloacae nitroreductase. *Proteins Struct. Funct. Bioinforma.* **80**, 2728–2741 (2012).
4. Pitsawong, W., Haynes, C. A., Koder, R. L., Rodgers, D. W. & Miller, A. F. Mechanism-informed refinement reveals altered substrate-binding mode for catalytically competent nitroreductase. *Structure* **25**, 978-987.e4 (2017).
5. Lovering, A. L., Hyde, E. I., Searle, P. F. & White, S. A. The structure of Escherichia coli nitroreductase complexed with nicotinic acid: Three crystal forms at 1.7 Å, 1.8 Å and 2.4 Å resolution. *J. Mol. Biol.* **309**, 203–213 (2001).
6. Johansson, E., Parkinson, G. N., Denny, W. A. & Neidle, S. Studies on the nitroreductase prodrug-activating system. Crystal structures of complexes with the inhibitor dicoumarol and dinitrobenzamide prodrugs and of the enzyme active form. *J. Med. Chem.* **46**, 4009–4020 (2003).
7. Gao, X., Turek-Herman, J. R., Choi, Y. J., Cohen, R. D. & Hyster, T. K. Photoenzymatic synthesis of α-tertiary amines by engineered flavin-dependent 'ene'-reductases. *J. Am. Chem. Soc.* **143**, 19643–19647 (2021).
8. Sandoval, B. A., Kurtoic, S. I., Chung, M. M., Biegasiewicz, K. F. & Hyster, T. K. Photoenzymatic catalysis enables radical-mediated ketone reduction in ene-reductases. *Angew. Chem. Int. Ed.* **58**, 8714–8718 (2019).
9. Sandoval, B. A., Meichan, A. J. & Hyster, T. K. Enantioselective hydrogen atom transfer: discovery of catalytic promiscuity in flavin-dependent 'ene'-reductases. *J. Am. Chem. Soc.* **139**, 11313–11316 (2017).
10. Sandoval, B. A. *et al.* Photoenzymatic reductions enabled by direct excitation of flavin-dependent 'ene'-reductases. *J. Am. Chem. Soc.* **143**, 1735–1739 (2021).
11. Race, P. R. *et al.* Kinetic and structural characterisation of Escherichia coli nitroreductase mutants showing improved efficacy for the prodrug substrate CB1954. *J. Mol. Biol.* **368**, 481–492 (2007).
12. Yang, J., Bai, J., Qu, M., Xie, B. & Yang, Q. Biochemical characteristics of a nitroreductase with diverse substrate specificity from *Streptomyces mirabilis* DUT001. *Biotechnol. Appl. Biochem.* **1**, 1–10 (2018).
13. Enamine Ltd. Spectrum ID Z57127905.
14. John Wiley & Sons, I. Spectrum ID CC-03-C_SPC-16427.
15. Nozawa-Kumada, K., Abe, E., Ito, S., Shigeno, M. & Kondo, Y. Super electron donor-mediated reductive transformation of nitrobenzenes: A novel strategy to synthesize azobenzenes and phenazines. *Org. Biomol. Chem.* **16**, 3095–3098 (2018).
16. Udumula, V. *et al.* Dual optimization approach to bimetallic nanoparticle catalysis: Impact of M1/M2 ratio and supporting polymer structure on reactivity. *ACS Catal.* **5**, 3457–3462 (2015).
17. Yang, H., Li, Y., Jiang, M., Wang, J. & Fu, H. General copper-catalyzed transformations of functional groups from arylboronic acids in water. *Chem. Eur. J.* **17**, 5652–5660 (2011).

18. Enamine Ltd. Spectrum ID EN300-19100.
19. Enamine Ltd. Spectrum ID EN300-19099.
20. Enamine Ltd. Spectrum ID EN300-17968.
21. John Wiley & Sons, I. Spectrum ID CC-02-H_NMR-2039.
22. Enamine Ltd. Spectrum ID EN300-20241.
23. National Institute of Advanced Industrial Science and Technology. Spectrum ID WHSP49413.
24. Rao, H., Fu, H., Jiang, Y. & Zhao, Y. Easy copper-catalyzed synthesis of primary aromatic amines by coupling aromatic boronic acids with aqueous ammonia at room temperature. *Angew. Chem. Int. Ed.* **48**, 1114–1116 (2009).
25. Enamine Ltd. Spectrum ID Z104472524.
26. Wang, Y., Ouyang, G., Zhang, J. & Wang, Z. A DNA-templated catalyst: The preparation of metal-DNA nanohybrids and their application in organic reactions. *Chem. Commun.* **46**, 7912–7914 (2010).
27. John Wiley & Sons, I. Spectrum ID CNCC-56295-365B.
28. Czaplik, W. M., Neudörfl, J. M. & von Wangelin, A. J. On the quantitative recycling of Raney–Nickel catalysts on a lab-scale. *Green Chem.* **9**, 1163–1165 (2007).
29. Enamine Ltd. Spectrum ID EN300-20837.
30. Enamine Ltd. Spectrum ID Z285150732.
31. Enamine Ltd. Spectrum ID EN300-04981.
32. Enamine Ltd. Spectrum ID Z273611666.
33. Enamine Ltd. Spectrum ID Z385438802.
34. Enamine Ltd. Spectrum ID EN300-43328.
35. Sasaki, E. *et al.* Highly sensitive near-infrared fluorescent probes for nitric oxide and their application to isolated organs. *J. Am. Chem. Soc.* **127**, 3684–3685 (2005).
36. Chang, C. F. & Liu, S. T. Catalytic oxidation of anilines into azoxybenzenes on mesoporous silicas containing cobalt oxide. *J. Mol. Catal. A Chem.* **299**, 121–126 (2009).
37. Technology, N. I. of A. I. S. and. Spectrum ID WHPM03369.
38. Enamine Ltd. Spectrum ID EN300-95260.nmr.y202.
39. Marsais, F. & Queguiner, G. Review on the metallation of π-deficient heteroaromatic compounds. *Tetrahedron* **39**, 2009–2021 (1983).
40. Enamine Ltd. Spectrum ID EN300-56505.
41. Jih, R. H., Das, A. R., Chia, W. Y., Huang, J. J. & Hsu, M. H. 1,2-Eliminations in a novel reductive coupling of nitroarenes to give azoxy arenes by sodium bis(trimethylsilyl)amide. *Org. Lett.* **7**, 3211–3214 (2005).
42. Technology, N. I. of A. I. S. and. Spectrum ID WCDS04778.
43. Thorwirth, R., Bernhardt, F., Stolle, A., Ondruschka, B. & Asghari, J. Switchable selectivity during oxidation of anilines in a ball mill. *Chem. Eur. J.* **16**, 13236–13242 (2010).
44. Chen, Y. F., Chen, J., Lin, L. J. & Chuang, G. J. Synthesis of azoxybenzenes by reductive dimerization of nitrosobenzene. *J. Org. Chem.* **82**, 11626–11630 (2017).
45. Yan, Z. *et al.* Tandem selective reduction of nitroarenes catalyzed by palladium nanoclusters. *Green Chem.* **22**, 1301–1307 (2020).

46. Sakai, N., Fujii, K., Nabeshima, S., Ikeda, R. & Konakahara, T. Highly selective conversion of nitrobenzenes using a simple reducing system combined with a trivalent indium salt and a hydrosilane. *Chem. Commun.* **46**, 3173–3175 (2010).
47. Chang, L., Li, J., Wu, N. & Cheng, X. Chemoselective electrochemical reduction of nitroarenes with gaseous ammonia. *Org. Biomol. Chem.* **19**, 2468–2472 (2021).
48. Morrison, J., Wan, P., Corrie, J. E. T. & Munasinghe, V. R. N. Chemistry of photogenerated α-hydroxy-p-nitrobenzyl carbanions in aqueous solution: Protonation vs. disproportionation. *Can. J. Chem.* **81**, 586–597 (2003).
49. Ke, L. *et al.* Catalytic selective oxidative coupling of secondary N-alkylanilines: An approach to azoxyarene. *Org. Lett.* **21**, 4008–4013 (2019).
50. da Silva, A. G. M. *et al.* Plasmonic nanorattles as next-generation catalysts for surface plasmon resonance-mediated oxidations promoted by activated oxygen. *Angew. Chem. Int. Ed.* **55**, 7111–7115 (2016).
51. Pothula, K., Tang, L., Zha, Z. & Wang, Z. Bismuth nanoparticles: an efficient catalyst for reductive coupling of nitroarenes to azo-compounds. *RSC Adv.* **5**, 83144–83148 (2015).
52. Enamine Ltd. Spectrum ID EN300-17405.
53. Kageyama, Y. & Murata, S. Oxidative formation of thiolesters in a model system of the pyruvate dehydrogenase complex. *J. Org. Chem.* **70**, 3140–3147 (2005).
54. Guisado, C., Waterhouse, J. E., Price, W. S., Jorgensen, R. & Miller, A. D. The facile preparation of primary and secondary amines. *Society* 1049–1057 (2005).
55. Enamine Ltd. Spectrum ID EN300-17861.
56. Sigma-Aldrich & Advanced Chemistry Development, I. Spectrum ID 340847.h.
57. Enamine Ltd. Spectrum ID EN300-41636.
58. Enamine Ltd. Spectrum ID EN300-18739.
59. Enamine Ltd. Spectrum ID EN300-17862.
60. Sigma-Aldrich & Advanced Chemistry Development, I. Spectrum ID 189308.h.
61. Sigma-Aldrich & John Wiley & Sons, I. Spectrum ID SLSH_000937.
62. Pradhan, P. K., Dey, S., Jaisankar, P. & Giri, V. S. Fe-HCl: An efficient reagent for deprotection of oximes as well as selective oxidative hydrolysis of nitroalkenes and nitroalkanes to ketones. *Synth. Commun.* **35**, 913–922 (2005).
63. Nixon, T. D., Whittlesey, M. K. & Williams, J. M. J. Ruthenium-catalysed transfer hydrogenation reactions with dimethylamine borane. *Tetrahedron Lett.* **52**, 6652–6654 (2011).
64. Enamine Ltd. Spectrum ID EN300-16217.
65. Enamine Ltd. Spectrum ID Z57102467.
66. Enamine Ltd. Spectrum ID EN300-16214.
67. Enamine Ltd. Spectrum ID EN300-16222.
68. Bio-Rad Laboratories. Spectrum ID HB_07656.
